# Multi-Omic Profiling of Macrophages Treated with Phospholipids Containing Omega-3 and Omega-6 Fatty Acids Reveals Complex Immunomodulatory Adaptations at Protein, Lipid and Metabolic Levels

**DOI:** 10.3390/ijms23042139

**Published:** 2022-02-15

**Authors:** Tatiana Maurício, Susana Aveiro, Sofia Guedes, Diana Lopes, Tânia Melo, Bruno M. Neves, Rosário Domingues, Pedro Domingues

**Affiliations:** 1 Mass Spectrometry Centre, LAQV-REQUIMTE, Department of Chemistry, Santiago University Campus, University of Aveiro, 3810-193 Aveiro, Portugal; tatianascm97@ua.pt (T.M.); s.aveiro@ua.pt (S.A.); sguedes@ua.pt (S.G.); dianasalzedaslopes@ua.pt (D.L.); taniamelo@ua.pt (T.M.); mrd@ua.pt (R.D.); 2 GreenCoLab-Green Ocean Association, University of Algarve, 8005-139 Faro, Portugal; 3 CESAM-Centre for Environmental and Marine Studies, Department of Chemistry, Santiago University Campus, University of Aveiro, 3810-193 Aveiro, Portugal; 4 Department of Medical Sciences and Institute of Biomedicine—iBiMED, University of Aveiro, 3810-193 Aveiro, Portugal; bruno.neves@ua.pt

**Keywords:** macrophage, omega-3 phospholipid, omega-6 phospholipid, LPS, proteomics, lipidomics, metabolomics

## Abstract

In recent years, several studies have demonstrated that polyunsaturated fatty acids have strong immunomodulatory properties, altering several functions of macrophages. In the present work, we sought to provide a multi-omic approach combining the analysis of the lipidome, the proteome, and the metabolome of RAW 264.7 macrophages supplemented with phospholipids containing omega-3 (PC 18:0/22:6; ω3-PC) or omega-6 (PC 18:0/20:4; ω6-PC) fatty acids, alone and in the presence of lipopolysaccharide (LPS). Supplementation of macrophages with ω3 and ω6 phospholipids plus LPS produced a significant reprogramming of the proteome of macrophages and amplified the immune response; it also promoted the expression of anti-inflammatory proteins (e.g., pleckstrin). Supplementation with the ω3-PC and ω6-PC induced significant changes in the lipidome, with a marked increase in lipid species linked to the inflammatory response, attributed to several pro-inflammatory signalling pathways (e.g., LPCs) but also to the pro-resolving effect of inflammation (e.g., PIs). Finally, the metabolomic analysis demonstrated that supplementation with ω3-PC and ω6-PC induced the expression of several metabolites with a pronounced inflammatory and anti-inflammatory effect (e.g., succinate). Overall, our data show that supplementation of macrophages with ω3-PC and ω6-PC effectively modulates the lipidome, proteome, and metabolome of these immune cells, affecting several metabolic pathways involved in the immune response that are triggered by inflammation.

## 1. Introduction

Macrophages are specialised cells of the innate immune system and the mononuclear phagocyte system and are strongly involved in all stages of inflammation [1]. Macrophages display a wide range of functions, such as (1) recognition of pathogen-associated molecular patterns (PAMPs) or danger-associated molecular patterns (DAMPs) [2,3]; (2) antigen presentation; (3) phagocytosis of microorganisms, debris, and apoptotic cells; and (4) the secretion of several molecules, including a variety of cytokines, lipid mediators [1,4] and the production of reactive oxygen species (ROS), and reactive nitrogen species (RSN), such as nitric oxide (NO), to eliminate phagocytosed harmful organisms [3]. Two major populations of macrophages with distinct characteristics and functions have been established, depending on the microenvironmental conditions that drive their polarisation: M1-like macrophages, also known as classically activated or pro-inflammatory macrophages and M2-like macrophages, or alternatively activated macrophages with a predominant anti-inflammatory role [4,5,6]. It is known that a balance between M1-like and M2-like macrophages is crucial for the resolution of inflammation and to ensure homeostasis [7].

Dietary intake of polyunsaturated fatty acids (PUFAs), mainly omega-3 and omega-6 FAs, is thought to modulate the functions of the innate and adaptive immune system with an increased ratio of omega-6/omega-3 PUFAs associated with the development or aggravation of inflammatory conditions [8,9]. PUFAs have been demonstrated to impact the functions of epithelial cells, dendritic cells, macrophages, neutrophils, and T and B cells [2,10]. Regarding the effects on macrophages, they mainly depend on direct and indirect interferences with inflammation-related signalling pathways [11]. These dietary compounds have been the subjects of numerous studies aimed to evaluate their immunomodulatory potential, particularly in the context of attenuating inflammation in chronic inflammatory-related disorders (reviewed in [12]).

The omega-3 PUFAs family includes three main acids, α-linolenic acid (ALA), docosahexaenoic acid (DHA), and eicosapentaenoic acid (EPA) [8]. Omega-3 FAs and their respective metabolites may down-regulate the secretion of pro-inflammatory cytokines by macrophages and impair the activation of the NLRP3 inflammasome as well as the production of reactive oxygen species, while increasing phagocytic activity [13,14,15]. Omega-3 FAs have also been demonstrated to promote the polarisation of macrophages into the M2-like anti-inflammatory phenotype [13,16]. These immunomodulatory effects have been attributed to the regulation of key intracellular signalling cascades, such as the NF-κB and PPAR-γ pathways or the GPR-120-mediated ERK activation [17,18,19,20]. These data indicate that omega-3 FAs may be useful in repressing macrophage-induced tissue inflammation in many chronic inflammatory diseases.

Omega-6 fatty acids include linoleic acid (LA), γ-linolenic acid (GLA), and most importantly, arachidonic acid (AA) [8,21]. These compounds are generally associated with pro-inflammatory action and are linked to several chronic inflammatory disorders [22], although it has recently been demonstrated that omega-6 fatty acids may have an anti-inflammatory effect [23]. Similarly to omega-3 fatty acids, omega-6 FAs are also associated with multiple effects on immune cells, particularly on macrophages [8]. Supplementation with omega-6 FAs, in particular LA and AA, is classically described to enhance the macrophage production of inflammatory cytokines, such as IL-6 and TNF-a and the production of ROS, as well as their phagocytic capacity [24,25,26,27]. However, there is increasing evidence that omega-6 fatty acids and their derivatives may also exert anti-inflammatory effects. For example, it has been demonstrated in vitro that LA promotes a decrease in macrophage production of IL-6 and TNF-α while increasing the levels of the anti-inflammatory cytokine interleukin 10 [24]. Additionally, AA and LA can be metabolised to pro-resolving lipoxins and CYP450-derived oxylipins, and the omega-6 PUFAs dihomo-γ-linolenic acid (DGLA) and adrenic acid (AdA) could compete with AA for metabolisation, reducing the formation of pro-inflammatory mediators [28]. Recently, AdA has been demonstrated to block in vivo the production of LTB4 by neutrophils and to enhance macrophage efferocytosis of apoptotic neutrophils in an in vivo murine model of arthritis [29]. Finally, several meta-analysis studies report the beneficial effects of dietary intake of omega-6 PUFAs, such as LA [30,31].

Therefore, the dichotomous classification of omega-3 and omega-6 PUFAs as being exclusively anti- and pro-inflammatory, respectively, is too simplistic, indicating that further studies with broader and integrative approaches are needed to shed light on the effects of these bioactive lipids. For example, studies simultaneously describing the impact of omega-3 and omega-6 fatty acids on lipids, proteins, and metabolites remodelling in macrophages are still scarce.

Thus, with this study, we sought to provide a multi-omic analysis, involving lipidomics, proteomics, and metabolomics approaches based on a liquid chromatography-mass spectrometry (LC-MS) analysis of cell extracts from the RAW 264.7 macrophage-like cell line exposed to phospholipids containing omega-3 and omega-6 fatty acids. The effects of FAs were studied on resting macrophages and M1-like pro-inflammatory macrophages polarised by exposure to LPS.

## 2. Results

### 2.1. Impact of Omega-3 and Omega-6 PCs on Macrophage Viability and LPS-Triggered Nitric Oxide Production

**Figure 1:**
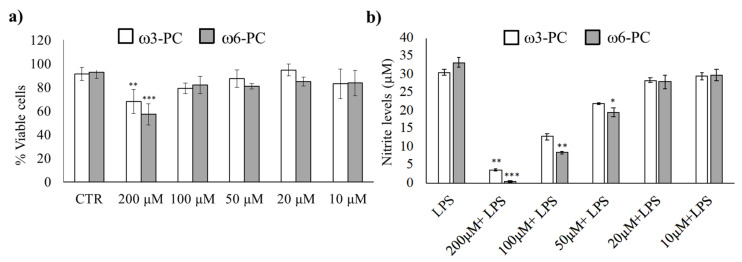
Impact of ω3-PC and ω6-PC on macrophage viability and LPS-triggered nitric oxide production (**a**). Raw 264.7 macrophages were incubated with 10–200 μM of ω3-PC and ω6-PC for 24 h. (*n* = 4) and cell viability was assessed by the resazurin reduction assay (**b**). Raw 264.7 macrophages were incubated with 0–200 μM of ω3-PC and ω6-PC plus LPS for 24 h and the levels of nitrites in the culture medium were then determined by performing the Griess assay (*n* = 3). * *p* < 0.05, ** *p* < 0.01, *** *p* < 0.001, compared with the control group (CTRL). Error bars represented as mean ± SD.

We first determined the working concentration of omega-3 (ω3-PC) and omega-6 (ω6-PC) phospholipids by evaluating their toxicity, (Alamar Blue reduction assay), as well as the anti-inflammatory activity of the compounds (Griess Reaction assay). The results in Figure 1a show that the number of viable cells remained high for concentrations up to 100 μM. Only treatment with 200 μM for both ω3-PC and ω6-PC phospholipids resulted in a significant decrease in cell viability compared with control cells.

The effect of the phospholipids on LPS-induced NO production was then investigated. NO production was suppressed upon treatment with ω3-PC and ω6-PC in a dose-dependent manner (Figure 1b). A significant decrease in NO production was observed with 100 and 200 μM of ω3-PC and with 50, 100, and 200 μM of ω6-PC. Therefore, to prevent the possible influence of unspecific mechanisms triggered by cytotoxicity, we used the subtoxic concentration of 100 μM for both ω3-PC and ω6-PC in the following experimental procedures.

### 2.2. Supplementation with Omega-3 and Omega-6 PCs Promotes Macrophage Proteome Noticeable Remodelling

**Figure 2:**
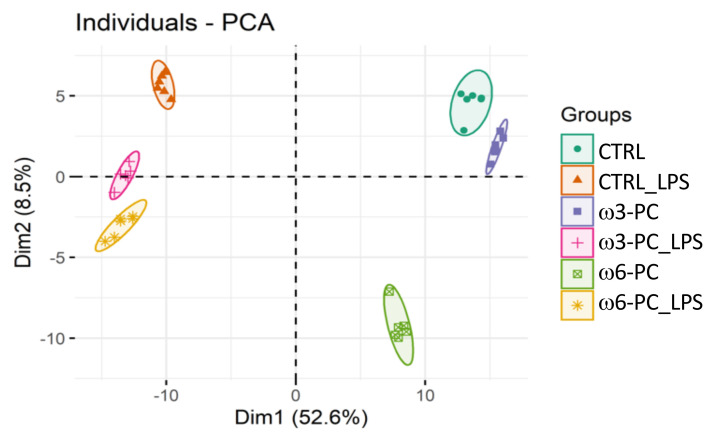
Principal component analysis (PCA) score plot of the proteomic profile, obtained for macrophages incubated with omega-3 and omega-6 phospholipids (ω3-PC and ω6-PC), with omega-3 and omega-6 phospholipids and LPS (ω3-PC_LPS and ω6-PC_LPS) and controls (CTRL and CTRL_LPS) (*n* = 6).

In the proteomic analysis, we identified and semi-quantified a total of 3238 proteins (Supplementary Table S1), of which 228 were involved in lipid metabolism and 461 were involved in the regulation of the immune system (https://www.ebi.ac.uk/QuickGO/ (accessed on 15 June 2021)). The PCA score plot (Figure 2), shows that the six groups studied were well clustered according to changes in the proteomic profile and all samples were well separated by the first two dimensions (Dim1 52.6% and Dim2 8.5%).

Comparing the relative abundance of the proteins identified in macrophages control samples (CTRL) and samples treated with omega-3 (ω3-PC) or omega-6 (ω6-PC) phospholipids, the number of significantly different proteins was 225 and 726, respectively (Table 1). Comparing macrophages samples treated with ω3-PC and ω6-PC revealed that the relative abundance of 887 proteins was significantly different. For the samples treated with LPS, 1854 proteins changed significantly between ω3 and ω3-PC_LPS, and 1256 proteins between ω6-PC and ω6-PC_LPS. Between the ω3-PC_LPS group and the ω6-PC_LPS group, only 526 proteins were significantly different.

**Figure 3:**
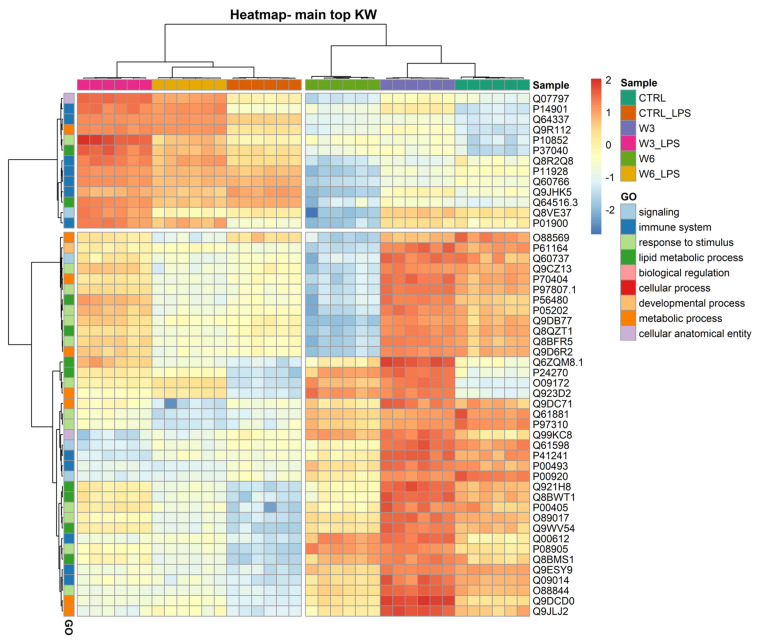
Two-dimensional hierarchical clustering heatmap of the 50 main proteins (lower q values) under the 6 conditions: control (CTRL), control for LPS (CTRL_LPS), omega-3 PC (ω3-PC), omega-3 PC with LPS (ω3-PC_LPS), omega-6 PC (ω6-PC), and omega-6 PC with LPS (ω6-PC_LPS), after the Kruskal–Wallis test. At the top is the dendrogram of the samples, and on the left is the dendrogram of proteins labelled with their generic function according to GO analysis.

The clustering and functions of the 50 most significantly modulated proteins can be visualised in a two-dimensional hierarchical clustering heatmap, as shown in in Figure 3, Supplementary Table S2, in the Supplementary Materials. The dendrogram at the top of the heatmap shows that the samples have been clustered in the first leaf into two groups and are clearly dependent on LPS treatment. The first group (on the left) contains all experimental conditions exposed to LPS and the second group (on the right) includes conditions in which macrophages were not treated with the bacterial PAMP. The dendrogram on the right side of the heatmap shows the proteins annotated according to GO functions. In the first leaf, the proteins were clustered into two groups; the first group contained 13 proteins, seven of which (P14901, Q64337, Q8R2Q8, P11928, Q60766, Q9JHK5, and P01900) are involved in the immune system and are upregulated in response to LPS treatment. It should be noted that the increase triggered by LPS observed in the 13 proteins of this first cluster is higher when the cells were simultaneously exposed to PC, the ω3 phospholipid being the one that caused the most pronounced effects. Exposure to PCs alone caused minimal changes in these proteins. The second group contained 37 proteins, which are mainly involved in lipid metabolic processes and response to a stimulus. The proteins clustering in this group are overall downregulated by LPS treatment independently of the presence of PCs, and strongly upregulated by culture supplementation with the ω3 phospholipid. In this second leaf, a sub-group of 13 proteins (O88569, P61164, Q60737, Q9CZ13, P70404, P97807.1, P56480, P05202, Q9DB77, Q8QZT1, Q8BFR5, and Q9D6R2) is strongly downregulated by ω6 PC either in the presence or absence of LPS. Among the 37 proteins, five (P41241, P00493, Q00612, Q9ESY9, and Q09014) are involved in the immune system. Another sub-group of proteins can be noted in the second leaf of the dendrogram, consisting of only four proteins (Q6ZQM8.1 (lipid metabolic process), P24270 (lipid metabolic process), O09172 (response to stimulus), and Q923D2 (metabolic process)), the expression of which was markedly increased by both ω3 and ω6 phospholipids.

### 2.3. Lipidomics

To characterise the lipid profile of macrophages, identification and quantification of lipid species were performed using high-resolution HILIC-MS/MS. In the lipidomic analysis, we identified and semi-quantified a total of 299 individual polar lipid species, including glycerophospholipids, glycerolipids, sphingolipids, and acyl-carnitines. In total, 13 classes of lipid species have been identified: acyl-carnitines (CAR), di-acylglycerols (DG), ceramides (Cer), hexosylceramides (HexCer), sphingomyelins (SM), phosphatidylcholines (PC), phosphatidylethanolamines (PE), phosphatidylinositols (PI), phosphatidylglycerols (PG), phosphatidylserine (PS), cardiolipins (CL), lysophosphatidylcholine (LPC), and lysophosphatidylethanolamines (LPE). Some classes, such as PC, PE, LPC, and LPE, also include alkylacyl lipids. DG, CAR, Cer, HexCer, SM, LPC, and PC species were identified in the positive ion mode, as [M + H]^+^ ions. In the negative mode, LPE, PE, PI, PG, and PS were identified as [M − H]^−^ ions and CLs as [M − 2H]^2−^ molecular ions. All identified lipid species can be found in Supplementary Table S3.

**Figure 4:**
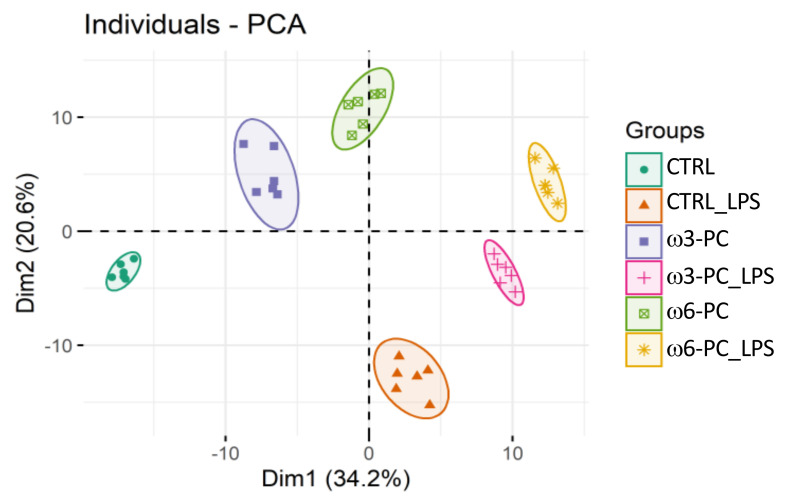
Principal component analysis (PCA) score plot of the phospholipid dataset acquired by LC-MS analysis, for the 6 groups: control (CTRL), control with LPS treatment (CTRL_LPS), ω3-PC, ω3-PC with LPS (ω3-PC _LPS), ω6-PC, and ω6-PC with LPS (ω6-PC _LPS) (*n* = 6).

The PCA analysis of the LC-MS dataset shows the clustering of samples into the six groups (CTRL, CTRL_LPS, ω3-PC, ω3-PC_LPS, ω6-PC, and ω6-PC_LPS), which were well separated with no outliers (Figure 4). A clear distinction can also be observed between groups treated with (CTRL_LPS, ω3-PC_LPS, and ω6-PC_LPS) or without (CTRL, ω3-PC, and ω6-PC) LPS, being well separated along Dim1 (34.2%). 

There were 273 lipid species, whose relative abundance changed significantly between conditions (*q*-value < 0.05) (Supplementary Table S4). The relative abundance of several species belonging to different classes, was significantly altered between conditions, with a *q*-value < 0.05 and a fold change > 1.5, such as in the Cer, CL, DG, LPC, LPE, PC, PG, and PI species (Table 2). Little to no variation was observed when comparing the control group vs. macrophages cultured with PCs (CTRL vs. ω3-PC and CTRL vs. ω6-PC), although ω-6 PC promoted lipidome remodelling to a greater extent compared with the ω-3 phospholipid (three vs. 14 species altered). This stronger effect of ω6-PC on the macrophage lipidome was also observed when comparing cells treated with LPS (CTRL-LPS) with ω3-LPS or ω6-LPS. In macrophages activated by LPS, the culture supplementation with ω6-PC caused significant alterations in 23 lipid species, whereas supplementation with ω3-PC caused differences in only five species (Table 2). When comparing untreated cells with those exposed to LPS (CTRL vs. CTRL_LPS), 44 lipids were significantly modulated, mainly including the upregulation of Cer, CL, DG, PI, LPE, and LPC. 

### 2.4. Metabolomics

LC-MS based untargeted metabolomics provides in-depth profiling of the macrophage metabolome, revealing changes in metabolite abundance upon exogenous stimuli. Our analysis allowed us to identify and quantify a total of 103 species of metabolites, detailed in Supplementary Table S5.

**Figure 5:**
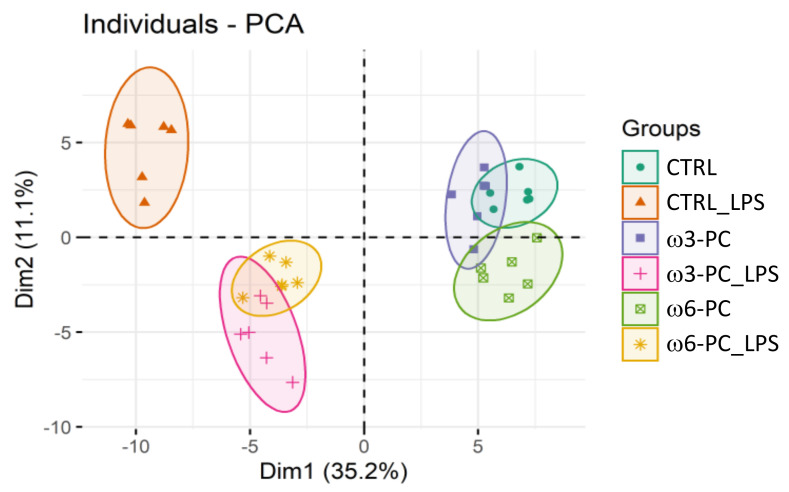
Principal component analysis (PCA) score plot of the metabolite species dataset acquired by LC-MS analysis for the 6 groups: control (CTRL), control for LPS (CTRL_LPS), ω3-PC, ω3-PC with LPS (ω3-PC _LPS), ω6-PC (ω6-PC), and ω6-PC with LPS (ω6-PC_LPS) (*n* = 6).

The PCA analysis showed the clustering of conditions into three main groups: macrophages untreated or cultured with PC (CTRL; ω3-PC; ω6-PC), macrophages treated with LPS (CTRL_LPS), and cells cultured with PC and treated with LPS (ω3-PC_LPS, ω6-PC_LPS) (Figure 5). As observed for the impact on the lipidome, PC has little effect on the metabolome of macrophage, with ω6-PC again having a more pronounced effect than ω3-PC. The activation of macrophages with LPS had a considerable impact on the metabolome compared to untreated cells (main discriminant component Dim1, 35.2%) and the concomitant culture with PCs notably modifies these alterations triggered by LPS.

**Figure 6:**
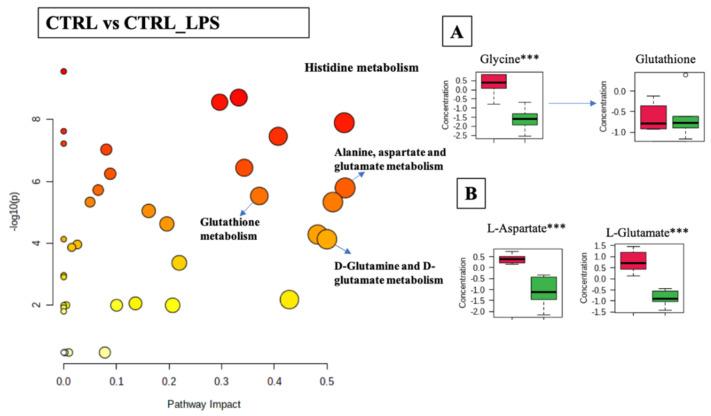
Pathway analysis associated with CTRL or CTRL_LPS of the RAW 264.7 cell line (log(*p*) values from pathway enrichment analysis vs. values of the pathway impact). (**A**) Summary of relevant nodes in the pathway associated with the glutathione metabolism. (**B**) Summary of relevant nodes in the pathway associated with the D-glutamine and D-glutamate metabolism. Red boxplots-CTRL, green boxplots-CTRL_LPS. *** *p* < 0.001.

Differential analysis of cellular metabolites in the comparison of CTRL and CTRL-LPS was associated with 33 metabolic pathways (FDR < 0.05) including: histidine metabolism (*p*-value  <  0.001, FDR < 0.001); alanine, aspartate, and glutamate metabolism (*p*-value  <  0.001, FDR < 0.001); D-glutamine and D-glutamate metabolism (*p*-value  <  0.001, FDR < 0.001); glutathione metabolism (*p*-value  <  0.001, FDR < 0.001); and cysteine and methionine metabolism (*p*-value  <  0.001, FDR < 0.001), (Figure 6 and Supplementary Table S6). The boxplots in Figure 6A,B show a general trend for the decrease of the relative abundance of metabolites linked to these pathways when cells were stimulated with LPS (CTRL_LPS). Statistically significant differences were observed in the glutathione metabolic pathway, with the concentration of glycine and glutamate decreased upon treatment with LPS. However, no significant differences were found in the concentration of glutathione. The histidine and aspartate metabolic pathways were also significantly modulated, with LPS causing a significant increase in the levels of L-histidine and N-methyl-L-histidine, while decreasing L-glutamate and histamine.

**Figure 7:**
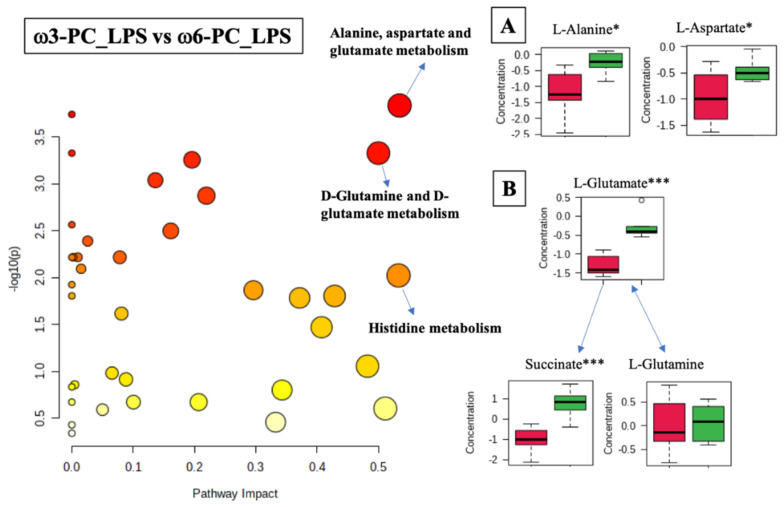
Pathway analysis associated with ω3-PC or ω6-PC with LPS supplementation and stimulation of the RAW 264.7 cell line (log(*p*) values from pathway enrichment analysis vs. values of the pathway impact). (**A**) Summary of relevant nodes in the pathway associated with the metabolism of alanine, aspartate, and glutamate. (**B**) Summary of relevant nodes in the pathway associated with the metabolism of D-glutamine and D-glutamate. Red boxplots ω3-PC_LPS, green boxplots ω6-PC_LPS. * *p* < 0.05 and *** *p* < 0.001.

The differential analysis of the metabolites of cells treated with ω3-PC_LPS vs. ω6-PC_LPS was associated with 22 metabolic pathways (FDR < 0.05) including the alanine, aspartate, and glutamate metabolism (*p*-value  =  0.00015, FDR < 0.01); D-glutamine and D-glutamate metabolism (*p*-value  =  0.0005, FDR < 0.01); and histidine metabolism (*p*-value  =  0.0095, FDR < 0.05), (Figure 7 and Supplementary Table S7). The boxplots of Figure 7A–C show statistically significant differences observed in the alanine and aspartate metabolism, where L-alanine and L-aspartate were significantly increased under ω6-PC_LPS conditions compared to ω3-PC_LPS conditions. In addition, in the metabolism of D-glutamine and D-glutamate, L-glutamate and succinate were significantly increased under ω6-PC _LPS conditions compared to ω3-PC_LPS conditions.

## 3. Discussion

Intense research has been carried out on the biological activity of PUFAs, in particular on their contribution to the modulation of inflammatory responses and their impact on immune cells. The classic dichotomous classification of ω3-PC as anti-inflammatory and ω6-PC as pro-inflammatory has been challenged by numerous studies, indicating that the impact of these bioactive lipids on immune cells is much more complex. In the present work, we sought, through a multi-omic approach, to explore the effects of two phospholipids containing ω3 and ω6 PUFAs (PC 18:0/22:6 or PC 18:0/20:4) on the macrophage’s proteome, lipidome, and metabolome under resting and LPS-activated conditions.

In this study, the Griess reaction was used to estimate the total NO concentration in the culture medium and to assess the activation/inflammatory state of macrophages triggered by LPS and the modulation caused by ω3 and ω6 PLs supplementation. Although several more sensitive techniques can be used to measure nitrite, including fluorometric, electrochemical, and 3-nitrotyrosine quantification assays [32], the classical Griess spectrophotometric assay is most frequently used. In this experimental in vitro model of inflammation, NO levels reached 0.6–34 μM in the culture medium and these values are within the detection range of the Griess reaction [33]. Our results demonstrate that ω3-PC and ω6-PC inhibit the lipopolysaccharide-stimulated production of nitric oxide. This decrease in NO production caused by ω3-PC and ω6-PC is not expected to be the main mechanistic cause of the observed effects of PCs on the macrophage lipidome, metabolome, and proteome. Other studies, for example using NOS inhibitors, will clarify the role of NO in this process.

Our results demonstrated that the supplementation of macrophages with ω3-PC or ω6-PC has a distinct impact on the reprogramming of the proteome, lipidome, and metabolome. Regarding the number of molecular species found to be modulated, ω6-PC supplementation caused the most pronounced effects on the three omic profiles analysed. However, if we focus on the analysis of the 50 most significantly modulated proteins, ω3-PC is clearly responsible for a pronounced impact, causing increased expression in almost all of the datasets. Among the proteins strongly upregulated by ω3-PC, were tyrosine-protein kinase CSK (P41241), neutrophil cytosol factor 1 (Q09014), catalase (P24270), and several enzymes involved in lipid metabolism. Tyrosine-protein kinase CSK is a protein that downregulates the pro-inflammatory cytokine interleukin-6 and TNF-α, as well as the TLR-4-mediated activation of the ERK and p38 signalling pathways [34]. Neutrophil cytosol factor 1 (Ncf1), also known as p47(phox), is a subunit of the NADPH oxidase complex, being involved in the ROS biosynthetic process and in the regulation of the respiratory burst during inflammatory responses [35,36]. In turn, catalase is a detoxifying enzyme that neutralises ROS, mainly hydrogen peroxide (H_2_O_2_), thus assuming an important role in cellular protection against oxidative damage [37]. Catalase has other anti-inflammatory roles, as it is also involved in the downregulation of NF-κB activity during a respiratory burst in macrophages, as well as in the upregulation of the PI3K signalling pathway [38,39]. The PI3K pathway has been reported to negatively modulate the MAPK signalling cascades, namely ERK1/2, p38, JNK, and the NF-κB pathway, and its activation has been demonstrated to be required for macrophage polarisation through an M2 phenotype [40,41,42].

Among the proteins related to lipid metabolism induced by ω3-PC, acetyl-CoA acetyltransferase (Q8QZT1), 3-ketoacyl-CoA thiolase (Q8BWT1), and the trifunctional enzyme subunit alpha (Q8BMS1) may contribute to the ω3 PUFAs anti-inflammatory properties. These three proteins are involved in fatty acid β-oxidation (FAO), a process by which fatty acids are broken down into acetyl-CoA, which is also used in other metabolic pathways, such as the tricarboxylic acid (TCA) cycle or the oxidative phosphorylation (OXPHOS), to produce energy [43]. Recent studies have suggested that the polarisation of macrophages into M2-type (anti-inflammatory) is linked to an increase in the FAO pathway [44]. However, the role of FAO in the polarisation of macrophages is not entirely clear, as FAO has also been implicated in the activation of the NLRP3 inflammasome in M1-like macrophages, increasing the secretion of the pro-inflammatory cytokine IL-1β [45].

Regarding the impact of ω6-PC on the proteome of macrophages, the most significant changes found included the downregulation of proteins, such as aspartate aminotransferase (P05202), fumarate hydratase (P97807), and isocitrate dehydrogenase (P70404). Aspartate aminotransferase is a protein involved in the malate-aspartate shuttle that is also responsible for glutamate metabolism and converts L-aspartate into L-glutamate [46]. Isocitrate dehydrogenase is a key enzyme involved in the conversion of isocitrate to α-ketoglutarate in the TCA cycle. Its decreased activity or expression is expected to result in decreased formation of α-ketoglutarate, a key metabolite for the alternative (M2) activation of macrophages [47]. Finally, fumarate hydratase catalyses the reversible hydration/dehydration of fumarate to malate, which are intermediates of the TCA cycle [48]. It has been reported that the inhibition or decreased activity of fumarate hydratase leads to accumulation of fumarate and increased succinate levels [49,50]. High levels of fumarate inhibit the hypoxia-inducible factors (HIF) prolyl hydroxylases, leading to HIF-1α stabilisation, which results in increased transcription of inflammatory and immune-related genes [51]. It has been demonstrated that the stabilisation of HIF-1α is directly involved in the upregulation of the macrophage M1 markers [52,53]. High levels of fumarate also result in the succination of cysteine-containing proteins, such as the kelch-like EC-associated protein 1 (KEAP1), inactivating it and impairing the expression of NRF2-ARE-dependent genes, such as HMOX1 and NQO1, which are involved in the cytoprotective and antioxidant response [54]. In turn, the accumulation of succinate has been demonstrated to play an important role in inflammation by enhancing IL-1β production through a HIF-1α stabilisation-dependent mechanism [55]. However, recent reports demonstrate that the immunomodulatory functions of succinate are more complex, as it can suppress the secretion of the inflammatory mediators IL-6, tumours TNF-α and NO, as well as inhibit IL-1b mRNA transcription in inflammatory macrophages [56]. In addition, the silencing of SUCNR1, the receptor for succinate, results in the upregulation of the pro-inflammatory genes IL-1β, IL-6, and IL-12b [57].

In contrast to the effects on the proteome, our results indicate that supplementation with ω3-PC and ω6-PC had a modest impact on the lipidome and metabolome of resting macrophages. However, in M1-like LPS-activated macrophages, profound alterations were observed in the three omic profiles of ω3-PC and ω6-PC co-treated cells. At the protein level, the most striking effects were observed in a group of proteins whose LPS-induced expression is further exacerbated by culture with PUFAs, in particular by ω3-PC. Among the highly upregulated proteins, we found heme oxygenase-1 (P14901) NADPH-cytochrome P450 reductase (P37040), immunity-related GTPase family M protein 1 (Irgm1) (Q60766), sequestosome-1 (Q64337), and pleckstrin (Q9JHK5). This potentiation by PC-PUFAs of the expression of these proteins in M1 macrophages could contribute to ω3-PC and ω6-PC anti-inflammatory potential via multiple mechanisms. Heme oxygenase-1 catalyses the NADPH-cytochrome P450 reductase-dependent metabolisation of the pro-oxidant heme to the antioxidant biliverdin and CO. These proteins, therefore, have important antioxidant and anti-inflammatory actions by inhibiting the secretion of the PAMP-induced pro-inflammatory cytokine while increasing the production of IL-10 in macrophages [58].

Irgm1 is involved in the innate immune response and is canonically induced by interferons-beta and gamma [59]. Previous studies have demonstrated that Irgm1 negatively regulate the production of pro-inflammatory cytokines mediated by TLR-4 in LPS-stimulated macrophages [60]. Mice lacking this protein demonstrate uncontrolled production of cytokines, contributing to excessive inflammation. Sequestosome-1 (SQSTM1), also known as the ubiquitin-binding protein p62, is central in autophagy processes and is also involved in the downregulation of NF-κB signalling, impairing the expression of TNF-α, IL-1β, IL-6, and IFN-β [61]. Regarding pleckstrin, it is an abundant protein in platelets and leukocytes, where it affects the second messenger-based signalling events mediated by phospholipase C, PI3Kγ, and inositol 5-phosphatases [62,63].

This PUFAs-induced expression of pleckstrin and its effects on the metabolic process of phosphatidylinositol resulted in the increased levels of PIs observed in our lipidomic analysis. PIs are precursors of signalling molecules, such as phosphatidylinositol phosphates (PIPs), which play a central role in the activation of the PI3K-Akt pathway. As the PI3K-Akt pathway critically restricts proinflammatory responses in TLR-stimulated macrophages, it is plausible to hypothesise that the increased formation of PI mediated by ω3-PC and ω6-PC is one mechanism by which these active lipids exert anti-inflammatory effects.

The role of phospholipids in inflammation is increasingly recognised, with both anti-inflammatory and pro-inflammatory effects in macrophages and other immune cells being reported. Consistent with previous work, we found that the LPS-treatment of macrophages resulted in a profound lipidome remodelling with an emphasis on the increase of lipids classes canonically associated with inflammation, such as PC, LPC, LPE, Cer, and CL [64,65,66]. In addition to the aforementioned increase in PI production triggered by ω3-PC and ω6-PC supplementation, our data demonstrated that these PUFAs decreased the relative abundance of LPE and CL in LPS-stimulated macrophages, thereby contributing to a less pronounced inflammatory state. LPE arises from the hydrolysis of PE by phospholipase A2 (PLA2), its inflammatory actions being potentially due to the increased intracellular calcium levels via activation of PKC [67,68]. Regarding cardiolipins, under homeostatic conditions they are found exclusively in the inner membrane of mitochondria; however, under PAMP stimulation they translocate to the outer mitochondrial membrane, thus activating the NLRP3 inflammasome, which leads to increased production of inflammatory cytokines IL-1β and IL-18 [69,70]. Despite the referred changes induced by ω3-PC and ω6-PC in the lipidome of LPS-activated macrophages that may contribute to some extent to limiting their inflammatory state, we also observed alterations that may aggravate inflammation. It should be noted that the treatment with ω3-PC and ω6-PC of the LPS-activated macrophages resulted in increased levels of LPCs. LPCs are produced by PLA2-mediated hydrolysis of PC species and in macrophages have been demonstrated to promote M1 polarisation through TLR-mediated signalling and to increase phagocytic activity via AMPK and p38 MAPK-dependent mechanisms [71,72,73].

It has been demonstrated that the treatment of macrophages with LPS promotes a pronounced metabolic switch from oxidative phosphorylation toward glycolysis [74]. Paradoxically, despite the decrease in mitochondrial respiration, LPS treatment causes a strong increase in the TCA cycle intermediates fumarate, malate, and succinate. Succinate is transported from the mitochondria, through the dicarboxylic acid transporter to the cytosol, where it stabilises HIF-1α, boosting LPS-induced IL-1 mRNA transcription [53]. Moreover, succinate oxidation via mitochondrial succinate dehydrogenase has also been reported as a key event in the repropose of macrophage mitochondria from ATP synthesis to ROS production [75]. In these works, glutamine-dependent anaplerosis was found to be the major source of succinate. This is corroborated by our results, where we observed a significant decrease of glutamate in LPS-stimulated macrophages. Glutamine is converted to glutamate, which is then consumed to form α-ketoglutarate and then succinate.

Regarding the impact of ω3-PC and ω6-PC on the metabolome of resting and LPS-activated macrophages, it demonstrated a behaviour similar to that observed for the lipidome profile: PUFAs minimally affected resting cells but strongly impacted the metabolome of LPS-activated macrophages. Compared to the ω3-PC respective conditions, the ω6-PC supplemented LPS-activated cells demonstrated significant increases in L-alanine, L-aspartate, L-glutamate, and succinate. As there was no difference in glutamine levels between cells exposed to ω3-PC and ω6-PC, we hypothesise that the increase in glutamate triggered by ω6-PC may result from the transamination of α-ketoglutarate. We also observed that ω6-PC decreases the expression of isocitrate dehydrogenase, the enzyme involved in the conversion of isocitrate to α-ketoglutarate in the TCA cycle. Therefore, both of these events may lead to a substantial decrease in α-ketoglutarate levels, skewing macrophages towards a more pronounced M1 phenotype [49,50]. Concerning succinate, its accumulation in macrophages supplemented with ω6-PC treated with LPS could result from the observed downregulation of fumarate hydratase protein expression triggered by ω6-PC. It is unclear whether the accumulation of succinate induced by ω6-PC contributes to exacerbating the inflammatory status in M1-activated macrophages or, on the contrary, if it downmodulates their activation due to the pro- and anti-inflammatory properties that have been attributed to this metabolite [55,56,57].

Overall, the data from our multi-omic approach were congruent, demonstrating the complex metabolic network underlying the effects of ω3 and ω6 on the proteome, lipidome, and metabolome of M1-polarised macrophages. Although the results obtained support the established notion that ω3 PUFAS have superior anti-inflammatory activity compared with ω6 PUFAs, they also evidence that neither ω3 nor ω6 have exclusively anti- or pro-inflammatory activities, respectively.

## 4. Materials and Methods

### 4.1. Chemicals and Reagents

Acetonitrile (ACN) was purchased from Merck (Darmstadt, Germany), the phospholipids were purchased from Avanti Lipids Polar (Alabaster, AL, USA), and the remaining chemicals/reagents were purchased from Sigma–Aldrich (St Louis, MO, USA). All chemicals were of analytical grade and milli-Q water was used throughout all experiments.

### 4.2. Cell Culture

The mouse leukemic monocyte–macrophage cell line RAW 264.7 (ATCC TIB-71, American Tissue Culture Collection, Manassas, VA, USA), was maintained in high-glucose Dulbecco’s Modified Eagle Medium (DMEM), supplemented with 10% non-inactivated foetal bovine serum (FBS), streptomycin (100 µg/mL) and penicillin (100 U/mL), 1.5 g/L sodium bicarbonate, and 2 mM of glutamine. The cells were kept in the culture in an incubator at 37 °C and 5% CO_2_ and were sub-cultured every 2–3 days to maintain cell density between 0.5 and 0.8 × 10^6^ cells/mL.

### 4.3. Cell Viability Assay

To determine the impact of the phospholipids on cell viability, the resazurin reduction assay was performed accordingly [76]. Macrophages were seeded at 40,000 cells/well in a 96-well plate and allowed to stabilise in the incubator for a period of 24 h. Afterwards, cells were either kept in fresh culture medium (CTRL) or treated with PC 18:0/22:6 (ω3-PC) or PC 18:0/20:4 (ω6-PC), previously incorporated into liposomes [77], with increasing concentrations of phospholipid (10, 20, 50, 100, and 200 μM). After 21 h of incubation, cells (*n* = 3) were treated with resazurin (50 μM) and incubated for 3 h and after 24 h, resorufin was quantified on a plate reader (Multiskan GO 1510-00111C, ThermoScientific, Waltham, MA, USA). All assays were performed in triplicate.

### 4.4. Quantitative Analysis of Nitrites by Griess Reaction

To assess the anti-inflammatory activity of the phospholipids, nitric oxide production was determined by quantitative analysis of nitrites with Griess reagent, as previously described [78]. Macrophages were seeded at 40,000 cells/well in a 96-well plate and allowed to stabilise for a period of 24 h. Cells were either kept in fresh culture medium (CTRL) or treated with PC 18:0/22:6 (ω3-PC) or PC 18:0/20:4 (ω6-PC), with increasing concentrations of phospholipids (10, 20, 50, 100, and 200 μM). After 1 h of incubation, LPS was added as a positive control for inflammatory activity at a final concentration of 100 ng/mL. Phospholipid incubation was carried out for a period of 24 h. All assays were performed in triplicate.

### 4.5. Incubation with Phospholipids

For phospholipid incubation assays, macrophages were seeded in 6-well culture plates at 1 × 10^6^ cells/mL per well. After 24 h, cells were either maintained in a fresh culture medium (control sample, CTRL) or incubated with 100 μM of omega-3 PC 18:0/22:6 (ω3-PC) or 100 μM of omega-6 PC 18:0/20:4 (ω6-PC), previously incorporated into liposomes [77]. After 1 h of phospholipid incubation, one sample of each condition (CTRL, ω3-PC, and ω6-PC) was further treated with 100 ng/mL of lipopolysaccharide (LPS) as a positive control for inflammatory activity (resulting in CTRL_LPS, ω3-PC_LPS, and ω6-PC_LPS samples, respectively). Incubation with phospholipids was performed for 24 h periods. In the case of proteomics analysis, all experiments with phospholipid incubation were carried out using free serum medium.

### 4.6. Proteomics

The proteins were extracted using RIPA buffer. The pellets were resuspended in RIPA solution supplemented with 1M DTT and anti-proteases cocktail (P8340, Sigma–Aldrich), vortexed, and kept on ice for 15 min. Samples were centrifuged at 14,000× *g* for 15 min (4 °C) and the resulting supernatants were carefully collected. The cell lysates were stored at −80 °C. Protein concentrations were determined using the RC/DC protein assay kit (BioRad, Hercules, CA, USA) according to the manufacturer’s protocol. Denaturing SDS-PAGE and in-gel digestion was performed as previously described [79].

The tryptic peptide samples that resulted from in-gel digestion were reconstituted in 40 μL of 5% ACN with 0.1% FA in liquid chromatography–mass spectrometry (LC-MS)-grade water and loaded onto an EASY-Spray high performance-liquid chromatography (HPLC) column (75 μm × 150 mm, 2 μm, 100 A; Thermo Fisher Scientific, Bremen, Germany) operated at 40 °C. Nanoflow LC-MS/MS was performed on a Q-Exactive hybrid quadrupole Orbitrap mass spectrometer (Thermo Fisher Scientific, Bremen, Germany) coupled to an HPLC system (Ultimate 3000 Dionex; Thermo Fisher Scientific, Bremen, Germany). The buffer system consisted of two mobile phases, buffer A (0.1% FA in HPLC grade H_2_O) and buffer B (80% HPLC grade ACN in 20% HPLC grade H_2_O and 0.1% FA) at a flow rate of 300 nL/min. Peptides were eluted with a linear gradient of 5–24% B over 50 min, increased to 36% B over 10 min and held for 5 min. The mass spectrometer was operated in the positive ion mode (electrospray voltage 2.0 kV) with a capillary temperature of 250 °C. MS survey scans were performed with a resolution of 70,000, automatic gain control (AGC) target 1 × 10^6^, and maximum injection time (IT) 100 ms. The 10 most intense peaks per MS scan were selected for higher-energy collisional dissociation (HCD) MS/MS experiments with a resolution of 17,500, AGC target of 5 × 10^4^, maximum IT of 50 ms, a normalised collision energy™ (CE) of 28, isolation width 1.2 Th, and dynamic exclusion of 30 s. Data acquisition was performed using the Xcalibur data system v3.3 (Thermo Fisher Scientific, Bremen, Germany).

Proteins were identified using the Proteome Discoverer software (v2.2.0.388, Thermo Fisher Scientific, Bremen, Germany). The MS data were searched against the Mus musculus (mouse) protein database (Accessed on 9 June 2021, Taxon identifier = 10,090, Swiss Prot), with SEQUEST HT and MS AMANDA 2.0 search engines, and with percolator validation. The search parameters were as follows: carbamidomethylation of cysteine (C) was defined as a static modification and oxidation of methionine (M) and acetylation of N-terminal protein (N-Terminus) were set as dynamic modifications. The mass tolerance of precursor and fragment ions was 10 ppm (MS1) and 0.02 Da (MS2). Up to two missed cleavages were considered for trypsin digestion. The threshold of the global false discovery rate (FDR) for peptides and proteins was set at 0.01. Only proteins with a minimum number of two unique peptides and with a peptide length no lower than 6 amino acids were considered. Proteins with low variability were removed from the analysis as artefacts. Gene ontology (GO) analysis was performed on QuickGO (https://www.ebi.ac.uk/QuickGO/ (accessed on 9 June 2021)) (EMBL-EBI, Cambridgeshire, UK), assigning the protein’s molecular functions, according to the Gene Ontology database.

### 4.7. Lipidomics

Total lipids were extracted from cells and harvested as described for the proteomics analysis using the Bligh and Dyer extraction method, and total phospholipids concentration was determined using the phosphorous assay, as described previously [80,81].

Samples (10 µL of each sample (equivalent to 10 μg of phospholipid), 82 µL of eluent (95% of mobile phase B and 5% of mobile phase A), and 8 µL) were mixed with 8 µL of a solution of PL standards (dimyristoyl-phosphatidylcholine (dMPC)-0.02 µg, dimyristoyl-phosphatidylethanolamine (dMPE)-0.02 µg, Sphingomyelin (SM) (d18:1/17:0)-0.02 µg, Lysophosphatidylcholine (LPC) (19:0)-0.02 µg, dipalmitoyl-phosphoinositol (dPPI)-0.02 µg, cardiolipin (CL) (14:0)4–0.02 µg, dimyristoyl-phosphatidylglycerol (dMPG)-0.012 µg, dimyristoyl-Phosphatidic acid (dMPA)-0.02 µg, ceramide (Cer) (d18:1/17:0)-0.02 µg, dimyristoyl-phosphatidylserine (dMPS)-0.02 µg (Avanti Polar Lipids, Inc., Alabaster, AL, USA)). This mixture (5 µL) was injected into the Ascentis^®^ Express microbore column (10 cm × 2.1 mm, 2.7 µm; Sigma–Aldrich, Saint Louis, MO, USA) at 35 °C and a flow-rate of 200 µL min−1 and analysed by hydrophilic interaction liquid chromatography-mass spectrometry (HILIC-LC-MS) on an Ultimate 3000 Dionex ultra-high-performance liquid chromatography (UHPLC) system (Thermo Fisher Scientific, Bremen, Germany) coupled online to an orbitrap Q-Exactive mass spectrometer (Thermo Fisher Scientific, Bremen, Germany), as previously described [82].

Lipid species were identified using mass spectrometry-data independent analysis (MS-DIAL) v4.70 software [83]. Identification of the lipid species was performed in the negative and positive ionisation modes, using the raw files acquired in MS/MS mode, and converted by the ABF converter (https://www.reifycs.com/AbfConverter/ (accessed on 5 July 2021)) against the lipid database provided by the MS-DIAL software. The tolerances for MS and MS/MS search were set at 0.01 Da and 0.05 Da, and all identifications were manually verified. The validated species were integrated and quantified in the MZmine v2.53 software [84]. Raw LC-MS files were subjected to smoothing and filtering methods, peak detection (including chromatogram construction, peak deconvolution and deisotoping) and peak alignment with gap filling. Later, the integrated peak areas of the extracted ion chromatograms (XIC) were exported, and normalisation was obtained by calculating the ratio against a selected internal lipid standard with the closest retention time.

### 4.8. Metabolomics

The dried samples taken from the aqueous phase of the Bligh and Dyer extraction method were resuspended in an ice-cold solution of MeOH: ACN: Mili-Q water (4:4:2) solution [85]. The samples were then kept at −80 °C for 20 min and centrifuged at 14,000× *g* for 10 min at 4–8 °C (B. Braun Biotech International GmbH, Berlin, Germany). The supernatants were collected and dried under a vacuum. Later, dried samples were resuspended in 80% MS grade MeOH containing an internal standard (Leu-Tyr, 0.02 mg/mL, Sigma–Aldrich, Saint Louis, MO, USA). Samples were normalised by the volume corresponding to the PL concentration measured from the phosphorous assay (0.1 μg/μL).

Metabolites were analysed by HILIC-LC-MS, under the same instrumental setup as described previously for lipidomics analysis, except for the HPLC programme and MS/MS acquisition conditions. The solvent system consisted of two mobile phases, a mobile phase A (ACN: MeOH: H2O, 2.5:2.5:95 (per volume) with 5 mM ammonium acetate and 0.1% of FA) and mobile phase B (ACN:MeOH, 90:10 (per volume) with 5 mM ammonium formate and 0.1% of FA). Metabolites were eluted with a 100% of mobile phase B for 1 min, followed by a linear gradient of 0–50% A over 15 min and held for 5 min. The mass spectrometer operated in the positive (3.1 kV) and negative (−2.8 kV) ion modes with a capillary temperature of 350 °C and a sheath gas flow of 20 U. MS survey scans were performed with a resolution of 70,000, AGC target 1 × 10^6^, and maximum IT 100 ms. Scan range between 65 to 900 m/z. The 10 most intense peaks per MS scan were selected for HCD MS/MS experiments with a resolution of 17,500, AGC target of 1 × 10^3^, and maximum IT of 50 ms; a normalised collision energy™ of 20, 30, and 40; isolation width 1.5 Th; and dynamic exclusion of 30 s. Quality control (QC) samples were included and prepared by pooling aliquots of all samples. Data acquisition was performed using the Xcalibur data system (v3.3, Thermo Fisher Scientific, Waltham, MA, USA).

For the processing of LC-MS data, the workflow was similar to the lipidomics data analysis. Metabolites were identified using MS-DIAL v4.70 software [83], against databases. Identification of metabolites was performed in negative and positive ionisation modes, using the QC raw files acquired in MS/MS analysis for each mode. The validated species were integrated and quantified, using MZmine v2.53 software [84]. Normalisation of the integrated peak areas of the XIC was achieved by calculating the ratio against the internal standard.

### 4.9. Statistical Analysis

Multivariate and univariate analyses were performed using R v3.6.2 [86] in Rstudio v1.2.5 [87]. The data from proteomics and lipidomics analysis were log-transformed, and the EigenMS was normalised [88] and subjected to auto-scaling. Metabolomics data were normalised by sum before being log-scaled, and the EigenMS was normalised [88] and subjected to auto-scaling. The missing values were replaced by 1/5 of the minimum positive values of their corresponding variables. The principal component analysis (PCA) was performed for all the omics, with the R built-in function and the R package pcaMethods [89]. In univariate analysis, the Kruskal–Wallis test followed by Dunn ‘s post hoc test was used for nonparametric comparisons and pairwise multiple-comparison between the 6 conditions, respectively. PCA graphs and boxplots were created using the R package ggplot2 [90], and heatmaps were created using the R package pheatmap, using “Euclidean” as the clustering distance, and “ward.D” as the clustering method [91]. To explore the metabolomic profiles, we also applied the pathway analysis module from Metaboanalyst (https://www.metaboanalyst.ca (accessed on 12 July 2021)) [92], using the following pathway analysis parameters: enrichment method—Global Test, topology analysis—relative-betweenness centrality; pathway library—Mus musculus (KEGG).

## Figures and Tables

**Table 1:** Number of proteins with significantly different relative abundance (q < 0.05) between conditions (FDR adjusted Dunn’s multiple comparison test).

	CTRL	ω3-PC	ω6-PC	CTRL_LPS	ω3-PC_LPS
CTRL	-				
ω3-PC	215	-			
ω6-PC	726	887	-		
CTRL_LPS	1346	2173	993	-	
ω3-PC_LPS	1498	1854	1182	480	-
ω6-PC_LPS	2008	2541	1256	225	526

**Table 2:** Number of lipids with fold change (FC) > 1.5 between conditions and q < 0.05. Control (CTRL), control with LPS (CTRL_LPS), omega-3 PC (ω3-PC), omega-3 PC with LPS (ω3-PC_LPS), omega-6 PC (ω6-PC), and omega-6 PC with LPS (ω6-PC_LPS) (*n* = 6).

	CTRL	ω3-PC	ω6-PC	CTRL_LPS	ω3-PC_LPS
ω3	3 (1CL; 1LPC; 1PC)	-			
ω6	14 (2CAR; 5DG; 3LPE; 2PE; 2 PI)	0	-		
CTRL-LPS	44 (2CER;8CL; 10DG;1 HEXCER; 9LPE; 1PE; 5PG; 8PI)	25 (1CAR;1CER;5CL; 7DG; 4LPE; 3PC; 3PG; 1PI)	19 (3CAR;2CER;3CL; 1DG;5PC;1PE; 3PG; 1PI)	-	
ω3-PC_LPS	53 (5CER;5CL; 7DG;7LPC;9LPE; 2PC; 3PG; 12PI; 2SM)	34 (2CAR; 2CER; 2CL; 2DG;7LPC;4LPE; 1PC; 2PG; 11PI)	16 (2CAR;3CER;2CL; 2LPC;1PE; 2PG; 4PI)	5 (1DG;3LPC;1PC)	-
ω6-PC_LPS	66 (1CAR;4CER;5CL; 8DG;11LPC; 14LPE; 4PC; 1PE; 4PG; 12PI; 1SM)	49 (2CER;4CL; 6DG;10LPC; 9LPE; 2PC; 1PE; 2PG; 12PI; 1SM)	31 (1CAR; 2CER;3CL;5LPC;3LPE; 3PC; 4PG; 9PI; 1SM)	23 (1CAR; 1CL; 3DG; 8LPC;2LP; 6PC;2SM)	3 (2LPE; 1PC)

## Data Availability

The data that support the findings of this study are available from the corresponding author upon reasonable request.

## Supplementary Materials

The following supporting information can be downloaded at: https://www.mdpi.com/article/10.3390/ijms23042139/s1.
