# Supplementary material for: Multi-Omic Profiling of Macrophages Treated with Phospholipids Containing Omega-3 and Omega-6 Fatty Acids Reveals Complex Immunomodulatory Adaptations at Protein, Lipid and Metabolic Levels
Source: Int J Mol Sci. 2022 Feb 15;23(4):2139. doi: 10.3390/ijms23042139 (PMC8879791; doi:10.3390/ijms23042139)
Supplement: Supplementary file 1 [file ijms-23-02139-s001.zip › ijms-1580716 supplementary.pdf]

International Journal of *Molecular Sciences*

MDPI

Metabolic Adaptation of Macrophages to supplementation with phospholipids with Omega-3 and Omega-6 fatty acids

Tatiana Maurício [1], Susana Aveiro[2], Sofia Guedes[1], Diana Lopes[1,3], Tânia Melo[1,3], Bruno Neves[4], Rosário Domingues[1,3] and Pedro Domingues[1*]

Supplementary Table S1. Proteins identified and quantified by proteomics in RAW 264.7 macrophages stimulated with phospholipid ω3-PC or ω6-PC alone and with LPS.

ID	Protein name	Coverage [%]	No. of identified peptides	Nº. of identified unique peptides
A1L314	Macrophage-expressed gene 1 protein [OS=Mus musculus]	12	5	5
A2A432-1	Cullin-4B [OS=Mus musculus]	23	18	12
A2AAY5	SH3 and PX domain-containing protein 2B [OS=Mus musculus]	8	5	5
A2ADY9	Protein DDI1 homolog 2 [OS=Mus musculus]	14	3	3
A2AGT5	Cytoskeleton-associated protein 5 [OS=Mus musculus]	22	29	29
A2AIV2-1	protein virilizer homolog [OS=Mus musculus]	3	3	3
A2AIV8	caspase recruitment domain-containing protein 9 [OS=Mus musculus]	11	4	4
A2AJ15	endoplasmic reticulum mannosyl-oligosaccharide 1,2-alpha-mannosidase [OS=Mus musculus]	5	2	2
A2AJI0	MAP7 domain-containing protein 1 [OS=Mus musculus]	5	4	4
A2APY7-1	Arginine-hydroxylase NDUFAF5, mitochondrial [OS=Mus musculus]	14	3	3
A2BE28	Ribosomal biogenesis protein LAS1L [OS=Mus musculus]	15	9	9
A2BH40	AT-rich interactive domain-containing protein 1A [OS=Mus musculus]	7	10	10
A6H5X4-1	PHD finger protein 11 [OS=Mus musculus]	23	5	5
A6H611	Mitochondrial intermediate peptidase [OS=Mus musculus]	19	9	9
A6H8H2-2	Isoform 2 of DENN domain-containing protein 4C [OS=Mus musculus]	1	2	2
A8C756-1	Thyroid adenoma-associated protein homolog [OS=Mus musculus]	1	2	2
B1AVY7	Kinesin-like protein KIF16B [OS=Mus musculus]	3	2	2
B1AZI6	THO complex subunit 2 [OS=Mus musculus]	2	2	2
B2RRE7	OTU domain-containing protein 4 [OS=Mus musculus]	6	6	6
B2RXS4	Plexin-B2 [OS=Mus musculus]	33	38	38
B2RY56	RNA-binding protein 25 [OS=Mus musculus]	20	10	10
B7ZMP1-1	Probable Xaa-Pro aminopeptidase 3 [OS=Mus musculus]	17	4	4

B9EJ86	Oxysterol-binding protein-related protein 8 [OS=Mus musculus]	31	18	18
B9EJR8	Dynein assembly factor 5, axonemal [OS=Mus musculus]	9	4	4
D0QMC3	myeloid cell nuclear differentiation antigen-like protein [OS=Mus musculus]	8	3	2
D3Z6Q9	Bridging integrator 2 [OS=Mus musculus]	24	7	7
D3Z7P3-1	Glutaminase kidney isoform, mitochondrial [OS=Mus musculus]	56	28	5
D3Z7P3-2	Isoform 2 of Glutaminase kidney isoform, mitochondrial [OS=Mus musculus]	57	26	3
E9PVA8	eIF-2-alpha kinase activator GCN1 [OS=Mus musculus]	29	48	48
E9PVX6	Proliferation marker protein Ki-67 [OS=Mus musculus]	24	39	39
E9Q1P8	Interferon regulatory factor 2-binding protein 2 [OS=Mus musculus]	27	8	5
E9Q3L2	phosphatidylinositol 4-kinase alpha [OS=Mus musculus]	4	6	6
E9Q557	Desmoplakin [OS=Mus musculus]	1	2	2
E9Q5C9	nucleolar and coiled-body phosphoprotein 1 [OS=Mus musculus]	5	3	3
E9Q5G3	Kinesin-like protein KIF23 [OS=Mus musculus]	7	4	4
E9Q634	unconventional myosin-Ie [OS=Mus musculus]	29	21	15
E9Q735	ubiquitin conjugation factor e4 a [OS=Mus musculus]	3	3	3
E9Q7G0	nuclear mitotic apparatus protein 1 [OS=Mus musculus]	33	46	46
E9Q9A9-2	Isoform 2 of 2'-5'-oligoadenylate synthase 2 [OS=Mus musculus]	10	5	5
F6ZDS4	Nucleoprotein TPR [OS=Mus musculus]	26	45	45
G5E829	Plasma membrane calcium-transporting ATPase 1 [OS=Mus musculus]	9	7	7
G5E870	E3 ubiquitin-protein ligase TRIP12 [OS=Mus musculus]	16	20	20
O08528	Hexokinase-2 [OS=Mus musculus]	42	30	25
O08529	Calpain-2 catalytic subunit [OS=Mus musculus]	37	15	15
O08539-1	Myc box-dependent-interacting protein 1 [OS=Mus musculus]	23	7	7
O08547	Vesicle-trafficking protein SEC22b [OS=Mus musculus]	62	13	13
O08553	Dihydropyrimidinase-related protein 2 [OS=Mus musculus]	63	24	24
O08579	Emerin [OS=Mus musculus]	50	8	8

O08582	GTP-binding protein 1 [OS=Mus musculus]	20	8	8
O08583-1	THO complex subunit 4 [OS=Mus musculus]	47	9	9
O08585	clathrin light chain A [OS=Mus musculus]	5	2	2
O08599-2	Isoform 2 of Syntaxin-binding protein 1 [OS=Mus musculus]	37	13	13
O08604	Retinoic acid early-inducible protein 1-gamma [OS=Mus musculus]	14	3	2
O08663	methionine aminopeptidase 2 [OS=Mus musculus]	46	12	12
O08691	arginase-2, mitochondrial [OS=Mus musculus]	37	7	7
O08709	Peroxiredoxin-6 [OS=Mus musculus]	81	17	17
O08734	bcl-2 homologous antagonist/killer [OS=Mus musculus]	53	7	7
O08749	Dihydrolipoyl dehydrogenase, mitochondrial [OS=Mus musculus]	45	15	15
O08756	3-hydroxyacyl-CoA dehydrogenase type-2 [OS=Mus musculus]	54	8	8
O08759	Ubiquitin-protein ligase E3A [OS=Mus musculus]	23	14	14
O08784	Treacle protein [OS=Mus musculus]	14	12	12
O08788-1	Dynactin subunit 1 [OS=Mus musculus]	34	29	29
O08795-2	Isoform 2 of Glucosidase 2 subunit beta [OS=Mus musculus]	29	11	11
O08800	Serpin B8 [OS=Mus musculus]	26	6	6
O08807	Peroxiredoxin-4 [OS=Mus musculus]	61	13	11
O08808	Protein diaphanous homolog 1 [OS=Mus musculus]	20	17	17
O08810	116 kDa U5 small nuclear ribonucleoprotein component [OS=Mus musculus]	21	14	14
O08915	AH receptor-interacting protein [OS=Mus musculus]	27	6	6
O08992	Syntenin-1 [OS=Mus musculus]	37	6	6
O08997	Copper transport protein ATOX1 [OS=Mus musculus]	47	2	2
O09043	Napsin-A [OS=Mus musculus]	22	3	3
O09044	Synaptosomal-associated protein 23 [OS=Mus musculus]	45	6	6
O09061	proteasome subunit beta type-1 [OS=Mus musculus]	58	13	13
O09106	histone deacetylase 1 [OS=Mus musculus]	42	12	9
O09111	NADH dehydrogenase [ubiquinone] 1 beta subcomplex subunit 11, mitochondrial [OS=Mus musculus]	27	2	2

O09117-2	Isoform 2 of Synaptophysin-like protein 1 [OS=Mus musculus]	15	2	2
O09131	Glutathione S-transferase omega-1 [OS=Mus musculus]	35	5	5
O09159	Lysosomal alpha-mannosidase [OS=Mus musculus]	21	1<br>3	13
O09167	60S ribosomal protein L21 [OS=Mus musculus]	38	5	5
O09172	Glutamate--cysteine ligase regulatory subunit [OS=Mus musculus]	32	6	6
O35114	Lysosome membrane protein 2 [OS=Mus musculus]	15	5	5
O35129	Prohibitin-2 [OS=Mus musculus]	60	1<br>5	15
O35134	DNA-directed RNA polymerase I subunit RPA1 [OS=Mus musculus]	6	6	6
O35144-1	Telomeric repeat-binding factor 2 [OS=Mus musculus]	6	3	3
O35166	Golgi SNAP receptor complex member 2 [OS=Mus musculus]	31	4	4
O35215	D-dopachrome decarboxylase [OS=Mus musculus]	79	7	7
O35218	Cleavage and polyadenylation specificity factor subunit 2 [OS=Mus musculus]	24	1<br>2	12
O35226	26S proteasome non-ATPase regulatory subunit 4 [OS=Mus musculus]	38	1<br>0	2
O35226-2	Isoform Rpn10B of 26S proteasome non-ATPase regulatory subunit 4 [OS=Mus musculus]	38	1<br>1	3
O35286	Pre-mRNA-splicing factor ATP-dependent RNA helicase DHX15 [OS=Mus musculus]	22	1<br>3	13
O35295	Transcriptional activator protein Pur-beta [OS=Mus musculus]	38	6	6
O35309	N-myc-interactor [OS=Mus musculus]	18	3	3
O35326	Serine/arginine-rich splicing factor 5 [OS=Mus musculus]	18	4	3
O35343	Importin subunit alpha-3 [OS=Mus musculus]	47	1<br>3	9
O35344	Importin subunit alpha-4 [OS=Mus musculus]	56	1<br>5	11
O35345	Importin subunit alpha-7 [OS=Mus musculus]	43	1<br>5	10
O35375-1	Neuropilin-2 [OS=Mus musculus]	5	3	3
O35381	Acidic leucine-rich nuclear phosphoprotein 32 family member A [OS=Mus musculus]	38	9	8
O35382	Exocyst complex component 4 [OS=Mus musculus]	9	5	5
O35387	HCLS1-associated protein X-1 [OS=Mus musculus]	29	5	5
O35405	Phospholipase D3 [OS=Mus musculus]	14	4	4
O35435	Dihydroorotate dehydrogenase (Quinone), mitochondrial [OS=Mus musculus]	36	8	8
O35448	Lysosomal thioesterase PPT2 [OS=Mus musculus]	11	2	2
O35451	Cyclic AMP-dependent transcription factor ATF-6 beta [OS=Mus musculus]	5	2	2

O35459	Delta(3,5)-Delta(2,4)-dienoyl-CoA isomerase, mitochondrial [OS=Mus musculus]	35	9	9
O35465-2	Isoform 2 of Peptidyl-prolyl cis-trans isomerase FKBP8 [OS=Mus musculus]	32	8	8
O35516	Neurogenic locus notch homolog protein 2 [OS=Mus musculus]	5	8	8
O35551-6	Isoform 6 of Rab GTPase-binding effector protein 1 [OS=Mus musculus]	3	2	2
O35593	26S proteasome non-ATPase regulatory subunit 14 [OS=Mus musculus]	53	9	9
O35598	Disintegrin and metalloproteinase domain-containing protein 10 [OS=Mus musculus]	6	2	2
O35601	FYN-binding protein 1 [OS=Mus musculus]	27	12	12
O35609	Secretory carrier-associated membrane protein 3 [OS=Mus musculus]	19	4	4
O35613	Death domain-associated protein 6 [OS=Mus musculus]	12	6	6
O35623	BET1 homolog [OS=Mus musculus]	25	2	2
O35638	Cohesin subunit SA-2 [OS=Mus musculus]	13	11	11
O35639	annexin A3 [OS=Mus musculus]	68	20	19
O35643	AP-1 complex subunit beta-1 [OS=Mus musculus]	45	34	16
O35654	DNA polymerase delta subunit 2 [OS=Mus musculus]	18	5	5
O35657	sialidase-1 [OS=Mus musculus]	11	2	2
O35658	Complement component 1 Q subcomponent-binding protein, mitochondrial [OS=Mus musculus]	55	9	9
O35685	nuclear migration protein nudC [OS=Mus musculus]	41	13	13
O35691	Pinin [OS=Mus musculus]	4	2	2
O35704	serine palmitoyltransferase 1 [OS=Mus musculus]	21	6	6
O35737	Heterogeneous nuclear ribonucleoprotein H [OS=Mus musculus]	51	15	8
O35841	Apoptosis inhibitor 5 [OS=Mus musculus]	55	20	20
O35855	Branched-chain-amino-acid aminotransferase, mitochondrial [OS=Mus musculus]	40	14	14
O35857	Mitochondrial import inner membrane translocase subunit TIM44 [OS=Mus musculus]	28	12	12
O35864	COP9 signalosome complex subunit 5 [OS=Mus musculus]	43	9	9

O35887	Calumenin [OS=Mus musculus]	64	14	14
O35900	U6 snRNA-associated Sm-like protein LSm2 [OS=Mus musculus]	48	3	3
O35955	Proteasome subunit beta type-10 [OS=Mus musculus]	19	3	3
O35972	39S ribosomal protein L23, mitochondrial [OS=Mus musculus]	45	4	4
O54692	Centromere/kinetochore protein zw10 homolog [OS=Mus musculus]	34	16	16
O54734	Dolichyl-diphosphooligosaccharide--protein glycosyltransferase 48 kDa subunit [OS=Mus musculus]	52	14	14
O54782	Epididymis-specific alpha-mannosidase [OS=Mus musculus]	9	6	6
O54784	Death-associated protein kinase 3 [OS=Mus musculus]	6	2	2
O54825	Bystin [OS=Mus musculus]	37	12	12
O54833	casein kinase II subunit alpha' [OS=Mus musculus]	44	13	12
O54879	High mobility group protein B3 [OS=Mus musculus]	38	5	5
O54885	TYRO protein tyrosine kinase-binding protein [OS=Mus musculus]	25	2	2
O54941	SWI/SNF-related matrix-associated actin-dependent regulator of chromatin subfamily E member 1 [OS=Mus musculus]	7	2	2
O54946-1	DnaJ homolog subfamily B member 6 [OS=Mus musculus]	13	4	4
O54962	Barrier-to-autointegration factor [OS=Mus musculus]	40	2	2
O54984	ATPase ASNA1 [OS=Mus musculus]	50	12	12
O54988-1	STE20-like serine/threonine-protein kinase [OS=Mus musculus]	26	18	18
O55013	Trafficking protein particle complex subunit 3 [OS=Mus musculus]	20	3	3
O55022	Membrane-associated progesterone receptor component 1 [OS=Mus musculus]	54	9	8
O55023	Inositol monophosphatase 1 [OS=Mus musculus]	30	6	6
O55028	[3-methyl-2-oxobutanoate dehydrogenase [lipoamide]] kinase, mitochondrial [OS=Mus musculus]	25	6	6
O55098	Serine/threonine-protein kinase 10 [OS=Mus musculus]	18	12	12
O55100-1	Synaptogyrin-1 [OS=Mus musculus]	10	2	2
O55101	Synaptogyrin-2 [OS=Mus musculus]	19	2	2
O55106	striatin [OS=Mus musculus]	17	7	7
O55125	Protein NipSnap homolog 1 [OS=Mus musculus]	37	6	6
O55126	Protein NipSnap homolog 2 [OS=Mus musculus]	17	3	3
O55131	Septin-7 [OS=Mus musculus]	50	14	13
O55135	eukaryotic translation initiation factor 6 [OS=Mus musculus]	51	7	7

O55142	60S ribosomal protein L35a [OS=Mus musculus]	15	3	3
O55143-1	Sarcoplasmic/endoplasmic reticulum calcium ATPase 2 [OS=Mus musculus]	27	20	20
O55222	Integrin-linked protein kinase [OS=Mus musculus]	17	5	5
O55234	proteasome subunit beta type-5 [OS=Mus musculus]	42	10	10
O55242-1	sigma non-opioid intracellular receptor 1 [OS=Mus musculus]	34	4	4
O70126	Aurora kinase B [OS=Mus musculus]	21	5	5
O70145	Neutrophil cytosol factor 2 [OS=Mus musculus]	44	15	15
O70152	Dolichol-phosphate mannosyltransferase subunit 1 [OS=Mus musculus]	45	8	8
O70172	phosphatidylinositol 5-phosphate 4-kinase type-2 alpha [OS=Mus musculus]	15	5	4
O70194	Eukaryotic translation initiation factor 3 subunit D [OS=Mus musculus]	52	18	18
O70251	Elongation factor 1-beta [OS=Mus musculus]	54	8	7
O70252	Heme oxygenase 2 [OS=Mus musculus]	67	18	18
O70274	Protein tyrosine phosphatase type IVA 2 [OS=Mus musculus]	36	4	4
O70305-1	Ataxin-2 [OS=Mus musculus]	4	3	3
O70309-2	Isoform Beta-5B of Integrin beta-5 [OS=Mus musculus]	12	7	6
O70310	glycylpeptide N-tetradecanoyltransferase 1 [OS=Mus musculus]	33	12	9
O70311	Glycylpeptide N-tetradecanoyltransferase 2 [OS=Mus musculus]	17	5	2
O70318	band 4.1-like protein 2 [OS=Mus musculus]	33	19	19
O70370	cathepsin S [OS=Mus musculus]	49	12	12
O70378	ER membrane protein complex subunit 8 [OS=Mus musculus]	42	6	6
O70404	vesicle-associated membrane protein 8 [OS=Mus musculus]	33	4	4
O70422	General transcription factor IIH subunit 4 [OS=Mus musculus]	7	2	2
O70435	Proteasome subunit alpha type-3 [OS=Mus musculus]	45	10	10
O70439	Syntaxin-7 [OS=Mus musculus]	55	8	8
O70469	Docking protein 2 [OS=Mus musculus]	19	5	5
O70475	UDP-glucose 6-dehydrogenase [OS=Mus musculus]	23	7	7
O70480	Vesicle-associated membrane protein 4 [OS=Mus musculus]	28	3	3
O70492	sorting nexin-3 [OS=Mus musculus]	59	8	7
O70493	Sorting nexin-12 [OS=Mus musculus]	47	6	5

O70503-1	Very-long-chain 3-oxoacyl-CoA reductase [OS=Mus musculus]	56	14	14
O70551	SRSF protein kinase 1 [OS=Mus musculus]	25	10	10
O70566	Protein diaphanous homolog 2 [OS=Mus musculus]	2	2	2
O70589-1	peripheral plasma membrane protein CASK [OS=Mus musculus]	7	4	4
O70591	Prefoldin subunit 2 [OS=Mus musculus]	27	3	3
O88271	Craniofacial development protein 1 [OS=Mus musculus]	16	3	3
O88286	Protein Wiz [OS=Mus musculus]	2	2	2
O88342	WD repeat-containing protein 1 [OS=Mus musculus]	45	13	13
O88351	Inhibitor of nuclear factor kappa-B kinase subunit beta [OS=Mus musculus]	9	4	4
O88384	vesicle transport through interaction with t-SNAREs homolog 1B [OS=Mus musculus]	24	4	4
O88441	Metaxin-2 [OS=Mus musculus]	60	8	8
O88447	Kinesin light chain 1 [OS=Mus musculus]	27	11	7
O88448	Kinesin light chain 2 [OS=Mus musculus]	17	5	2
O88456	Calpain small subunit 1 [OS=Mus musculus]	48	7	7
O88487	cytoplasmic dynein 1 intermediate chain 2 [OS=Mus musculus]	40	13	13
O88512	AP-1 complex subunit gamma-like 2 [OS=Mus musculus]	5	2	2
O88531	Palmitoyl-protein thioesterase 1 [OS=Mus musculus]	37	8	8
O88532	Zinc finger RNA-binding protein [OS=Mus musculus]	6	4	4
O88543	COP9 signalosome complex subunit 3 [OS=Mus musculus]	23	5	5
O88544	COP9 signalosome complex subunit 4 [OS=Mus musculus]	57	15	15
O88545	COP9 signalosome complex subunit 6 [OS=Mus musculus]	35	8	8
O88569	heterogeneous nuclear ribonucleoproteins A2/B1 [OS=Mus musculus]	59	22	20
O88574	Histone deacetylase complex subunit SAP30 [OS=Mus musculus]	14	2	2
O88587-1	Catechol O-methyltransferase [OS=Mus musculus]	56	10	10
O88643	Serine/threonine-protein kinase PAK 1 [OS=Mus musculus]	20	8	3
O88653	ragulator complex protein LAMTOR3 [OS=Mus musculus]	63	4	4
O88668	Protein CREG1 [OS=Mus musculus]	29	4	4
O88685	26S proteasome regulatory subunit 6A [OS=Mus musculus]	55	18	18
O88696	ATP-dependent Clp protease proteolytic subunit, mitochondrial [OS=Mus musculus]	38	6	6

O88708	origin recognition complex subunit 4 [OS=Mus musculus]	12	3	3
O88712-1	C-terminal-binding protein 1 [OS=Mus musculus]	32	8	7
O88736	3-keto-steroid reductase [OS=Mus musculus]	16	4	4
O88746	Target of Myb protein 1 [OS=Mus musculus]	16	4	4
O88796	ribonuclease P protein subunit p30 [OS=Mus musculus]	10	2	2
O88811-1	Signal transducing adapter molecule 2 [OS=Mus musculus]	13	4	4
O88839-1	Disintegrin and metalloproteinase domain-containing protein 15 [OS=Mus musculus]	6	4	4
O88842-1	FYVE, RhoGEF and PH domain-containing protein 3 [OS=Mus musculus]	27	11	11
O88844	Isocitrate dehydrogenase [NADP] cytoplasmic [OS=Mus musculus]	55	23	22
O88874	Cyclin-K [OS=Mus musculus]	10	3	3
O88895-1	Histone deacetylase 3 [OS=Mus musculus]	6	2	2
O88907	E3 SUMO-protein ligase PIAS1 [OS=Mus musculus]	6	2	2
O88952	Protein lin-7 homolog C [OS=Mus musculus]	34	4	4
O88967	ATP-dependent zinc metalloprotease YME1L1 [OS=Mus musculus]	36	15	15
O88983	Syntaxin-8 [OS=Mus musculus]	12	2	2
O88986	2-amino-3-ketobutyrate coenzyme A ligase, mitochondrial [OS=Mus musculus]	55	11	11
O89001	Carboxypeptidase D [OS=Mus musculus]	15	15	15
O89017	Legumain [OS=Mus musculus]	32	9	9
O89023	Tripeptidyl-peptidase 1 [OS=Mus musculus]	38	9	9
O89051	Integral membrane protein 2B [OS=Mus musculus]	11	2	2
O89053	Coronin-1A [OS=Mus musculus]	52	19	19
O89079	coatomer subunit epsilon [OS=Mus musculus]	73	14	14
O89086	RNA-binding protein 3 [OS=Mus musculus]	42	3	3
O89110	Caspase-8 [OS=Mus musculus]	30	9	9
O89116	Vesicle transport through interaction with t-SNAREs homolog 1A [OS=Mus musculus]	20	2	2
P00375	dihydrofolate reductase [OS=Mus musculus]	41	5	5
P00405	Cytochrome c oxidase subunit 2 [OS=Mus musculus]	41	5	5
P00493	Hypoxanthine-guanine phosphoribosyltransferase [OS=Mus musculus]	71	12	12
P00860	ornithine decarboxylase [OS=Mus musculus]	10	2	2
P00920	Carbonic anhydrase 2 [OS=Mus musculus]	47	8	8
P01582	interleukin-1 alpha [OS=Mus musculus]	42	8	8

P01887	Beta-2-microglobulin [OS=Mus musculus]	60	4	4
P01897	H-2 class I histocompatibility antigen, L-D alpha chain [OS=Mus musculus]	41	13	11
P01900	H-2 class I histocompatibility antigen, D-D alpha chain [OS=Mus musculus]	35	10	3
P01902	h-2 class I histocompatibility antigen, K-D alpha chain [OS=Mus musculus]	43	15	11
P01942	Hemoglobin subunit alpha [OS=Mus musculus]	19	2	2
P02468	Laminin subunit gamma-1 [OS=Mus musculus]	9	8	8
P02535-1	Keratin, type I cytoskeletal 10 [OS=Mus musculus]	18	9	5
P03930	ATP synthase protein 8 [OS=Mus musculus]	34	2	2
P03958	adenosine deaminase [OS=Mus musculus]	13	3	3
P03966	N-myc proto-oncogene protein [OS=Mus musculus]	13	2	2
P03995	Glial fibrillary acidic protein [OS=Mus musculus]	17	6	6
P04104	Keratin, type II cytoskeletal 1 [OS=Mus musculus]	5	4	2
P04184	Thymidine kinase, cytosolic [OS=Mus musculus]	25	5	5
P04202	Transforming growth factor beta-1 [OS=Mus musculus]	19	4	4
P04370-1	myelin basic protein [OS=Mus musculus]	9	2	2
P04441	H-2 class II histocompatibility antigen gamma chain [OS=Mus musculus]	17	5	5
P05063	Fructose-bisphosphate aldolase C [OS=Mus musculus]	58	17	15
P05064	fructose-bisphosphate aldolase A [OS=Mus musculus]	73	21	19
P05132-1	cAMP-dependent protein kinase catalytic subunit alpha [OS=Mus musculus]	36	10	5
P05201	Aspartate aminotransferase, cytoplasmic [OS=Mus musculus]	64	18	18
P05202	Aspartate aminotransferase, mitochondrial [OS=Mus musculus]	66	25	25
P05555-1	Integrin alpha-M [OS=Mus musculus]	41	38	38
P06151	L-lactate dehydrogenase A chain [OS=Mus musculus]	78	26	25
P06339	H-2 class I histocompatibility antigen, D-37 alpha chain [OS=Mus musculus]	14	4	2
P06537-2	Isoform 2 of Glucocorticoid receptor [OS=Mus musculus]	5	2	2
P06745	glucose-6-phosphate isomerase [OS=Mus musculus]	61	29	29
P06797	Cathepsin L1 [OS=Mus musculus]	52	13	13

P06800	Receptor-type tyrosine-protein phosphatase C [OS=Mus musculus]	16	13	13
P06801	NADP-dependent malic enzyme [OS=Mus musculus]	20	6	6
P06804	tumor necrosis factor [OS=Mus musculus]	21	3	3
P07091	Protein S100-A4 [OS=Mus musculus]	39	5	5
P07310	Creatine kinase M-type [OS=Mus musculus]	12	2	2
P07356	Annexin A2 [OS=Mus musculus]	65	23	23
P07607	thymidylate synthase [OS=Mus musculus]	36	7	7
P07724	Serum albumin [OS=Mus musculus]	7	3	3
P07742	Ribonucleoside-diphosphate reductase large subunit [OS=Mus musculus]	51	25	25
P07901	Heat shock protein HSP 90-alpha [OS=Mus musculus]	65	55	37
P08003	Protein disulfide-isomerase A4 [OS=Mus musculus]	59	37	36
P08030	Adenine phosphoribosyltransferase [OS=Mus musculus]	82	11	11
P08101-1	Low affinity immunoglobulin gamma Fc region receptor II [OS=Mus musculus]	21	5	5
P08103-1	Tyrosine-protein kinase HCK [OS=Mus musculus]	36	14	11
P08113	Endoplasmin [OS=Mus musculus]	63	53	51
P08207	Protein S100-A10 [OS=Mus musculus]	49	3	3
P08228	Superoxide dismutase [Cu-Zn] [OS=Mus musculus]	73	8	8
P08249	Malate dehydrogenase, mitochondrial [OS=Mus musculus]	59	18	18
P08551	Neurofilament light polypeptide [OS=Mus musculus]	11	4	4
P08556	GTPase NRas [OS=Mus musculus]	51	7	2
P08730-1	Keratin, type I cytoskeletal 13 [OS=Mus musculus]	13	8	2
P08752	guanine nucleotide-binding protein G(i) subunit alpha-2 [OS=Mus musculus]	63	20	13
P08775	DNA-directed RNA polymerase II subunit RPB1 [OS=Mus musculus]	13	14	14
P08905	lysozyme c-2 [OS=Mus musculus]	50	5	5
P09055	Integrin beta-1 [OS=Mus musculus]	34	17	17
P09103	Protein disulfide-isomerase [OS=Mus musculus]	72	39	39

P09405	Nucleolin [OS=Mus musculus]	46	29	29
P09411	phosphoglycerate kinase 1 [OS=Mus musculus]	73	28	28
P09450	transcription factor jun-B [OS=Mus musculus]	35	6	6
P09528	Ferritin heavy chain [OS=Mus musculus]	63	13	13
P09581	Macrophage colony-stimulating factor 1 receptor [OS=Mus musculus]	32	22	22
P09671	Superoxide dismutase [Mn], mitochondrial [OS=Mus musculus]	77	11	11
P0C0S6	Histone H2A.Z [OS=Mus musculus]	43	4	3
P0C6B7	Immunoglobulin superfamily member 6 [OS=Mus musculus]	19	3	3
P0C7N9	Proteasome assembly chaperone 4 [OS=Mus musculus]	46	3	3
P0DN34	NADH dehydrogenase [ubiquinone] 1 beta subcomplex subunit 1 [OS=Mus musculus]	49	2	2
P10107	annexin A1 [OS=Mus musculus]	61	25	25
P10126	Elongation factor 1-alpha 1 [OS=Mus musculus]	62	21	14
P10404	MLV-related proviral Env polyprotein [OS=Mus musculus]	17	11	7
P10518	Delta-aminolevulinic acid dehydratase [OS=Mus musculus]	49	11	11
P10605	Cathepsin B [OS=Mus musculus]	68	16	16
P10639	thioredoxin [OS=Mus musculus]	47	5	5
P10649	Glutathione S-transferase Mu 1 [OS=Mus musculus]	37	8	6
P10711	Transcription elongation factor A protein 1 [OS=Mus musculus]	39	8	8
P10810	Monocyte differentiation antigen CD14 [OS=Mus musculus]	60	16	16
P10833	Ras-related protein R-Ras [OS=Mus musculus]	33	7	6
P10852	4F2 cell-surface antigen heavy chain [OS=Mus musculus]	48	17	17
P10855	C-C motif chemokine 3 [OS=Mus musculus]	41	2	2
P10923	Osteopontin [OS=Mus musculus]	39	8	8
P11031	Activated RNA polymerase II transcriptional coactivator p15 [OS=Mus musculus]	31	4	4
P11103	Poly [ADP-ribose] polymerase 1 [OS=Mus musculus]	27	22	22
P11152	lipoprotein lipase [OS=Mus musculus]	13	4	4

P11157	ribonucleoside-diphosphate reductase subunit M2 [OS=Mus musculus]	6	2	2
P11352	Glutathione peroxidase 1 [OS=Mus musculus]	43	4	4
P11370	Retrovirus-related Env polyprotein from Fv-4 locus [OS=Mus musculus]	7	6	2
P11438	Lysosome-associated membrane glycoprotein 1 [OS=Mus musculus]	25	6	6
P11440	Cyclin-dependent kinase 1 [OS=Mus musculus]	71	17	15
P11499	Heat shock protein HSP 90-beta [OS=Mus musculus]	71	56	36
P11672	Neutrophil gelatinase-associated lipocalin [OS=Mus musculus]	17	2	2
P11680	Properdin [OS=Mus musculus]	21	7	7
P11688	Integrin alpha-5 [OS=Mus musculus]	10	8	8
P11835	Integrin beta-2 [OS=Mus musculus]	51	32	31
P11928	2'-5'-oligoadenylate synthase 1A [OS=Mus musculus]	55	21	21
P11930	nucleoside diphosphate-linked moiety X motif 19 [OS=Mus musculus]	17	3	3
P11983	T-complex protein 1 subunit alpha [OS=Mus musculus]	60	20	20
P12032	Metalloproteinase inhibitor 1 [OS=Mus musculus]	19	2	2
P12265	beta-glucuronidase [OS=Mus musculus]	30	13	13
P12367	cAMP-dependent protein kinase type II-alpha regulatory subunit [OS=Mus musculus]	34	10	8
P12382	ATP-dependent 6-phosphofructokinase, liver type [OS=Mus musculus]	7	4	2
P12388	plasminogen activator inhibitor 2, macrophage [OS=Mus musculus]	23	5	5
P12787	Cytochrome c oxidase subunit 5A, mitochondrial [OS=Mus musculus]	53	7	7
P12815	programmed cell death protein 6 [OS=Mus musculus]	53	6	6
P12970	60S ribosomal protein L7a [OS=Mus musculus]	48	18	18
P13011	Acyl-CoA desaturase 2 [OS=Mus musculus]	23	4	4
P13020-1	Gelsolin [OS=Mus musculus]	39	17	17
P13439	uridine 5'-monophosphate synthase [OS=Mus musculus]	61	16	16
P13597-1	Intercellular adhesion molecule 1 [OS=Mus musculus]	16	7	7

P13864	DNA (cytosine-5)-methyltransferase 1 [OS=Mus musculus]	17	18	18
P14069	protein S100-A6 [OS=Mus musculus]	62	4	4
P14115	60S ribosomal protein L27a [OS=Mus musculus]	33	8	8
P14131	40S ribosomal protein S16 [OS=Mus musculus]	53	10	10
P14148	60S ribosomal protein L7 [OS=Mus musculus]	47	18	18
P14152	Malate dehydrogenase, cytoplasmic [OS=Mus musculus]	54	12	12
P14206	40S ribosomal protein SA [OS=Mus musculus]	64	19	19
P14211	Calreticulin [OS=Mus musculus]	68	25	25
P14234	Tyrosine-protein kinase Fgr [OS=Mus musculus]	21	8	7
P14576-1	signal recognition particle 54 kDa protein [OS=Mus musculus]	33	12	12
P14685	26S proteasome non-ATPase regulatory subunit 3 [OS=Mus musculus]	55	21	21
P14733	Lamin-B1 [OS=Mus musculus]	25	10	10
P14824	annexin A6 [OS=Mus musculus]	43	20	20
P14869	60S acidic ribosomal protein P0 [OS=Mus musculus]	49	15	15
P14901	heme oxygenase 1 [OS=Mus musculus]	63	16	16
P15379-14	CD44 antigen [OS=Mus musculus]	13	6	6
P15532	Nucleoside diphosphate kinase A [OS=Mus musculus]	74	13	8
P15535	Beta-1,4-galactosyltransferase 1 [OS=Mus musculus]	9	3	2
P15864	Histone H1.2 [OS=Mus musculus]	28	9	3
P16045	Galectin-1 [OS=Mus musculus]	87	9	9
P16110	Galectin-3 [OS=Mus musculus]	33	7	7
P16254	Signal recognition particle 14 kDa protein [OS=Mus musculus]	25	2	2
P16330	2',3'-cyclic-nucleotide 3'-phosphodiesterase [OS=Mus musculus]	24	7	7
P16332	Methylmalonyl-CoA mutase, mitochondrial [OS=Mus musculus]	40	16	16
P16460	Argininosuccinate synthase [OS=Mus musculus]	8	2	2
P16546	Spectrin alpha chain, non-erythrocytic 1 [OS=Mus musculus]	26	41	41

P16627	Heat shock 70 kDa protein 1-like [OS=Mus musculus]	9	5	2
P16675	lysosomal protective protein [OS=Mus musculus]	37	14	14
P16858	glyceraldehyde-3-phosphate dehydrogenase [OS=Mus musculus]	74	18	18
P16879	Tyrosine-protein kinase Fes/Fps [OS=Mus musculus]	11	5	5
P17047-2	Isoform LAMP-2B of Lysosome-associated membrane glycoprotein 2 [OS=Mus musculus]	18	7	7
P17182	alpha-enolase [OS=Mus musculus]	75	32	24
P17183	Gamma-enolase [OS=Mus musculus]	28	9	3
P17225	Polypyrimidine tract-binding protein 1 [OS=Mus musculus]	55	17	16
P17426	AP-2 complex subunit alpha-1 [OS=Mus musculus]	43	29	23
P17427	AP-2 complex subunit alpha-2 [OS=Mus musculus]	49	30	24
P17433	transcription factor PU.1 [OS=Mus musculus]	12	3	3
P17439	glucosylceramidase [OS=Mus musculus]	30	10	10
P17563	Selenium-binding protein 1 [OS=Mus musculus]	14	4	4
P17665	Cytochrome c oxidase subunit 7C, mitochondrial [OS=Mus musculus]	17	2	2
P17710-3	Isoform HK1 of Hexokinase-1 [OS=Mus musculus]	52	39	35
P17742	peptidyl-prolyl cis-trans isomerase A [OS=Mus musculus]	61	10	10
P17751	Triosephosphate isomerase [OS=Mus musculus]	63	17	17
P17809	Solute carrier family 2, facilitated glucose transporter member 1 [OS=Mus musculus]	4	2	2
P17897	Lysozyme c-1 [OS=Mus musculus]	20	2	2
P17918	proliferating cell nuclear antigen [OS=Mus musculus]	75	12	12
P18155	Bifunctional methylenetetrahydrofolate dehydrogenase/cyclohydrolase, mitochondrial [OS=Mus musculus]	69	15	15
P18242	Cathepsin D [OS=Mus musculus]	44	16	16
P18572-1	Basigin [OS=Mus musculus]	23	9	9
P18653	Ribosomal protein S6 kinase alpha-1 [OS=Mus musculus]	20	11	5

P18654	Ribosomal protein S6 kinase alpha-3 [OS=Mus musculus]	27	16	10
P18760	Cofilin-1 [OS=Mus musculus]	70	17	17
P19096	Fatty acid synthase [OS=Mus musculus]	11	17	17
P19157	Glutathione S-transferase P 1 [OS=Mus musculus]	15	2	2
P19246	Neurofilament heavy polypeptide [OS=Mus musculus]	2	2	2
P19253	60S ribosomal protein L13a [OS=Mus musculus]	26	5	5
P19426-1	Negative elongation factor E [OS=Mus musculus]	18	4	4
P19536	Cytochrome c oxidase subunit 5B, mitochondrial [OS=Mus musculus]	39	6	6
P19783	Cytochrome c oxidase subunit 4 isoform 1, mitochondrial [OS=Mus musculus]	50	9	9
P19973	Lymphocyte-specific protein 1 [OS=Mus musculus]	35	6	6
P20029	78 kDa glucose-regulated protein [OS=Mus musculus]	62	40	38
P20060	Beta-hexosaminidase subunit beta [OS=Mus musculus]	43	15	15
P20108	Thioredoxin-dependent peroxide reductase, mitochondrial [OS=Mus musculus]	53	10	10
P20152	Vimentin [OS=Mus musculus]	54	20	20
P20491	High affinity immunoglobulin epsilon receptor subunit gamma [OS=Mus musculus]	33	3	3
P20664	DNA primase small subunit [OS=Mus musculus]	14	4	4
P21107	Tropomyosin alpha-3 chain [OS=Mus musculus]	48	17	4
P21107-2	Isoform 2 of Tropomyosin alpha-3 chain [OS=Mus musculus]	64	25	12
P21126	Ubiquitin-like protein 4A [OS=Mus musculus]	27	3	3
P21279	Guanine nucleotide-binding protein G(Q) subunit alpha [OS=Mus musculus]	40	10	10
P21460	Cystatin-C [OS=Mus musculus]	43	6	6
P21550	Beta-enolase [OS=Mus musculus]	30	9	2
P21855	B-cell differentiation antigen CD72 [OS=Mus musculus]	38	7	7
P21956-1	Lactadherin [OS=Mus musculus]	17	5	5
P21981	Protein-glutamine gamma-glutamyltransferase 2 [OS=Mus musculus]	11	5	5
P22315	Ferrochelatase, mitochondrial [OS=Mus musculus]	18	4	4

P22366-1	Myeloid differentiation primary response protein MyD88 [OS=Mus musculus]	9	2	2
P22437	Prostaglandin G/H synthase 1 [OS=Mus musculus]	25	10	9
P22682	E3 ubiquitin-protein ligase CBL [OS=Mus musculus]	9	6	6
P22752	histone H2A type 1 [OS=Mus musculus]	58	6	3
P22892	AP-1 complex subunit gamma-1 [OS=Mus musculus]	21	11	11
P22893	mRNA decay activator protein ZFP36 [OS=Mus musculus]	26	3	3
P22907-1	porphobilinogen deaminase [OS=Mus musculus]	35	8	8
P23116	Eukaryotic translation initiation factor 3 subunit A [OS=Mus musculus]	39	43	43
P23188	Furin [OS=Mus musculus]	9	5	5
P23198	chromobox protein homolog 3 [OS=Mus musculus]	37	6	4
P23492	purine nucleoside phosphorylase [OS=Mus musculus]	62	12	12
P23506-2	Isoform 2 of Protein-L-isoaspartate(D-aspartate) O-methyltransferase [OS=Mus musculus]	46	6	6
P23591	GDP-L-fucose synthase [OS=Mus musculus]	35	8	8
P23780	Beta-galactosidase [OS=Mus musculus]	19	9	9
P24270	catalase [OS=Mus musculus]	65	27	27
P24288	Branched-chain-amino-acid aminotransferase, cytosolic [OS=Mus musculus]	30	8	8
P24369	peptidyl-prolyl cis-trans isomerase B [OS=Mus musculus]	61	15	15
P24452	Macrophage-capping protein [OS=Mus musculus]	47	8	8
P24527	leukotriene A-4 hydrolase [OS=Mus musculus]	41	18	18
P24547	inosine-5'-monophosphate dehydrogenase 2 [OS=Mus musculus]	61	26	26
P24668	Cation-dependent mannose-6-phosphate receptor [OS=Mus musculus]	35	6	6
P24860	G2/mitotic-specific cyclin-B1 [OS=Mus musculus]	15	4	4
P25085	Interleukin-1 receptor antagonist protein [OS=Mus musculus]	56	8	8
P25119	Tumor necrosis factor receptor superfamily member 1B [OS=Mus musculus]	18	4	4
P25206	DNA replication licensing factor mcm3 [OS=Mus musculus]	60	35	35
P25322	G1/S-specific cyclin-D1 [OS=Mus musculus]	9	2	2

P25425-12	Isoform 12 of POU domain, class 2, transcription factor 1 [OS=Mus musculus]	8	4	4
P25444	40S ribosomal protein S2 [OS=Mus musculus]	41	12	12
P25799	Nuclear factor NF-kappa-B p105 subunit [OS=Mus musculus]	16	9	9
P25911-2	Isoform 2 of Tyrosine-protein kinase Lyn [OS=Mus musculus]	59	21	19
P25976-1	Nucleolar transcription factor 1 [OS=Mus musculus]	4	2	2
P26039	Talin-1 [OS=Mus musculus]	62	105	95
P26040	Ezrin [OS=Mus musculus]	31	16	10
P26041	Moesin [OS=Mus musculus]	54	31	23
P26043	radixin [OS=Mus musculus]	41	20	12
P26151	high affinity immunoglobulin gamma Fc receptor I [OS=Mus musculus]	28	11	11
P26323	Friend leukemia integration 1 transcription factor [OS=Mus musculus]	20	5	5
P26369	Splicing factor U2AF 65 kDa subunit [OS=Mus musculus]	43	12	12
P26443	Glutamate dehydrogenase 1, mitochondrial [OS=Mus musculus]	65	27	27
P26450	Phosphatidylinositol 3-kinase regulatory subunit alpha [OS=Mus musculus]	9	3	3
P26516	26S proteasome non-ATPase regulatory subunit 7 [OS=Mus musculus]	64	11	11
P26638	Serine--tRNA ligase, cytoplasmic [OS=Mus musculus]	56	25	25
P26645	Myristoylated alanine-rich C-kinase substrate [OS=Mus musculus]	22	2	2
P26883	Peptidyl-prolyl cis-trans isomerase FKBP1A [OS=Mus musculus]	42	5	5
P27046	Alpha-mannosidase 2 [OS=Mus musculus]	8	6	6
P27512	Tumor necrosis factor receptor superfamily member 5 [OS=Mus musculus]	32	5	5
P27546-1	Microtubule-associated protein 4 [OS=Mus musculus]	21	12	12
P27601	Guanine nucleotide-binding protein subunit alpha-13 [OS=Mus musculus]	37	12	11
P27659	60S ribosomal protein L3 [OS=Mus musculus]	28	9	9

P27773	Protein disulfide-isomerase A3 [OS=Mus musculus]	69	41	40
P27870	Proto-oncogene vav [OS=Mus musculus]	8	5	5
P28063	Proteasome subunit beta type-8 [OS=Mus musculus]	42	9	9
P28076	Proteasome subunit beta type-9 [OS=Mus musculus]	54	6	6
P28271	Cytoplasmic aconitate hydratase [OS=Mus musculus]	16	8	8
P28352	DNA-(apurinic or apyrimidinic site) lyase [OS=Mus musculus]	56	14	14
P28474	alcohol dehydrogenase class-3 [OS=Mus musculus]	16	3	3
P28650	Adenylosuccinate synthetase isozyme 1 [OS=Mus musculus]	44	15	14
P28656	Nucleosome assembly protein 1-like 1 [OS=Mus musculus]	63	16	14
P28658	Ataxin-10 [OS=Mus musculus]	37	12	12
P28659-4	Isoform 4 of CUGBP Elav-like family member 1 [OS=Mus musculus]	12	3	2
P28667	MARCKS-related protein [OS=Mus musculus]	62	5	5
P28704	Retinoic acid receptor RXR-beta [OS=Mus musculus]	10	4	4
P28740-1	Isoform 1 of Kinesin-like protein KIF2A [OS=Mus musculus]	4	2	2
P28798	Granulins [OS=Mus musculus]	32	12	12
P29341	Polyadenylate-binding protein 1 [OS=Mus musculus]	53	27	27
P29351-2	Isoform 2 of Tyrosine-protein phosphatase non-receptor type 6 [OS=Mus musculus]	63	28	28
P29391	Ferritin light chain 1 [OS=Mus musculus]	49	8	8
P29416	Beta-hexosaminidase subunit alpha [OS=Mus musculus]	38	17	17
P29452	caspase-1 [OS=Mus musculus]	34	10	10
P29477	Nitric oxide synthase, inducible [OS=Mus musculus]	41	30	30
P29595	NEDD8 [OS=Mus musculus]	41	3	3
P29758	Ornithine aminotransferase, mitochondrial [OS=Mus musculus]	76	21	21
P30204-1	Macrophage scavenger receptor types I and II [OS=Mus musculus]	19	6	6
P30285	Cyclin-dependent kinase 4 [OS=Mus musculus]	53	10	9
P30355	Arachidonate 5-lipoxygenase-activating protein [OS=Mus musculus]	25	4	4
P30412	peptidyl-prolyl cis-trans isomerase C [OS=Mus musculus]	43	5	5

P30416	Peptidyl-prolyl cis-trans isomerase FKBP4 [OS=Mus musculus]	64	25	25
P30681	High mobility group protein B2 [OS=Mus musculus]	49	14	12
P30999-1	Catenin delta-1 [OS=Mus musculus]	37	25	25
P31230	aminoacyl tRNA synthase complex-interacting multifunctional protein 1 [OS=Mus musculus]	59	10	10
P31266-1	Recombining binding protein suppressor of hairless [OS=Mus musculus]	10	4	4
P31750	RAC-alpha serine/threonine-protein kinase [OS=Mus musculus]	5	2	2
P31786	acyl-CoA-binding protein [OS=Mus musculus]	68	6	6
P31938	Dual specificity mitogen-activated protein kinase kinase 1 [OS=Mus musculus]	43	11	9
P31996-1	Macrosialin [OS=Mus musculus]	22	6	6
P32020	Non-specific lipid-transfer protein [OS=Mus musculus]	20	13	13
P32067	Lupus La protein homolog [OS=Mus musculus]	38	16	16
P32233	developmentally-regulated GTP-binding protein 1 [OS=Mus musculus]	54	14	14
P32883-1	GTPase KRas [OS=Mus musculus]	56	7	2
P32921	Tryptophan--tRNA ligase, cytoplasmic [OS=Mus musculus]	57	19	19
P33609	DNA polymerase alpha catalytic subunit [OS=Mus musculus]	6	5	5
P33610	DNA primase large subunit [OS=Mus musculus]	25	9	9
P33611	DNA polymerase alpha subunit B [OS=Mus musculus]	20	7	7
P34022	Ran-specific GTPase-activating protein [OS=Mus musculus]	55	6	6
P34152	Focal adhesion kinase 1 [OS=Mus musculus]	9	6	6
P34884	Macrophage Migration inhibitory factor [OS=Mus musculus]	43	5	5
P35123	Ubiquitin carboxyl-terminal hydrolase 4 [OS=Mus musculus]	27	16	15
P35235	tyrosine-protein phosphatase non-receptor type 11 [OS=Mus musculus]	23	9	9
P35278	Ras-related protein Rab-5C [OS=Mus musculus]	66	9	6
P35282	Ras-related protein Rab-21 [OS=Mus musculus]	54	10	10
P35293	Ras-related protein Rab-18 [OS=Mus musculus]	59	11	11
P35456	Urokinase plasminogen activator surface receptor [OS=Mus musculus]	14	3	3

P35486	Pyruvate dehydrogenase E1 component subunit alpha, somatic form, mitochondrial [OS=Mus musculus]	42	11	11
P35550	rRNA 2'-O-methyltransferase fibrillarin [OS=Mus musculus]	47	11	11
P35564	Calnexin [OS=Mus musculus]	57	30	30
P35585	AP-1 complex subunit mu-1 [OS=Mus musculus]	19	6	6
P35601-1	Replication factor C subunit 1 [OS=Mus musculus]	10	7	7
P35700	peroxiredoxin-1 [OS=Mus musculus]	89	26	24
P35821	Tyrosine-protein phosphatase non-receptor type 1 [OS=Mus musculus]	33	9	9
P35831	Tyrosine-protein phosphatase non-receptor type 12 [OS=Mus musculus]	5	3	3
P35951	Low-density lipoprotein receptor [OS=Mus musculus]	23	12	12
P35979	60S ribosomal protein L12 [OS=Mus musculus]	59	7	7
P35980	60S ribosomal protein L18 [OS=Mus musculus]	26	6	6
P35991	Tyrosine-protein kinase BTK [OS=Mus musculus]	19	9	9
P36536	GTP-binding protein SAR1a [OS=Mus musculus]	65	7	6
P36552	Oxygen-dependent coproporphyrinogen-III oxidase, mitochondrial [OS=Mus musculus]	35	11	11
P36916	Guanine nucleotide-binding protein-like 1 [OS=Mus musculus]	14	5	5
P36993-5	Isoform Beta-5 of Protein phosphatase 1B [OS=Mus musculus]	9	2	2
P37040	NADPH--cytochrome P450 reductase [OS=Mus musculus]	63	33	33
P37913	DNA ligase 1 [OS=Mus musculus]	41	25	25
P38060	Hydroxymethylglutaryl-CoA lyase, mitochondrial [OS=Mus musculus]	44	10	10
P38647	Stress-70 protein, mitochondrial [OS=Mus musculus]	67	36	36
P39053	Dynamin-1 [OS=Mus musculus]	10	7	2
P39054-1	Dynamin-2 [OS=Mus musculus]	42	26	21
P39749	Flap endonuclease 1 [OS=Mus musculus]	31	9	9
P40124	adenylyl cyclase-associated protein 1 [OS=Mus musculus]	66	24	24
P40142	Transketolase [OS=Mus musculus]	71	31	31
P40240	CD9 antigen [OS=Mus musculus]	26	3	3

P40336	Vacuolar protein sorting-associated protein 26A [OS=Mus musculus]	47	10	10
P40630-1	Transcription factor A, mitochondrial [OS=Mus musculus]	20	4	4
P41105	60S ribosomal protein L28 [OS=Mus musculus]	26	3	3
P41216	Long-chain-fatty-acid--CoA ligase 1 [OS=Mus musculus]	9	5	3
P41241	tyrosine-protein kinase CSK [OS=Mus musculus]	39	12	12
P42125	Enoyl-CoA delta isomerase 1, mitochondrial [OS=Mus musculus]	42	8	8
P42128	Forkhead box protein K1 [OS=Mus musculus]	4	2	2
P42208	septin-2 [OS=Mus musculus]	52	11	11
P42225	signal transducer and activator of transcription 1 [OS=Mus musculus]	31	18	18
P42227	Signal transducer and activator of transcription 3 [OS=Mus musculus]	11	5	5
P42567-1	epidermal growth factor receptor substrate 15 [OS=Mus musculus]	19	12	12
P42669	Transcriptional activator protein Pur-alpha [OS=Mus musculus]	37	5	5
P42932	T-complex protein 1 subunit theta [OS=Mus musculus]	68	36	36
P43024	Cytochrome c oxidase subunit 6A1, mitochondrial [OS=Mus musculus]	61	4	4
P43247	DNA mismatch repair protein MSH2 [OS=Mus musculus]	14	9	9
P43274	Histone H1.4 [OS=Mus musculus]	26	7	3
P43275	Histone H1.1 [OS=Mus musculus]	29	5	4
P43276	Histone H1.5 [OS=Mus musculus]	19	4	4
P43346	deoxycytidine kinase [OS=Mus musculus]	29	5	5
P43406	Integrin alpha-V [OS=Mus musculus]	9	6	6
P45376	aldose reductase [OS=Mus musculus]	41	8	8
P45377	Aldose reductase-related protein 2 [OS=Mus musculus]	54	14	10
P45878	Peptidyl-prolyl cis-trans isomerase FKBP2 [OS=Mus musculus]	51	6	6
P45952	medium-chain specific acyl-CoA dehydrogenase, mitochondrial [OS=Mus musculus]	39	13	13
P46061	Ran GTPase-activating protein 1 [OS=Mus musculus]	47	21	21
P46460	Vesicle-fusing ATPase [OS=Mus musculus]	48	30	30
P46467	Vacuolar protein sorting-associated protein 4B [OS=Mus musculus]	36	12	9

P46471	26S proteasome regulatory subunit 7 [OS=Mus musculus]	68	28	28
P46638	Ras-related protein Rab-11B [OS=Mus musculus]	56	13	2
P46660	alpha-internexin [OS=Mus musculus]	6	2	2
P46664	adenylosuccinate synthetase isozyme 2 [OS=Mus musculus]	51	16	15
P47199	Quinone oxidoreductase [OS=Mus musculus]	24	4	4
P47226-1	Testin [OS=Mus musculus]	20	6	6
P47713	Cytosolic phospholipase A2 [OS=Mus musculus]	45	28	28
P47738	Aldehyde dehydrogenase, mitochondrial [OS=Mus musculus]	75	29	28
P47740	Fatty aldehyde dehydrogenase [OS=Mus musculus]	26	8	8
P47753	F-actin-capping protein subunit alpha-1 [OS=Mus musculus]	66	14	12
P47754	F-actin-capping protein subunit alpha-2 [OS=Mus musculus]	72	15	13
P47757-2	Isoform 2 of F-actin-capping protein subunit beta [OS=Mus musculus]	66	16	16
P47758	signal recognition particle receptor subunit beta [OS=Mus musculus]	62	16	16
P47791-2	Isoform Cytoplasmic of Glutathione reductase, mitochondrial [OS=Mus musculus]	51	14	14
P47802	Metaxin-1 [OS=Mus musculus]	45	12	12
P47809	Dual specificity mitogen-activated protein kinase kinase 4 [OS=Mus musculus]	8	2	2
P47811	Mitogen-activated protein kinase 14 [OS=Mus musculus]	52	12	12
P47856	glutamine--fructose-6-phosphate aminotransferase [isomerizing] 1 [OS=Mus musculus]	28	12	12
P47911	60S ribosomal protein L6 [OS=Mus musculus]	36	12	12
P47930-1	Fos-related antigen 2 [OS=Mus musculus]	5	2	2
P47934	Carnitine O-acetyltransferase [OS=Mus musculus]	9	2	2
P47941	Crk-like protein [OS=Mus musculus]	28	5	5
P47955	60S acidic ribosomal protein P1 [OS=Mus musculus]	80	4	4
P47962	60S ribosomal protein L5 [OS=Mus musculus]	34	10	10
P47963	60S ribosomal protein L13 [OS=Mus musculus]	36	8	8

P47964	60S ribosomal protein L36 [OS=Mus musculus]	21	3	3
P47968	ribose-5-phosphate isomerase [OS=Mus musculus]	14	3	3
P48024	eukaryotic translation initiation factor 1 [OS=Mus musculus]	81	7	4
P48036	annexin A5 [OS=Mus musculus]	76	2<br>6	26
P48193	protein 4.1 [OS=Mus musculus]	17	9	9
P48377	MHC class II regulatory factor RFX1 [OS=Mus musculus]	5	2	2
P48428	Tubulin-specific chaperone A [OS=Mus musculus]	26	2	2
P48453-1	serine/threonine-protein phosphatase 2B catalytic subunit beta isoform [OS=Mus musculus]	20	7	3
P48678-1	Prelamin-A/C [OS=Mus musculus]	58	3<br>4	34
P48722	Heat shock 70 kDa protein 4L [OS=Mus musculus]	16	9	7
P48758	Carbonyl reductase [NADPH] 1 [OS=Mus musculus]	34	6	5
P48771	Cytochrome c oxidase subunit 7A2, mitochondrial [OS=Mus musculus]	28	2	2
P48962	ADP/ATP translocase 1 [OS=Mus musculus]	43	1<br>4	6
P49138	MAP kinase-activated protein kinase 2 [OS=Mus musculus]	30	7	7
P49300	C-type lectin domain family 10 member A [OS=Mus musculus]	24	3	3
P49312-1	Heterogeneous nuclear ribonucleoprotein A1 [OS=Mus musculus]	42	1<br>6	14
P49442	Inositol polyphosphate 1-phosphatase [OS=Mus musculus]	12	3	3
P49586	choline-phosphate cytidylyltransferase A [OS=Mus musculus]	10	2	2
P49615	Cyclin-dependent-like kinase 5 [OS=Mus musculus]	8	3	2
P49710	Hematopoietic lineage cell-specific protein [OS=Mus musculus]	37	1<br>3	13
P49717	DNA replication licensing factor MCM4 [OS=Mus musculus]	45	2<br>8	28
P49718	DNA replication licensing factor mcm5 [OS=Mus musculus]	43	2<br>1	21
P49722	Proteasome subunit alpha type-2 [OS=Mus musculus]	55	9	9
P49769	Presenilin-1 [OS=Mus musculus]	7	2	2
P50096	Inosine-5'-monophosphate dehydrogenase 1 [OS=Mus musculus]	32	1<br>2	12
P50136	2-oxoisovalerate dehydrogenase subunit alpha, mitochondrial [OS=Mus musculus]	31	8	8
P50171-2	Isoform Long of Estradiol 17-beta-dehydrogenase 8 [OS=Mus musculus]	33	6	6
P50247	Adenosylhomocysteinase [OS=Mus musculus]	41	1<br>2	12

P50396	Rab GDP dissociation inhibitor alpha [OS=Mus musculus]	40	15	9
P50429	arylsulfatase B [OS=Mus musculus]	26	9	9
P50431	Serine hydroxymethyltransferase, cytosolic [OS=Mus musculus]	22	7	7
P50516-1	V-type proton ATPase catalytic subunit A [OS=Mus musculus]	75	32	32
P50518	V-type proton ATPase subunit E 1 [OS=Mus musculus]	42	7	7
P50543	protein S100-A11 [OS=Mus musculus]	64	3	3
P50544	Very long-chain specific acyl-CoA dehydrogenase, mitochondrial [OS=Mus musculus]	42	18	18
P50580	proliferation-associated protein 2G4 [OS=Mus musculus]	69	23	23
P50637	translocator protein [OS=Mus musculus]	51	4	4
P51125-1	Calpastatin [OS=Mus musculus]	21	7	7
P51150	ras-related protein Rab-7a [OS=Mus musculus]	79	16	16
P51174	Long-chain specific acyl-CoA dehydrogenase, mitochondrial [OS=Mus musculus]	39	16	16
P51175	protoporphyrinogen oxidase [OS=Mus musculus]	10	2	2
P51410	60S ribosomal protein L9 [OS=Mus musculus]	55	9	9
P51432	1-phosphatidylinositol 4,5-bisphosphate phosphodiesterase beta-3 [OS=Mus musculus]	7	6	6
P51569	alpha-galactosidase A [OS=Mus musculus]	8	3	3
P51660	peroxisomal multifunctional enzyme type 2 [OS=Mus musculus]	21	10	10
P51670	C-C motif chemokine 9 [OS=Mus musculus]	27	2	2
P51807	Dynein light chain Tctex-type 1 [OS=Mus musculus]	58	4	4
P51855	glutathione synthetase [OS=Mus musculus]	6	2	2
P51859	hepatoma-derived growth factor [OS=Mus musculus]	69	13	13
P51863	V-type proton ATPase subunit d 1 [OS=Mus musculus]	53	15	15
P51881	ADP/ATP translocase 2 [OS=Mus musculus]	50	17	9
P51912	Neutral amino acid transporter B(0) [OS=Mus musculus]	19	8	8
P51943	Cyclin-A2 [OS=Mus musculus]	17	4	4
P51949	CDK-activating kinase assembly factor MAT1 [OS=Mus musculus]	16	3	3
P52293	Importin subunit alpha-1 [OS=Mus musculus]	58	16	16
P52431	DNA polymerase delta catalytic subunit [OS=Mus musculus]	9	6	6

P52432	DNA-directed RNA polymerases I and III subunit RPAC1 [OS=Mus musculus]	18	3	3
P52479-2	Isoform 2 of Ubiquitin carboxyl-terminal hydrolase 10 [OS=Mus musculus]	16	7	7
P52480	Pyruvate kinase PKM [OS=Mus musculus]	70	37	5
P52480-2	Isoform M1 of Pyruvate kinase PKM [OS=Mus musculus]	69	35	3
P52482	Ubiquitin-conjugating enzyme E2 E1 [OS=Mus musculus]	31	3	3
P52503	NADH dehydrogenase [ubiquinone] iron-sulfur protein 6, mitochondrial [OS=Mus musculus]	53	5	5
P52624	uridine phosphorylase 1 [OS=Mus musculus]	40	8	8
P52633	Signal transducer and transcription activator 6 [OS=Mus musculus]	19	8	8
P52825	Carnitine O-palmitoyltransferase 2, mitochondrial [OS=Mus musculus]	25	10	10
P52912	nucleolysin TIA-1 [OS=Mus musculus]	18	4	3
P53026	60S ribosomal protein L10A [OS=Mus musculus]	50	13	13
P53395	Lipoamide acyltransferase component of branched-chain alpha-keto acid dehydrogenase complex, mitochondrial [OS=Mus musculus]	37	11	11
P53564	Homeobox protein cut-like 1 [OS=Mus musculus]	9	7	4
P53702	Cytochrome c-type heme lyase [OS=Mus musculus]	30	6	6
P53798	squalene synthase [OS=Mus musculus]	6	2	2
P53810	Phosphatidylinositol transfer protein alpha isoform [OS=Mus musculus]	41	10	9
P53811	Phosphatidylinositol transfer protein beta isoform [OS=Mus musculus]	52	9	8
P53994	Ras-related protein Rab-2A [OS=Mus musculus]	67	13	13
P54071	Isocitrate dehydrogenase [NADP], mitochondrial [OS=Mus musculus]	60	25	24
P54103	dnaJ homolog subfamily C member 2 [OS=Mus musculus]	30	11	11
P54116	erythrocyte band 7 integral membrane protein [OS=Mus musculus]	30	5	5
P54227	Stathmin [OS=Mus musculus]	24	4	4
P54276	DNA mismatch repair protein MSH6 [OS=Mus musculus]	2	2	2
P54726	UV excision repair protein RAD23 homolog A [OS=Mus musculus]	21	3	3
P54728	UV excision repair protein RAD23 homolog B [OS=Mus musculus]	36	9	9
P54729	NEDD8 ultimate buster 1 [OS=Mus musculus]	18	6	6
P54731	fas-associated factor 1 [OS=Mus musculus]	14	6	6

P54751	CMP-N-acetylneuraminate-beta-galactosamide-alpha-2,3-sialyltransferase 1 [OS=Mus musculus]	9	2	2
P54775	26S proteasome regulatory subunit 6B [OS=Mus musculus]	73	19	19
P54822	adenylosuccinate lyase [OS=Mus musculus]	54	20	20
P54823	Probable ATP-dependent RNA helicase DDX6 [OS=Mus musculus]	17	5	5
P54923	[Protein ADP-ribosylarginine] hydrolase [OS=Mus musculus]	24	5	5
P54987	cis-aconitate decarboxylase [OS=Mus musculus]	75	27	27
P55096	ATP-binding cassette sub-family D member 3 [OS=Mus musculus]	10	4	4
P55194	SH3 domain-binding protein 1 [OS=Mus musculus]	13	7	7
P55264	Adenosine kinase [OS=Mus musculus]	50	12	12
P55302	alpha-2-macroglobulin receptor-associated protein [OS=Mus musculus]	24	8	8
P55937	Golgin subfamily A member 3 [OS=Mus musculus]	17	16	16
P56135	ATP synthase subunit f, mitochondrial [OS=Mus musculus]	38	4	4
P56183-1	Ribosomal RNA processing protein 1 homolog A [OS=Mus musculus]	14	4	4
P56213	FAD-linked sulfhydryl oxidase ALR [OS=Mus musculus]	12	2	2
P56376	Acylphosphatase-1 [OS=Mus musculus]	26	2	2
P56391	Cytochrome c oxidase subunit 6B1 [OS=Mus musculus]	71	7	7
P56394	cytochrome C oxidase copper chaperone [OS=Mus musculus]	63	2	2
P56395	Cytochrome b5 [OS=Mus musculus]	43	6	6
P56399	Ubiquitin carboxyl-terminal hydrolase 5 [OS=Mus musculus]	48	25	25
P56477	interferon regulatory factor 5 [OS=Mus musculus]	21	7	7
P56480	ATP synthase subunit beta, mitochondrial [OS=Mus musculus]	77	30	30
P56542	Deoxyribonuclease-2-alpha [OS=Mus musculus]	16	4	4
P56546-2	Isoform 2 of C-terminal-binding protein 2 [OS=Mus musculus]	12	8	7
P56812	Programmed cell death protein 5 [OS=Mus musculus]	37	4	4
P56873	Sjoegren syndrome/scleroderma autoantigen 1 homolog [OS=Mus musculus]	46	4	4
P56959	RNA-binding protein FUS [OS=Mus musculus]	23	10	10
P56960	exosome component 10 [OS=Mus musculus]	27	14	14
P57080-1	ubiquitin carboxyl-terminal hydrolase 25 [OS=Mus musculus]	11	8	8
P57716	Nicastrin [OS=Mus musculus]	16	8	8

P57725	SAM domain-containing protein SAMSN-1 [OS=Mus musculus]	19	3	2
P57746	V-type proton ATPase subunit D [OS=Mus musculus]	21	2	2
P57759	Endoplasmic reticulum resident protein 29 [OS=Mus musculus]	47	13	13
P57776-1	Elongation factor 1-delta [OS=Mus musculus]	62	13	2
P57780	Alpha-actinin-4 [OS=Mus musculus]	62	45	30
P57784	U2 small nuclear ribonucleoprotein A' [OS=Mus musculus]	62	13	13
P57787	Monocarboxylate transporter 4 [OS=Mus musculus]	8	3	3
P58044	Isopentenyl-diphosphate Delta-isomerase 1 [OS=Mus musculus]	53	8	8
P58058	NAD kinase [OS=Mus musculus]	28	8	8
P58064	28S ribosomal protein S6, mitochondrial [OS=Mus musculus]	38	4	4
P58242	acid sphingomyelinase-like phosphodiesterase 3B [OS=Mus musculus]	39	10	10
P58252	Elongation factor 2 [OS=Mus musculus]	67	47	47
P58281-2	Isoform 2 of Dynamin-like 120 kDa protein, mitochondrial [OS=Mus musculus]	26	16	16
P58389	Serine/threonine-protein phosphatase 2A activator [OS=Mus musculus]	43	8	8
P58404-1	Striatin-4 [OS=Mus musculus]	8	4	4
P58462-3	Isoform 3 of Forkhead box protein P1 [OS=Mus musculus]	16	4	4
P58468	protein FAM207A [OS=Mus musculus]	42	6	6
P58742	Aladin [OS=Mus musculus]	11	4	4
P58771-1	Tropomyosin alpha-1 chain [OS=Mus musculus]	31	10	2
P58854	Gamma-tubulin complex component 3 [OS=Mus musculus]	5	3	3
P59016	Vacuolar protein sorting-associated protein 33B [OS=Mus musculus]	4	2	2
P59017	Bcl-2-like protein 13 [OS=Mus musculus]	15	4	4
P59108	Copine-2 [OS=Mus musculus]	4	2	2
P59235	Nucleoporin Nup43 [OS=Mus musculus]	19	4	4
P59325	Eukaryotic translation initiation factor 5 [OS=Mus musculus]	29	10	10
P59326	YTH domain-containing family protein 1 [OS=Mus musculus]	15	4	3
P59328	WD repeat and HMG-box DNA-binding protein 1 [OS=Mus musculus]	16	11	11
P59481	VIP36-like protein [OS=Mus musculus]	11	3	3
P59708	Splicing factor 3B subunit 6 [OS=Mus musculus]	32	3	3

P59808	SAM and SH3 domain-containing protein 1 [OS=Mus musculus]	5	4	3
P59999	Actin-related protein 2/3 complex subunit 4 [OS=Mus musculus]	68	10	10
P60060	Protein transport protein Sec61 subunit gamma [OS=Mus musculus]	37	2	2
P60122	RuvB-like 1 [OS=Mus musculus]	69	21	21
P60202	Myelin proteolipid protein [OS=Mus musculus]	12	3	3
P60229	Eukaryotic translation initiation factor 3 subunit E [OS=Mus musculus]	67	23	23
P60335	Poly(RC)-binding protein 1 [OS=Mus musculus]	72	14	11
P60521	Gamma-aminobutyric acid receptor-associated protein-like 2 [OS=Mus musculus]	20	2	2
P60670-1	Nuclear protein localization protein 4 homolog [OS=Mus musculus]	28	11	11
P60710	Actin, cytoplasmic 1 [OS=Mus musculus]	93	34	2
P60762	Mortality factor 4-like protein 1 [OS=Mus musculus]	38	10	8
P60766-2	Cell division control protein 42 homolog [OS=Mus musculus]	64	10	9
P60843	Eukaryotic initiation factor 4A-I [OS=Mus musculus]	79	26	25
P60867	40S ribosomal protein S20 [OS=Mus musculus]	32	6	6
P60898	DNA-directed RNA polymerase II subunit RPB9 [OS=Mus musculus]	29	2	2
P60904	DnaJ homolog subfamily C member 5 [OS=Mus musculus]	46	4	4
P61021	Ras-related protein Rab-5B [OS=Mus musculus]	28	5	2
P61022	Calcineurin B homologous protein 1 [OS=Mus musculus]	41	7	7
P61027	ras-related protein rab-10 [OS=Mus musculus]	30	6	3
P61028	Ras-related protein Rab-8B [OS=Mus musculus]	22	5	2
P61079	Ubiquitin-conjugating enzyme E2 D3 [OS=Mus musculus]	43	4	3
P61082	NEDD8-conjugating enzyme Ubc12 [OS=Mus musculus]	24	4	4
P61087	Ubiquitin-conjugating enzyme E2 K [OS=Mus musculus]	57	8	8
P61089	ubiquitin-conjugating enzyme E2 N [OS=Mus musculus]	72	9	8
P61161	Actin-related protein 2 [OS=Mus musculus]	53	15	15
P61164	Alpha-centractin [OS=Mus musculus]	53	12	5
P61202-1	COP9 signalosome complex subunit 2 [OS=Mus musculus]	45	15	15
P61211	ADP-ribosylation factor-like protein 1 [OS=Mus musculus]	50	6	6

P61222	ATP-binding cassette sub-family E member 1 [OS=Mus musculus]	8	3	3
P61226	ras-related protein Rap-2b [OS=Mus musculus]	51	8	4
P61255	60S ribosomal protein L26 [OS=Mus musculus]	28	6	6
P61290	Proteasome activator complex subunit 3 [OS=Mus musculus]	45	11	11
P61327	Protein mago nashi homolog [OS=Mus musculus]	52	7	7
P61358	60S ribosomal protein L27 [OS=Mus musculus]	49	8	8
P61514	60S ribosomal protein L37a [OS=Mus musculus]	33	2	2
P61600	N-alpha-acetyltransferase 20 [OS=Mus musculus]	15	2	2
P61620	Protein transport protein Sec61 subunit alpha isoform 1 [OS=Mus musculus]	13	3	3
P61750	ADP-ribosylation factor 4 [OS=Mus musculus]	71	10	3
P61759	Prefoldin subunit 3 [OS=Mus musculus]	18	3	3
P61804	Dolichyl-diphosphooligosaccharide--protein glycosyltransferase subunit DAD1 [OS=Mus musculus]	28	3	3
P61924	Coatomer subunit zeta-1 [OS=Mus musculus]	44	7	7
P61961	Ubiquitin-fold modifier 1 [OS=Mus musculus]	59	2	2
P61965	WD repeat-containing protein 5 [OS=Mus musculus]	20	4	4
P61971	nuclear transport factor 2 [OS=Mus musculus]	89	6	6
P61979-2	Isoform 2 of Heterogeneous nuclear ribonucleoprotein K [OS=Mus musculus]	58	22	2
P61982	14-3-3 protein gamma [OS=Mus musculus]	67	14	10
P62046	Leucine-rich repeat and calponin homology domain-containing protein 1 [OS=Mus musculus]	5	2	2
P62075	mitochondrial import inner membrane translocase subunit TIM13 [OS=Mus musculus]	54	4	4
P62077	mitochondrial import inner membrane translocase subunit Tim8 B [OS=Mus musculus]	29	2	2
P62082	40S ribosomal protein S7 [OS=Mus musculus]	52	10	10
P62137	serine/threonine-protein phosphatase PP1-alpha catalytic subunit [OS=Mus musculus]	69	21	7
P62141	Serine/threonine-protein phosphatase PP1-beta catalytic subunit [OS=Mus musculus]	61	18	5
P62192	26S proteasome regulatory subunit 4 [OS=Mus musculus]	52	18	17
P62196	26S proteasome regulatory subunit 8 [OS=Mus musculus]	56	19	18
P62204	Calmodulin [OS=Mus musculus]	81	8	8

P62242	40S ribosomal protein S8 [OS=Mus musculus]	54	12	12
P62245	40S ribosomal protein S15a [OS=Mus musculus]	64	11	11
P62254	Ubiquitin-conjugating enzyme E2 G1 [OS=Mus musculus]	16	2	2
P62257	ubiquitin-conjugating enzyme E2 H [OS=Mus musculus]	16	2	2
P62259	14-3-3 protein epsilon [OS=Mus musculus]	82	20	18
P62264	40S ribosomal protein S14 [OS=Mus musculus]	62	11	11
P62267	40S ribosomal protein S23 [OS=Mus musculus]	27	3	3
P62270	40S ribosomal protein S18 [OS=Mus musculus]	54	11	11
P62281	40S ribosomal protein S11 [OS=Mus musculus]	42	9	9
P62301	40S ribosomal protein S13 [OS=Mus musculus]	45	11	11
P62305	small nuclear ribonucleoprotein E [OS=Mus musculus]	57	4	4
P62307	Small nuclear ribonucleoprotein F [OS=Mus musculus]	40	2	2
P62309	Small nuclear ribonucleoprotein G [OS=Mus musculus]	17	2	2
P62311	U6 snRNA-associated Sm-like protein LSm3 [OS=Mus musculus]	40	3	3
P62313	U6 snRNA-associated Sm-like protein LSm6 [OS=Mus musculus]	46	2	2
P62315	Small nuclear ribonucleoprotein Sm D1 [OS=Mus musculus]	55	5	5
P62317	Small nuclear ribonucleoprotein Sm D2 [OS=Mus musculus]	51	7	7
P62320	small nuclear ribonucleoprotein sm d3 [OS=Mus musculus]	48	4	4
P62331	ADP-ribosylation factor 6 [OS=Mus musculus]	69	11	11
P62334	26S proteasome regulatory subunit 10B [OS=Mus musculus]	62	18	18
P62342	Thioredoxin reductase-like selenoprotein T [OS=Mus musculus]	17	2	2
P62482	Voltage-gated potassium channel subunit beta-2 [OS=Mus musculus]	36	7	7
P62488	DNA-directed RNA polymerase II subunit rpb7 [OS=Mus musculus]	23	3	3
P62492	Ras-related protein Rab-11A [OS=Mus musculus]	68	13	2
P62627	Dynein light chain roadblock-type 1 [OS=Mus musculus]	60	4	4
P62700	Protein yippee-like 5 [OS=Mus musculus]	21	2	2
P62702	40S ribosomal protein S4, X isoform [OS=Mus musculus]	53	16	16
P62717	60S ribosomal protein L18a [OS=Mus musculus]	38	10	10
P62748	Hippocalcin-like protein 1 [OS=Mus musculus]	25	4	4

P62751	60S ribosomal protein L23a [OS=Mus musculus]	44	9	9
P62754	40S RIBOSOMAL PROTEIN S6 [OS=Mus musculus]	34	9	9
P62774	Myotrophin [OS=Mus musculus]	78	7	7
P62806	histone H4 [OS=Mus musculus]	57	10	10
P62814	V-type proton ATPase subunit B, brain isoform [OS=Mus musculus]	62	21	21
P62821	Ras-related protein Rab-1A [OS=Mus musculus]	70	10	6
P62827	GTP-binding nuclear protein RAN [OS=Mus musculus]	45	11	11
P62830	60S ribosomal protein L23 [OS=Mus musculus]	39	6	6
P62835	ras-related protein Rap-1A [OS=Mus musculus]	83	15	5
P62843	40S ribosomal protein S15 [OS=Mus musculus]	56	5	5
P62849-1	40S ribosomal protein S24 [OS=Mus musculus]	36	6	6
P62852	40S ribosomal protein S25 [OS=Mus musculus]	26	5	5
P62855	40S ribosomal protein S26 [OS=Mus musculus]	44	4	4
P62858	40S ribosomal protein S28 [OS=Mus musculus]	61	7	7
P62862	40S ribosomal protein S30 [OS=Mus musculus]	20	3	3
P62869	Elongin-B [OS=Mus musculus]	78	7	7
P62874	Guanine nucleotide-binding protein G(I)/G(S)/G(T) subunit beta-1 [OS=Mus musculus]	64	14	10
P62878	E3 ubiquitin-protein ligase RBX1 [OS=Mus musculus]	31	3	3
P62880	Guanine nucleotide-binding protein G(I)/G(S)/G(T) subunit beta-2 [OS=Mus musculus]	62	14	8
P62889	60S ribosomal protein L30 [OS=Mus musculus]	51	5	5
P62897	Cytochrome c, somatic [OS=Mus musculus]	52	7	7
P62900	60S ribosomal protein L31 [OS=Mus musculus]	26	4	4
P62908	40S ribosomal protein S3 [OS=Mus musculus]	60	16	16
P62911	60S ribosomal protein L32 [OS=Mus musculus]	35	7	7
P62915	Transcription initiation factor IIB [OS=Mus musculus]	30	6	6
P62918	60S ribosomal protein L8 [OS=Mus musculus]	37	7	7
P62960	Nuclease-sensitive element-binding protein 1 [OS=Mus musculus]	63	13	8
P62962	profilin-1 [OS=Mus musculus]	89	13	13
P62983	Ubiquitin-40S ribosomal protein S27a [OS=Mus musculus]	63	11	11
P63001	Ras-related C3 botulinum toxin substrate 1 [OS=Mus musculus]	56	9	5

P63017	Heat shock cognate 71 kDa protein [OS=Mus musculus]	76	42	32
P63024	Vesicle-associated membrane protein 3 [OS=Mus musculus]	55	5	5
P63028	Translationally-controlled tumor protein [OS=Mus musculus]	76	7	7
P63030	Mitochondrial pyruvate carrier 1 [OS=Mus musculus]	43	2	2
P63037	DnaJ homolog subfamily A member 1 [OS=Mus musculus]	62	17	17
P63038-1	60 kDa heat shock protein, mitochondrial [OS=Mus musculus]	78	46	46
P63073	Eukaryotic translation initiation factor 4E [OS=Mus musculus]	52	7	7
P63082	V-type proton ATPase 16 kDa proteolipid subunit [OS=Mus musculus]	32	2	2
P63085	mitogen-activated protein kinase 1 [OS=Mus musculus]	68	16	11
P63087-2	Isoform Gamma-2 of Serine/threonine-protein phosphatase PP1-gamma catalytic subunit [OS=Mus musculus]	62	18	3
P63101	14-3-3 protein zeta/delta [OS=Mus musculus]	70	18	15
P63158	High mobility group protein B1 [OS=Mus musculus]	53	13	11
P63163	small nuclear ribonucleoprotein-associated protein N [OS=Mus musculus]	18	4	4
P63166	Small ubiquitin-related modifier 1 [OS=Mus musculus]	34	3	3
P63168	Dynein light chain 1, cytoplasmic [OS=Mus musculus]	64	5	2
P63213	Guanine nucleotide-binding protein G(I)/G(S)/G(O) subunit gamma-2 [OS=Mus musculus]	39	2	2
P63242	Eukaryotic translation initiation factor 5A-1 [OS=Mus musculus]	79	18	18
P63254	Cysteine-rich protein 1 [OS=Mus musculus]	68	3	3
P63271	Transcription elongation factor SPT4-A [OS=Mus musculus]	21	2	2
P63276	40S ribosomal protein S17 [OS=Mus musculus]	53	10	10
P63280	SUMO-conjugating enzyme ubc9 [OS=Mus musculus]	76	8	8
P63321	Ras-related protein Ral-A [OS=Mus musculus]	50	9	3
P63323	40S ribosomal protein S12 [OS=Mus musculus]	58	8	8
P63325	40S ribosomal protein S10 [OS=Mus musculus]	54	9	9
P63328	Serine/threonine-protein phosphatase 2B catalytic subunit alpha isoform [OS=Mus musculus]	19	7	3
P63330	Serine/threonine-protein phosphatase 2A catalytic subunit alpha isoform [OS=Mus musculus]	65	12	10

P67778	Prohibitin [OS=Mus musculus]	68	15	15
P67871	Casein kinase II subunit beta [OS=Mus musculus]	38	6	6
P67984	60S ribosomal protein L22 [OS=Mus musculus]	53	7	7
P68037	Ubiquitin-conjugating enzyme E2 L3 [OS=Mus musculus]	65	8	8
P68040	Receptor of activated protein C kinase 1 [OS=Mus musculus]	88	21	21
P68181-4	Isoform 4 of cAMP-dependent protein kinase catalytic subunit beta [OS=Mus musculus]	24	8	3
P68254-1	14-3-3 protein theta [OS=Mus musculus]	59	17	15
P68368	Tubulin alpha-4A chain [OS=Mus musculus]	66	22	7
P68369	tubulin alpha-1A chain [OS=Mus musculus]	80	28	3
P68373	Tubulin alpha-1C chain [OS=Mus musculus]	80	27	2
P68510	14-3-3 protein eta [OS=Mus musculus]	70	19	17
P69566	Ran-binding protein 9 [OS=Mus musculus]	9	3	2
P70122	Ribosome maturation protein SBDS [OS=Mus musculus]	30	6	6
P70158	acid sphingomyelinase-like phosphodiesterase 3a [OS=Mus musculus]	11	2	2
P70168	Importin subunit beta-1 [OS=Mus musculus]	71	39	39
P70182	Phosphatidylinositol 4-phosphate 5-kinase type-1 alpha [OS=Mus musculus]	14	4	3
P70195	Proteasome subunit beta type-7 [OS=Mus musculus]	51	10	10
P70248	Unconventional myosin-If [OS=Mus musculus]	14	12	6
P70268-2	Isoform 2 of Serine/threonine-protein kinase N1 [OS=Mus musculus]	7	5	5
P70280	vesicle-associated membrane protein 7 [OS=Mus musculus]	15	2	2
P70288	Histone deacetylase 2 [OS=Mus musculus]	35	9	6
P70290	55 kDa erythrocyte membrane protein [OS=Mus musculus]	30	8	8
P70295	Ancient ubiquitous protein 1 [OS=Mus musculus]	30	6	6
P70296	phosphatidylethanolamine-binding protein 1 [OS=Mus musculus]	87	11	11
P70302	stromal interaction molecule 1 [OS=Mus musculus]	8	4	4
P70315	Wiskott-Aldrich syndrome protein homolog [OS=Mus musculus]	30	8	8

P70318	Nucleolysin TIAR [OS=Mus musculus]	35	8	7
P70333	heterogeneous nuclear ribonucleoprotein H2 [OS=Mus musculus]	39	11	5
P70336-2	Isoform 2 of Rho-associated protein kinase 2 [OS=Mus musculus]	6	6	6
P70349	Histidine triad nucleotide-binding protein 1 [OS=Mus musculus]	72	8	8
P70362	Ubiquitin recognition factor in ER-associated degradation protein 1 [OS=Mus musculus]	30	8	8
P70372	ELAV-like protein 1 [OS=Mus musculus]	51	13	13
P70388	DNA repair protein Rad50 [OS=Mus musculus]	13	12	12
P70398	Probable ubiquitin carboxyl-terminal hydrolase FAF-X [OS=Mus musculus]	4	6	6
P70404	Isocitrate dehydrogenase [NAD] subunit gamma 1, mitochondrial [OS=Mus musculus]	39	10	10
P70429-1	Ena/VASP-like protein [OS=Mus musculus]	22	5	5
P70441-1	Na(+)/H(+) exchange regulatory cofactor NHE-RF1 [OS=Mus musculus]	21	5	5
P70444	BH3-interacting domain death agonist [OS=Mus musculus]	18	2	2
P70452	Syntaxin-4 [OS=Mus musculus]	35	6	6
P70460	Vasodilator-stimulated phosphoprotein [OS=Mus musculus]	61	16	16
P70665	sialate O-acetylesterase [OS=Mus musculus]	5	2	2
P70670	Nascent polypeptide-associated complex subunit alpha, muscle-specific form [OS=Mus musculus]	3	6	6
P70671	interferon regulatory factor 3 [OS=Mus musculus]	17	4	4
P70677	Caspase-3 [OS=Mus musculus]	43	8	8
P70697	Uroporphyrinogen decarboxylase [OS=Mus musculus]	26	6	6
P70698	CTP synthase 1 [OS=Mus musculus]	41	15	14
P70699	lysosomal alpha-glucosidase [OS=Mus musculus]	22	12	12
P80313	T-complex protein 1 subunit eta [OS=Mus musculus]	55	25	25
P80314	T-complex protein 1 subunit beta [OS=Mus musculus]	71	30	30
P80315	T-complex protein 1 subunit delta [OS=Mus musculus]	66	26	25
P80316	T-complex protein 1 subunit epsilon [OS=Mus musculus]	68	28	27

P80317	T-complex protein 1 subunit zeta [OS=Mus musculus]	60	20	20
P80318	T-complex protein 1 subunit gamma [OS=Mus musculus]	62	32	32
P81069	GA-binding protein subunit beta-2 [OS=Mus musculus]	10	2	2
P81117	Nucleobindin-2 [OS=Mus musculus]	31	9	9
P82343-1	N-acylglucosamine 2-epimerase [OS=Mus musculus]	8	2	2
P83870	PHD finger-like domain-containing protein 5A [OS=Mus musculus]	40	3	3
P83887	tubulin gamma-1 chain [OS=Mus musculus]	49	10	10
P83917	Chromobox protein homolog 1 [OS=Mus musculus]	40	4	3
P83940	elongin-C [OS=Mus musculus]	49	5	5
P84078	ADP-ribosylation factor 1 [OS=Mus musculus]	81	14	3
P84089	Enhancer of rudimentary homolog [OS=Mus musculus]	46	4	4
P84091	AP-2 complex subunit mu [OS=Mus musculus]	10	3	3
P84096	Rho-related GTP-binding protein RhoG [OS=Mus musculus]	54	10	9
P84099	60S ribosomal protein L19 [OS=Mus musculus]	9	2	2
P84104-1	Serine/arginine-rich splicing factor 3 [OS=Mus musculus]	29	4	3
P85094	isochorismatase domain-containing protein 2A [OS=Mus musculus]	54	5	5
P97287	Induced myeloid leukemia cell differentiation protein Mcl-1 homolog [OS=Mus musculus]	36	7	7
P97300	Neuroplastin [OS=Mus musculus]	18	5	5
P97304	DNA-directed RNA polymerases I and III subunit RPAC2 [OS=Mus musculus]	25	2	2
P97310	DNA replication licensing factor mcm2 [OS=Mus musculus]	46	28	28
P97311	DNA replication licensing factor MCM6 [OS=Mus musculus]	48	28	28
P97315	Cysteine and glycine-rich protein 1 [OS=Mus musculus]	46	5	5
P97329	kinesin-like protein KIF20A [OS=Mus musculus]	6	4	4
P97351	40S ribosomal protein S3a [OS=Mus musculus]	61	19	19
P97355	Spermine synthase [OS=Mus musculus]	48	10	10
P97360	Transcription factor ETV6 [OS=Mus musculus]	10	3	3
P97363	Serine palmitoyltransferase 2 [OS=Mus musculus]	30	11	11
P97364	Selenide, water dikinase 2 [OS=Mus musculus]	9	2	2

P97369	Neutrophil cytosol factor 4 [OS=Mus musculus]	48	15	15
P97370	sodium/potassium-transporting ATPase subunit beta-3 [OS=Mus musculus]	46	11	11
P97371	Proteasome activator complex subunit 1 [OS=Mus musculus]	57	13	13
P97372	proteasome activator complex subunit 2 [OS=Mus musculus]	66	11	11
P97376	Protein FRG1 [OS=Mus musculus]	23	4	4
P97377-1	cyclin-dependent kinase 2 [OS=Mus musculus]	48	11	9
P97379-1	Ras GTPase-activating protein-binding protein 2 [OS=Mus musculus]	32	10	9
P97384	annexin A11 [OS=Mus musculus]	24	10	9
P97386	DNA ligase 3 [OS=Mus musculus]	3	2	2
P97390	Vacuolar protein sorting-associated protein 45 [OS=Mus musculus]	11	5	5
P97429	annexin A4 [OS=Mus musculus]	50	14	14
P97449	aminopeptidase N [OS=Mus musculus]	7	5	5
P97450	ATP synthase-coupling factor 6, mitochondrial [OS=Mus musculus]	34	2	2
P97452	Ribosome biogenesis protein BOP1 [OS=Mus musculus]	7	3	3
P97461	40S ribosomal protein S5 [OS=Mus musculus]	45	8	8
P97477-2	Isoform 2 of Aurora kinase A [OS=Mus musculus]	10	3	3
P97480	eyes absent homolog 3 [OS=Mus musculus]	9	2	2
P97493	Thioredoxin, mitochondrial [OS=Mus musculus]	27	4	4
P97494	glutamate--cysteine ligase catalytic subunit [OS=Mus musculus]	42	19	19
P97496	SWI/SNF complex subunit SMARCC1 [OS=Mus musculus]	26	16	11
P97760	DNA-directed RNA polymerase II subunit RPB3 [OS=Mus musculus]	28	4	4
P97770-1	THUMP domain-containing protein 3 [OS=Mus musculus]	7	2	2
P97797-2	Isoform 2 of Tyrosine-protein phosphatase non-receptor type substrate 1 [OS=Mus musculus]	18	5	5
P97807-1	fumarate hydratase, mitochondrial [OS=Mus musculus]	58	20	20
P97808	FXYD domain-containing ion transport regulator 5 [OS=Mus musculus]	40	4	4
P97814	Proline-serine-threonine phosphatase-interacting protein 1 [OS=Mus musculus]	12	3	3
P97821	Dipeptidyl peptidase 1 [OS=Mus musculus]	18	7	7

P97822	Acidic leucine-rich nuclear phosphoprotein 32 family member E [OS=Mus musculus]	42	7	7
P97823-1	Acyl-protein thioesterase 1 [OS=Mus musculus]	41	5	5
P97825	Jupiter microtubule associated homolog 1 [OS=Mus musculus]	28	2	2
P97855	Ras GTPase-activating protein-binding protein 1 [OS=Mus musculus]	51	18	17
P97930	thymidylate kinase [OS=Mus musculus]	23	4	4
P98078	Disabled homolog 2 [OS=Mus musculus]	40	21	21
P98083-1	SHC-transforming protein 1 [OS=Mus musculus]	18	5	5
P99024	tubulin beta-5 chain [OS=Mus musculus]	81	29	5
P99026	Proteasome subunit beta type-4 [OS=Mus musculus]	47	7	7
P99027	60S acidic ribosomal protein P2 [OS=Mus musculus]	76	8	8
P99029	Peroxiredoxin-5, mitochondrial [OS=Mus musculus]	57	12	12
Q00422	GA-binding protein alpha chain [OS=Mus musculus]	16	6	6
Q00547-1	Hyaluronan mediated motility receptor [OS=Mus musculus]	8	2	2
Q00612	Glucose-6-phosphate 1-dehydrogenase X [OS=Mus musculus]	68	33	33
Q00623	Apolipoprotein A-I [OS=Mus musculus]	4	2	2
Q00651	Integrin alpha-4 [OS=Mus musculus]	17	12	12
Q00899	Transcriptional repressor protein YY1 [OS=Mus musculus]	12	3	3
Q00PI9	heterogeneous nuclear ribonucleoprotein U-like protein 2 [OS=Mus musculus]	10	5	5
Q01405	protein transport protein Sec23A [OS=Mus musculus]	7	4	2
Q01730	Ras suppressor protein 1 [OS=Mus musculus]	49	8	8
Q01768	nucleoside diphosphate kinase b [OS=Mus musculus]	74	11	6
Q01853	Transitional endoplasmic reticulum ATPase [OS=Mus musculus]	77	58	57
Q01965	T-lymphocyte surface antigen Ly-9 [OS=Mus musculus]	11	4	4
Q02053	Ubiquitin-like modifier-activating enzyme 1 [OS=Mus musculus]	73	47	47
Q02257	Junction plakoglobin [OS=Mus musculus]	8	4	4
Q02566	myosin-6 [OS=Mus musculus]	4	6	6
Q02819	Nucleobindin-1 [OS=Mus musculus]	53	18	18
Q03147	Cyclin-dependent kinase 7 [OS=Mus musculus]	15	3	3

Q03265	ATP synthase subunit alpha, mitochondrial [OS=Mus musculus]	61	28	28
Q03958	Prefoldin subunit 6 [OS=Mus musculus]	31	2	2
Q03963	interferon-induced, double-stranded RNA-activated protein kinase [OS=Mus musculus]	23	7	7
Q04207-1	Transcription factor p65 [OS=Mus musculus]	6	2	2
Q04447	Creatine kinase B-type [OS=Mus musculus]	65	18	18
Q04646-1	sodium/potassium-transporting ATPase subunit gamma [OS=Mus musculus]	43	2	2
Q04692-2	Isoform 2 of SWI/SNF-related matrix-associated actin-dependent regulator of chromatin subfamily A containing DEAD/H box 1 [OS=Mus musculus]	6	3	3
Q04750	DNA topoisomerase 1 [OS=Mus musculus]	17	8	8
Q04899	Cyclin-dependent kinase 18 [OS=Mus musculus]	14	5	4
Q05144	Ras-related C3 botulinum toxin substrate 2 [OS=Mus musculus]	54	9	5
Q05186	Reticulocalbin-1 [OS=Mus musculus]	11	3	3
Q05512	Serine/threonine-protein kinase MARK2 [OS=Mus musculus]	14	6	6
Q05769	Prostaglandin G/H synthase 2 [OS=Mus musculus]	66	31	30
Q05816	Fatty acid-binding protein, epidermal [OS=Mus musculus]	77	11	11
Q05910	Disintegrin and metalloproteinase domain-containing protein 8 [OS=Mus musculus]	29	11	11
Q05915	GTP cyclohydrolase 1 [OS=Mus musculus]	35	5	5
Q05CL8	La-related protein 7 [OS=Mus musculus]	21	7	7
Q05D44	Eukaryotic translation initiation factor 5B [OS=Mus musculus]	22	15	15
Q06138	Calcium-binding protein 39 [OS=Mus musculus]	30	9	9
Q06185	ATP synthase subunit e, mitochondrial [OS=Mus musculus]	55	4	4
Q07076	Annexin A7 [OS=Mus musculus]	17	7	7
Q07417	Short-chain specific acyl-CoA dehydrogenase, mitochondrial [OS=Mus musculus]	44	12	12
Q07797	Galectin-3-binding protein [OS=Mus musculus]	43	17	17
Q07813-1	Apoptosis regulator BAX [OS=Mus musculus]	60	10	10
Q08024-1	Core-binding factor subunit beta [OS=Mus musculus]	29	3	3
Q08288	Cell growth-regulating nucleolar protein [OS=Mus musculus]	41	10	10

Q08509	Epidermal growth factor receptor kinase substrate 8 [OS=Mus musculus]	8	4	4
Q08639	Transcription factor Dp-1 [OS=Mus musculus]	9	2	2
Q08857	Platelet glycoprotein 4 [OS=Mus musculus]	39	14	14
Q08943-2	Isoform 2 of FACT complex subunit SSRP1 [OS=Mus musculus]	3	2	2
Q09014	Neutrophil cytosol factor 1 [OS=Mus musculus]	46	13	13
Q09200	Beta-1,4 N-acetylgalactosaminyltransferase 1 [OS=Mus musculus]	29	11	11
Q0GNC1	Inverted formin-2 [OS=Mus musculus]	38	27	27
Q0P678	Zinc finger CCCH domain-containing protein 18 [OS=Mus musculus]	4	2	2
Q0VGB7	Serine/threonine-protein phosphatase 4 regulatory subunit 2 [OS=Mus musculus]	32	10	10
Q11011	puromycin-sensitive aminopeptidase [OS=Mus musculus]	24	15	15
Q149F1	RNA pseudouridylate synthase domain-containing protein 2 [OS=Mus musculus]	8	3	3
Q14C51	pentatricopeptide repeat domain-containing protein 3, mitochondrial [OS=Mus musculus]	26	15	15
Q14CH7	alanine--tRNA ligase, mitochondrial [OS=Mus musculus]	3	3	3
Q1HFZ0-1	tRNA (Cytosine(34)-C(5))-methyltransferase [OS=Mus musculus]	56	29	29
Q2EMV9	poly [ADP-ribose] polymerase 14 [OS=Mus musculus]	3	3	3
Q2NL51	Glycogen synthase kinase-3 alpha [OS=Mus musculus]	13	3	3
Q2TBE6	Phosphatidylinositol 4-kinase type 2-alpha [OS=Mus musculus]	19	6	6
Q2YDW2-1	Protein misato homolog 1 [OS=Mus musculus]	24	9	9
Q31125	Zinc transporter SLC39A7 [OS=Mus musculus]	19	4	4
Q3B7Z2	Oxysterol-binding protein 1 [OS=Mus musculus]	6	3	3
Q3TB92	Allergin-1 [OS=Mus musculus]	14	2	2
Q3TBD2-1	Rho GTPase-activating protein 45 [OS=Mus musculus]	20	14	14
Q3TBT3-2	Isoform 2 of Stimulator of interferon genes protein [OS=Mus musculus]	7	2	2
Q3TBW2	39S ribosomal protein L10, mitochondrial [OS=Mus musculus]	36	5	5
Q3TCH7	cullin-4A [OS=Mus musculus]	12	8	2
Q3TCJ1	BRISC complex subunit Abraxas 2 [OS=Mus musculus]	10	3	3
Q3TCN2	Putative phospholipase B-like 2 [OS=Mus musculus]	21	9	9
Q3TCX3	Protein BLOM7 [OS=Mus musculus]	8	3	3
Q3TDD9-1	Protein phosphatase 1 regulatory subunit 21 [OS=Mus musculus]	6	3	3

Q3TDN2-1	FAS-associated factor 2 [OS=Mus musculus]	51	14	14
Q3TEA8-1	Heterochromatin protein 1-binding protein 3 [OS=Mus musculus]	26	10	10
Q3TFK5	G patch domain-containing protein 4 [OS=Mus musculus]	7	2	2
Q3TFQ1	SPRY domain-containing protein 7 [OS=Mus musculus]	22	3	3
Q3THE2	Myosin regulatory light chain 12B [OS=Mus musculus]	51	9	9
Q3THG9	Alanyl-tRNA editing protein Aarsd1 [OS=Mus musculus]	31	7	7
Q3THK3	General transcription factor iif subunit 1 [OS=Mus musculus]	20	7	7
Q3THK7	GMP synthase [glutamine-hydrolyzing] [OS=Mus musculus]	39	20	20
Q3THS6	S-adenosylmethionine synthase isoform type-2 [OS=Mus musculus]	29	10	10
Q3TIR3	Synembryn-A [OS=Mus musculus]	10	4	4
Q3TIU4	2',5'-phosphodiesterase 12 [OS=Mus musculus]	21	7	7
Q3TIV5-1	Zinc finger CCCH domain-containing protein 15 [OS=Mus musculus]	24	7	7
Q3TIX9	U4/U6.U5 tri-snRNP-associated protein 2 [OS=Mus musculus]	15	4	4
Q3TJZ6	protein FAM98A [OS=Mus musculus]	26	9	7
Q3TKT4-2	Isoform 2 of Transcription activator BRG1 [OS=Mus musculus]	9	10	10
Q3TKY6	Peptidyl-prolyl cis-trans isomerase CWC27 homolog [OS=Mus musculus]	15	3	3
Q3TL44	NLR family member X1 [OS=Mus musculus]	6	3	3
Q3TLD5	Unconventional prefoldin RPB5 interactor [OS=Mus musculus]	14	4	4
Q3TLH4	Protein Prrc2c [OS=Mus musculus]	12	18	18
Q3TMX7	Sulfhydryl oxidase 2 [OS=Mus musculus]	11	4	4
Q3TN34	MICAL-like protein 2 [OS=Mus musculus]	7	4	4
Q3TPX4	Exocyst complex component 5 [OS=Mus musculus]	5	2	2
Q3TRM4	Neuropathy target esterase [OS=Mus musculus]	2	2	2
Q3TRM8	Hexokinase-3 [OS=Mus musculus]	29	18	16
Q3TSG4	RNA demethylase ALKBH5 [OS=Mus musculus]	10	2	2
Q3TTY5	Keratin, type II cytoskeletal 2 epidermal [OS=Mus musculus]	5	4	3
Q3TUH1	Phosphatidate cytidylyltransferase, mitochondrial [OS=Mus musculus]	18	3	3
Q3TVI8	Pre-B-cell leukemia transcription factor-interacting protein 1 [OS=Mus musculus]	2	2	2
Q3TW96-1	UDP-N-acetylhexosamine pyrophosphorylase-like protein 1 [OS=Mus musculus]	35	14	12
Q3TWW8	Serine/arginine-rich splicing factor 6 [OS=Mus musculus]	11	4	3

Q3TX08	tRNA (guanine(26)-N(2))-dimethyltransferase [OS=Mus musculus]	7	3	3
Q3TXS7	26S proteasome non-ATPase regulatory subunit 1 [OS=Mus musculus]	60	37	37
Q3TXU5	deoxyhypusine synthase [OS=Mus musculus]	11	3	3
Q3TYS2	Uncharacterized protein C17orf62 homolog [OS=Mus musculus]	12	2	2
Q3TYX3	SET and MYND domain-containing protein 5 [OS=Mus musculus]	10	3	3
Q3U0K8-1	prolyl 3-hydroxylase OGFOD1 [OS=Mus musculus]	15	3	3
Q3U0V1	Far upstream element-binding protein 2 [OS=Mus musculus]	45	24	23
Q3U0V2	Tumor necrosis factor receptor type 1-associated DEATH domain protein [OS=Mus musculus]	30	6	6
Q3U186	probable arginine--tRNA ligase, mitochondrial [OS=Mus musculus]	17	8	8
Q3U1V6	Ubiquitin-conjugating enzyme E2 variant 3 [OS=Mus musculus]	11	3	3
Q3U1Y4-1	DENN domain-containing protein 4B [OS=Mus musculus]	2	2	2
Q3U1Z5-1	G-protein-signaling modulator 3 [OS=Mus musculus]	23	2	2
Q3U2C5-1	E3 ubiquitin-protein ligase RNF149 [OS=Mus musculus]	7	2	2
Q3U308	Cytoplasmic tRNA 2-thiolation protein 2 [OS=Mus musculus]	5	2	2
Q3U319	E3 ubiquitin-protein ligase BRE1B [OS=Mus musculus]	17	11	9
Q3U4G3	Xyloside xylosyltransferase 1 [OS=Mus musculus]	13	4	4
Q3U5Q7	UMP-CMP kinase 2, mitochondrial [OS=Mus musculus]	52	14	14
Q3U7R1	Extended synaptotagmin-1 [OS=Mus musculus]	54	41	41
Q3UA06-1	Pachytene checkpoint protein 2 homolog [OS=Mus musculus]	28	8	8
Q3UA16-1	Kinetochore protein Spc25 [OS=Mus musculus]	18	3	3
Q3UA37	Glutamine-rich protein 1 [OS=Mus musculus]	10	4	4
Q3UBG2	PTB-containing, cubilin and LRP1-interacting protein [OS=Mus musculus]	29	3	3
Q3UBX0	Transmembrane protein 109 [OS=Mus musculus]	17	4	4
Q3UCV8	Ubiquitin thioesterase otulin [OS=Mus musculus]	4	2	2
Q3UDE2	Tubulin--tyrosine ligase-like protein 12 [OS=Mus musculus]	30	13	13
Q3UDK1-1	TRAF-type zinc finger domain-containing protein 1 [OS=Mus musculus]	12	5	5
Q3UEB3-1	poly(U)-binding-splicing factor PUF60 [OS=Mus musculus]	30	11	11
Q3UFM5	nucleolar MIF4G domain-containing protein 1 [OS=Mus musculus]	4	2	2
Q3UFY8	Mitochondrial ribonuclease P protein 1 [OS=Mus musculus]	15	4	4
Q3UGC7	eukaryotic translation initiation factor 3 subunit J-A [OS=Mus musculus]	43	8	8

Q3UGP9	Leucine-rich repeat-containing protein 58 [OS=Mus musculus]	12	3	3
Q3UGR5-1	haloacid dehalogenase-like hydrolase domain-containing protein 2 [OS=Mus musculus]	42	6	6
Q3UHH8-1	Glucoside xylosyltransferase 1 [OS=Mus musculus]	6	2	2
Q3UHX2	28 kDa heat- and acid-stable phosphoprotein [OS=Mus musculus]	16	2	2
Q3UIA2	Rho GTPase-activating protein 17 [OS=Mus musculus]	16	8	8
Q3UIR3-1	E3 ubiquitin-protein ligase DTX3L [OS=Mus musculus]	9	4	4
Q3UIU2	NADH dehydrogenase [ubiquinone] 1 beta subcomplex subunit 6 [OS=Mus musculus]	35	4	4
Q3UJB9	Enhancer of mRNA-decapping protein 4 [OS=Mus musculus]	16	12	12
Q3UJD6	Ubiquitin carboxyl-terminal hydrolase 19 [OS=Mus musculus]	12	8	8
Q3UJU9	Regulator of microtubule dynamics protein 3 [OS=Mus musculus]	20	6	6
Q3ULD5	Methylcrotonoyl-CoA carboxylase beta chain, mitochondrial [OS=Mus musculus]	15	5	5
Q3ULF4	Paraplegin [OS=Mus musculus]	13	5	5
Q3ULJ0-1	Glycerol-3-phosphate dehydrogenase 1-like protein [OS=Mus musculus]	42	10	10
Q3UM18	Large subunit GTPase 1 homolog [OS=Mus musculus]	9	3	3
Q3UM29	Conserved oligomeric Golgi complex subunit 7 [OS=Mus musculus]	5	2	2
Q3UM45	Protein phosphatase 1 regulatory subunit 7 [OS=Mus musculus]	48	12	12
Q3UMC0	Spermatogenesis-associated protein 5 [OS=Mus musculus]	5	3	2
Q3UMQ8	H/ACA ribonucleoprotein complex non-core subunit NAF1 [OS=Mus musculus]	18	5	5
Q3UMU9-3	Isoform 3 of Hepatoma-derived growth factor-related protein 2 [OS=Mus musculus]	3	2	2
Q3UND0-1	src kinase-associated phosphoprotein 2 [OS=Mus musculus]	53	13	13
Q3UPF5-1	zinc finger CCCH-type antiviral protein 1 [OS=Mus musculus]	10	6	6
Q3UPH1	protein PRRC1 [OS=Mus musculus]	38	8	8
Q3UPL0	Protein transport protein Sec31A [OS=Mus musculus]	28	21	21
Q3UQ84	Threonine--tRNA ligase, mitochondrial [OS=Mus musculus]	18	9	9
Q3UQN2	F-BAR domain only protein 2 [OS=Mus musculus]	3	2	2
Q3URQ0	Testis-expressed protein 10 [OS=Mus musculus]	5	4	4
Q3URS9	Coiled-coil domain-containing protein 51 [OS=Mus musculus]	12	4	4
Q3UU41	SLP adapter and CSK-interacting membrane protein [OS=Mus musculus]	29	2	2
Q3UVG3	Protein FAM91A1 [OS=Mus musculus]	3	2	2

Q3UVL4-1	Vacuolar protein sorting-associated protein 51 homolog [OS=Mus musculus]	16	7	7
Q3UW53	Protein Niban [OS=Mus musculus]	31	21	21
Q3UY34	uncharacterized protein C12orf43 homolog [OS=Mus musculus]	18	2	2
Q3UYV9	Nuclear cap-binding protein subunit 1 [OS=Mus musculus]	39	21	21
Q3UZ39-1	Leucine-rich repeat flightless-interacting protein 1 [OS=Mus musculus]	27	11	11
Q3UZA1	CapZ-interacting protein [OS=Mus musculus]	11	3	3
Q3V009	Transmembrane emp24 domain-containing protein 1 [OS=Mus musculus]	11	2	2
Q3V1L4	Cytosolic purine 5'-nucleotidase [OS=Mus musculus]	19	6	6
Q3V1V3	ESF1 homolog [OS=Mus musculus]	3	2	2
Q3V300	Kinesin-like protein KIF22 [OS=Mus musculus]	8	4	4
Q3V384	AFG1-like ATPase [OS=Mus musculus]	7	2	2
Q3V3R1	Monofunctional C1-tetrahydrofolate synthase, mitochondrial [OS=Mus musculus]	60	40	40
Q4FZC9-1	Nesprin-3 [OS=Mus musculus]	21	10	10
Q4KML4	Costars family protein ABRACL [OS=Mus musculus]	31	2	2
Q4PJX1-1	Protein odr-4 homolog [OS=Mus musculus]	6	2	2
Q4QRL3	Coiled-coil domain-containing protein 88B [OS=Mus musculus]	10	10	10
Q4VA53-3	Isoform 3 of Sister chromatid cohesion protein PDS5 homolog B [OS=Mus musculus]	21	20	16
Q4VAA2-1	Protein CDV3 [OS=Mus musculus]	36	4	4
Q501J2	Protein FAM173A [OS=Mus musculus]	30	3	3
Q501J6	Probable ATP-dependent RNA helicase DDX17 [OS=Mus musculus]	43	23	17
Q501J7-3	Isoform 3 of Phosphatase and actin regulator 4 [OS=Mus musculus]	5	2	2
Q504P2	C-type lectin domain family 12 member A [OS=Mus musculus]	7	2	2
Q505B7	Protein archease [OS=Mus musculus]	16	2	2
Q505D7	optic atrophy 3 protein homolog [OS=Mus musculus]	28	3	3
Q505F5	Leucine-rich repeat-containing protein 47 [OS=Mus musculus]	40	15	15
Q52KI8-1	Serine/arginine repetitive matrix protein 1 [OS=Mus musculus]	8	4	4
Q569Z6	Thyroid hormone receptor-associated protein 3 [OS=Mus musculus]	12	7	7
Q56A08	G patch domain and KOW motifs-containing protein [OS=Mus musculus]	20	5	5
Q571E4	N-acetylgalactosamine-6-sulfatase [OS=Mus musculus]	5	2	2

Q571I9	Aldehyde dehydrogenase family 16 member A1 [OS=Mus musculus]	18	7	7
Q59J78	NADH dehydrogenase [ubiquinone] 1 alpha subcomplex assembly factor 2 [OS=Mus musculus]	43	5	5
Q5DTM8	E3 ubiquitin-protein ligase BRE1A [OS=Mus musculus]	20	13	11
Q5EG47	5'-AMP-activated protein kinase catalytic subunit alpha-1 [OS=Mus musculus]	11	4	4
Q5F2E7-2	Isoform 2 of Nuclear fragile X mental retardation-interacting protein 2 [OS=Mus musculus]	17	6	6
Q5FWK3	rho GTPase-activating protein 1 [OS=Mus musculus]	59	14	14
Q5NBU8	XIAP-associated factor 1 [OS=Mus musculus]	27	4	4
Q5NC05	transcription termination factor 2 [OS=Mus musculus]	7	5	5
Q5PRF0-1	HEAT repeat-containing protein 5A [OS=Mus musculus]	2	2	2
Q5PSV9	Mediator of DNA damage checkpoint protein 1 [OS=Mus musculus]	2	2	2
Q5SFM8	RNA-binding protein 27 [OS=Mus musculus]	14	8	7
Q5SS80-1	Dehydrogenase/reductase SDR family member 13 [OS=Mus musculus]	8	2	2
Q5SSI6	U3 small nucleolar RNA-associated protein 18 homolog [OS=Mus musculus]	7	2	2
Q5SSK3	Transcription elongation factor, mitochondrial [OS=Mus musculus]	13	3	3
Q5SSW2-1	Proteasome activator complex subunit 4 [OS=Mus musculus]	1	2	2
Q5SSZ5-1	Tensin-3 [OS=Mus musculus]	6	4	4
Q5SUA5	unconventional myosin-Ig [OS=Mus musculus]	16	11	10
Q5SUC9	Protein SCO1 homolog, mitochondrial [OS=Mus musculus]	20	3	3
Q5SUF2-2	Isoform 2 of Luc7-like protein 3 [OS=Mus musculus]	12	4	4
Q5SUR0	Phosphoribosylformylglycinamidine synthase [OS=Mus musculus]	51	38	38
Q5SW19-1	Clustered mitochondria protein homolog [OS=Mus musculus]	6	5	5
Q5SWD9-1	Pre-rRNA-processing protein TSR1 homolog [OS=Mus musculus]	16	9	9
Q5SX39	Myosin-4 [OS=Mus musculus]	2	2	2
Q5SXY1-1	Cytospin-B [OS=Mus musculus]	11	8	2
Q5SYD0	Unconventional myosin-Id [OS=Mus musculus]	6	4	3
Q5SYH2	Transmembrane protein 199 [OS=Mus musculus]	31	4	4
Q5U3K5	Rab-like protein 6 [OS=Mus musculus]	4	2	2
Q5U458	DnaJ homolog subfamily C member 11 [OS=Mus musculus]	18	6	6
Q5U4D9	THO complex subunit 6 homolog [OS=Mus musculus]	10	2	2
Q5XG73	Acyl-CoA-binding domain-containing protein 5 [OS=Mus musculus]	9	3	3
Q5XJY5	Coatomer subunit delta [OS=Mus musculus]	34	13	13

Q60520-1	Isoform 2 of Paired amphipathic helix protein Sin3a [OS=Mus musculus]	11	9	9
Q60597	2-oxoglutarate dehydrogenase, mitochondrial [OS=Mus musculus]	48	39	39
Q60605-2	Isoform Smooth muscle of Myosin light polypeptide 6 [OS=Mus musculus]	84	11	10
Q60631	Growth factor receptor-bound protein 2 [OS=Mus musculus]	39	6	6
Q60648	Ganglioside GM2 activator [OS=Mus musculus]	24	3	3
Q60649	Caseinolytic peptidase B protein homolog [OS=Mus musculus]	17	6	6
Q60664	Lymphoid-restricted membrane protein [OS=Mus musculus]	14	6	6
Q60668-1	heterogeneous nuclear ribonucleoprotein D0 [OS=Mus musculus]	25	10	8
Q60676	Serine/threonine-protein phosphatase 5 [OS=Mus musculus]	18	6	6
Q60692	Proteasome subunit beta type-6 [OS=Mus musculus]	41	6	6
Q60710	deoxynucleoside triphosphate triphosphohydrolase SAMHD1 [OS=Mus musculus]	55	23	23
Q60737	Casein kinase II subunit alpha [OS=Mus musculus]	46	13	12
Q60749	KH domain-containing, RNA-binding, signal transduction-associated protein 1 [OS=Mus musculus]	20	6	6
Q60759	Glutaryl-CoA dehydrogenase, mitochondrial [OS=Mus musculus]	9	3	3
Q60766	Immunity-related GTPase family M protein 1 [OS=Mus musculus]	55	17	17
Q60770	syntaxin-binding protein 3 [OS=Mus musculus]	24	9	9
Q60780-1	Growth arrest-specific protein 7 [OS=Mus musculus]	15	4	4
Q60787	lymphocyte cytosolic protein 2 [OS=Mus musculus]	46	13	13
Q60790	Ras GTPase-activating protein 3 [OS=Mus musculus]	8	4	4
Q60809	CCR4-NOT transcription complex subunit 7 [OS=Mus musculus]	8	2	2
Q60848-1	Lymphocyte-specific helicase [OS=Mus musculus]	23	12	12
Q60854	serpin B6 [OS=Mus musculus]	40	11	11
Q60855	Receptor-interacting serine/threonine-protein kinase 1 [OS=Mus musculus]	4	2	2
Q60862	Origin recognition complex subunit 2 [OS=Mus musculus]	9	3	3
Q60864	stress-induced-phosphoprotein 1 [OS=Mus musculus]	47	30	30
Q60865	Caprin-1 [OS=Mus musculus]	26	10	10
Q60866	Phosphotriesterase-related protein [OS=Mus musculus]	14	3	3

Q60870	Receptor expression-enhancing protein 5 [OS=Mus musculus]	13	3	3
Q60875	Rho guanine nucleotide exchange factor 2 [OS=Mus musculus]	32	21	21
Q60902-1	Epidermal growth factor receptor substrate 15-like 1 [OS=Mus musculus]	10	7	7
Q60930	Voltage-dependent anion-selective channel protein 2 [OS=Mus musculus]	55	12	12
Q60931	Voltage-dependent anion-selective channel protein 3 [OS=Mus musculus]	54	12	11
Q60932-2	Isoform Mt-VDAC1 of Voltage-dependent anion-selective channel protein 1 [OS=Mus musculus]	81	19	18
Q60953-1	Protein PML [OS=Mus musculus]	11	6	6
Q60960	Importin subunit alpha-5 [OS=Mus musculus]	41	13	8
Q60967	bifunctional 3'-phosphoadenosine 5'-phosphosulfate synthase 1 [OS=Mus musculus]	19	8	8
Q60972	Histone-binding protein RBBP4 [OS=Mus musculus]	76	17	12
Q60973	Histone-binding protein RBBP7 [OS=Mus musculus]	76	16	11
Q60996-1	Serine/threonine-protein phosphatase 2A 56 kDa regulatory subunit gamma isoform [OS=Mus musculus]	18	7	6
Q61024	Asparagine synthetase [glutamine-hydrolyzing] [OS=Mus musculus]	21	8	8
Q61029	Lamina-associated polypeptide 2, isoforms beta/delta/epsilon/gamma [OS=Mus musculus]	48	15	7
Q61033-1	Lamina-associated polypeptide 2, isoforms alpha/zeta [OS=Mus musculus]	42	19	11
Q61035	Histidine--tRNA ligase, cytoplasmic [OS=Mus musculus]	49	18	16
Q61074	Protein phosphatase 1G [OS=Mus musculus]	34	12	12
Q61081	Hsp90 co-chaperone Cdc37 [OS=Mus musculus]	38	13	13
Q61093	Cytochrome b-245 heavy chain [OS=Mus musculus]	16	5	5
Q61103	Zinc finger protein ubi-d4 [OS=Mus musculus]	19	5	5
Q61112-1	45 kDa calcium-binding protein [OS=Mus musculus]	14	3	3
Q61127	NGFI-A-binding protein 2 [OS=Mus musculus]	10	3	3
Q61160	FAS-associated death domain protein [OS=Mus musculus]	18	2	2
Q61164	Transcriptional repressor CTCF [OS=Mus musculus]	9	3	3
Q61166	Microtubule-associated protein RP/EB family member 1 [OS=Mus musculus]	75	14	14

Q61171	Peroxiredoxin-2 [OS=Mus musculus]	85	13	13
Q61179	Interferon regulatory factor 9 [OS=Mus musculus]	8	2	2
Q61187	tumor susceptibility gene 101 protein [OS=Mus musculus]	17	5	5
Q61188-1	Histone-lysine N-methyltransferase EZH2 [OS=Mus musculus]	4	2	2
Q61189	Methylosome subunit pICln [OS=Mus musculus]	23	3	3
Q61191	Host cell factor 1 [OS=Mus musculus]	12	14	14
Q61205	Platelet-activating factor acetylhydrolase IB subunit gamma [OS=Mus musculus]	24	3	3
Q61206	platelet-activating factor acetylhydrolase IB subunit beta [OS=Mus musculus]	58	6	6
Q61207	Prosaposin [OS=Mus musculus]	21	9	9
Q61210-5	Isoform 5 of Rho guanine nucleotide exchange factor 1 [OS=Mus musculus]	37	26	26
Q61211-1	Eukaryotic translation initiation factor 2d [OS=Mus musculus]	16	5	5
Q61216-1	Double-strand break repair protein MRE11 [OS=Mus musculus]	16	8	8
Q61233	Plastin-2 [OS=Mus musculus]	84	53	48
Q61239	protein farnesyltransferase/geranylgeranyltransferase type-1 subunit alpha [OS=Mus musculus]	24	7	7
Q61249	Immunoglobulin-binding protein 1 [OS=Mus musculus]	30	7	7
Q61316	Heat shock 70 kDa protein 4 [OS=Mus musculus]	66	43	41
Q61333	Tumor necrosis factor alpha-induced protein 2 [OS=Mus musculus]	4	2	2
Q61334	B-cell receptor-associated protein 29 [OS=Mus musculus]	22	5	5
Q61335	B-cell receptor-associated protein 31 [OS=Mus musculus]	31	7	7
Q61425	Hydroxyacyl-coenzyme A dehydrogenase, mitochondrial [OS=Mus musculus]	46	8	8
Q61458	Cyclin-H [OS=Mus musculus]	22	4	4
Q61462	Cytochrome b-245 light chain [OS=Mus musculus]	22	2	2
Q61464	Zinc finger protein 638 [OS=Mus musculus]	5	5	5
Q61490	CD166 antigen [OS=Mus musculus]	38	16	16
Q61510	E3 ubiquitin/ISG15 ligase TRIM25 [OS=Mus musculus]	6	3	3
Q61543	Golgi apparatus protein 1 [OS=Mus musculus]	43	36	36
Q61545	RNA-binding protein EWS [OS=Mus musculus]	14	7	7
Q61550	Double-strand-break repair protein rad21 homolog [OS=Mus musculus]	22	8	8

Q61578	NADPH:adrenodoxin oxidoreductase, mitochondrial [OS=Mus musculus]	29	9	9
Q61584	Fragile X mental retardation syndrome-related protein 1 [OS=Mus musculus]	24	9	8
Q61595	Kinectin [OS=Mus musculus]	21	17	17
Q61598	Rab GDP dissociation inhibitor beta [OS=Mus musculus]	83	39	33
Q61599	Rho GDP-dissociation inhibitor 2 [OS=Mus musculus]	72	14	14
Q61655	ATP-dependent RNA helicase DDX19A [OS=Mus musculus]	5	2	2
Q61656	probable ATP-dependent RNA helicase DDX5 [OS=Mus musculus]	55	26	20
Q61699	Heat shock protein 105 kDa [OS=Mus musculus]	56	38	37
Q61712	DnaJ homolog subfamily C member 1 [OS=Mus musculus]	8	4	4
Q61733	28S ribosomal protein S31, mitochondrial [OS=Mus musculus]	26	8	7
Q61735-2	Isoform 2 of Leukocyte surface antigen CD47 [OS=Mus musculus]	8	2	2
Q61749-2	Isoform 2 of Translation initiation factor eIF-2B subunit delta [OS=Mus musculus]	31	10	10
Q61753	D-3-phosphoglycerate dehydrogenase [OS=Mus musculus]	52	22	22
Q61768	Kinesin-1 heavy chain [OS=Mus musculus]	43	30	30
Q61781	Keratin, type I cytoskeletal 14 [OS=Mus musculus]	27	12	3
Q61792	LIM and SH3 domain protein 1 [OS=Mus musculus]	16	4	4
Q61881	DNA replication licensing factor MCM7 [OS=Mus musculus]	62	33	33
Q61937	Nucleophosmin [OS=Mus musculus]	50	12	12
Q62084	Protein phosphatase 1 regulatory subunit 14B [OS=Mus musculus]	59	4	4
Q62086	Serum paraoxonase/arylesterase 2 [OS=Mus musculus]	57	10	10
Q62087	Serum paraoxonase/lactonase 3 [OS=Mus musculus]	42	8	8
Q62093	serine/arginine-rich splicing factor 2 [OS=Mus musculus]	19	3	3
Q62095	atp-dependent rna helicase ddx3y [OS=Mus musculus]	35	21	3
Q62159	Rho-related GTP-binding protein RhoC [OS=Mus musculus]	69	11	2

Q62167	ATP-dependent RNA helicase DDX3X [OS=Mus musculus]	48	26	8
Q62186	translocon-associated protein subunit delta [OS=Mus musculus]	33	6	6
Q62189	U1 SMALL NUCLEAR RIBONUCLEOPROTEIN A [OS=Mus musculus]	30	5	4
Q62192	CD180 antigen [OS=Mus musculus]	30	12	12
Q62193	Replication protein A 32 kDa subunit [OS=Mus musculus]	34	5	5
Q62203	splicing factor 3a subunit 2 [OS=Mus musculus]	11	3	3
Q62230	sialoadhesin [OS=Mus musculus]	15	14	14
Q62241	U1 small nuclear ribonucleoprotein C [OS=Mus musculus]	19	2	2
Q62261-1	Spectrin beta chain, non-erythrocytic 1 [OS=Mus musculus]	20	31	31
Q62318	Transcription intermediary factor 1-beta [OS=Mus musculus]	66	27	27
Q62348	Translin [OS=Mus musculus]	58	8	8
Q62351	Transferrin receptor protein 1 [OS=Mus musculus]	33	21	21
Q62376-1	U1 small nuclear ribonucleoprotein 70 kDa [OS=Mus musculus]	24	8	8
Q62383	transcription elongation factor spt6 [OS=Mus musculus]	8	8	8
Q62384	Zinc finger protein ZPR1 [OS=Mus musculus]	20	7	7
Q62393-1	Tumor protein D52 [OS=Mus musculus]	44	9	9
Q62418	Drebrin-like protein [OS=Mus musculus]	32	11	11
Q62419	Endophilin-A2 [OS=Mus musculus]	25	5	5
Q62422	osteoclast-stimulating factor 1 [OS=Mus musculus]	54	9	9
Q62425	Cytochrome c oxidase subunit NDUFA4 [OS=Mus musculus]	28	3	3
Q62426	Cystatin-B [OS=Mus musculus]	87	6	6
Q62431	AT-rich interactive domain-containing protein 3A [OS=Mus musculus]	5	2	2
Q62432	Mothers against decapentaplegic homolog 2 [OS=Mus musculus]	13	4	4
Q62433	Protein NDRG1 [OS=Mus musculus]	44	10	10
Q62446	peptidyl-prolyl cis-trans isomerase FKBP3 [OS=Mus musculus]	50	10	10
Q62448	Eukaryotic translation initiation factor 4 gamma 2 [OS=Mus musculus]	52	33	33
Q62465	Synaptic vesicle membrane protein VAT-1 homolog [OS=Mus musculus]	49	13	13
Q62523	Zyxin [OS=Mus musculus]	9	3	3
Q63829	COMM domain-containing protein 3 [OS=Mus musculus]	37	5	5

Q63844	mitogen-activated protein kinase 3 [OS=Mus musculus]	58	13	8
Q63850	nuclear pore glycoprotein p62 [OS=Mus musculus]	29	12	12
Q63932	Dual specificity mitogen-activated protein kinase kinase 2 [OS=Mus musculus]	28	6	4
Q63943	Myocyte-specific enhancer factor 2D [OS=Mus musculus]	5	2	2
Q64010-1	Adapter molecule crk [OS=Mus musculus]	14	3	3
Q64012	RNA-binding protein Raly [OS=Mus musculus]	14	3	3
Q640M1	U3 small nucleolar RNA-associated protein 14 homolog A [OS=Mus musculus]	14	7	7
Q640N3-1	Rho GTPase-activating protein 30 [OS=Mus musculus]	3	2	2
Q64105	Sepiapterin reductase [OS=Mus musculus]	47	8	8
Q64112	Interferon-induced protein with tetratricopeptide repeats 2 [OS=Mus musculus]	24	9	9
Q64152-1	transcription factor BTF3 [OS=Mus musculus]	49	7	7
Q64213	Splicing factor 1 [OS=Mus musculus]	26	13	13
Q64261	Cyclin-dependent kinase 6 [OS=Mus musculus]	22	5	4
Q64277	ADP-ribosyl cyclase/cyclic ADP-ribose hydrolase 2 [OS=Mus musculus]	19	5	5
Q64281	Leukocyte immunoglobulin-like receptor subfamily B member 4 [OS=Mus musculus]	29	5	5
Q64282	interferon-induced protein with tetratricopeptide repeats 1 [OS=Mus musculus]	23	7	7
Q64324	Syntaxin-binding protein 2 [OS=Mus musculus]	55	21	21
Q64337	sequestosome-1 [OS=Mus musculus]	85	21	21
Q64339	Ubiquitin-like protein ISG15 [OS=Mus musculus]	79	7	7
Q64345	Interferon-induced protein with tetratricopeptide repeats 3 [OS=Mus musculus]	27	8	8
Q64373	bcl-2-like protein 1 [OS=Mus musculus]	27	3	3
Q64378	Peptidyl-prolyl cis-trans isomerase FKBP5 [OS=Mus musculus]	8	2	2
Q64433	10 kDa heat shock protein, mitochondrial [OS=Mus musculus]	66	9	9
Q64455	Receptor-type tyrosine-protein phosphatase eta [OS=Mus musculus]	9	8	7
Q64514-1	Tripeptidyl-peptidase 2 [OS=Mus musculus]	33	27	27
Q64516-3	glycerol kinase [OS=Mus musculus]	44	17	17

Q64520	guanylate kinase [OS=Mus musculus]	14	2	2
Q64521	Glycerol-3-phosphate dehydrogenase, mitochondrial [OS=Mus musculus]	56	26	26
Q64522	Histone H2A type 2-B [OS=Mus musculus]	52	4	2
Q64674	spermidine synthase [OS=Mus musculus]	90	20	20
Q64695	Endothelial protein C receptor [OS=Mus musculus]	24	3	3
Q64701	Retinoblastoma-like protein 1 [OS=Mus musculus]	3	2	2
Q64727	Vinculin [OS=Mus musculus]	29	18	18
Q64735-1	Complement component receptor 1-like protein [OS=Mus musculus]	17	4	4
Q64737	trifunctional purine biosynthetic protein adenosine-3 [OS=Mus musculus]	43	27	27
Q64FW2	all-trans-retinol 13,14-reductase [OS=Mus musculus]	27	9	9
Q65Z40	Wings apart-like protein homolog [OS=Mus musculus]	21	13	13
Q66GT5	Phosphatidylglycerophosphatase and protein-tyrosine phosphatase 1 [OS=Mus musculus]	20	3	3
Q68FD5	Clathrin heavy chain 1 [OS=Mus musculus]	15	16	16
Q68FF6	ARF GTPase-activating protein GIT1 [OS=Mus musculus]	17	6	5
Q68FH4	N-acetylgalactosamine kinase [OS=Mus musculus]	9	3	3
Q68FL6	Methionine--tRNA ligase, cytoplasmic [OS=Mus musculus]	46	29	29
Q69Z38	Pseudopodium-enriched atypical kinase 1 [OS=Mus musculus]	15	16	16
Q69ZA1	Cyclin-dependent kinase 13 [OS=Mus musculus]	3	3	2
Q69ZK0	Phosphatidylinositol 3,4,5-trisphosphate-dependent Rac exchanger 1 protein [OS=Mus musculus]	2	2	2
Q69ZN7-1	Myoferlin [OS=Mus musculus]	25	34	34
Q69ZR2	E3 ubiquitin-protein ligase hectd1 [OS=Mus musculus]	8	11	11
Q6A026	Sister chromatid cohesion protein PDS5 homolog A [OS=Mus musculus]	36	31	28
Q6A028	Switch-associated protein 70 [OS=Mus musculus]	51	23	23
Q6A065	Centrosomal protein of 170 kDa [OS=Mus musculus]	7	7	7
Q6A068	Cell division cycle 5-like protein [OS=Mus musculus]	36	16	16

Q6A0A2	la-related protein 4B [OS=Mus musculus]	9	5	5
Q6A0A9	Constitutive coactivator of PPAR-gamma-like protein 1 [OS=Mus musculus]	16	11	11
Q6A0D4	raftlin [OS=Mus musculus]	17	4	4
Q6A4J8-2	Isoform 2 of Ubiquitin carboxyl-terminal hydrolase 7 [OS=Mus musculus]	30	20	20
Q6DFV1	condensin-2 complex subunit G2 [OS=Mus musculus]	3	3	3
Q6DFW4	Nucleolar protein 58 [OS=Mus musculus]	32	11	11
Q6DVA0	LEM domain-containing protein 2 [OS=Mus musculus]	7	3	3
Q6GSS7	Histone H2A type 2-A [OS=Mus musculus]	58	6	2
Q6IFX2	Keratin, type I cytoskeletal 42 [OS=Mus musculus]	24	10	4
Q6IFZ6	Keratin, type II cytoskeletal 1b [OS=Mus musculus]	12	4	2
Q6IRU2	Tropomyosin alpha-4 chain [OS=Mus musculus]	61	18	18
Q6KAR6	exocyst complex component 3 [OS=Mus musculus]	8	4	4
Q6NS82-1	Reticulophagy regulator 2 [OS=Mus musculus]	23	6	6
Q6NSQ7	protein LTV1 homolog [OS=Mus musculus]	7	2	2
Q6NSR8	Probable aminopeptidase NPEPL1 [OS=Mus musculus]	17	5	5
Q6NV83-1	U2 snRNP-associated SURP motif-containing protein [OS=Mus musculus]	15	10	10
Q6NVF9	Cleavage and polyadenylation specificity factor subunit 6 [OS=Mus musculus]	23	9	9
Q6NVG1	lysophospholipid acyltransferase LPCAT4 [OS=Mus musculus]	31	8	8
Q6NWW9	Fibronectin type III domain-containing protein 3B [OS=Mus musculus]	9	7	7
Q6NXI6	Regulation of nuclear pre-mRNA domain-containing protein 2 [OS=Mus musculus]	11	8	8
Q6NZB0	DnaJ homolog subfamily C member 8 [OS=Mus musculus]	28	5	5
Q6NZC7	SEC23-interacting protein [OS=Mus musculus]	16	10	10
Q6NZF1	Zinc finger CCCH domain-containing protein 11A [OS=Mus musculus]	11	6	6
Q6NZJ6	eukaryotic translation initiation factor 4 gamma 1 [OS=Mus musculus]	33	38	34
Q6NZN0-1	RNA-binding protein 26 [OS=Mus musculus]	16	12	11
Q6P069-1	Sorcin [OS=Mus musculus]	37	6	6
Q6P1B1	xaa-Pro aminopeptidase 1 [OS=Mus musculus]	45	16	16

Q6P1F6	Serine/threonine-protein phosphatase 2A 55 kDa regulatory subunit B alpha isoform [OS=Mus musculus]	30	9	9
Q6P1G0	HEAT repeat-containing protein 6 [OS=Mus musculus]	3	2	2
Q6P1H6-1	ankyrin repeat and LEM domain-containing protein 2 [OS=Mus musculus]	8	4	4
Q6P2B1-1	transportin-3 [OS=Mus musculus]	19	10	10
Q6P2K6-1	Serine/threonine-protein phosphatase 4 regulatory subunit 3A [OS=Mus musculus]	23	14	10
Q6P3A8-1	2-oxoisovalerate dehydrogenase subunit beta, mitochondrial [OS=Mus musculus]	12	3	3
Q6P4S6	Serine/threonine-protein kinase SIK3 [OS=Mus musculus]	6	5	5
Q6P4S8	integrator complex subunit 1 [OS=Mus musculus]	7	9	9
Q6P4T2	U5 small nuclear ribonucleoprotein 200 kDa helicase [OS=Mus musculus]	2	2	2
Q6P542	ATP-binding cassette sub-family F member 1 [OS=Mus musculus]	31	14	14
Q6P549	Phosphatidylinositol 3,4,5-trisphosphate 5-phosphatase 2 [OS=Mus musculus]	5	5	4
Q6P5B0	RRP12-like protein [OS=Mus musculus]	15	11	11
Q6P5D8	structural maintenance of chromosomes flexible hinge domain-containing protein 1 [OS=Mus musculus]	4	5	5
Q6P5E4	UDP-glucose:glycoprotein glucosyltransferase 1 [OS=Mus musculus]	54	52	52
Q6P5F6	Zinc transporter ZIP10 [OS=Mus musculus]	2	2	2
Q6P5F9	Exportin-1 [OS=Mus musculus]	56	45	45
Q6P5G6	UBX domain-containing protein 7 [OS=Mus musculus]	6	2	2
Q6P6J9	Thioredoxin domain-containing protein 15 [OS=Mus musculus]	10	2	2
Q6P8I4	PEST proteolytic signal-containing nuclear protein [OS=Mus musculus]	16	2	2
Q6P8X1	Sorting nexin-6 [OS=Mus musculus]	17	5	5
Q6P9L6	Kinesin-like protein KIF15 [OS=Mus musculus]	6	6	6
Q6P9P6	Kinesin-like protein KIF11 [OS=Mus musculus]	6	4	4
Q6P9Q4	FH1/FH2 domain-containing protein 1 [OS=Mus musculus]	4	3	3
Q6P9Q6-1	FK506-binding protein 15 [OS=Mus musculus]	9	7	7
Q6P9R2	Serine/threonine-protein kinase OSR1 [OS=Mus musculus]	28	9	9
Q6PA06-1	Atlastin-2 [OS=Mus musculus]	23	7	6
Q6PAM1	alpha-taxilin [OS=Mus musculus]	21	7	7
Q6PAQ4	RNA exonuclease 4 [OS=Mus musculus]	10	3	3

Q6PAR5-4	Isoform 4 of GTPase-activating protein and VPS9 domain-containing protein 1 [OS=Mus musculus]	7	8	8
Q6PB44-1	Tyrosine-protein phosphatase non-receptor type 23 [OS=Mus musculus]	4	5	5
Q6PB66	Leucine-rich PPR motif-containing protein, mitochondrial [OS=Mus musculus]	63	68	68
Q6PCM2-1	Integrator complex subunit 6 [OS=Mus musculus]	3	3	3
Q6PD03	Serine/threonine-protein phosphatase 2A 56 kDa regulatory subunit alpha isoform [OS=Mus musculus]	8	3	3
Q6PD26	GPI transamidase component PIG-S [OS=Mus musculus]	50	15	15
Q6PDG5-1	SWI/SNF complex subunit SMARCC2 [OS=Mus musculus]	16	13	8
Q6PDI5-1	Proteasome-associated protein ECM29 homolog [OS=Mus musculus]	28	34	34
Q6PDL0	Cytoplasmic dynein 1 light intermediate chain 2 [OS=Mus musculus]	30	8	7
Q6PDM2	Serine/arginine-rich splicing factor 1 [OS=Mus musculus]	37	8	8
Q6PDQ2	Chromodomain-helicase-DNA-binding protein 4 [OS=Mus musculus]	9	11	5
Q6PE01	U5 small nuclear ribonucleoprotein 40 kDa protein [OS=Mus musculus]	30	5	5
Q6PEB6	MOB-like protein phocein [OS=Mus musculus]	48	5	5
Q6PFD9	nuclear pore complex protein Nup98-Nup96 [OS=Mus musculus]	32	36	36
Q6PFQ7-1	Ras GTPase-activating protein 4 [OS=Mus musculus]	4	2	2
Q6PG16-1	Holliday junction recognition protein [OS=Mus musculus]	5	2	2
Q6PGB6-4	Isoform 4 of N-alpha-acetyltransferase 50 [OS=Mus musculus]	51	12	12
Q6PGG2	GEM-interacting protein [OS=Mus musculus]	18	9	9
Q6PGH1	Protein BUD31 homolog [OS=Mus musculus]	30	2	2
Q6PGH2	Jupiter microtubule associated homolog 2 [OS=Mus musculus]	26	3	3
Q6PGL7-1	WASH complex subunit 2 [OS=Mus musculus]	17	11	11
Q6PIC6	Sodium/potassium-transporting ATPase subunit alpha-3 [OS=Mus musculus]	17	12	2
Q6PR54-3	Isoform 3 of Telomere-associated protein RIF1 [OS=Mus musculus]	3	6	6
Q6Q899	Probable ATP-dependent RNA helicase DDX58 [OS=Mus musculus]	20	13	13
Q6R0H7	Guanine nucleotide-binding protein G(S) subunit alpha isoforms XLas [OS=Mus musculus]	14	11	10
Q6R891-1	Neurabin-2 [OS=Mus musculus]	3	2	2

Q6VN19	Ran-binding protein 10 [OS=Mus musculus]	12	5	4
Q6WVG3	BTB/POZ domain-containing protein KCTD12 [OS=Mus musculus]	42	11	10
Q6Y685	Transforming acidic coiled-coil-containing protein 1 [OS=Mus musculus]	8	4	4
Q6Y7W8-1	GRB10-interacting GYF protein 2 [OS=Mus musculus]	14	12	12
Q6ZPJ3	(E3-independent) E2 ubiquitin-conjugating enzyme UBE2O [OS=Mus musculus]	5	3	3
Q6ZPR5-1	Sphingomyelin phosphodiesterase 4 [OS=Mus musculus]	13	6	6
Q6ZPY7	Lysine-specific demethylase 3B [OS=Mus musculus]	8	7	7
Q6ZPZ3	Zinc finger CCCH domain-containing protein 4 [OS=Mus musculus]	6	4	4
Q6ZQ03-1	Formin-binding protein 4 [OS=Mus musculus]	6	4	4
Q6ZQ08-4	Isoform 4 of CCR4-NOT transcription complex subunit 1 [OS=Mus musculus]	13	22	22
Q6ZQ38	cullin-associated nedd8-dissociated protein 1 [OS=Mus musculus]	47	39	39
Q6ZQ58-1	La-related protein 1 [OS=Mus musculus]	29	19	19
Q6ZQB6	inositol hexakisphosphate and diphosphoinositol-pentakisphosphate kinase 2 [OS=Mus musculus]	4	3	3
Q6ZQI3	Malectin [OS=Mus musculus]	33	9	9
Q6ZQK0	condensin-2 complex subunit D3 [OS=Mus musculus]	3	4	4
Q6ZQK5	Arf-GAP with coiled-coil, ANK repeat and PH domain-containing protein 2 [OS=Mus musculus]	9	4	4
Q6ZQL4	WD repeat-containing protein 43 [OS=Mus musculus]	8	3	3
Q6ZQM8-1	UDP-glucuronosyltransferase 1-7C [OS=Mus musculus]	54	17	17
Q6ZWN5	40S ribosomal protein S9 [OS=Mus musculus]	34	12	12
Q6ZWQ7	Signal peptidase complex subunit 3 [OS=Mus musculus]	18	3	3
Q6ZWU9	40S ribosomal protein S27 [OS=Mus musculus]	30	3	2
Q6ZWV3	60S ribosomal protein L10 [OS=Mus musculus]	36	9	9
Q6ZWV7	60S ribosomal protein L35 [OS=Mus musculus]	23	4	4
Q6ZWX6	Eukaryotic translation initiation factor 2 subunit 1 [OS=Mus musculus]	54	14	14
Q6ZWY3	40S ribosomal protein S27-like [OS=Mus musculus]	31	3	2
Q75VT8	Protein HIDE1 [OS=Mus musculus]	15	2	2
Q76LS9-1	Ubiquitin carboxyl-terminal hydrolase MINDY-1 [OS=Mus musculus]	16	4	4
Q76MZ3	serine/threonine-protein phosphatase 2A 65 kDa regulatory subunit A alpha isoform [OS=Mus musculus]	62	28	20

Q78IK2	Up-regulated during skeletal muscle growth protein 5 [OS=Mus musculus]	45	3	3
Q78IK4	MICOS complex subunit MIC27 [OS=Mus musculus]	49	8	8
Q78JW9	Ubiquitin domain-containing protein UBFD1 [OS=Mus musculus]	8	2	2
Q78PY7	staphylococcal nuclease domain-containing protein 1 [OS=Mus musculus]	66	48	48
Q78T54	Vacuolar ATPase assembly integral membrane protein vma21 [OS=Mus musculus]	25	2	2
Q78YY6	DnaJ homolog subfamily C member 15 [OS=Mus musculus]	21	2	2
Q78ZA7	Nucleosome assembly protein 1-like 4 [OS=Mus musculus]	52	14	12
Q791V5	Mitochondrial carrier homolog 2 [OS=Mus musculus]	51	11	11
Q7JJ13-1	Bromodomain-containing protein 2 [OS=Mus musculus]	11	5	5
Q7TMF2	3'-5' exoribonuclease 1 [OS=Mus musculus]	9	2	2
Q7TMF3	NADH dehydrogenase [ubiquinone] 1 alpha subcomplex subunit 12 [OS=Mus musculus]	67	8	8
Q7TMK9	Heterogeneous nuclear ribonucleoprotein Q [OS=Mus musculus]	42	25	25
Q7TMM9	Tubulin beta-2A chain [OS=Mus musculus]	68	28	5
Q7TMR0-1	lysosomal Pro-X carboxypeptidase [OS=Mus musculus]	12	3	3
Q7TMY7	Importin-8 [OS=Mus musculus]	8	6	5
Q7TN29	Stromal membrane-associated protein 2 [OS=Mus musculus]	6	2	2
Q7TNC4	Putative RNA-binding protein Luc7-like 2 [OS=Mus musculus]	18	5	4
Q7TNE3	Sperm-associated antigen 7 [OS=Mus musculus]	22	3	3
Q7TNP2	Serine/threonine-protein phosphatase 2A 65 kDa regulatory subunit A beta isoform [OS=Mus musculus]	32	13	5
Q7TNV0	Protein DEK [OS=Mus musculus]	20	6	6
Q7TPD0	Integrator complex subunit 3 [OS=Mus musculus]	24	16	16
Q7TPR4	Alpha-actinin-1 [OS=Mus musculus]	45	31	16
Q7TPV4	Myb-binding protein 1A [OS=Mus musculus]	63	63	63
Q7TQ95	Protein lunapark [OS=Mus musculus]	35	9	9
Q7TQH0-2	Isoform 2 of Ataxin-2-like protein [OS=Mus musculus]	20	15	15
Q7TQI3	Ubiquitin thioesterase otub1 [OS=Mus musculus]	53	11	11
Q7TQK4	exosome complex component RRP40 [OS=Mus musculus]	38	5	5

Q7TQK5	Coiled-coil domain-containing protein 93 [OS=Mus musculus]	7	4	4
Q7TSC1	Protein PRRC2A [OS=Mus musculus]	5	5	5
Q7TSG2-1	RNA polymerase II subunit A C-terminal domain phosphatase [OS=Mus musculus]	14	8	8
Q7TSI3	Serine/threonine-protein phosphatase 6 regulatory subunit 1 [OS=Mus musculus]	20	10	9
Q7TSS2	Ubiquitin-conjugating enzyme E2 Q1 [OS=Mus musculus]	23	5	5
Q7TSV4	Phosphoglucomutase-2 [OS=Mus musculus]	19	6	6
Q80T69	Round spermatid basic protein 1 [OS=Mus musculus]	6	3	3
Q80TE0	RNA polymerase II-associated protein 1 [OS=Mus musculus]	3	3	3
Q80TH2-2	Isoform 2 of Erbin [OS=Mus musculus]	5	5	5
Q80TL7-1	Protein MON2 homolog [OS=Mus musculus]	2	3	3
Q80TM9-1	Nischarin [OS=Mus musculus]	6	5	5
Q80TP3	E3 ubiquitin-protein ligase UBR5 [OS=Mus musculus]	1	2	2
Q80TV8-1	CLIP-associating protein 1 [OS=Mus musculus]	7	7	4
Q80TY0	Formin-binding protein 1 [OS=Mus musculus]	32	15	15
Q80U58-1	pumilio homolog 2 [OS=Mus musculus]	5	4	3
Q80U70	Polycomb protein suz12 [OS=Mus musculus]	13	5	5
Q80U72-3	Isoform 3 of Protein scribble homolog [OS=Mus musculus]	11	9	9
Q80U87	ubiquitin carboxyl-terminal hydrolase 8 [OS=Mus musculus]	8	5	5
Q80U93	Nuclear pore complex protein Nup214 [OS=Mus musculus]	10	12	12
Q80U95	Ubiquitin-protein ligase E3C [OS=Mus musculus]	4	3	3
Q80UG5-1	Septin-9 [OS=Mus musculus]	18	7	7
Q80UJ7	Rab3 GTPase-activating protein catalytic subunit [OS=Mus musculus]	14	8	8
Q80UK0	SEC14 domain and spectrin repeat-containing protein 1 [OS=Mus musculus]	6	2	2
Q80UM3	N-alpha-acetyltransferase 15, NatA auxiliary subunit [OS=Mus musculus]	42	25	25
Q80UM7	Mannosyl-oligosaccharide glucosidase [OS=Mus musculus]	57	28	28
Q80UU9	Membrane-associated progesterone receptor component 2 [OS=Mus musculus]	56	8	7
Q80UW8	DNA-directed RNA polymerases I, II, and III subunit RPABC1 [OS=Mus musculus]	52	6	6
Q80UY1	carnosine N-methyltransferase [OS=Mus musculus]	6	2	2
Q80UY2	E3 ubiquitin-protein ligase KCMF1 [OS=Mus musculus]	7	2	2
Q80UZ2-1	Protein SDA1 homolog [OS=Mus musculus]	10	5	5
Q80V26	Inositol monophosphatase 3 [OS=Mus musculus]	19	3	3

Q80VA0	N-acetylgalactosaminyltransferase 7 [OS=Mus musculus]	6	2	2
Q80VD1	Protein Fam98b [OS=Mus musculus]	48	13	11
Q80VJ2	Steroid receptor RNA activator 1 [OS=Mus musculus]	17	2	2
Q80VJ3	2'-deoxynucleoside 5'-phosphate N-hydrolase 1 [OS=Mus musculus]	36	4	4
Q80VL1	tudor and KH domain-containing protein [OS=Mus musculus]	28	10	10
Q80VP1-2	Isoform 2 of Epsin-1 [OS=Mus musculus]	13	4	3
Q80VQ0	Aldehyde dehydrogenase family 3 member B1 [OS=Mus musculus]	18	7	7
Q80W00	Serine/threonine-protein phosphatase 1 regulatory subunit 10 [OS=Mus musculus]	10	6	6
Q80WJ7	protein LYRIC [OS=Mus musculus]	50	22	22
Q80WQ2	Protein VAC14 homolog [OS=Mus musculus]	24	12	12
Q80WW9	DDRGK domain-containing protein 1 [OS=Mus musculus]	34	5	5
Q80X41	Serine/threonine-protein kinase VRK1 [OS=Mus musculus]	15	3	3
Q80X82	Symplekin [OS=Mus musculus]	34	29	29
Q80X85	28S ribosomal protein S7, mitochondrial [OS=Mus musculus]	48	9	9
Q80X90	Filamin-B [OS=Mus musculus]	35	59	56
Q80XC2	tRNA (adenine(58)-N(1))-methyltransferase catalytic subunit TRMT61A [OS=Mus musculus]	11	2	2
Q80XI3-3	Isoform 3 of Eukaryotic translation initiation factor 4 gamma 3 [OS=Mus musculus]	10	12	8
Q80XL6	acyl-CoA dehydrogenase family member 11 [OS=Mus musculus]	4	2	2
Q80XQ2	TBC1 domain family member 5 [OS=Mus musculus]	6	3	3
Q80Y14	Glutaredoxin-related protein 5, mitochondrial [OS=Mus musculus]	22	2	2
Q80Y81	Zinc phosphodiesterase ELAC protein 2 [OS=Mus musculus]	33	16	16
Q80YD1	ATP-dependent RNA helicase SUPV3L1, mitochondrial [OS=Mus musculus]	8	3	3
Q80YQ2-2	Isoform 2 of Mediator of RNA polymerase II transcription subunit 23 [OS=Mus musculus]	3	3	3
Q80YQ8	Protein RMD5 homolog A [OS=Mus musculus]	6	2	2
Q80YR5	scaffold attachment factor B2 [OS=Mus musculus]	5	2	2
Q80YW0	Cytohesin-4 [OS=Mus musculus]	39	10	10
Q80ZK0	28S ribosomal protein S10, mitochondrial [OS=Mus musculus]	45	5	5

Q80ZS3	28S ribosomal protein S26, mitochondrial [OS=Mus musculus]	25	4	4
Q80ZW2	Protein THEM6 [OS=Mus musculus]	32	5	5
Q810A3	Tetratricopeptide repeat protein 9C [OS=Mus musculus]	16	2	2
Q810A7-1	ATP-dependent RNA helicase DDX42 [OS=Mus musculus]	28	17	17
Q810B6-1	Rabankyrin-5 [OS=Mus musculus]	22	15	15
Q810D6	Glutamate-rich WD repeat-containing protein 1 [OS=Mus musculus]	18	4	4
Q810Q5	Normal mucosa of esophagus-specific gene 1 protein [OS=Mus musculus]	29	2	2
Q810S1	Calcium uniporter regulatory subunit MCUb, mitochondrial [OS=Mus musculus]	12	3	3
Q810V0	U3 small nucleolar ribonucleoprotein protein Mpp10 [OS=Mus musculus]	10	4	4
Q811D0-3	Isoform 3 of Disks large homolog 1 [OS=Mus musculus]	8	3	3
Q8BFP9	[Pyruvate dehydrogenase (Acetyl-transferring)] kinase isozyme 1, mitochondrial [OS=Mus musculus]	25	7	7
Q8BFQ8	Glutamine amidotransferase-like class 1 domain-containing protein 1 [OS=Mus musculus]	28	4	4
Q8BFR4	N-acetylglucosamine-6-sulfatase [OS=Mus musculus]	26	8	8
Q8BFR5	elongation factor Tu, mitochondrial [OS=Mus musculus]	58	21	21
Q8BFS6-1	Serine/threonine-protein phosphatase CPPED1 [OS=Mus musculus]	11	2	2
Q8BFT2	HAUS augmin-like complex subunit 4 [OS=Mus musculus]	12	2	2
Q8BFV2	PCI domain-containing protein 2 [OS=Mus musculus]	14	4	4
Q8BFY6	Peflin [OS=Mus musculus]	25	4	4
Q8BFY9-2	Isoform 2 of Transportin-1 [OS=Mus musculus]	51	31	25
Q8BFZ3	Beta-actin-like protein 2 [OS=Mus musculus]	45	12	4
Q8BFZ9	Erlin-2 [OS=Mus musculus]	26	6	4
Q8BG05	Heterogeneous nuclear ribonucleoprotein A3 [OS=Mus musculus]	41	21	2
Q8BG05-2	Isoform 2 of Heterogeneous nuclear ribonucleoprotein A3 [OS=Mus musculus]	43	21	2
Q8BG07	Phospholipase D4 [OS=Mus musculus]	37	13	13
Q8BG30	Negative elongation factor A [OS=Mus musculus]	7	2	2
Q8BG32	26S proteasome non-ATPase regulatory subunit 11 [OS=Mus musculus]	61	24	24
Q8BG51-4	Isoform 4 of Mitochondrial Rho GTPase 1 [OS=Mus musculus]	8	5	3

Q8BG60	thioredoxin-interacting protein [OS=Mus musculus]	7	2	2
Q8BG67-2	Isoform 2 of Protein EFR3 homolog A [OS=Mus musculus]	9	5	5
Q8BG81	Polymerase delta-interacting protein 3 [OS=Mus musculus]	30	8	8
Q8BGB7	Enolase-phosphatase E1 [OS=Mus musculus]	19	2	2
Q8BGB8	Ubiquinone biosynthesis protein COQ4 homolog, mitochondrial [OS=Mus musculus]	9	2	2
Q8BGC0-1	HIV Tat-specific factor 1 homolog [OS=Mus musculus]	16	6	6
Q8BGC4	Prostaglandin reductase-3 [OS=Mus musculus]	23	5	5
Q8BGD9	eukaryotic translation initiation factor 4B [OS=Mus musculus]	24	10	10
Q8BGE6	Cysteine protease ATG4B [OS=Mus musculus]	16	4	4
Q8BGH2	sorting and assembly machinery component 50 homolog [OS=Mus musculus]	47	15	15
Q8BGQ7	Alanine--tRNA ligase, cytoplasmic [OS=Mus musculus]	48	26	26
Q8BGR2	volume-regulated anion channel subunit LRRC8D [OS=Mus musculus]	7	4	4
Q8BGR9	Ubiquitin-like domain-containing CTD phosphatase 1 [OS=Mus musculus]	12	3	3
Q8BGS2	bola-like protein 2 [OS=Mus musculus]	55	4	4
Q8BGT5	Alanine aminotransferase 2 [OS=Mus musculus]	6	2	2
Q8BGT7	Survival of motor neuron-related-splicing factor 30 [OS=Mus musculus]	38	5	5
Q8BGU5-1	cyclin-Y [OS=Mus musculus]	9	2	2
Q8BGW1	alpha-ketoglutarate-dependent dioxygenase FTO [OS=Mus musculus]	10	3	3
Q8BGX2	Mitochondrial import inner membrane translocase subunit Tim29 [OS=Mus musculus]	34	6	6
Q8BGY7	Protein FAM210A [OS=Mus musculus]	13	2	2
Q8BGZ4	cell division cycle protein 23 homolog [OS=Mus musculus]	9	4	4
Q8BH04	Phosphoenolpyruvate carboxykinase [GTP], mitochondrial [OS=Mus musculus]	53	24	24
Q8BH15	CCR4-NOT transcription complex subunit 10 [OS=Mus musculus]	6	3	3
Q8BH43	Wiskott-Aldrich syndrome protein family member 2 [OS=Mus musculus]	19	8	8
Q8BH58	TIP41-like protein [OS=Mus musculus]	35	5	5
Q8BH59	Calcium-binding mitochondrial carrier protein Aralar1 [OS=Mus musculus]	69	30	26
Q8BH69	Selenide, water dikinase 1 [OS=Mus musculus]	30	8	8
Q8BH74	Nuclear pore complex protein Nup107 [OS=Mus musculus]	33	19	19

Q8BH86	D-glutamate cyclase, mitochondrial [OS=Mus musculus]	35	13	13
Q8BH95	Enoyl-CoA hydratase, mitochondrial [OS=Mus musculus]	24	6	6
Q8BHC4	dephospho-CoA kinase domain-containing protein [OS=Mus musculus]	41	7	7
Q8BHD7	polypyrimidine tract-binding protein 3 [OS=Mus musculus]	41	11	10
Q8BHG1	Nardilysin [OS=Mus musculus]	21	17	17
Q8BHG9	CGG triplet repeat-binding protein 1 [OS=Mus musculus]	17	2	2
Q8BHJ5	F-box-like/WD repeat-containing protein TBL1XR1 [OS=Mus musculus]	25	7	4
Q8BHX1	HAUS augmin-like complex subunit 1 [OS=Mus musculus]	11	2	2
Q8BHY2	Nucleolar complex protein 4 homolog [OS=Mus musculus]	18	5	5
Q8BI72	CDKN2A-interacting protein [OS=Mus musculus]	14	5	5
Q8BI84-1	Transport and Golgi organization protein 1 homolog [OS=Mus musculus]	6	6	6
Q8BIG7	Catechol O-methyltransferase domain-containing protein 1 [OS=Mus musculus]	67	9	9
Q8BIH0-1	Histone deacetylase complex subunit SAP130 [OS=Mus musculus]	2	2	2
Q8BIJ6	Isoleucine--tRNA ligase, mitochondrial [OS=Mus musculus]	30	18	18
Q8BIJ7	RUN and FYVE domain-containing protein 1 [OS=Mus musculus]	3	2	2
Q8BIP0	Aspartate--tRNA ligase, mitochondrial [OS=Mus musculus]	32	13	13
Q8BIQ5-1	cleavage stimulation factor subunit 2 [OS=Mus musculus]	33	10	6
Q8BIW1	Exopolyphosphatase PRUNE1 [OS=Mus musculus]	10	2	2
Q8BIW9	Chromosome transmission fidelity protein 18 homolog [OS=Mus musculus]	3	2	2
Q8BJ48	N-acetylglucosamine-1-phosphodiester alpha-N-acetylglucosaminidase [OS=Mus musculus]	22	7	7
Q8BJ71-1	Nuclear pore complex protein Nup93 [OS=Mus musculus]	58	32	32
Q8BJS4	SUN domain-containing protein 2 [OS=Mus musculus]	5	2	2
Q8BJU0-1	Small glutamine-rich tetratricopeptide repeat-containing protein alpha [OS=Mus musculus]	27	6	6
Q8BJW6-1	Eukaryotic translation initiation factor 2A [OS=Mus musculus]	29	10	10
Q8BJY1	26S proteasome non-ATPase regulatory subunit 5 [OS=Mus musculus]	59	19	19

Q8BJZ4	28S ribosomal protein S35, mitochondrial [OS=Mus musculus]	48	9	9
Q8BK08	Transmembrane protein 11, mitochondrial [OS=Mus musculus]	13	2	2
Q8BK30	NADH dehydrogenase [ubiquinone] flavoprotein 3, mitochondrial [OS=Mus musculus]	37	2	2
Q8BK35	ribosome biogenesis protein NOP53 [OS=Mus musculus]	18	4	4
Q8BK63-1	Casein kinase I isoform alpha [OS=Mus musculus]	10	3	3
Q8BK64	activator of 90 kDa heat shock protein ATPase homolog 1 [OS=Mus musculus]	56	12	12
Q8BK67	Protein RCC2 [OS=Mus musculus]	42	17	17
Q8BK72	28S ribosomal protein S27, mitochondrial [OS=Mus musculus]	34	13	13
Q8BKC5-1	Importin-5 [OS=Mus musculus]	62	47	47
Q8BKC8-1	Phosphatidylinositol 4-kinase beta [OS=Mus musculus]	3	2	2
Q8BKE6	Cytochrome P450 20A1 [OS=Mus musculus]	26	7	7
Q8BKS9	Pumilio homolog 3 [OS=Mus musculus]	17	8	8
Q8BKT3	GC-rich sequence DNA-binding factor 2 [OS=Mus musculus]	5	3	3
Q8BKX1	Brain-specific angiogenesis inhibitor 1-associated protein 2 [OS=Mus musculus]	5	2	2
Q8BKZ9	Pyruvate dehydrogenase protein X component, mitochondrial [OS=Mus musculus]	21	6	6
Q8BL66	Early endosome antigen 1 [OS=Mus musculus]	31	29	29
Q8BL97-1	serine/arginine-rich splicing factor 7 [OS=Mus musculus]	11	3	2
Q8BLF1	Neutral cholesterol ester hydrolase 1 [OS=Mus musculus]	41	11	11
Q8BLH7	HIRA-interacting protein 3 [OS=Mus musculus]	6	2	2
Q8BLN5	lanosterol synthase [OS=Mus musculus]	25	14	14
Q8BM39-1	Pre-mRNA-splicing factor 18 [OS=Mus musculus]	9	3	3
Q8BM55	Transmembrane protein 214 [OS=Mus musculus]	27	13	13
Q8BM72-1	Heat shock 70 kDa protein 13 [OS=Mus musculus]	11	4	4
Q8BMA6	Signal recognition particle subunit SRP68 [OS=Mus musculus]	38	18	18
Q8BMB3	eukaryotic translation initiation factor 4E type 2 [OS=Mus musculus]	33	5	5
Q8BMC4	Nucleolar protein 9 [OS=Mus musculus]	18	6	6
Q8BMD8	Calcium-binding mitochondrial carrier protein SCaMC-1 [OS=Mus musculus]	45	14	14

Q8BMF4	Dihydrolipoyllysine-residue acetyltransferase component of pyruvate dehydrogenase complex, mitochondrial [OS=Mus musculus]	38	18	18
Q8BMG7	Rab3 GTPase-activating protein non-catalytic subunit [OS=Mus musculus]	7	7	7
Q8BMJ2	Leucine--tRNA ligase, cytoplasmic [OS=Mus musculus]	43	39	39
Q8BMK4	Cytoskeleton-associated protein 4 [OS=Mus musculus]	53	25	25
Q8BML9	glutamine--tRNA ligase [OS=Mus musculus]	53	30	30
Q8BMP6	Golgi resident protein GCP60 [OS=Mus musculus]	33	11	11
Q8BMQ2-1	General transcription factor 3C polypeptide 4 [OS=Mus musculus]	8	3	3
Q8BMS1	Trifunctional enzyme subunit alpha, mitochondrial [OS=Mus musculus]	63	35	35
Q8BMS4	Ubiquinone biosynthesis O-methyltransferase, mitochondrial [OS=Mus musculus]	20	5	5
Q8BN21	serine/threonine-protein kinase VRK2 [OS=Mus musculus]	9	3	3
Q8BNU0-1	Armadillo repeat-containing protein 6 [OS=Mus musculus]	13	3	3
Q8BNV1	tRNA (uracil-5-)-methyltransferase homolog A [OS=Mus musculus]	34	14	14
Q8BNW9	Kelch repeat and BTB domain-containing protein 11 [OS=Mus musculus]	19	7	7
Q8BP40	Lysophosphatidic acid phosphatase type 6 [OS=Mus musculus]	23	6	6
Q8BP47	Asparagine--tRNA ligase, cytoplasmic [OS=Mus musculus]	42	24	24
Q8BP48	Methionine aminopeptidase 1 [OS=Mus musculus]	15	3	3
Q8BP67	60S ribosomal protein L24 [OS=Mus musculus]	29	6	6
Q8BP92	Reticulocalbin-2 [OS=Mus musculus]	41	9	9
Q8BPB0	mob kinase activator 1b [OS=Mus musculus]	27	4	4
Q8BPM0	Disheveled-associated activator of morphogenesis 1 [OS=Mus musculus]	5	3	3
Q8BPU7-1	Engulfment and cell motility protein 1 [OS=Mus musculus]	33	17	17
Q8BPX9	Solute carrier family 15 member 3 [OS=Mus musculus]	5	2	2
Q8BQ30	Phostensin [OS=Mus musculus]	17	6	6
Q8BQ47	Protein canopy homolog 4 [OS=Mus musculus]	26	4	4
Q8BQM4	HEAT repeat-containing protein 3 [OS=Mus musculus]	32	14	14

Q8BRF7	Sec1 family domain-containing protein 1 [OS=Mus musculus]	49	17	17
Q8BRG8	Transmembrane protein 209 [OS=Mus musculus]	13	4	4
Q8BRN9	Coiled-coil and C2 domain-containing protein 1B [OS=Mus musculus]	5	2	2
Q8BRT1	CLIP-associating protein 2 [OS=Mus musculus]	10	9	2
Q8BSP2-2	Isoform 2 of Condensin-2 complex subunit H2 [OS=Mus musculus]	7	2	2
Q8BSQ9	Protein polybromo-1 [OS=Mus musculus]	6	6	6
Q8BSY0	Aspartyl/Asparaginyl beta-hydroxylase [OS=Mus musculus]	33	23	23
Q8BT07-1	Centrosomal protein of 55 kDa [OS=Mus musculus]	14	5	5
Q8BTH8	Casein kinase I isoform gamma-1 [OS=Mus musculus]	5	2	2
Q8BTI8	serine/arginine repetitive matrix protein 2 [OS=Mus musculus]	3	5	5
Q8BTM8	Filamin-A [OS=Mus musculus]	56	96	93
Q8BTS4	Nuclear pore complex protein Nup54 [OS=Mus musculus]	34	12	12
Q8BTU1-1	Cilia- and flagella-associated protein 20 [OS=Mus musculus]	19	2	2
Q8BTW3	Exosome complex component MTR3 [OS=Mus musculus]	26	5	5
Q8BTY2	Sodium bicarbonate cotransporter 3 [OS=Mus musculus]	14	7	7
Q8BTY8-1	Sec1 family domain-containing protein 2 [OS=Mus musculus]	5	2	2
Q8BTZ4-1	Anaphase-promoting complex subunit 5 [OS=Mus musculus]	7	3	3
Q8BTZ7	Mannose-1-phosphate guanyltransferase beta [OS=Mus musculus]	16	4	4
Q8BU11	TOX high mobility group box family member 4 [OS=Mus musculus]	12	5	4
Q8BU30	isoleucine--tRNA ligase, cytoplasmic [OS=Mus musculus]	40	37	37
Q8BU31	ras-related protein Rap-2c [OS=Mus musculus]	63	9	5
Q8BU88	39S ribosomal protein L22, mitochondrial [OS=Mus musculus]	26	4	4
Q8BUK6	Protein Hook homolog 3 [OS=Mus musculus]	19	9	9
Q8BUV3	Gephyrin [OS=Mus musculus]	32	12	12
Q8BV13	COP9 signalosome complex subunit 7b [OS=Mus musculus]	24	4	4
Q8BV49	Pyrin and HIN domain-containing protein 1 [OS=Mus musculus]	18	4	4
Q8BVE3	V-type proton ATPase subunit H [OS=Mus musculus]	36	11	11
Q8BVF2	phosducin-like protein 3 [OS=Mus musculus]	36	6	6
Q8BVI4	dihydropteridine reductase [OS=Mus musculus]	36	6	6
Q8BVI5	Syntaxin-16 [OS=Mus musculus]	18	3	3
Q8BVK9	SP110 nuclear body protein [OS=Mus musculus]	9	2	2
Q8BVQ5	Protein phosphatase methylesterase 1 [OS=Mus musculus]	18	4	4

Q8BVU0	Leucine-rich repeat and calponin homology domain-containing protein 3 [OS=Mus musculus]	6	3	3
Q8BVW3	Tripartite motif-containing protein 14 [OS=Mus musculus]	27	9	9
Q8BVY0	Ribosomal L1 domain-containing protein 1 [OS=Mus musculus]	45	16	16
Q8BVZ5	Interleukin-33 [OS=Mus musculus]	7	2	2
Q8BW10	RNA-binding protein NOB1 [OS=Mus musculus]	25	5	5
Q8BWF0	Succinate-semialdehyde dehydrogenase, mitochondrial [OS=Mus musculus]	6	2	2
Q8BWG8-1	Beta-arrestin-1 [OS=Mus musculus]	10	3	3
Q8BWM0	Prostaglandin E synthase 2 [OS=Mus musculus]	25	6	6
Q8BWQ6-1	UPF0505 protein C16orf62 homolog [OS=Mus musculus]	17	10	10
Q8BWT1	3-ketoacyl-CoA thiolase, mitochondrial [OS=Mus musculus]	59	15	15
Q8BWU5	Probable tRNA N6-adenosine threonylcarbamoyltransferase [OS=Mus musculus]	36	7	7
Q8BWW4	La-related protein 4 [OS=Mus musculus]	14	6	6
Q8BWY3	Eukaryotic peptide chain release factor subunit 1 [OS=Mus musculus]	51	18	18
Q8BWY9	Protein CIP2A [OS=Mus musculus]	7	4	4
Q8BWZ3	N-alpha-acetyltransferase 25, NatB auxiliary subunit [OS=Mus musculus]	21	12	12
Q8BX02	KN motif and ankyrin repeat domain-containing protein 2 [OS=Mus musculus]	4	2	2
Q8BX09	Retinoblastoma-binding protein 5 [OS=Mus musculus]	4	2	2
Q8BX10-2	Isoform 2 of Serine/threonine-protein phosphatase PGAM5, mitochondrial [OS=Mus musculus]	25	5	5
Q8BX90	Fibronectin type-III domain-containing protein 3a [OS=Mus musculus]	7	5	5
Q8BXA1	Golgi integral membrane protein 4 [OS=Mus musculus]	7	4	4
Q8BXC6	COMM domain-containing protein 2 [OS=Mus musculus]	14	3	3
Q8BXL7	ADP-ribosylation factor-related protein 1 [OS=Mus musculus]	14	2	2
Q8BXQ2	GPI transamidase component PIG-T [OS=Mus musculus]	14	6	6
Q8BXV2	BRI3-binding protein [OS=Mus musculus]	17	4	4
Q8BXZ1	Protein disulfide-isomerase TMX3 [OS=Mus musculus]	34	10	10
Q8BY71	histone acetyltransferase type B catalytic subunit [OS=Mus musculus]	41	9	9
Q8BY87-1	ubiquitin carboxyl-terminal hydrolase 47 [OS=Mus musculus]	6	6	6
Q8BYA0	Tubulin-specific chaperone D [OS=Mus musculus]	11	8	8

Q8BYB9	Protein O-glucosyltransferase 1 [OS=Mus musculus]	8	3	3
Q8BYC6	Serine/threonine-protein kinase TAO3 [OS=Mus musculus]	9	7	7
Q8BYI6-1	Lysophosphatidylcholine acyltransferase 2 [OS=Mus musculus]	16	5	5
Q8BYK4	Retinol dehydrogenase 12 [OS=Mus musculus]	11	3	2
Q8BYK6-3	Isoform 3 of YTH domain-containing family protein 3 [OS=Mus musculus]	12	5	5
Q8BYL4	Tyrosine--tRNA ligase, mitochondrial [OS=Mus musculus]	36	10	10
Q8BYM8	probable cysteine--tRNA ligase, mitochondrial [OS=Mus musculus]	6	2	2
Q8BYP3	Rho-related GTP-binding protein RhoF [OS=Mus musculus]	16	2	2
Q8BYU6	Torsin-1A-interacting protein 2 [OS=Mus musculus]	7	2	2
Q8BZA9	Fructose-2,6-bisphosphatase TIGAR [OS=Mus musculus]	28	5	5
Q8BZM1	Glomulin [OS=Mus musculus]	7	3	3
Q8BZN6-4	Isoform 4 of Dedicator of cytokinesis protein 10 [OS=Mus musculus]	9	12	12
Q8BZQ7	anaphase-promoting complex subunit 2 [OS=Mus musculus]	7	4	4
Q8C052	Microtubule-associated protein 1S [OS=Mus musculus]	17	10	10
Q8C079	striatin-interacting protein 1 [OS=Mus musculus]	10	4	4
Q8C0C7	Phenylalanine--tRNA ligase alpha subunit [OS=Mus musculus]	42	14	14
Q8C0I1	Alkyldihydroxyacetonephosphate synthase, peroxisomal [OS=Mus musculus]	64	26	26
Q8C0L0	Thioredoxin-related transmembrane protein 4 [OS=Mus musculus]	29	5	5
Q8C129	Leucyl-cystinyl aminopeptidase [OS=Mus musculus]	32	25	25
Q8C147	Dedicator of cytokinesis protein 8 [OS=Mus musculus]	17	25	24
Q8C156	Condensin complex subunit 2 [OS=Mus musculus]	25	11	11
Q8C166	Copine-1 [OS=Mus musculus]	28	10	10
Q8C1A5	thimet oligopeptidase [OS=Mus musculus]	25	12	12
Q8C1B7	Septin-11 [OS=Mus musculus]	48	15	11
Q8C1D8	protein IWS1 homolog [OS=Mus musculus]	4	3	3
Q8C2Q3	RNA-binding protein 14 [OS=Mus musculus]	20	8	8
Q8C3I8	Protein HGH1 homolog [OS=Mus musculus]	10	2	2
Q8C3J5	Dedicator of cytokinesis protein 2 [OS=Mus musculus]	9	13	13

Q8C3X2	Coiled-coil domain-containing protein 90B, mitochondrial [OS=Mus musculus]	23	4	4
Q8C436	Protein-lysine methyltransferase mettl21d [OS=Mus musculus]	15	2	2
Q8C4B4	Protein unc-119 homolog B [OS=Mus musculus]	16	3	2
Q8C4Y3	Negative elongation factor B [OS=Mus musculus]	15	5	5
Q8C570	mRNA export factor [OS=Mus musculus]	49	9	9
Q8C5H8-1	NAD kinase 2, mitochondrial [OS=Mus musculus]	22	6	6
Q8C5L3-1	CCR4-NOT transcription complex subunit 2 [OS=Mus musculus]	17	3	3
Q8C5P5-1	5'-nucleotidase domain-containing protein 1 [OS=Mus musculus]	15	4	4
Q8C5Q4	G-rich sequence factor 1 [OS=Mus musculus]	26	8	8
Q8C6B9	Active regulator of SIRT1 [OS=Mus musculus]	22	2	2
Q8C6G8	WD repeat-containing protein 26 [OS=Mus musculus]	18	7	7
Q8C7K6	Prenylcysteine oxidase-like [OS=Mus musculus]	11	3	3
Q8C7Q4-1	RNA-binding protein 4 [OS=Mus musculus]	7	2	2
Q8C7R4	Ubiquitin-like modifier-activating enzyme 6 [OS=Mus musculus]	23	17	17
Q8C7V8-1	Coiled-coil domain-containing protein 134 [OS=Mus musculus]	23	3	3
Q8C854-4	Myelin expression factor 2 [OS=Mus musculus]	15	7	7
Q8C8U0-3	Isoform 3 of Liprin-beta-1 [OS=Mus musculus]	5	4	4
Q8C9B9	Death-inducer obliterator 1 [OS=Mus musculus]	5	6	6
Q8CAQ8-1	MICOS complex subunit Mic60 [OS=Mus musculus]	54	31	2
Q8CAY6	Acetyl-CoA acetyltransferase, cytosolic [OS=Mus musculus]	46	8	8
Q8CB77	Elongin-A [OS=Mus musculus]	23	8	8
Q8CB96	Ras association domain-containing protein 4 [OS=Mus musculus]	27	5	4
Q8CBB9	Radical S-adenosyl methionine domain-containing protein 2 [OS=Mus musculus]	17	4	4
Q8CBW3-1	abl interactor 1 [OS=Mus musculus]	12	6	5
Q8CBY8-1	Dynactin subunit 4 [OS=Mus musculus]	15	5	5
Q8CC88	von Willebrand factor A domain-containing protein 8 [OS=Mus musculus]	3	5	5
Q8CCB4	Vacuolar protein sorting-associated protein 53 homolog [OS=Mus musculus]	7	3	3
Q8CCF0	U4/U6 small nuclear ribonucleoprotein Prp31 [OS=Mus musculus]	40	13	13
Q8CCJ3-3	E3 UFM1-protein ligase 1 [OS=Mus musculus]	33	19	19
Q8CCM6-2	Isoform 2 of Mitochondrial import inner membrane translocase subunit Tim21 [OS=Mus musculus]	12	2	2
Q8CCS6	polyadenylate-binding protein 2 [OS=Mus musculus]	36	5	5
Q8CD10	calcium uptake protein 2, mitochondrial [OS=Mus musculus]	12	3	3

Q8CD15	ribosomal oxygenase 2 [OS=Mus musculus]	20	5	5
Q8CDG3	Deubiquitinating protein VCIP135 [OS=Mus musculus]	6	5	5
Q8CDM8	Protein FAM160B1 [OS=Mus musculus]	2	2	2
Q8CDN6	Thioredoxin-like protein 1 [OS=Mus musculus]	72	18	18
Q8CE96	tRNA (adenine(58)-N(1))-methyltransferase non-catalytic subunit TRM6 [OS=Mus musculus]	12	4	4
Q8CEC0	Nuclear pore complex protein Nup88 [OS=Mus musculus]	27	11	11
Q8CEE7	Retinol dehydrogenase 13 [OS=Mus musculus]	13	3	3
Q8CF66	ragulator complex protein LAMTOR4 [OS=Mus musculus]	28	2	2
Q8CFB4	Guanylate-binding protein 5 [OS=Mus musculus]	6	2	2
Q8CFD4	Sorting nexin-8 [OS=Mus musculus]	5	2	2
Q8CFE2	Histone PARylation factor 1 [OS=Mus musculus]	25	6	6
Q8CFE3	REST corepressor 1 [OS=Mus musculus]	8	2	2
Q8CFE6	sodium-coupled neutral amino acid transporter 2 [OS=Mus musculus]	7	2	2
Q8CFI7	DNA-directed RNA polymerase II subunit RPB2 [OS=Mus musculus]	14	11	11
Q8CFQ3	Intron-binding protein aquarius [OS=Mus musculus]	1	2	2
Q8CFX1	GDH/6PGL endoplasmic bifunctional protein [OS=Mus musculus]	17	8	8
Q8CG48	structural maintenance of chromosomes protein 2 [OS=Mus musculus]	37	35	35
Q8CG72-1	Poly(ADP-ribose) glycohydrolase ARH3 [OS=Mus musculus]	14	3	3
Q8CG76	aflatoxin B1 aldehyde reductase member 2 [OS=Mus musculus]	21	5	5
Q8CGA0	Protein phosphatase 1F [OS=Mus musculus]	11	2	2
Q8CGC6	RNA-binding protein 28 [OS=Mus musculus]	10	6	6
Q8CGC7	Bifunctional glutamate/proline--tRNA ligase [OS=Mus musculus]	46	50	50
Q8CGF7-1	Transcription elongation regulator 1 [OS=Mus musculus]	17	15	15
Q8CGK3	Lon protease homolog, mitochondrial [OS=Mus musculus]	60	39	39
Q8CGP2-2	Isoform 2 of Histone H2B type 1-P [OS=Mus musculus]	25	4	4
Q8CGY8-1	UDP-N-acetylglucosamine--peptide N-acetylglucosaminyltransferase 110 kDa subunit [OS=Mus musculus]	24	19	19
Q8CGZ0	Calcium homeostasis endoplasmic reticulum protein [OS=Mus musculus]	17	10	10
Q8CH02	SURP and G-patch domain-containing protein 1 [OS=Mus musculus]	5	2	2
Q8CH18-1	Cell division cycle and apoptosis regulator protein 1 [OS=Mus musculus]	10	7	7

Q8CH25-1	SAFB-like transcription modulator [OS=Mus musculus]	3	2	2
Q8CH77	neuron navigator 1 [OS=Mus musculus]	3	2	2
Q8CHG3	GRIP and coiled-coil domain-containing protein 2 [OS=Mus musculus]	2	3	3
Q8CHH9-2	Isoform 2 of Septin-8 [OS=Mus musculus]	17	5	3
Q8CHP8	glycerol-3-phosphate phosphatase [OS=Mus musculus]	51	8	8
Q8CHT0	Delta-1-pyrroline-5-carboxylate dehydrogenase, mitochondrial [OS=Mus musculus]	12	4	4
Q8CHT3	Integrator complex subunit 5 [OS=Mus musculus]	7	5	5
Q8CHW4	Translation initiation factor eIF-2B subunit epsilon [OS=Mus musculus]	20	8	8
Q8CHY6	Transcriptional repressor p66 alpha [OS=Mus musculus]	6	2	2
Q8CI11	Guanine nucleotide-binding protein-like 3 [OS=Mus musculus]	43	17	17
Q8CI32	BAG family molecular chaperone regulator 5 [OS=Mus musculus]	12	3	3
Q8CI33-1	CWF19-like protein 1 [OS=Mus musculus]	6	2	2
Q8CI71	Syndetin [OS=Mus musculus]	11	6	6
Q8CI94	Glycogen phosphorylase, brain form [OS=Mus musculus]	28	19	19
Q8CIG8	Protein arginine N-methyltransferase 5 [OS=Mus musculus]	15	5	5
Q8CIH5	1-phosphatidylinositol 4,5-bisphosphate phosphodiesterase gamma-2 [OS=Mus musculus]	21	16	16
Q8CII2-1	Cell division cycle protein 123 homolog [OS=Mus musculus]	8	2	2
Q8CIM8-1	integrator complex subunit 4 [OS=Mus musculus]	5	3	3
Q8CIN4	Serine/threonine-protein kinase PAK 2 [OS=Mus musculus]	61	19	14
Q8CIV8	Tubulin-specific chaperone E [OS=Mus musculus]	14	4	4
Q8JZK9	hydroxymethylglutaryl-CoA synthase, cytoplasmic [OS=Mus musculus]	33	10	10
Q8JZM0	Dimethyladenosine transferase 1, mitochondrial [OS=Mus musculus]	11	2	2
Q8JZM7	Parafibromin [OS=Mus musculus]	12	4	4
Q8JZN5	Acyl-CoA dehydrogenase family member 9, mitochondrial [OS=Mus musculus]	38	15	15
Q8JZN7	Mitochondrial Rho GTPase 2 [OS=Mus musculus]	12	5	3
Q8JZQ2	AFG3-like protein 2 [OS=Mus musculus]	14	8	8
Q8JZQ9	Eukaryotic translation initiation factor 3 subunit B [OS=Mus musculus]	35	18	18
Q8JZR0	Long-chain-fatty-acid--CoA ligase 5 [OS=Mus musculus]	44	20	18
Q8JZS9	39S ribosomal protein L48, mitochondrial [OS=Mus musculus]	11	2	2

Q8JZU2	Tricarboxylate transport protein, mitochondrial [OS=Mus musculus]	27	5	5
Q8JZV7	N-acetylglucosamine-6-phosphate deacetylase [OS=Mus musculus]	19	4	4
Q8JZX4	Splicing factor 45 [OS=Mus musculus]	20	5	4
Q8JZY4	Mitochondrial ribonuclease P protein 3 [OS=Mus musculus]	5	2	2
Q8K009	Mitochondrial 10-formyltetrahydrofolate dehydrogenase [OS=Mus musculus]	30	17	17
Q8K012	Formin-binding protein 1-like [OS=Mus musculus]	10	4	4
Q8K019-1	Bcl-2-associated transcription factor 1 [OS=Mus musculus]	5	3	3
Q8K021	Secretory carrier-associated membrane protein 1 [OS=Mus musculus]	26	4	4
Q8K0C1-2	Isoform 2 of Importin-13 [OS=Mus musculus]	3	2	2
Q8K0C4	lanosterol 14-alpha demethylase [OS=Mus musculus]	41	14	14
Q8K0C9	GDP-mannose 4,6 dehydratase [OS=Mus musculus]	12	3	3
Q8K0D5	Elongation factor G, mitochondrial [OS=Mus musculus]	25	11	11
Q8K0F1	TBC1 domain family member 23 [OS=Mus musculus]	16	5	5
Q8K0G5	EARP-interacting protein [OS=Mus musculus]	8	2	2
Q8K0Q5	Rho GTPase-activating protein 18 [OS=Mus musculus]	6	3	3
Q8K0V4	CCR4-NOT transcription complex subunit 3 [OS=Mus musculus]	4	2	2
Q8K0Z7	Translational activator of cytochrome c oxidase 1 [OS=Mus musculus]	22	4	4
Q8K124	pleckstrin homology domain-containing family O member 2 [OS=Mus musculus]	44	12	12
Q8K183	Pyridoxal kinase [OS=Mus musculus]	25	6	6
Q8K194-2	Isoform 2 of U4/U6.U5 small nuclear ribonucleoprotein 27 kDa protein [OS=Mus musculus]	15	2	2
Q8K1A6	Coiled-coil and C2 domain-containing protein 1A [OS=Mus musculus]	3	2	2
Q8K1B8	Fermitin family homolog 3 [OS=Mus musculus]	27	11	11
Q8K1C9	Leucine-rich repeat-containing protein 41 [OS=Mus musculus]	3	2	2
Q8K1E0-1	Syntaxin-5 [OS=Mus musculus]	17	4	4
Q8K1I7	WAS/WASL-interacting protein family member 1 [OS=Mus musculus]	15	4	4
Q8K1J6-1	CCA tRNA nucleotidyltransferase 1, mitochondrial [OS=Mus musculus]	19	6	6
Q8K1M6-3	Isoform 3 of Dynamin-1-like protein [OS=Mus musculus]	57	26	26
Q8K1R3	Polyribonucleotide nucleotidyltransferase 1, mitochondrial [OS=Mus musculus]	18	10	10

Q8K1T1	Leucine-rich repeat-containing protein 25 [OS=Mus musculus]	27	5	5
Q8K1Z0	Ubiquinone biosynthesis protein COQ9, mitochondrial [OS=Mus musculus]	26	5	5
Q8K202	DNA-directed RNA polymerase I subunit RPA49 [OS=Mus musculus]	5	2	2
Q8K224	RNA cytidine acetyltransferase [OS=Mus musculus]	3	2	2
Q8K273	Membrane magnesium transporter 1 [OS=Mus musculus]	34	2	2
Q8K297	Procollagen galactosyltransferase 1 [OS=Mus musculus]	27	13	13
Q8K298	Anillin [OS=Mus musculus]	8	6	6
Q8K2B3	Succinate dehydrogenase [ubiquinone] flavoprotein subunit, mitochondrial [OS=Mus musculus]	45	17	17
Q8K2C7	Protein os-9 [OS=Mus musculus]	22	7	7
Q8K2D3	Enhancer of mRNA-decapping protein 3 [OS=Mus musculus]	18	5	5
Q8K2F0	bromodomain-containing protein 3 [OS=Mus musculus]	6	3	2
Q8K2F8	protein LSM14 homolog A [OS=Mus musculus]	12	3	3
Q8K2H2	OTU domain-containing protein 6B [OS=Mus musculus]	9	2	2
Q8K2K6-4	Arf-GAP domain and FG repeat-containing protein 1 [OS=Mus musculus]	15	5	5
Q8K2M0	39S ribosomal protein L38, mitochondrial [OS=Mus musculus]	31	7	7
Q8K2Q0	COMM domain-containing protein 9 [OS=Mus musculus]	54	6	6
Q8K2Q7-1	BRO1 domain-containing protein BROX [OS=Mus musculus]	16	4	4
Q8K2Q9-1	Shootin-1 [OS=Mus musculus]	26	12	12
Q8K2T1-1	NmrA-like family domain-containing protein 1 [OS=Mus musculus]	29	6	6
Q8K2T8	RNA polymerase II-associated factor 1 homolog [OS=Mus musculus]	7	2	2
Q8K2V1-1	Serine/threonine-protein phosphatase 4 regulatory subunit 1 [OS=Mus musculus]	2	2	2
Q8K2V6-1	Importin-11 [OS=Mus musculus]	25	11	11
Q8K2X3	CST complex subunit STN1 [OS=Mus musculus]	14	3	3
Q8K2Y7	39S ribosomal protein L47, mitochondrial [OS=Mus musculus]	14	5	5
Q8K2Z4-2	Isoform 2 of Condensin complex subunit 1 [OS=Mus musculus]	34	31	31
Q8K310	Matrin-3 [OS=Mus musculus]	36	20	20
Q8K327	Chromosome alignment-maintaining phosphoprotein 1 [OS=Mus musculus]	3	2	2
Q8K354	Carbonyl reductase [NADPH] 3 [OS=Mus musculus]	31	6	5
Q8K363	ATP-dependent RNA helicase DDX18 [OS=Mus musculus]	33	13	13
Q8K368-1	Fanconi anemia group I protein homolog [OS=Mus musculus]	3	2	2

Q8K385	Ferric-chelate reductase 1 [OS=Mus musculus]	4	2	2
Q8K3A9	7SK snRNA methylphosphate capping enzyme [OS=Mus musculus]	12	4	4
Q8K3H0	DCC-interacting protein 13-alpha [OS=Mus musculus]	21	8	8
Q8K3J1	NADH dehydrogenase [ubiquinone] iron-sulfur protein 8, mitochondrial [OS=Mus musculus]	23	4	4
Q8K3W0-2	BRISC and BRCA1-A complex member 2 [OS=Mus musculus]	14	4	4
Q8K3X4	interferon regulatory factor 2-binding protein-like [OS=Mus musculus]	32	13	10
Q8K3Z9	Nuclear envelope pore membrane protein POM 121 [OS=Mus musculus]	5	3	3
Q8K411-1	Presequence protease, mitochondrial [OS=Mus musculus]	31	20	20
Q8K482	EMILIN-2 [OS=Mus musculus]	8	5	5
Q8K4B0	Metastasis-associated protein MTA1 [OS=Mus musculus]	9	5	2
Q8K4F5	Protein Abhd11 [OS=Mus musculus]	38	5	5
Q8K4F6	Probable 28S rRNA (cytosine-C(5))-methyltransferase [OS=Mus musculus]	13	4	4
Q8K4I3	Rho guanine nucleotide exchange factor 6 [OS=Mus musculus]	23	11	9
Q8K4M5	COMM domain-containing protein 1 [OS=Mus musculus]	24	3	3
Q8K4P0	pre-mRNA 3' end processing protein WDR33 [OS=Mus musculus]	1	2	2
Q8K4Q8	Collectin-12 [OS=Mus musculus]	16	8	8
Q8K4Z3	NAD(P)H-hydrate epimerase [OS=Mus musculus]	30	6	6
Q8K4Z5	splicing factor 3A subunit 1 [OS=Mus musculus]	40	22	22
Q8QZS1	3-hydroxyisobutyryl-CoA hydrolase, mitochondrial [OS=Mus musculus]	42	12	12
Q8QZT1	Acetyl-CoA acetyltransferase, mitochondrial [OS=Mus musculus]	49	16	16
Q8QZV7	integrator complex subunit 13 [OS=Mus musculus]	5	2	2
Q8QZY1	eukaryotic translation initiation factor 3 subunit L [OS=Mus musculus]	47	19	19
Q8QZY9	Splicing factor 3b subunit 4 [OS=Mus musculus]	23	5	5
Q8QZZ7	EKC/KEOPS complex subunit TPRKB [OS=Mus musculus]	10	2	2
Q8R001	Microtubule-associated protein RP/EB family member 2 [OS=Mus musculus]	10	2	2
Q8R010	aminoacyl tRNA synthase complex-interacting multifunctional protein 2 [OS=Mus musculus]	52	8	8
Q8R016	bleomycin hydrolase [OS=Mus musculus]	49	17	17
Q8R035-1	Peptidyl-tRNA hydrolase ICT1, mitochondrial [OS=Mus musculus]	20	3	3

Q8R050	Eukaryotic peptide chain release factor GTP-binding subunit ERF3A [OS=Mus musculus]	8	3	3
Q8R059	UDP-glucose 4-epimerase [OS=Mus musculus]	14	3	3
Q8R081	Heterogeneous nuclear ribonucleoprotein L [OS=Mus musculus]	65	20	20
Q8R087-1	Beta-1,4-galactosyltransferase 7 [OS=Mus musculus]	9	2	2
Q8R0A0	General transcription factor IIF subunit 2 [OS=Mus musculus]	43	6	6
Q8R0F3	sulfatase-modifying factor 1 [OS=Mus musculus]	12	3	3
Q8R0F6	Integrin-linked kinase-associated serine/threonine phosphatase 2C [OS=Mus musculus]	12	3	3
Q8R0G9	Nuclear pore complex protein Nup133 [OS=Mus musculus]	4	2	2
Q8R0H9	ADP-ribosylation factor-binding protein GGA1 [OS=Mus musculus]	16	4	4
Q8R0X7	sphingosine-1-phosphate lyase 1 [OS=Mus musculus]	18	7	7
Q8R146-2	Isoform 2 of Acylamino-acid-releasing enzyme [OS=Mus musculus]	17	5	5
Q8R164	valacyclovir hydrolase [OS=Mus musculus]	34	6	6
Q8R180	ERO1-like protein alpha [OS=Mus musculus]	65	22	20
Q8R1B4	Eukaryotic translation initiation factor 3 subunit C [OS=Mus musculus]	33	25	25
Q8R1F1	Niban-like protein 1 [OS=Mus musculus]	40	20	20
Q8R1F5	Putative hydroxypyruvate isomerase [OS=Mus musculus]	8	2	2
Q8R1G6-1	PDZ and LIM domain protein 2 [OS=Mus musculus]	11	3	3
Q8R1I1	cytochrome b-c1 complex subunit 9 [OS=Mus musculus]	38	2	2
Q8R1J9	Torsin-2A [OS=Mus musculus]	17	3	3
Q8R1N4	NudC domain-containing protein 3 [OS=Mus musculus]	12	3	3
Q8R1Q8	Cytoplasmic dynein 1 light intermediate chain 1 [OS=Mus musculus]	49	16	15
Q8R1S0	Ubiquinone biosynthesis monooxygenase COQ6, mitochondrial [OS=Mus musculus]	6	2	2
Q8R1T1-1	Charged multivesicular body protein 7 [OS=Mus musculus]	4	2	2
Q8R1V4	Transmembrane emp24 domain-containing protein 4 [OS=Mus musculus]	37	7	6
Q8R1X6	Spartin [OS=Mus musculus]	10	3	3
Q8R2E9	ERO1-like protein beta [OS=Mus musculus]	20	8	6
Q8R2K1	fucose mutarotase [OS=Mus musculus]	18	2	2
Q8R2M2	Deoxynucleotidyltransferase terminal-interacting protein 2 [OS=Mus musculus]	4	2	2
Q8R2Q4	Ribosome-releasing factor 2, mitochondrial [OS=Mus musculus]	4	2	2
Q8R2Q8	bone marrow stromal antigen 2 [OS=Mus musculus]	11	2	2
Q8R2T8	general transcription factor 3C polypeptide 5 [OS=Mus musculus]	13	5	5

Q8R2U4	N-terminal Xaa-Pro-Lys N-methyltransferase 1 [OS=Mus musculus]	16	3	3
Q8R2Y0-1	Monoacylglycerol lipase ABHD6 [OS=Mus musculus]	18	4	4
Q8R2Y8	Peptidyl-tRNA hydrolase 2, mitochondrial [OS=Mus musculus]	46	6	6
Q8R317	Ubiquilin-1 [OS=Mus musculus]	22	9	7
Q8R322	Nucleoporin GLE1 [OS=Mus musculus]	4	2	2
Q8R323	replication factor C subunit 3 [OS=Mus musculus]	38	9	9
Q8R326	Paraspeckle component 1 [OS=Mus musculus]	15	6	6
Q8R332	Nucleoporin p58/p45 [OS=Mus musculus]	13	6	6
Q8R361	Rab11 family-interacting protein 5 [OS=Mus musculus]	9	4	4
Q8R366	Immunoglobulin superfamily member 8 [OS=Mus musculus]	18	7	7
Q8R3C0	Mini-chromosome maintenance complex-binding protein [OS=Mus musculus]	35	13	13
Q8R3C6-1	Probable RNA-binding protein 19 [OS=Mus musculus]	6	4	4
Q8R3F5	Malonyl-CoA-acyl carrier protein transacylase, mitochondrial [OS=Mus musculus]	15	4	4
Q8R3F9	Speckle targeted PIP5K1A-regulated poly(A) polymerase [OS=Mus musculus]	5	2	2
Q8R3G1	Nuclear inhibitor of protein phosphatase 1 [OS=Mus musculus]	29	5	5
Q8R3H7	Heparan sulfate 2-O-sulfotransferase 1 [OS=Mus musculus]	10	2	2
Q8R3L2-1	Transcription factor 25 [OS=Mus musculus]	12	4	4
Q8R3N1	Nucleolar protein 14 [OS=Mus musculus]	15	8	8
Q8R3P2	Probable E3 ubiquitin-protein ligase DTX2 [OS=Mus musculus]	5	2	2
Q8R3P6-1	Integrator complex subunit 14 [OS=Mus musculus]	15	4	4
Q8R3Q6	coiled-coil domain-containing protein 58 [OS=Mus musculus]	53	7	7
Q8R3V5-4	Endophilin-B2 [OS=Mus musculus]	13	3	3
Q8R3Y8	Interferon regulatory factor 2-binding protein 1 [OS=Mus musculus]	7	2	2
Q8R404	MICOS complex subunit MIC13 [OS=Mus musculus]	62	3	3
Q8R409	Protein HEXIM1 [OS=Mus musculus]	20	3	3
Q8R480	nuclear pore complex protein Nup85 [OS=Mus musculus]	26	15	15
Q8R4N0	Citrate lyase subunit beta-like protein, mitochondrial [OS=Mus musculus]	22	4	4
Q8R4R6	Nucleoporin NUP53 [OS=Mus musculus]	41	8	8
Q8R4U7	Leucine zipper protein 1 [OS=Mus musculus]	4	3	3
Q8R4X3	RNA-binding protein 12 [OS=Mus musculus]	8	5	5
Q8R574	Phosphoribosyl pyrophosphate synthase-associated protein 2 [OS=Mus musculus]	37	9	8
Q8R5A3	Amyloid beta A4 precursor protein-binding family B member 1-interacting protein [OS=Mus musculus]	13	5	5
Q8R5C5	Beta-centractin [OS=Mus musculus]	45	10	3

Q8R5F7-1	Interferon-induced helicase C domain-containing protein 1 [OS=Mus musculus]	2	2	2
Q8R5H1-1	ubiquitin carboxyl-terminal hydrolase 15 [OS=Mus musculus]	30	20	19
Q8R5K4	Nucleolar protein 6 [OS=Mus musculus]	3	2	2
Q8R5M8	Cell adhesion molecule 1 [OS=Mus musculus]	25	6	6
Q8VBT0	Thioredoxin-related transmembrane protein 1 [OS=Mus musculus]	13	3	3
Q8VBT6	Apolipoprotein B receptor [OS=Mus musculus]	40	26	26
Q8VBT9-1	tether containing UBX domain for GLUT4 [OS=Mus musculus]	18	5	5
Q8VBV3	Exosome complex component Rrp4 [OS=Mus musculus]	14	2	2
Q8VBV7	COP9 signalosome complex subunit 8 [OS=Mus musculus]	57	6	6
Q8VBW6	NEDD8-activating enzyme E1 regulatory subunit [OS=Mus musculus]	19	5	5
Q8VBZ0	Dehydrogenase/reductase SDR family member on chromosome X homolog [OS=Mus musculus]	11	3	3
Q8VC04	transmembrane protein 106A [OS=Mus musculus]	16	2	2
Q8VC19	5-aminolevulinate synthase, nonspecific, mitochondrial [OS=Mus musculus]	7	3	3
Q8VC57	BTB/POZ domain-containing protein KCTD5 [OS=Mus musculus]	16	2	2
Q8VCC1	15-hydroxyprostaglandin dehydrogenase [NAD(+)] [OS=Mus musculus]	15	3	3
Q8VCF0	Mitochondrial antiviral-signaling protein [OS=Mus musculus]	35	8	8
Q8VCH8	UBX domain-containing protein 4 [OS=Mus musculus]	23	5	5
Q8VCL2	Protein SCO2 homolog, mitochondrial [OS=Mus musculus]	11	2	2
Q8VCM8	Nicalin [OS=Mus musculus]	29	13	13
Q8VCS3	Glycosaminoglycan xylosylkinase [OS=Mus musculus]	7	2	2
Q8VCT3	aminopeptidase B [OS=Mus musculus]	38	17	17
Q8VCW8	Acyl-CoA synthetase family member 2, mitochondrial [OS=Mus musculus]	7	2	2
Q8VCY6	U3 small nucleolar RNA-associated protein 6 homolog [OS=Mus musculus]	4	2	2
Q8VD00	sigma intracellular receptor 2 [OS=Mus musculus]	24	2	2
Q8VD04	GRIP1-associated protein 1 [OS=Mus musculus]	24	13	13
Q8VD58	Protein EVI2B [OS=Mus musculus]	13	4	4
Q8VD75	Huntingtin-interacting protein 1 [OS=Mus musculus]	8	4	4
Q8VDC0	Probable Leucine--tRNA ligase, mitochondrial [OS=Mus musculus]	9	4	4

Q8VDD5	Myosin-9 [OS=Mus musculus]	54	103	88
Q8VDD8	WASH complex subunit 1 [OS=Mus musculus]	9	2	2
Q8VDF2	E3 ubiquitin-protein ligase UHRF1 [OS=Mus musculus]	7	4	4
Q8VDG3	poly(A)-specific ribonuclease PARN [OS=Mus musculus]	4	2	2
Q8VDJ3	Vigilin [OS=Mus musculus]	35	29	29
Q8VDK1-1	Deaminated glutathione amidase [OS=Mus musculus]	24	5	5
Q8VDL4	ADP-dependent glucokinase [OS=Mus musculus]	42	13	13
Q8VDM4	26S proteasome non-ATPase regulatory subunit 2 [OS=Mus musculus]	59	41	41
Q8VDM6	Heterogeneous nuclear ribonucleoprotein U-like protein 1 [OS=Mus musculus]	23	14	14
Q8VDN2	Sodium/potassium-transporting ATPase subunit alpha-1 [OS=Mus musculus]	36	25	15
Q8VDP4	Cell cycle and apoptosis regulator protein 2 [OS=Mus musculus]	47	25	25
Q8VDQ1-1	Prostaglandin reductase 2 [OS=Mus musculus]	17	3	3
Q8VDQ8-1	NAD-dependent protein deacetylase sirtuin-2 [OS=Mus musculus]	21	5	5
Q8VDS8-1	Syntaxin-18 [OS=Mus musculus]	16	3	3
Q8VDT9	39S ribosomal protein L50, mitochondrial [OS=Mus musculus]	20	2	2
Q8VDV3	Guanine nucleotide exchange factor for Rab-3A [OS=Mus musculus]	33	7	7
Q8VDW0-1	ATP-dependent RNA helicase DDX39A [OS=Mus musculus]	59	20	10
Q8VE18	Protein SMG8 [OS=Mus musculus]	4	2	2
Q8VE22	28S ribosomal protein S23, mitochondrial [OS=Mus musculus]	49	8	8
Q8VE37	Regulator of chromosome condensation [OS=Mus musculus]	74	16	16
Q8VE38-1	Oxidoreductase NAD-binding domain-containing protein 1 [OS=Mus musculus]	7	2	2
Q8VE47	Ubiquitin-like modifier-activating enzyme 5 [OS=Mus musculus]	29	6	6
Q8VE62	Polyadenylate-binding protein-interacting protein 1 [OS=Mus musculus]	11	3	3
Q8VE70	programmed cell death protein 10 [OS=Mus musculus]	22	3	3
Q8VE88-2	Isoform 2 of Protein FAM114A2 [OS=Mus musculus]	25	7	7
Q8VE91	Reticulophagy regulator 1 [OS=Mus musculus]	25	5	5
Q8VE99	Coiled-coil domain-containing protein 115 [OS=Mus musculus]	49	7	7
Q8VEA4	Mitochondrial intermembrane space import and assembly protein 40 [OS=Mus musculus]	37	2	2

Q8VEA8-1	ras-related protein Rab-7b [OS=Mus musculus]	15	2	2
Q8VEB4	Group XV phospholipase A2 [OS=Mus musculus]	17	5	5
Q8VEC4	Calcium homeostasis modulator protein 2 [OS=Mus musculus]	8	2	2
Q8VEE4	Replication protein A 70 kDa DNA-binding subunit [OS=Mus musculus]	21	8	8
Q8VEH3	ADP-ribosylation factor-like protein 8A [OS=Mus musculus]	56	11	3
Q8VEH6-1	COBW domain-containing protein 1 [OS=Mus musculus]	9	2	2
Q8VEH8	Endoplasmic reticulum lectin 1 [OS=Mus musculus]	6	2	2
Q8VEJ9	Vacuolar protein sorting-associated protein 4A [OS=Mus musculus]	13	5	2
Q8VEK0	Cell cycle control protein 50A [OS=Mus musculus]	11	3	3
Q8VEK3	Heterogeneous nuclear ribonucleoprotein U [OS=Mus musculus]	40	22	22
Q8VEM8	Phosphate carrier protein, mitochondrial [OS=Mus musculus]	15	4	4
Q8VH51-1	RNA-binding protein 39 [OS=Mus musculus]	20	8	8
Q8VHE0	Translocation protein SEC63 homolog [OS=Mus musculus]	30	15	15
Q8VHI3	GDP-fucose protein O-fucosyltransferase 2 [OS=Mus musculus]	13	4	4
Q8VHK9	ATP-dependent RNA helicase DHX36 [OS=Mus musculus]	3	2	2
Q8VHN8-2	Isoform 2 of Tudor-interacting repair regulator protein [OS=Mus musculus]	12	2	2
Q8VHR5	Transcriptional repressor p66-beta [OS=Mus musculus]	21	8	8
Q8VHZ7	U3 small nucleolar ribonucleoprotein protein IMP4 [OS=Mus musculus]	10	2	2
Q8VI36	Paxillin [OS=Mus musculus]	34	10	10
Q8VI75	Importin-4 [OS=Mus musculus]	23	19	19
Q8VI93	2'-5'-oligoadenylate synthase 3 [OS=Mus musculus]	42	26	26
Q8VI94	2'-5'-oligoadenylate synthase-like protein 1 [OS=Mus musculus]	39	13	13
Q8VIB3	Type 2 lactosamine alpha-2,3-sialyltransferase [OS=Mus musculus]	7	2	2
Q8VIJ6	splicing factor, proline- and glutamine-rich [OS=Mus musculus]	29	13	13
Q8WTY4	Anamorsin [OS=Mus musculus]	56	10	10
Q91V12-1	cytosolic acyl coenzyme A thioester hydrolase [OS=Mus musculus]	6	2	2
Q91V41	Ras-related protein Rab-14 [OS=Mus musculus]	81	17	15
Q91V61	Sideroflexin-3 [OS=Mus musculus]	45	9	7

Q91V64	Isochorismatase domain-containing protein 1 [OS=Mus musculus]	15	3	3
Q91V81-2	Isoform 2 of RNA-binding protein 42 [OS=Mus musculus]	6	2	2
Q91V92	ATP-citrate synthase [OS=Mus musculus]	37	28	28
Q91VA6	Polymerase delta-interacting protein 2 [OS=Mus musculus]	28	7	7
Q91VC3	Eukaryotic initiation factor 4A-III [OS=Mus musculus]	13	4	3
Q91VD9	NADH-ubiquinone oxidoreductase 75 kDa subunit, mitochondrial [OS=Mus musculus]	54	26	26
Q91VE6	MKI67 FHA domain-interacting nucleolar phosphoprotein [OS=Mus musculus]	26	5	5
Q91VH2	Sorting nexin-9 [OS=Mus musculus]	37	17	17
Q91VH6	Protein MEMO1 [OS=Mus musculus]	37	6	6
Q91VI7	Ribonuclease inhibitor [OS=Mus musculus]	89	27	27
Q91VK1	Basic leucine zipper and W2 domain-containing protein 2 [OS=Mus musculus]	26	8	6
Q91VK4	Integral membrane protein 2C [OS=Mus musculus]	23	3	3
Q91VL8	Telomeric repeat-binding factor 2-interacting protein 1 [OS=Mus musculus]	11	2	2
Q91VM5	RNA binding motif protein, X-linked-like-1 [OS=Mus musculus]	11	4	4
Q91VM9	Inorganic pyrophosphatase 2, mitochondrial [OS=Mus musculus]	35	8	8
Q91VN4	MICOS complex subunit mic25 [OS=Mus musculus]	16	3	3
Q91VN6	Probable ATP-dependent RNA helicase DDX41 [OS=Mus musculus]	9	3	3
Q91VR2	ATP synthase subunit gamma, mitochondrial [OS=Mus musculus]	43	10	10
Q91VR5	ATP-dependent RNA helicase DDX1 [OS=Mus musculus]	44	25	25
Q91VT4	Carbonyl reductase family member 4 [OS=Mus musculus]	11	2	2
Q91VU0	Protein FAM3C [OS=Mus musculus]	20	3	3
Q91VW3	SH3 domain-binding glutamic acid-rich-like protein 3 [OS=Mus musculus]	57	4	4
Q91VW5	Golgin subfamily A member 4 [OS=Mus musculus]	1	2	2
Q91VX2	Ubiquitin-associated protein 2 [OS=Mus musculus]	11	7	7
Q91VY9	Zinc finger protein 622 [OS=Mus musculus]	11	3	3
Q91VZ6	Stromal membrane-associated protein 1 [OS=Mus musculus]	6	2	2
Q91W34-1	RUS1 family protein C16orf58 homolog [OS=Mus musculus]	5	2	2
Q91W50	cold shock domain-containing protein E1 [OS=Mus musculus]	25	13	13
Q91W53	Golgin subfamily A member 7 [OS=Mus musculus]	26	3	3

Q91W59-1	RNA-binding motif, single-stranded-interacting protein 1 [OS=Mus musculus]	13	2	2
Q91W90	Thioredoxin domain-containing protein 5 [OS=Mus musculus]	61	18	18
Q91WC0	Histone-lysine N-methyltransferase setd3 [OS=Mus musculus]	12	5	5
Q91WD5	NADH dehydrogenase [ubiquinone] iron-sulfur protein 2, mitochondrial [OS=Mus musculus]	44	14	14
Q91WE2	Protein FAM192A [OS=Mus musculus]	10	2	2
Q91WJ8-2	Isoform 2 of Far upstream element-binding protein 1 [OS=Mus musculus]	31	15	14
Q91WK2	Eukaryotic translation initiation factor 3 subunit H [OS=Mus musculus]	38	8	8
Q91WK5	Glycine cleavage system H protein, mitochondrial [OS=Mus musculus]	18	2	2
Q91WM2	Haloacid dehalogenase-like hydrolase domain-containing 5 [OS=Mus musculus]	10	3	3
Q91WN1	dnaJ homolog subfamily C member 9 [OS=Mus musculus]	19	3	3
Q91WQ3	Tyrosine--tRNA ligase, cytoplasmic [OS=Mus musculus]	58	24	24
Q91WR3-3	Isoform 3 of Activating signal cointegrator 1 complex subunit 2 [OS=Mus musculus]	6	3	3
Q91WS0	CDGSH iron-sulfur domain-containing protein 1 [OS=Mus musculus]	43	5	5
Q91WU5	Arsenite methyltransferase [OS=Mus musculus]	10	3	3
Q91X20	Set1/Ash2 histone methyltransferase complex subunit ASH2 [OS=Mus musculus]	19	7	7
Q91X21-2	Isoform 2 of Uncharacterized protein KIAA2013 [OS=Mus musculus]	15	5	5
Q91X78	erlin-1 [OS=Mus musculus]	20	5	3
Q91XB0	Three-prime repair exonuclease 1 [OS=Mus musculus]	25	6	6
Q91XC9	Peroxisomal membrane protein PEX16 [OS=Mus musculus]	11	2	2
Q91XI1	tRNA-dihydrouridine(47) synthase [NAD(P)(+)]-like [OS=Mus musculus]	7	3	3
Q91XR9-1	Phospholipid hydroperoxide glutathione peroxidase, nuclear [OS=Mus musculus]	10	2	2
Q91XU3	Phosphatidylinositol 5-phosphate 4-kinase type-2 gamma [OS=Mus musculus]	9	3	3
Q91XV3	Brain acid soluble protein 1 [OS=Mus musculus]	66	8	8
Q91YD3	mRNA-decapping enzyme 1A [OS=Mus musculus]	12	5	5
Q91YE6	Importin-9 [OS=Mus musculus]	16	9	9
Q91YE7	RNA-binding protein 5 [OS=Mus musculus]	5	2	2
Q91YH5-1	Atlastin-3 [OS=Mus musculus]	56	17	16

Q91YI0	argininosuccinate lyase [OS=Mus musculus]	14	5	5
Q91YJ2	sorting nexin-4 [OS=Mus musculus]	11	4	4
Q91YJ3	Thymocyte nuclear protein 1 [OS=Mus musculus]	14	3	3
Q91YK2	Ribosomal RNA processing protein 1 homolog B [OS=Mus musculus]	16	6	6
Q91YL2	E3 ubiquitin-protein ligase RNF126 [OS=Mus musculus]	12	2	2
Q91YM4	Protein TBRG4 [OS=Mus musculus]	36	14	14
Q91YN9	BAG family molecular chaperone regulator 2 [OS=Mus musculus]	50	7	7
Q91YP0	L-2-hydroxyglutarate dehydrogenase, mitochondrial [OS=Mus musculus]	8	3	3
Q91YP2	Neurolysin, mitochondrial [OS=Mus musculus]	19	9	9
Q91YP3	deoxyribose-phosphate aldolase [OS=Mus musculus]	34	9	9
Q91YQ5	Dolichyl-diphosphooligosaccharide--protein glycosyltransferase subunit 1 [OS=Mus musculus]	52	24	24
Q91YR5	Methyltransferase-like protein 13 [OS=Mus musculus]	11	4	4
Q91YR7	Pre-mRNA-processing factor 6 [OS=Mus musculus]	19	13	13
Q91YR9	Prostaglandin reductase 1 [OS=Mus musculus]	14	3	3
Q91YS8	calcium/calmodulin-dependent protein kinase type 1 [OS=Mus musculus]	41	10	10
Q91YT0	NADH dehydrogenase [ubiquinone] flavoprotein 1, mitochondrial [OS=Mus musculus]	51	16	16
Q91YT7	YTH domain-containing family protein 2 [OS=Mus musculus]	12	6	5
Q91YU8	suppressor of SWI4 1 homolog [OS=Mus musculus]	6	2	2
Q91YW3	DnaJ homolog subfamily C member 3 [OS=Mus musculus]	57	20	20
Q91YX0	protein THEMIS2 [OS=Mus musculus]	10	3	3
Q91YY4	ATP synthase mitochondrial F1 complex assembly factor 2 [OS=Mus musculus]	19	3	3
Q91Z38	tetratricopeptide repeat protein 1 [OS=Mus musculus]	24	4	4
Q91Z53	Glyoxylate reductase/hydroxypyruvate reductase [OS=Mus musculus]	33	6	6
Q91Z67	SLIT-ROBO Rho GTPase-activating protein 2 [OS=Mus musculus]	9	6	6
Q91ZA3	Propionyl-CoA carboxylase alpha chain, mitochondrial [OS=Mus musculus]	36	18	18
Q91ZE0	Trimethyllysine dioxygenase, mitochondrial [OS=Mus musculus]	16	5	5
Q91ZJ5-2	Isoform 2 of UTP--glucose-1-phosphate uridylyltransferase [OS=Mus musculus]	30	9	9
Q91ZR1	Ras-related protein Rab-4B [OS=Mus musculus]	54	8	7
Q91ZV0	Melanoma inhibitory activity protein 2 [OS=Mus musculus]	12	12	12

Q91ZW2	GDP-fucose protein O-fucosyltransferase 1 [OS=Mus musculus]	24	5	5
Q91ZX7	prolow-density lipoprotein receptor-related protein 1 [OS=Mus musculus]	8	21	21
Q920A5	Retinoid-inducible serine carboxypeptidase [OS=Mus musculus]	14	5	5
Q920A7-1	Afg3-like protein 1 [OS=Mus musculus]	13	7	7
Q920E5	Farnesyl pyrophosphate synthase [OS=Mus musculus]	71	17	17
Q920Q8-1	Influenza virus NS1A-binding protein homolog [OS=Mus musculus]	12	3	3
Q921C5-2	Isoform 2 of Protein bicaudal D homolog 2 [OS=Mus musculus]	14	7	7
Q921E2	ras-related protein rab-31 [OS=Mus musculus]	52	7	7
Q921E6-2	Isoform 2 of Polycomb protein EED [OS=Mus musculus]	5	2	2
Q921F2	TAR DNA-binding protein 43 [OS=Mus musculus]	45	12	12
Q921F4-1	Heterogeneous nuclear ribonucleoprotein L-like [OS=Mus musculus]	22	8	8
Q921G6	Leucine-rich repeat and calponin homology domain-containing protein 4 [OS=Mus musculus]	31	11	11
Q921G7	Electron transfer flavoprotein-ubiquinone oxidoreductase, mitochondrial [OS=Mus musculus]	23	10	10
Q921G8	Gamma-tubulin complex component 2 [OS=Mus musculus]	8	5	5
Q921H8	3-ketoacyl-CoA thiolase A, peroxisomal [OS=Mus musculus]	69	22	22
Q921H9	Cytochrome c oxidase assembly factor 7 [OS=Mus musculus]	10	2	2
Q921I2	kelch domain-containing protein 4 [OS=Mus musculus]	5	2	2
Q921I9	Exosome complex component Rrp41 [OS=Mus musculus]	29	5	5
Q921J4	ubiquitin-conjugating enzyme E2 S [OS=Mus musculus]	19	3	3
Q921L3	Calcium load-activated calcium channel [OS=Mus musculus]	20	3	3
Q921M4-1	Golgin subfamily A member 2 [OS=Mus musculus]	16	9	9
Q921M7	protein FAM49B [OS=Mus musculus]	64	14	14
Q921S7	39S ribosomal protein L37, mitochondrial [OS=Mus musculus]	60	16	16
Q921T2-1	Torsin-1A-interacting protein 1 [OS=Mus musculus]	45	17	17
Q921V5	Alpha-1,6-mannosyl-glycoprotein 2-beta-N-acetylglucosaminyltransferase [OS=Mus musculus]	33	9	9
Q921W4-1	Quinone oxidoreductase-like protein 1 [OS=Mus musculus]	42	8	8
Q921X6	DNA-directed RNA polymerase III subunit RPC6 [OS=Mus musculus]	8	2	2
Q921X9	Protein disulfide-isomerase A5 [OS=Mus musculus]	20	5	5
Q921Y2	U3 small nucleolar ribonucleoprotein protein IMP3 [OS=Mus musculus]	18	2	2

Q921Z5-1	Tumor necrosis factor alpha-induced protein 8 [OS=Mus musculus]	22	2	2
Q922B1	O-acetyl-ADP-ribose deacetylase MACROD1 [OS=Mus musculus]	11	2	2
Q922B2	Aspartate--tRNA ligase, cytoplasmic [OS=Mus musculus]	11	4	4
Q922D4-1	Serine/threonine-protein phosphatase 6 regulatory subunit 3 [OS=Mus musculus]	22	13	12
Q922D8	C-1-tetrahydrofolate synthase, cytoplasmic [OS=Mus musculus]	53	30	30
Q922E4	ethanolamine-phosphate cytidylyltransferase [OS=Mus musculus]	10	2	2
Q922F4	Tubulin beta-6 chain [OS=Mus musculus]	76	25	11
Q922H2	[Pyruvate dehydrogenase (acetyl-transferring)] kinase isozyme 3, mitochondrial [OS=Mus musculus]	23	6	6
Q922H4	Mannose-1-phosphate guanyltransferase alpha [OS=Mus musculus]	23	6	6
Q922J3-1	CAP-Gly domain-containing linker protein 1 [OS=Mus musculus]	10	9	9
Q922J9-4	Isoform 4 of Fatty acyl-CoA reductase 1 [OS=Mus musculus]	14	5	5
Q922K7	Probable 28S rRNA (cytosine-C(5))-methyltransferase [OS=Mus musculus]	33	16	16
Q922L6	Negative elongation factor D [OS=Mus musculus]	12	5	5
Q922M5	cell division cycle-associated 7-like protein [OS=Mus musculus]	9	2	2
Q922P9	Putative oxidoreductase GLYR1 [OS=Mus musculus]	13	5	5
Q922Q1	Mitochondrial amidoxime reducing component 2 [OS=Mus musculus]	33	8	8
Q922Q4	Pyrroline-5-carboxylate reductase 2 [OS=Mus musculus]	41	10	8
Q922Q8	Leucine-rich repeat-containing protein 59 [OS=Mus musculus]	48	13	13
Q922Q9-1	Chitinase domain-containing protein 1 [OS=Mus musculus]	18	7	7
Q922R5-1	Serine/threonine-protein phosphatase 4 regulatory subunit 3B [OS=Mus musculus]	16	8	4
Q922R8	Protein disulfide-isomerase A6 [OS=Mus musculus]	68	21	21
Q922S4	cGMP-dependent 3',5'-cyclic phosphodiesterase [OS=Mus musculus]	27	13	13
Q922S8	Kinesin-like protein KIF2C [OS=Mus musculus]	22	10	10
Q922U1	U4/U6 small nuclear ribonucleoprotein Prp3 [OS=Mus musculus]	23	8	8
Q922U2	keratin, type II cytoskeletal 5 [OS=Mus musculus]	11	6	3
Q922V4	Pleiotropic regulator 1 [OS=Mus musculus]	7	2	2
Q922X9	Protein arginine N-methyltransferase 7 [OS=Mus musculus]	5	2	2
Q922Y1	UBX domain-containing protein 1 [OS=Mus musculus]	60	9	9
Q923A2	protein Spindly [OS=Mus musculus]	6	2	2

Q923D2	flavin reductase (NADPH) [OS=Mus musculus]	78	11	11
Q923D4	Splicing factor 3B subunit 5 [OS=Mus musculus]	59	3	3
Q923D5	WW domain-binding protein 11 [OS=Mus musculus]	6	3	3
Q923G2	DNA-directed RNA polymerases I, II, and III subunit RPABC3 [OS=Mus musculus]	61	6	6
Q923T9-1	Calcium/calmodulin-dependent protein kinase type II subunit gamma [OS=Mus musculus]	11	4	2
Q924C1	exportin-5 [OS=Mus musculus]	22	17	17
Q924D0	Reticulon-4-interacting protein 1, mitochondrial [OS=Mus musculus]	16	3	3
Q924K8	Metastasis-associated protein MTA3 [OS=Mus musculus]	12	5	2
Q924S8	sprouty-related, EVH1 domain-containing protein 1 [OS=Mus musculus]	28	8	8
Q924T2	28S ribosomal protein S2, mitochondrial [OS=Mus musculus]	32	5	5
Q924Z4	Ceramide synthase 2 [OS=Mus musculus]	5	2	2
Q925I1-1	ATPase family AAA domain-containing protein 3 [OS=Mus musculus]	31	15	15
Q925J9-1	Mediator of RNA polymerase II transcription subunit 1 [OS=Mus musculus]	5	4	4
Q93092	Transaldolase [OS=Mus musculus]	52	17	17
Q99020	Heterogeneous nuclear ribonucleoprotein A/B [OS=Mus musculus]	36	14	13
Q99J09	Methylosome protein 50 [OS=Mus musculus]	35	7	7
Q99J36	THUMP domain-containing protein 1 [OS=Mus musculus]	29	7	7
Q99J45	Nuclear receptor-binding protein [OS=Mus musculus]	13	5	5
Q99J47	Dehydrogenase/reductase SDR family member 7B [OS=Mus musculus]	27	8	8
Q99J62	replication factor C subunit 4 [OS=Mus musculus]	41	10	10
Q99J77	sialic acid synthase [OS=Mus musculus]	34	9	9
Q99J83	Autophagy protein 5 [OS=Mus musculus]	12	2	2
Q99J95-1	Cyclin-dependent kinase 9 [OS=Mus musculus]	24	7	6
Q99J99	3-mercaptopyruvate sulfurtransferase [OS=Mus musculus]	22	4	4
Q99JB2	Stomatin-like protein 2, mitochondrial [OS=Mus musculus]	50	11	11
Q99JF5	Diphosphomevalonate decarboxylase [OS=Mus musculus]	18	3	3
Q99JF8	PC4 and SFRS1-interacting protein [OS=Mus musculus]	23	8	8

Q99JI4	26S proteasome non-ATPase regulatory subunit 6 [OS=Mus musculus]	26	10	10
Q99JI6	Ras-related protein Rap-1b [OS=Mus musculus]	83	15	5
Q99JR1	Sideroflexin-1 [OS=Mus musculus]	51	11	9
Q99JR8-1	SWI/SNF-related matrix-associated actin-dependent regulator of chromatin subfamily D member 2 [OS=Mus musculus]	21	7	7
Q99JT1	Glutamyl-tRNA(Gln) amidotransferase subunit B, mitochondrial [OS=Mus musculus]	12	4	4
Q99JT9	1,2-dihydroxy-3-keto-5-methylthiopentene dioxygenase [OS=Mus musculus]	46	6	6
Q99JW2	Aminoacylase-1 [OS=Mus musculus]	10	3	3
Q99JW4	LIM and senescent cell antigen-like-containing domain protein 1 [OS=Mus musculus]	17	4	4
Q99JX3	Golgi reassembly-stacking protein 2 [OS=Mus musculus]	25	6	6
Q99JX4	Eukaryotic translation initiation factor 3 subunit M [OS=Mus musculus]	64	18	18
Q99JX7	nuclear RNA export factor 1 [OS=Mus musculus]	17	6	6
Q99JY0	Trifunctional enzyme subunit beta, mitochondrial [OS=Mus musculus]	51	19	19
Q99JY4	traB domain-containing protein [OS=Mus musculus]	32	8	8
Q99JY9	actin-related protein 3 [OS=Mus musculus]	69	21	21
Q99K01-4	Isoform 4 of Pyridoxal-dependent decarboxylase domain-containing protein 1 [OS=Mus musculus]	40	20	20
Q99K23	Ufm1-specific protease 2 [OS=Mus musculus]	32	9	9
Q99K28-2	Isoform 2 of ADP-ribosylation factor GTPase-activating protein 2 [OS=Mus musculus]	14	5	5
Q99K43	Protein regulator of cytokinesis 1 [OS=Mus musculus]	8	3	2
Q99K48	Non-POU domain-containing octamer-binding protein [OS=Mus musculus]	47	13	13
Q99K51	Plastin-3 [OS=Mus musculus]	65	32	25
Q99K70	ras-related GTP-binding protein C [OS=Mus musculus]	12	5	5
Q99K74-1	Mediator of RNA polymerase II transcription subunit 24 [OS=Mus musculus]	8	5	5
Q99K82-2	Isoform 2 of Spermine oxidase [OS=Mus musculus]	10	3	3
Q99K85	phosphoserine aminotransferase [OS=Mus musculus]	68	19	19
Q99K95	protein RTF2 homolog [OS=Mus musculus]	10	2	2

Q99KB8	Hydroxyacylglutathione hydrolase, mitochondrial [OS=Mus musculus]	16	3	3
Q99KC8	von Willebrand factor A domain-containing protein 5A [OS=Mus musculus]	36	21	21
Q99KD5	Protein unc-45 homolog A [OS=Mus musculus]	7	4	4
Q99KE1	NAD-dependent malic enzyme, mitochondrial [OS=Mus musculus]	72	25	25
Q99KF1	Transmembrane emp24 domain-containing protein 9 [OS=Mus musculus]	53	13	12
Q99KG3-2	Isoform 2 of RNA-binding protein 10 [OS=Mus musculus]	11	8	8
Q99KH8	Serine/threonine-protein kinase 24 [OS=Mus musculus]	44	12	12
Q99KI0	Aconitate hydratase, mitochondrial [OS=Mus musculus]	59	36	36
Q99KI3	ER membrane protein complex subunit 3 [OS=Mus musculus]	25	4	4
Q99KJ8	dynactin subunit 2 [OS=Mus musculus]	49	13	13
Q99KK2	N-acylneuraminate cytidylyltransferase [OS=Mus musculus]	6	2	2
Q99KK7	dipeptidyl peptidase 3 [OS=Mus musculus]	43	18	18
Q99KK9	probable histidine--tRNA ligase, mitochondrial [OS=Mus musculus]	18	8	6
Q99KN2	Probable cytosolic iron-sulfur protein assembly protein Ciao1 [OS=Mus musculus]	16	3	3
Q99KN9-1	Clathrin interactor 1 [OS=Mus musculus]	26	12	12
Q99KP3	Lambda-crystallin homolog [OS=Mus musculus]	35	7	7
Q99KP6-2	Isoform 2 of Pre-mRNA-processing factor 19 [OS=Mus musculus]	43	11	11
Q99KQ4	nicotinamide phosphoribosyltransferase [OS=Mus musculus]	48	16	16
Q99KR3	Endoribonuclease LACTB2 [OS=Mus musculus]	17	4	4
Q99KV1	DnaJ homolog subfamily B member 11 [OS=Mus musculus]	37	11	11
Q99L04	Dehydrogenase/reductase SDR family member 1 [OS=Mus musculus]	50	11	11
Q99L13	3-hydroxyisobutyrate dehydrogenase, mitochondrial [OS=Mus musculus]	46	13	13
Q99L27	GMP reductase 2 [OS=Mus musculus]	13	3	3
Q99L45	Eukaryotic translation initiation factor 2 subunit 2 [OS=Mus musculus]	54	16	16
Q99L47	Hsc70-interacting protein [OS=Mus musculus]	24	8	8

Q99LB2	dehydrogenase/reductase sdr family member 4 [OS=Mus musculus]	19	5	5
Q99LB6-1	Methionine adenosyltransferase 2 subunit beta [OS=Mus musculus]	39	7	7
Q99LC2	Cleavage stimulation factor subunit 1 [OS=Mus musculus]	26	7	7
Q99LC3	NADH dehydrogenase [ubiquinone] 1 alpha subcomplex subunit 10, mitochondrial [OS=Mus musculus]	60	14	14
Q99LC5	Electron transfer flavoprotein subunit alpha, mitochondrial [OS=Mus musculus]	61	15	15
Q99LC8	Translation initiation factor eIF-2B subunit alpha [OS=Mus musculus]	22	5	5
Q99LD4-2	Isoform 2 of COP9 signalosome complex subunit 1 [OS=Mus musculus]	26	8	8
Q99LD9	Translation initiation factor eIF-2B subunit beta [OS=Mus musculus]	34	6	6
Q99LE6	ATP-binding cassette sub-family F member 2 [OS=Mus musculus]	27	13	13
Q99LF4	tRNA-splicing ligase RtcB homolog [OS=Mus musculus]	50	19	19
Q99LG2	Transportin-2 [OS=Mus musculus]	32	17	11
Q99LH1	Nucleolar GTP-binding protein 2 [OS=Mus musculus]	11	5	5
Q99LI2	chloride channel CLIC-like protein 1 [OS=Mus musculus]	25	9	9
Q99LI7	Cleavage stimulation factor subunit 3 [OS=Mus musculus]	17	7	7
Q99LJ0	CTTNBP2 N-terminal-like protein [OS=Mus musculus]	17	6	6
Q99LJ1	tissue alpha-L-fucosidase [OS=Mus musculus]	6	2	2
Q99LL5	Periodic tryptophan protein 1 homolog [OS=Mus musculus]	19	5	5
Q99LM2	CDK5 regulatory subunit-associated protein 3 [OS=Mus musculus]	27	9	9
Q99LP6	GrpE protein homolog 1, mitochondrial [OS=Mus musculus]	61	12	12
Q99LQ7	transmembrane protein 189 [OS=Mus musculus]	13	2	2
Q99LR1	Monoacylglycerol lipase ABHD12 [OS=Mus musculus]	40	10	10
Q99LS3	phosphoserine phosphatase [OS=Mus musculus]	29	4	4
Q99LT0	Protein dpy-30 homolog [OS=Mus musculus]	36	2	2
Q99LX0	protein/nucleic acid deglycase DJ-1 [OS=Mus musculus]	70	9	9
Q99LY9	NADH dehydrogenase [ubiquinone] iron-sulfur protein 5 [OS=Mus musculus]	25	3	3
Q99M04	Lipoyl synthase, mitochondrial [OS=Mus musculus]	20	4	4
Q99M15	Proline-serine-threonine phosphatase-interacting protein 2 [OS=Mus musculus]	12	3	3
Q99M31-2	Isoform 2 of Heat shock 70 kDa protein 14 [OS=Mus musculus]	36	12	12

Q99M87-1	DnaJ homolog subfamily A member 3, mitochondrial [OS=Mus musculus]	24	9	9
Q99MD9-1	Nuclear autoantigenic sperm protein [OS=Mus musculus]	33	17	17
Q99ME9	Nucleolar GTP-binding protein 1 [OS=Mus musculus]	14	7	7
Q99MI1	ELKS/Rab6-interacting/CAST family member 1 [OS=Mus musculus]	4	3	3
Q99MK8	Beta-adrenergic receptor kinase 1 [OS=Mus musculus]	27	13	13
Q99MN1	Lysine--tRNA ligase [OS=Mus musculus]	53	25	25
Q99MN9	propionyl-CoA carboxylase beta chain, mitochondrial [OS=Mus musculus]	33	11	11
Q99MR6-1	serrate RNA effector molecule homolog [OS=Mus musculus]	31	22	22
Q99MR8	Methylcrotonoyl-CoA carboxylase subunit alpha, mitochondrial [OS=Mus musculus]	5	2	2
Q99MU3	Double-stranded RNA-specific adenosine deaminase [OS=Mus musculus]	11	9	9
Q99MZ7	peroxisomal trans-2-enoyl-CoA reductase [OS=Mus musculus]	8	2	2
Q99N69	Leupaxin [OS=Mus musculus]	30	8	8
Q99N84	28S ribosomal protein S18b, mitochondrial [OS=Mus musculus]	21	2	2
Q99N85	28S ribosomal protein S18a, mitochondrial [OS=Mus musculus]	29	3	3
Q99N87	28S ribosomal protein S5, mitochondrial [OS=Mus musculus]	16	5	5
Q99N89	39S ribosomal protein L43, mitochondrial [OS=Mus musculus]	13	2	2
Q99N93	39S ribosomal protein L16, mitochondrial [OS=Mus musculus]	18	3	3
Q99N94	39S ribosomal protein L9, mitochondrial [OS=Mus musculus]	24	5	5
Q99N95	39S ribosomal protein L3, mitochondrial [OS=Mus musculus]	21	4	4
Q99N96	39S ribosomal protein L1, mitochondrial [OS=Mus musculus]	34	7	7
Q99NB8	Ubiquilin-4 [OS=Mus musculus]	28	8	7
Q99NB9	splicing factor 3B subunit 1 [OS=Mus musculus]	55	48	48
Q99NH0-1	Ankyrin repeat domain-containing protein 17 [OS=Mus musculus]	4	6	6
Q99P31	Hsp70-binding protein 1 [OS=Mus musculus]	75	18	18
Q99P72-2	Reticulon-4 [OS=Mus musculus]	47	25	25
Q99P88	nuclear pore complex protein nup155 [OS=Mus musculus]	14	13	13
Q99P91	Transmembrane glycoprotein NMB [OS=Mus musculus]	9	4	4
Q99PG2-1	opioid growth factor receptor [OS=Mus musculus]	18	6	6

Q99PL5-1	Ribosome-binding protein 1 [OS=Mus musculus]	25	31	31
Q99PL6	UBX domain-containing protein 6 [OS=Mus musculus]	12	3	3
Q99PM9	Uridine-cytidine kinase 2 [OS=Mus musculus]	72	10	10
Q99PP7	E3 ubiquitin-protein ligase TRIM33 [OS=Mus musculus]	6	5	5
Q99PT1	rho GDP-dissociation inhibitor 1 [OS=Mus musculus]	68	11	11
Q99PU8-3	Isoform 3 of Putative ATP-dependent RNA helicase DHX30 [OS=Mus musculus]	9	8	8
Q99PV0	Pre-mRNA-processing-splicing factor 8 [OS=Mus musculus]	4	5	5
Q99PW4	TP53-regulating kinase [OS=Mus musculus]	15	2	2
Q9BCZ4	selenoprotein S [OS=Mus musculus]	30	3	3
Q9CPP0	Nucleoplasmin-3 [OS=Mus musculus]	30	3	3
Q9CPP6	NADH dehydrogenase [ubiquinone] 1 alpha subcomplex subunit 5 [OS=Mus musculus]	70	7	7
Q9CPQ1	Cytochrome c oxidase subunit 6C [OS=Mus musculus]	42	4	4
Q9CPQ3	Mitochondrial import receptor subunit TOM22 homolog [OS=Mus musculus]	65	5	5
Q9CPQ8	ATP synthase subunit g, mitochondrial [OS=Mus musculus]	34	3	3
Q9CPR4	60S ribosomal protein L17 [OS=Mus musculus]	49	11	11
Q9CPR5	39S ribosomal protein L15, mitochondrial [OS=Mus musculus]	39	8	8
Q9CPS7	RNA-binding protein pno1 [OS=Mus musculus]	23	4	4
Q9CPT4	Myeloid-derived growth factor [OS=Mus musculus]	22	3	3
Q9CPT5	nucleolar protein 16 [OS=Mus musculus]	19	3	3
Q9CPU0	lactoylglutathione lyase [OS=Mus musculus]	54	7	7
Q9CPU4	Microsomal glutathione S-transferase 3 [OS=Mus musculus]	34	3	3
Q9CPV4-1	Glyoxalase domain-containing protein 4 [OS=Mus musculus]	43	9	9
Q9CPW4	Actin-related protein 2/3 complex subunit 5 [OS=Mus musculus]	65	7	7
Q9CPX6	ubiquitin-like-conjugating enzyme ATG3 [OS=Mus musculus]	16	4	4
Q9CPX7	28S ribosomal protein S16, mitochondrial [OS=Mus musculus]	34	3	3
Q9CPY7-1	cytosol aminopeptidase [OS=Mus musculus]	70	22	22
Q9CQ01	Ribonuclease T2 [OS=Mus musculus]	15	4	4
Q9CQ02	COMM domain-containing protein 4 [OS=Mus musculus]	18	2	2
Q9CQ06	39S ribosomal protein L24, mitochondrial [OS=Mus musculus]	25	4	4
Q9CQ10	Charged multivesicular body protein 3 [OS=Mus musculus]	9	2	2
Q9CQ18	Ribonuclease H2 subunit C [OS=Mus musculus]	27	2	2
Q9CQ22	ragulator complex protein LAMTOR1 [OS=Mus musculus]	75	9	9
Q9CQ37	Ubiquitin-conjugating enzyme E2 T [OS=Mus musculus]	29	5	4

Q9CQ40	39S ribosomal protein L49, mitochondrial [OS=Mus musculus]	45	4	4
Q9CQ48	NudC domain-containing protein 2 [OS=Mus musculus]	52	5	5
Q9CQ49	Nuclear cap-binding protein subunit 2 [OS=Mus musculus]	18	2	2
Q9CQ54	NADH dehydrogenase [ubiquinone] 1 subunit C2 [OS=Mus musculus]	28	3	3
Q9CQ56	Vesicle transport protein USE1 [OS=Mus musculus]	9	2	2
Q9CQ60	6-phosphogluconolactonase [OS=Mus musculus]	79	11	11
Q9CQ65	S-methyl-5'-thioadenosine phosphorylase [OS=Mus musculus]	47	8	8
Q9CQ69	Cytochrome b-c1 complex subunit 8 [OS=Mus musculus]	56	4	4
Q9CQ71	Replication protein A 14 kDa subunit [OS=Mus musculus]	61	5	5
Q9CQ80	Vacuolar protein-sorting-associated protein 25 [OS=Mus musculus]	52	7	7
Q9CQ89	Protein CutA [OS=Mus musculus]	18	2	2
Q9CQ92	mitochondrial fission 1 protein [OS=Mus musculus]	36	5	5
Q9CQA1	Trafficking protein particle complex subunit 5 [OS=Mus musculus]	16	2	2
Q9CQA3	Succinate dehydrogenase [ubiquinone] iron-sulfur subunit, mitochondrial [OS=Mus musculus]	37	10	10
Q9CQA6	Coiled-coil-helix-coiled-coil-helix domain-containing protein 1 [OS=Mus musculus]	40	3	3
Q9CQB5	CDGSH iron-sulfur domain-containing protein 2 [OS=Mus musculus]	43	5	5
Q9CQC6	Basic leucine zipper and W2 domain-containing protein 1 [OS=Mus musculus]	58	16	14
Q9CQC7	NADH dehydrogenase [ubiquinone] 1 beta subcomplex subunit 4 [OS=Mus musculus]	47	5	5
Q9CQC8-1	Maspardin [OS=Mus musculus]	20	4	4
Q9CQC9	GTP-binding protein SAR1b [OS=Mus musculus]	74	10	9
Q9CQD1	Ras-related protein Rab-5A [OS=Mus musculus]	48	8	5
Q9CQE1	Protein NipSnap homolog 3B [OS=Mus musculus]	24	4	4
Q9CQE3	28S ribosomal protein S17, mitochondrial [OS=Mus musculus]	55	5	5
Q9CQE5	Regulator of G-protein signaling 10 [OS=Mus musculus]	25	3	3
Q9CQE7-2	Isoform 2 of Endoplasmic reticulum-Golgi intermediate compartment protein 3 [OS=Mus musculus]	14	3	3
Q9CQE8	UPF0568 protein C14orf166 homolog [OS=Mus musculus]	47	9	9
Q9CQF0	39S ribosomal protein L11, mitochondrial [OS=Mus musculus]	28	4	4
Q9CQF3	Cleavage and polyadenylation specificity factor subunit 5 [OS=Mus musculus]	49	9	9
Q9CQF9	Prenylcysteine oxidase [OS=Mus musculus]	25	8	8
Q9CQG2	U6 small nuclear RNA (adenine-(43)-N(6))-methyltransferase [OS=Mus musculus]	19	7	7

Q9CQH3	NADH dehydrogenase [ubiquinone] 1 beta subcomplex subunit 5, mitochondrial [OS=Mus musculus]	32	5	5
Q9CQH7	transcription factor BTF3 homolog 4 [OS=Mus musculus]	37	2	2
Q9CQI3	glia maturation factor beta [OS=Mus musculus]	37	4	4
Q9CQI6	coactosin-like protein [OS=Mus musculus]	51	6	6
Q9CQI7	U2 small nuclear ribonucleoprotein B'' [OS=Mus musculus]	26	4	3
Q9CQJ4	E3 ubiquitin-protein ligase RING2 [OS=Mus musculus]	13	3	2
Q9CQJ6	density-regulated protein [OS=Mus musculus]	19	3	3
Q9CQJ8	NADH dehydrogenase [ubiquinone] 1 beta subcomplex subunit 9 [OS=Mus musculus]	44	5	5
Q9CQL4	39S ribosomal protein L20, mitochondrial [OS=Mus musculus]	28	3	3
Q9CQL5	39S ribosomal protein L18, mitochondrial [OS=Mus musculus]	18	3	3
Q9CQM5	Thioredoxin domain-containing protein 17 [OS=Mus musculus]	33	3	3
Q9CQM9	glutaredoxin-3 [OS=Mus musculus]	62	15	15
Q9CQN1	heat shock protein 75 kDa, mitochondrial [OS=Mus musculus]	55	30	29
Q9CQN7	39S ribosomal protein L41, mitochondrial [OS=Mus musculus]	24	3	3
Q9CQP0	39S ribosomal protein L33, mitochondrial [OS=Mus musculus]	23	2	2
Q9CQQ4	Gem-associated protein 2 [OS=Mus musculus]	12	2	2
Q9CQQ7	ATP synthase F(0) complex subunit B1, mitochondrial [OS=Mus musculus]	51	15	15
Q9CQQ8	U6 snRNA-associated Sm-like protein LSm7 [OS=Mus musculus]	30	3	3
Q9CQR2	40S ribosomal protein S21 [OS=Mus musculus]	60	6	6
Q9CQR4	Acyl-coenzyme A thioesterase 13 [OS=Mus musculus]	25	3	3
Q9CQR6	Serine/threonine-protein phosphatase 6 catalytic subunit [OS=Mus musculus]	36	7	7
Q9CQS5	Serine/threonine-protein kinase rio2 [OS=Mus musculus]	8	3	3
Q9CQS8	protein transport protein Sec61 subunit beta [OS=Mus musculus]	36	2	2
Q9CQT1	methylthioribose-1-phosphate isomerase [OS=Mus musculus]	60	11	11
Q9CQT2	RNA-binding protein 7 [OS=Mus musculus]	25	2	2
Q9CQU0	Thioredoxin domain-containing protein 12 [OS=Mus musculus]	46	4	4
Q9CQU5	ZW10 interactor [OS=Mus musculus]	24	3	3
Q9CQV1	Mitochondrial import inner membrane translocase subunit TIM16 [OS=Mus musculus]	43	3	3
Q9CQV4-1	Reticulophagy regulator 3 [OS=Mus musculus]	11	3	3
Q9CQV5	28S ribosomal protein S24, mitochondrial [OS=Mus musculus]	28	3	3
Q9CQV7-2	Isoform 2 of Mitochondrial import inner membrane translocase subunit TIM14 [OS=Mus musculus]	21	2	2

Q9CQV8-1	14-3-3 protein beta/alpha [OS=Mus musculus]	64	15	10
Q9CQW1	Synaptobrevin homolog YKT6 [OS=Mus musculus]	34	5	5
Q9CQW2	ADP-ribosylation factor-like protein 8B [OS=Mus musculus]	56	13	5
Q9CQW9	Interferon-induced transmembrane protein 3 [OS=Mus musculus]	49	5	5
Q9CQX2	Cytochrome b5 type B [OS=Mus musculus]	58	5	5
Q9CQX4	PCNA-associated factor [OS=Mus musculus]	28	2	2
Q9CQX8	28S ribosomal protein S36, mitochondrial [OS=Mus musculus]	44	2	2
Q9CQY6	Ubiquinol-cytochrome-c reductase complex assembly factor 2 [OS=Mus musculus]	21	2	2
Q9CQZ5	NADH dehydrogenase [ubiquinone] 1 alpha subcomplex subunit 6 [OS=Mus musculus]	40	4	4
Q9CQZ6	NADH dehydrogenase [ubiquinone] 1 beta subcomplex subunit 3 [OS=Mus musculus]	28	2	2
Q9CR00	26S proteasome non-ATPase regulatory subunit 9 [OS=Mus musculus]	13	2	2
Q9CR09	ubiquitin-fold modifier-conjugating enzyme 1 [OS=Mus musculus]	40	5	5
Q9CR16	peptidyl-prolyl cis-trans isomerase D [OS=Mus musculus]	62	20	20
Q9CR26	Vacuolar protein sorting-associated protein VTA1 homolog [OS=Mus musculus]	20	4	4
Q9CR51	V-type proton ATPase subunit G 1 [OS=Mus musculus]	40	4	4
Q9CR57	60S ribosomal protein L14 [OS=Mus musculus]	22	4	4
Q9CR61	NADH dehydrogenase [ubiquinone] 1 beta subcomplex subunit 7 [OS=Mus musculus]	47	5	5
Q9CR62	Mitochondrial 2-oxoglutarate/malate carrier protein [OS=Mus musculus]	52	13	13
Q9CR64-1	Protein kish-A [OS=Mus musculus]	28	3	3
Q9CR67	Transmembrane protein 33 [OS=Mus musculus]	27	6	6
Q9CR68	cytochrome b-c1 complex subunit Rieske, mitochondrial [OS=Mus musculus]	44	8	8
Q9CR70	EKC/KEOPS complex subunit LAGE3 [OS=Mus musculus]	33	2	2
Q9CR88	28S ribosomal protein S14, mitochondrial [OS=Mus musculus]	22	2	2
Q9CR98	Protein FAM136A [OS=Mus musculus]	36	3	3
Q9CRA4	Methylsterol monooxygenase 1 [OS=Mus musculus]	18	3	3
Q9CRA5-1	Golgi phosphoprotein 3 [OS=Mus musculus]	39	8	7
Q9CRA8	Exosome complex component RRP46 [OS=Mus musculus]	38	4	4
Q9CRB2	H/ACA ribonucleoprotein complex subunit 2 [OS=Mus musculus]	35	3	3
Q9CRB3	5-hydroxyisourate hydrolase [OS=Mus musculus]	27	2	2

Q9CRB9	MICOS complex subunit MIC19 [OS=Mus musculus]	45	11	11
Q9CRC8	Leucine-rich repeat-containing protein 40 [OS=Mus musculus]	17	8	8
Q9CRD0-1	OCIA domain-containing protein 1 [OS=Mus musculus]	43	7	7
Q9CRD2	ER membrane protein complex subunit 2 [OS=Mus musculus]	54	10	10
Q9CRT8	Exportin-T [OS=Mus musculus]	18	12	12
Q9CS42	ribose-phosphate pyrophosphokinase 2 [OS=Mus musculus]	49	11	4
Q9CSH3	exosome complex exonuclease RRP44 [OS=Mus musculus]	6	4	4
Q9CSN1	SNW domain-containing protein 1 [OS=Mus musculus]	9	3	3
Q9CSU0	regulation of nuclear pre-mRNA domain-containing protein 1B [OS=Mus musculus]	46	9	7
Q9CT10	Ran-binding protein 3 [OS=Mus musculus]	18	5	5
Q9CU62	structural maintenance of chromosomes protein 1a [OS=Mus musculus]	23	22	22
Q9CVB6	Actin-related protein 2/3 complex subunit 2 [OS=Mus musculus]	71	18	18
Q9CW03	Structural maintenance of chromosomes protein 3 [OS=Mus musculus]	25	21	21
Q9CW46	Ribonucleoprotein PTB-binding 1 [OS=Mus musculus]	51	16	16
Q9CWE0	Mitochondrial fission regulator 1-like [OS=Mus musculus]	24	4	4
Q9CWI3	BRCA2 and CDKN1A-interacting protein [OS=Mus musculus]	27	6	6
Q9CWJ9	bifunctional purine biosynthesis protein purH [OS=Mus musculus]	78	34	34
Q9CWK3	CD2 antigen cytoplasmic tail-binding protein 2 [OS=Mus musculus]	7	2	2
Q9CWK8	Sorting nexin-2 [OS=Mus musculus]	44	17	15
Q9CWL8	Beta-catenin-like protein 1 [OS=Mus musculus]	12	6	6
Q9CWM4	prefoldin subunit 1 [OS=Mus musculus]	17	2	2
Q9CWN7	CCR4-NOT transcription complex subunit 11 [OS=Mus musculus]	14	4	4
Q9CWP6-1	motile sperm domain-containing protein 2 [OS=Mus musculus]	11	5	5
Q9CWT6	Probable ATP-dependent RNA helicase DDX28 [OS=Mus musculus]	6	2	2
Q9CWU9	Nucleoporin Nup37 [OS=Mus musculus]	28	6	6
Q9CWW6	Peptidyl-prolyl cis-trans isomerase NIMA-interacting 4 [OS=Mus musculus]	42	4	4
Q9CWW7	CXXC-type zinc finger protein 1 [OS=Mus musculus]	6	3	3
Q9CWX9	Probable ATP-dependent RNA helicase DDX47 [OS=Mus musculus]	9	3	3
Q9CWY8	Ribonuclease H2 subunit A [OS=Mus musculus]	32	7	7

Q9CWZ3	RNA-binding protein 8A [OS=Mus musculus]	32	4	4
Q9CWZ7	Gamma-soluble NSF attachment protein [OS=Mus musculus]	20	5	5
Q9CX00	IST1 homolog [OS=Mus musculus]	9	2	2
Q9CX34	Protein SGT1 homolog [OS=Mus musculus]	37	9	9
Q9CX56	26S proteasome non-ATPase regulatory subunit 8 [OS=Mus musculus]	39	10	10
Q9CX86	Heterogeneous nuclear ribonucleoprotein A0 [OS=Mus musculus]	35	7	7
Q9CX97	WD repeat-containing protein 55 [OS=Mus musculus]	13	2	2
Q9CXE7	Transmembrane emp24 domain-containing protein 5 [OS=Mus musculus]	33	5	5
Q9CXF4	TBC1 domain family member 15 [OS=Mus musculus]	17	11	11
Q9CXI0	2-methoxy-6-polyprenyl-1,4-benzoquinol methylase, mitochondrial [OS=Mus musculus]	11	3	3
Q9CXI5	Mesencephalic astrocyte-derived neurotrophic factor [OS=Mus musculus]	36	6	6
Q9CXK8	60S ribosome subunit biogenesis protein NIP7 homolog [OS=Mus musculus]	51	7	7
Q9CXK9-1	RNA-binding protein 33 [OS=Mus musculus]	5	3	3
Q9CXL3	uncharacterized protein C7orf50 homolog [OS=Mus musculus]	26	2	2
Q9CXR1	Dehydrogenase/reductase SDR family member 7 [OS=Mus musculus]	27	5	5
Q9CXT8	mitochondrial-processing peptidase subunit beta [OS=Mus musculus]	37	11	11
Q9CXV9	DCN1-like protein 5 [OS=Mus musculus]	19	3	3
Q9CXW2	28S ribosomal protein S22, mitochondrial [OS=Mus musculus]	33	8	8
Q9CXW3	Calcyclin-binding protein [OS=Mus musculus]	67	15	15
Q9CXW4	60S ribosomal protein L11 [OS=Mus musculus]	61	11	11
Q9CXY6	Interleukin enhancer-binding factor 2 [OS=Mus musculus]	48	10	10
Q9CXY9	GPI-anchor transamidase [OS=Mus musculus]	29	6	6
Q9CXZ1	NADH dehydrogenase [ubiquinone] iron-sulfur protein 4, mitochondrial [OS=Mus musculus]	35	5	5
Q9CY16	28S ribosomal protein S28, mitochondrial [OS=Mus musculus]	30	3	3
Q9CY21	Probable 18S rRNA (guanine-N(7))-methyltransferase [OS=Mus musculus]	25	4	4
Q9CY27	Very-long-chain enoyl-CoA reductase [OS=Mus musculus]	9	2	2
Q9CY45	EEF1A lysine methyltransferase 1 [OS=Mus musculus]	12	2	2
Q9CY50	Translocon-associated protein subunit alpha [OS=Mus musculus]	33	5	5

Q9CY52	probable tRNA(His) guanylyltransferase [OS=Mus musculus]	13	3	3
Q9CY64	Biliverdin reductase A [OS=Mus musculus]	59	16	16
Q9CY66-1	H/ACA ribonucleoprotein complex subunit 1 [OS=Mus musculus]	12	2	2
Q9CY73-1	39S ribosomal protein L44, mitochondrial [OS=Mus musculus]	30	8	8
Q9CY97-1	RNA polymerase II subunit A C-terminal domain phosphatase SSU72 [OS=Mus musculus]	29	4	4
Q9CYA0	Cysteine-rich with EGF-like domain protein 2 [OS=Mus musculus]	33	9	9
Q9CYA6	Zinc finger CCHC domain-containing protein 8 [OS=Mus musculus]	21	8	8
Q9CYC5	Kinetochore-associated protein DSN1 homolog [OS=Mus musculus]	14	2	2
Q9CYG7	Mitochondrial import receptor subunit TOM34 [OS=Mus musculus]	23	5	5
Q9CYH6	Ribosome biogenesis regulatory protein homolog [OS=Mus musculus]	7	2	2
Q9CYI0	Protein Njmu-R1 [OS=Mus musculus]	11	3	3
Q9CYI4-1	putative RNA-binding protein Luc7-like 1 [OS=Mus musculus]	13	3	2
Q9CYL5	Golgi-associated plant pathogenesis-related protein 1 [OS=Mus musculus]	56	5	5
Q9CYN2	Signal peptidase complex subunit 2 [OS=Mus musculus]	21	3	3
Q9CYN9	Renin receptor [OS=Mus musculus]	28	8	8
Q9CYR0	Single-stranded DNA-binding protein, mitochondrial [OS=Mus musculus]	28	3	3
Q9CYR6	phosphoacetylglucosamine mutase [OS=Mus musculus]	14	6	6
Q9CYW4	Haloacid dehalogenase-like hydrolase domain-containing protein 3 [OS=Mus musculus]	21	3	3
Q9CYX7	RRP15-like protein [OS=Mus musculus]	6	2	2
Q9CYZ2	Tumor protein D54 [OS=Mus musculus]	31	5	5
Q9CZ04-2	Isoform 2 of COP9 signalosome complex subunit 7a [OS=Mus musculus]	31	6	6
Q9CZ13	Cytochrome b-c1 complex subunit 1, mitochondrial [OS=Mus musculus]	54	19	19
Q9CZ15	DNA replication complex GINS protein PSF1 [OS=Mus musculus]	15	2	2
Q9CZ28	Vacuolar-sorting protein SNF8 [OS=Mus musculus]	12	2	2
Q9CZ30-1	obg-like ATPase 1 [OS=Mus musculus]	52	13	13
Q9CZ44-3	Isoform 3 of NSFL1 cofactor p47 [OS=Mus musculus]	47	11	11
Q9CZ83-2	Isoform 2 of 39S ribosomal protein L55, mitochondrial [OS=Mus musculus]	17	2	2
Q9CZB0	Succinate dehydrogenase cytochrome b560 subunit, mitochondrial [OS=Mus musculus]	38	5	5

Q9CZD3	Glycine--tRNA ligase [OS=Mus musculus]	40	22	22
Q9CZE3	Ras-related protein Rab-32 [OS=Mus musculus]	28	5	5
Q9CZH3	Proteasome assembly chaperone 3 [OS=Mus musculus]	50	4	4
Q9CZL2	Uncharacterized protein C4orf32 homolog [OS=Mus musculus]	28	2	2
Q9CZM2	60S ribosomal protein L15 [OS=Mus musculus]	23	5	5
Q9CZN8	Glutamyl-tRNA(Gln) amidotransferase subunit A, mitochondrial [OS=Mus musculus]	14	4	4
Q9CZR8	Elongation factor Ts, mitochondrial [OS=Mus musculus]	53	8	8
Q9CZS1	Aldehyde dehydrogenase X, mitochondrial [OS=Mus musculus]	23	9	8
Q9CZT4-1	DNA-directed RNA polymerase III subunit RPC5 [OS=Mus musculus]	4	2	2
Q9CZU3	Superkiller viralicidic activity 2-like 2 [OS=Mus musculus]	16	9	9
Q9CZU4	GTPase Era, mitochondrial [OS=Mus musculus]	5	2	2
Q9CZU6	citrate synthase, mitochondrial [OS=Mus musculus]	49	19	19
Q9CZW5	Mitochondrial import receptor subunit TOM70 [OS=Mus musculus]	51	20	20
Q9CZX8	40S ribosomal protein S19 [OS=Mus musculus]	35	8	8
Q9CZX9	ER membrane protein complex subunit 4 [OS=Mus musculus]	28	4	4
Q9CZY3	Ubiquitin-conjugating enzyme E2 variant 1 [OS=Mus musculus]	50	6	6
Q9D020	Cytosolic 5'-nucleotidase 3A [OS=Mus musculus]	17	4	4
Q9D023	Mitochondrial pyruvate carrier 2 [OS=Mus musculus]	23	3	3
Q9D024	Coiled-coil domain-containing protein 47 [OS=Mus musculus]	44	15	15
Q9D051	Pyruvate dehydrogenase E1 component subunit beta, mitochondrial [OS=Mus musculus]	52	13	13
Q9D071	MMS19 nucleotide excision repair protein homolog [OS=Mus musculus]	27	14	14
Q9D0B5	thiosulfate sulfurtransferase/rhodanese-like domain-containing protein 3 [OS=Mus musculus]	22	2	2
Q9D0B6-1	Protein PBDC1 [OS=Mus musculus]	41	5	5
Q9D0C4-1	tRNA (guanine(37)-N1)-methyltransferase [OS=Mus musculus]	8	3	3
Q9D0D3	Poly(A) RNA polymerase, mitochondrial [OS=Mus musculus]	24	7	7
Q9D0D4	Probable dimethyladenosine transferase [OS=Mus musculus]	31	6	6
Q9D0D5	General transcription factor IIE subunit 1 [OS=Mus musculus]	5	2	2
Q9D0E1	Heterogeneous nuclear ribonucleoprotein M [OS=Mus musculus]	57	29	29
Q9D0F3	Protein ERGIC-53 [OS=Mus musculus]	49	15	15
Q9D0F6	Replication factor C subunit 5 [OS=Mus musculus]	51	12	12

Q9D0F9	Phosphoglucomutase-1 [OS=Mus musculus]	60	22	22
Q9D0G0	28S ribosomal protein S30, mitochondrial [OS=Mus musculus]	40	13	13
Q9D0I4	Syntaxin-17 [OS=Mus musculus]	15	2	2
Q9D0I8	mRNA turnover protein 4 homolog [OS=Mus musculus]	21	5	5
Q9D0I9	arginine--tRNA ligase, cytoplasmic [OS=Mus musculus]	57	34	34
Q9D0J4	ADP-ribosylation factor-like protein 2 [OS=Mus musculus]	51	6	6
Q9D0K2	Succinyl-CoA:3-ketoacid coenzyme A transferase 1, mitochondrial [OS=Mus musculus]	55	17	17
Q9D0L7	Armadillo repeat-containing protein 10 [OS=Mus musculus]	33	6	6
Q9D0L8-1	mRNA cap guanine-N7 methyltransferase [OS=Mus musculus]	25	7	7
Q9D0M0	Exosome complex exonuclease RRP42 [OS=Mus musculus]	46	7	7
Q9D0M1	Phosphoribosyl pyrophosphate synthase-associated protein 1 [OS=Mus musculus]	24	6	5
Q9D0M3	Cytochrome c1, heme protein, mitochondrial [OS=Mus musculus]	47	9	9
Q9D0M5	Dynein light chain 2, cytoplasmic [OS=Mus musculus]	64	5	2
Q9D0Q7	39S ribosomal protein L45, mitochondrial [OS=Mus musculus]	31	6	6
Q9D0R2	Threonine--tRNA ligase, cytoplasmic [OS=Mus musculus]	41	25	25
Q9D0R4	Probable ATP-dependent RNA helicase DDX56 [OS=Mus musculus]	32	10	10
Q9D0R8	Protein LSM12 homolog [OS=Mus musculus]	28	3	3
Q9D0S9	Histidine triad nucleotide-binding protein 2, mitochondrial [OS=Mus musculus]	29	3	3
Q9D0T1	NHP2-like protein 1 [OS=Mus musculus]	60	6	6
Q9D125	28S ribosomal protein S25, mitochondrial [OS=Mus musculus]	23	4	4
Q9D136	2-oxoglutarate and iron-dependent oxygenase domain-containing protein 3 [OS=Mus musculus]	12	2	2
Q9D162-2	Isoform 2 of Coiled-coil domain-containing protein 167 [OS=Mus musculus]	25	2	2
Q9D168	integrator complex subunit 12 [OS=Mus musculus]	17	4	4
Q9D172	ES1 protein homolog, mitochondrial [OS=Mus musculus]	46	7	7
Q9D1A2	cytosolic non-specific dipeptidase [OS=Mus musculus]	73	27	27
Q9D1B9	39S ribosomal protein L28, mitochondrial [OS=Mus musculus]	45	6	6
Q9D1C1	Ubiquitin-conjugating enzyme E2 C [OS=Mus musculus]	63	6	6
Q9D1C8	vacuolar protein sorting-associated protein 28 homolog [OS=Mus musculus]	25	4	4

Q9D1C9	Ribosomal RNA-processing protein 7 homolog A [OS=Mus musculus]	18	3	3
Q9D1D4	Transmembrane emp24 domain-containing protein 10 [OS=Mus musculus]	42	9	9
Q9D1E6	tubulin-folding cofactor B [OS=Mus musculus]	38	7	7
Q9D1G1	ras-related protein Rab-1B [OS=Mus musculus]	67	9	5
Q9D1G2-1	phosphomevalonate kinase [OS=Mus musculus]	10	2	2
Q9D1H6	NADH dehydrogenase [ubiquinone] 1 alpha subcomplex assembly factor 4 [OS=Mus musculus]	24	3	3
Q9D1H7	Golgi to ER traffic protein 4 homolog [OS=Mus musculus]	9	2	2
Q9D1H8	39S ribosomal protein L53, mitochondrial [OS=Mus musculus]	64	5	5
Q9D1I6	39S ribosomal protein L14, mitochondrial [OS=Mus musculus]	20	2	2
Q9D1J1	Adaptin ear-binding coat-associated protein 2 [OS=Mus musculus]	26	4	4
Q9D1J3	SAP domain-containing ribonucleoprotein [OS=Mus musculus]	60	10	10
Q9D1K2	V-type proton ATPase subunit F [OS=Mus musculus]	70	6	6
Q9D1K7	UPF0687 protein C20orf27 homolog [OS=Mus musculus]	19	2	2
Q9D1L0	Coiled-coil-helix-coiled-coil-helix domain-containing protein 2 [OS=Mus musculus]	22	2	2
Q9D1M0	protein SEC13 homolog [OS=Mus musculus]	57	9	9
Q9D1M4	Eukaryotic translation elongation factor 1 epsilon-1 [OS=Mus musculus]	36	5	5
Q9D1M7	Peptidyl-prolyl cis-trans isomerase FKBP11 [OS=Mus musculus]	9	2	2
Q9D1N9	39S ribosomal protein L21, mitochondrial [OS=Mus musculus]	33	5	5
Q9D1P0	39S ribosomal protein L13, mitochondrial [OS=Mus musculus]	49	6	6
Q9D1P4	cysteine and histidine-rich domain-containing protein 1 [OS=Mus musculus]	15	3	3
Q9D1Q6	Endoplasmic reticulum resident protein 44 [OS=Mus musculus]	51	15	15
Q9D1R2	Protein KTI12 homolog [OS=Mus musculus]	7	2	2
Q9D1R9	60S ribosomal protein L34 [OS=Mus musculus]	13	2	2
Q9D287	Pre-mRNA-splicing factor spf27 [OS=Mus musculus]	48	6	6
Q9D2C2-1	protein saal1 [OS=Mus musculus]	35	10	10
Q9D2E2	Target of EGR1 protein 1 [OS=Mus musculus]	6	3	3
Q9D2G2-1	Dihydrolipoyllysine-residue succinyltransferase component of 2-oxoglutarate dehydrogenase complex, mitochondrial [OS=Mus musculus]	29	10	10
Q9D2N9	Vacuolar protein sorting-associated protein 33A [OS=Mus musculus]	22	7	7
Q9D2R0	acetoacetyl-CoA synthetase [OS=Mus musculus]	17	8	8

Q9D2R6	Cytochrome c oxidase assembly factor 3 homolog, mitochondrial [OS=Mus musculus]	30	3	3
Q9D2V7	Coronin-7 [OS=Mus musculus]	21	9	9
Q9D338	39S ribosomal protein L19, mitochondrial [OS=Mus musculus]	40	9	9
Q9D379	epoxide hydrolase 1 [OS=Mus musculus]	34	10	10
Q9D3D9	ATP synthase subunit delta, mitochondrial [OS=Mus musculus]	38	3	3
Q9D3P8	Plasminogen receptor (KT) [OS=Mus musculus]	22	3	3
Q9D4H1	exocyst complex component 2 [OS=Mus musculus]	3	2	2
Q9D554	splicing factor 3a subunit 3 [OS=Mus musculus]	54	18	18
Q9D5T0	ATPase family aaa domain-containing protein 1 [OS=Mus musculus]	43	11	11
Q9D5V5	Cullin-5 [OS=Mus musculus]	21	11	11
Q9D5V6	Synapse-associated protein 1 [OS=Mus musculus]	22	5	5
Q9D620	Rab11 family-interacting protein 1 [OS=Mus musculus]	25	10	10
Q9D662	protein transport protein Sec23B [OS=Mus musculus]	45	21	19
Q9D6F9	Tubulin beta-4A chain [OS=Mus musculus]	79	26	4
Q9D6J5	NADH dehydrogenase [ubiquinone] 1 beta subcomplex subunit 8, mitochondrial [OS=Mus musculus]	15	2	2
Q9D6J6	NADH dehydrogenase [ubiquinone] flavoprotein 2, mitochondrial [OS=Mus musculus]	40	8	8
Q9D6K5	Synaptojanin-2-binding protein [OS=Mus musculus]	46	4	4
Q9D6K8	FUN14 domain-containing protein 2 [OS=Mus musculus]	15	2	2
Q9D6M3	Mitochondrial glutamate carrier 1 [OS=Mus musculus]	33	6	6
Q9D6N1	Carbonic anhydrase 13 [OS=Mus musculus]	23	3	3
Q9D6N5	Dr1-associated corepressor [OS=Mus musculus]	11	3	3
Q9D6R2	Isocitrate dehydrogenase [NAD] subunit alpha, mitochondrial [OS=Mus musculus]	37	13	13
Q9D6S7	Ribosome-recycling factor, mitochondrial [OS=Mus musculus]	27	3	3
Q9D6T0-1	nitric oxide synthase-interacting protein [OS=Mus musculus]	21	3	3
Q9D6U8	Protein FAM162A [OS=Mus musculus]	34	7	7
Q9D6Y7	Mitochondrial peptide methionine sulfoxide reductase [OS=Mus musculus]	27	4	4
Q9D6Y9	1,4-alpha-glucan-branching enzyme [OS=Mus musculus]	10	5	5
Q9D6Z1	Nucleolar protein 56 [OS=Mus musculus]	44	15	15

Q9D706	RNA polymerase II-associated protein 3 [OS=Mus musculus]	20	8	8
Q9D710	Thioredoxin-related transmembrane protein 2 [OS=Mus musculus]	21	4	4
Q9D753	Exosome complex component RRP43 [OS=Mus musculus]	30	5	5
Q9D771	Transmembrane protein 206 [OS=Mus musculus]	9	2	2
Q9D773	39S ribosomal protein L2, mitochondrial [OS=Mus musculus]	19	3	3
Q9D786	HAUS augmin-like complex subunit 5 [OS=Mus musculus]	7	3	3
Q9D787	Peptidyl-prolyl cis-trans isomerase-like 2 [OS=Mus musculus]	4	2	2
Q9D7A6	Signal recognition particle 19 kDa protein [OS=Mus musculus]	27	2	2
Q9D7A8	armadillo repeat-containing protein 1 [OS=Mus musculus]	28	5	5
Q9D7B1	tRNA-dihydrouridine(20) synthase [NAD(P)+]-like [OS=Mus musculus]	9	3	3
Q9D7G0	ribose-phosphate pyrophosphokinase 1 [OS=Mus musculus]	51	1<br>1	4
Q9D7H3	RNA 3'-terminal phosphate cyclase [OS=Mus musculus]	10	3	3
Q9D7J4	Cytochrome c oxidase protein 20 homolog [OS=Mus musculus]	31	2	2
Q9D7M1	Glucose-induced degradation protein 8 homolog [OS=Mus musculus]	17	2	2
Q9D7N3	28S ribosomal protein S9, mitochondrial [OS=Mus musculus]	43	1<br>1	11
Q9D7N6	39S ribosomal protein L30, mitochondrial [OS=Mus musculus]	23	2	2
Q9D7N9	Adipocyte plasma membrane-associated protein [OS=Mus musculus]	43	9	9
Q9D7S7	60S ribosomal protein L22-like 1 [OS=Mus musculus]	39	3	3
Q9D7X3	dual specificity protein phosphatase 3 [OS=Mus musculus]	66	7	7
Q9D819	Inorganic pyrophosphatase [OS=Mus musculus]	77	1<br>8	18
Q9D824-1	Pre-mRNA 3'-end-processing factor FIP1 [OS=Mus musculus]	30	8	8
Q9D832	DnaJ homolog subfamily B member 4 [OS=Mus musculus]	20	4	3
Q9D855	Cytochrome b-c1 complex subunit 7 [OS=Mus musculus]	51	6	6
Q9D868	peptidyl-prolyl cis-trans isomerase H [OS=Mus musculus]	24	3	3
Q9D880	Mitochondrial import inner membrane translocase subunit TIM50 [OS=Mus musculus]	27	7	7
Q9D883	Splicing factor U2AF 35 kDa subunit [OS=Mus musculus]	33	8	8
Q9D892	Inosine triphosphate pyrophosphatase [OS=Mus musculus]	47	5	5
Q9D898	actin-related protein 2/3 complex subunit 5-like protein [OS=Mus musculus]	54	5	5
Q9D8B3	Charged multivesicular body protein 4b [OS=Mus musculus]	13	2	2
Q9D8C4	Interferon-induced 35 kDa protein homolog [OS=Mus musculus]	34	6	6
Q9D8E6	60S ribosomal protein L4 [OS=Mus musculus]	42	2<br>2	22
Q9D8M4	60S ribosomal protein L7-like 1 [OS=Mus musculus]	22	4	4

Q9D8N0	elongation factor 1-gamma [OS=Mus musculus]	69	24	24
Q9D8P4	39S ribosomal protein L17, mitochondrial [OS=Mus musculus]	32	3	3
Q9D8S4	Oligoribonuclease, mitochondrial [OS=Mus musculus]	27	5	5
Q9D8S9	BolA-like protein 1 [OS=Mus musculus]	39	3	3
Q9D8T2	Gasdermin-D [OS=Mus musculus]	30	8	8
Q9D8T7	SRA stem-loop-interacting RNA-binding protein, mitochondrial [OS=Mus musculus]	36	2	2
Q9D8U8	sorting nexin-5 [OS=Mus musculus]	59	21	21
Q9D8V7	Signal peptidase complex catalytic subunit SEC11C [OS=Mus musculus]	19	4	4
Q9D8W5	26s proteasome non-atpase regulatory subunit 12 [OS=Mus musculus]	51	19	19
Q9D8Y0	EF-hand domain-containing protein D2 [OS=Mus musculus]	54	12	12
Q9D8Y1	Transmembrane protein 126A [OS=Mus musculus]	17	2	2
Q9D8Y7	Tumor necrosis factor alpha-induced protein 8-like protein 2 [OS=Mus musculus]	40	4	4
Q9D902	General transcription factor IIE subunit 2 [OS=Mus musculus]	23	4	4
Q9D903	Probable rRNA-processing protein EBP2 [OS=Mus musculus]	8	2	2
Q9D906	Ubiquitin-like modifier-activating enzyme ATG7 [OS=Mus musculus]	20	10	10
Q9D945	Protein LLP homolog [OS=Mus musculus]	29	2	2
Q9D964	glycine amidinotransferase, mitochondrial [OS=Mus musculus]	61	17	17
Q9D967	Magnesium-dependent phosphatase 1 [OS=Mus musculus]	18	3	3
Q9D975	sulfiredoxin-1 [OS=Mus musculus]	65	3	3
Q9D9V3-1	Ethylmalonyl-CoA decarboxylase [OS=Mus musculus]	22	4	4
Q9DAK9	14 kDa phosphohistidine phosphatase [OS=Mus musculus]	27	2	2
Q9DAP7	Histone chaperone ASF1B [OS=Mus musculus]	38	4	4
Q9DAR7	M7GpppX diphosphatase [OS=Mus musculus]	35	8	8
Q9DAT5	Mitochondrial tRNA-specific 2-thiouridylase 1 [OS=Mus musculus]	10	2	2
Q9DAU1	Protein canopy homolog 3 [OS=Mus musculus]	18	5	5
Q9DAW6	U4/U6 small nuclear ribonucleoprotein Prp4 [OS=Mus musculus]	34	11	11
Q9DB05	alpha-soluble nsf attachment protein [OS=Mus musculus]	82	18	18
Q9DB15	39S ribosomal protein L12, mitochondrial [OS=Mus musculus]	48	6	6
Q9DB20	ATP synthase subunit O, mitochondrial [OS=Mus musculus]	55	9	9

Q9DB27-2	Isoform 2 of Malignant T-cell-amplified sequence 1 [OS=Mus musculus]	31	4	4
Q9DB29	Isoamyl acetate-hydrolyzing esterase 1 homolog [OS=Mus musculus]	36	5	5
Q9DB40-1	Mediator of RNA polymerase II transcription subunit 27 [OS=Mus musculus]	14	3	3
Q9DB43	Zinc finger protein-like 1 [OS=Mus musculus]	22	5	5
Q9DB73-1	NADH-cytochrome b5 reductase 1 [OS=Mus musculus]	54	8	8
Q9DB77	Cytochrome b-c1 complex subunit 2, mitochondrial [OS=Mus musculus]	73	19	19
Q9DB85	ribosomal rna-processing protein 8 [OS=Mus musculus]	6	2	2
Q9DB96	neuroguidin [OS=Mus musculus]	19	4	4
Q9DBB8	Trans-1,2-dihydrobenzene-1,2-diol dehydrogenase [OS=Mus musculus]	26	6	6
Q9DBC7	cAMP-dependent protein kinase type I-alpha regulatory subunit [OS=Mus musculus]	41	12	12
Q9DBD5	Proline-, glutamic acid- and leucine-rich protein 1 [OS=Mus musculus]	17	11	11
Q9DBE9	pre-rRNA processing protein FTSJ3 [OS=Mus musculus]	26	12	12
Q9DBG3-2	Isoform 2 of AP-2 complex subunit beta [OS=Mus musculus]	57	45	27
Q9DBG5	Perilipin-3 [OS=Mus musculus]	31	7	7
Q9DBG6	Dolichyl-diphosphooligosaccharide--protein glycosyltransferase subunit 2 [OS=Mus musculus]	30	10	10
Q9DBG7	signal recognition particle receptor subunit alpha [OS=Mus musculus]	38	16	16
Q9DBH0	NEDD4-like E3 ubiquitin-protein ligase WWP2 [OS=Mus musculus]	3	2	2
Q9DBH5	Vesicular integral-membrane protein VIP36 [OS=Mus musculus]	59	17	17
Q9DBJ1	Phosphoglycerate mutase 1 [OS=Mus musculus]	77	17	15
Q9DBL1	Short/branched chain specific acyl-CoA dehydrogenase, mitochondrial [OS=Mus musculus]	9	2	2
Q9DBL7	Bifunctional coenzyme A synthase [OS=Mus musculus]	13	4	4
Q9DBN5-1	Lon protease homolog 2, peroxisomal [OS=Mus musculus]	5	3	3
Q9DBP5	UMP-CMP kinase [OS=Mus musculus]	55	10	10
Q9DBQ7-1	Protein-associating with the carboxyl-terminal domain of ezrin [OS=Mus musculus]	7	4	4
Q9DBR0	A-kinase anchor protein 8 [OS=Mus musculus]	21	7	7

Q9DBR1	5'-3' exoribonuclease 2 [OS=Mus musculus]	46	26	26
Q9DBR3	Armadillo repeat-containing protein 8 [OS=Mus musculus]	14	5	5
Q9DBR7-1	Protein phosphatase 1 regulatory subunit 12A [OS=Mus musculus]	8	6	5
Q9DBS1	Transmembrane protein 43 [OS=Mus musculus]	30	8	8
Q9DBT5	AMP deaminase 2 [OS=Mus musculus]	28	18	18
Q9DBY8	Nuclear valosin-containing protein-like [OS=Mus musculus]	3	2	2
Q9DBZ5	Eukaryotic translation initiation factor 3 subunit K [OS=Mus musculus]	70	12	12
Q9DC16	Endoplasmic reticulum-Golgi intermediate compartment protein 1 [OS=Mus musculus]	29	7	7
Q9DC23	DnaJ homolog subfamily C member 10 [OS=Mus musculus]	18	9	9
Q9DC51	Guanine nucleotide-binding protein G(k) subunit alpha [OS=Mus musculus]	48	14	7
Q9DC61	Mitochondrial-processing peptidase subunit alpha [OS=Mus musculus]	49	19	19
Q9DC69	NADH dehydrogenase [ubiquinone] 1 alpha subcomplex subunit 9, mitochondrial [OS=Mus musculus]	51	15	15
Q9DC70	NADH dehydrogenase [ubiquinone] iron-sulfur protein 7, mitochondrial [OS=Mus musculus]	38	5	5
Q9DC71	28S ribosomal protein S15, mitochondrial [OS=Mus musculus]	17	4	4
Q9DCA2	28S ribosomal protein S11, mitochondrial [OS=Mus musculus]	9	2	2
Q9DCA5	Ribosome biogenesis protein BRX1 homolog [OS=Mus musculus]	24	5	5
Q9DCB8	iron-sulfur cluster assembly 2 homolog, mitochondrial [OS=Mus musculus]	29	3	3
Q9DCC4	Pyrroline-5-carboxylate reductase 3 [OS=Mus musculus]	13	2	2
Q9DCD0	6-phosphogluconate dehydrogenase, decarboxylating [OS=Mus musculus]	80	30	30
Q9DCD2	Pre-mRNA-splicing factor SYF1 [OS=Mus musculus]	3	2	2
Q9DCG9	Multifunctional methyltransferase subunit TRM112-like protein [OS=Mus musculus]	48	4	4
Q9DCH4	Eukaryotic translation initiation factor 3 subunit F [OS=Mus musculus]	43	12	12
Q9DCJ5	NADH dehydrogenase [ubiquinone] 1 alpha subcomplex subunit 8 [OS=Mus musculus]	56	7	7
Q9DCJ9	N-acetylneuraminate lyase [OS=Mus musculus]	27	5	5
Q9DCL9	multifunctional protein ADE2 [OS=Mus musculus]	63	22	22
Q9DCM0	Persulfide dioxygenase ETHE1, mitochondrial [OS=Mus musculus]	41	6	6

Q9DCN2	NADH-cytochrome b5 reductase 3 [OS=Mus musculus]	69	17	17
Q9DCS2	Methyltransferase-like 26 [OS=Mus musculus]	25	2	2
Q9DCS3	Enoyl-[acyl-carrier-protein] reductase, mitochondrial [OS=Mus musculus]	66	13	13
Q9DCS9	NADH dehydrogenase [ubiquinone] 1 beta subcomplex subunit 10 [OS=Mus musculus]	56	7	7
Q9DCT2	NADH dehydrogenase [ubiquinone] iron-sulfur protein 3, mitochondrial [OS=Mus musculus]	41	10	10
Q9DCU6	39S ribosomal protein L4, mitochondrial [OS=Mus musculus]	38	6	6
Q9DCV4	regulator of microtubule dynamics protein 1 [OS=Mus musculus]	8	2	2
Q9DCW4	electron transfer flavoprotein subunit beta [OS=Mus musculus]	50	12	12
Q9DCX2	ATP synthase subunit d, mitochondrial [OS=Mus musculus]	63	8	8
Q9DCZ1	GMP reductase 1 [OS=Mus musculus]	11	2	2
Q9DCZ4-3	Isoform 3 of MICOS complex subunit Mic26 [OS=Mus musculus]	32	5	5
Q9DD18	D-aminoacyl-tRNA deacylase 1 [OS=Mus musculus]	15	2	2
Q9EP72	ER membrane protein complex subunit 7 [OS=Mus musculus]	22	3	3
Q9EP89	Serine beta-lactamase-like protein LACTB, mitochondrial [OS=Mus musculus]	29	9	9
Q9EP97	Sentrin-specific protease 3 [OS=Mus musculus]	15	4	4
Q9EPB5	Serine hydrolase-like protein [OS=Mus musculus]	21	4	4
Q9EPJ9	ADP-ribosylation factor GTPase-activating protein 1 [OS=Mus musculus]	17	4	4
Q9EPK6	nucleotide exchange factor SIL1 [OS=Mus musculus]	12	3	3
Q9EPK7	exportin-7 [OS=Mus musculus]	11	7	7
Q9EPL8	Importin-7 [OS=Mus musculus]	42	33	32
Q9EPU0-1	Regulator of nonsense transcripts 1 [OS=Mus musculus]	30	25	25
Q9EQ06	Estradiol 17-beta-dehydrogenase 11 [OS=Mus musculus]	40	8	8
Q9EQ28	DNA polymerase delta subunit 3 [OS=Mus musculus]	12	4	4
Q9EQ32-1	Phosphoinositide 3-kinase adapter protein 1 [OS=Mus musculus]	15	9	9
Q9EQ61	Pescadillo homolog [OS=Mus musculus]	27	10	10
Q9EQ80	NIF3-like protein 1 [OS=Mus musculus]	26	6	6
Q9EQC5	N-terminal kinase-like protein [OS=Mus musculus]	10	3	3
Q9EQG9-1	Collagen type IV alpha-3-binding protein [OS=Mus musculus]	15	4	4
Q9EQH2	endoplasmic reticulum aminopeptidase 1 [OS=Mus musculus]	13	10	10

Q9EQH3	Vacuolar protein sorting-associated protein 35 [OS=Mus musculus]	58	3<br>2	32
Q9EQI8	39S ribosomal protein L46, mitochondrial [OS=Mus musculus]	28	6	6
Q9EQK5	major vault protein [OS=Mus musculus]	59	3<br>3	33
Q9EQP2	EH domain-containing protein 4 [OS=Mus musculus]	64	2<br>6	25
Q9EQQ9-3	Isoform 3 of Protein O-GlcNAcase [OS=Mus musculus]	11	5	5
Q9EQS3	C-Myc-binding protein [OS=Mus musculus]	30	2	2
Q9EQU5	Protein SET [OS=Mus musculus]	41	1<br>2	3
Q9ER00	Syntaxin-12 [OS=Mus musculus]	39	6	6
Q9ER38	Torsin-3A [OS=Mus musculus]	21	6	6
Q9ER39	Torsin-1A [OS=Mus musculus]	20	5	5
Q9ER41	Torsin-1B [OS=Mus musculus]	18	4	4
Q9ER69	Pre-mRNA-splicing regulator WTAP [OS=Mus musculus]	11	3	3
Q9ER72	Cysteine--tRNA ligase, cytoplasmic [OS=Mus musculus]	42	2<br>8	28
Q9ER88-1	28S ribosomal protein S29, mitochondrial [OS=Mus musculus]	54	1<br>4	14
Q9ERB0	Synaptosomal-associated protein 29 [OS=Mus musculus]	23	4	4
Q9ERD7	tubulin beta-3 chain [OS=Mus musculus]	53	2<br>2	5
Q9ERD8	Gamma-parvin [OS=Mus musculus]	33	8	8
Q9ERE7	LRP chaperone MESD [OS=Mus musculus]	21	3	3
Q9ERF3	WD repeat-containing protein 61 [OS=Mus musculus]	49	8	8
Q9ERG0	LIM domain and actin-binding protein 1 [OS=Mus musculus]	4	2	2
Q9ERG2	Striatin-3 [OS=Mus musculus]	7	4	4
Q9ERH4-1	Nucleolar and spindle-associated protein 1 [OS=Mus musculus]	12	2	2
Q9ERI2	Ras-related protein Rab-27A [OS=Mus musculus]	11	2	2
Q9ERI5-1	Bifunctional arginine demethylase and lysyl-hydroxylase JMJD6 [OS=Mus musculus]	14	4	4
Q9ERK4	Exportin-2 [OS=Mus musculus]	55	3<br>9	39
Q9ERN0	Secretory carrier-associated membrane protein 2 [OS=Mus musculus]	11	4	4
Q9ERR7	selenoprotein F [OS=Mus musculus]	52	7	7
Q9ERS2	NADH dehydrogenase [ubiquinone] 1 alpha subcomplex subunit 13 [OS=Mus musculus]	31	5	5
Q9ERS5	Pleckstrin homology domain-containing family A member 2 [OS=Mus musculus]	6	2	2

Q9ERU9	E3 SUMO-protein ligase RanBP2 [OS=Mus musculus]	21	37	37
Q9ES00	Ubiquitin conjugation factor E4 B [OS=Mus musculus]	18	14	14
Q9ES28-1	Rho guanine nucleotide exchange factor 7 [OS=Mus musculus]	24	12	10
Q9ES46	Beta-parvin [OS=Mus musculus]	31	7	7
Q9ES52-1	Phosphatidylinositol 3,4,5-trisphosphate 5-phosphatase 1 [OS=Mus musculus]	16	13	13
Q9ES74	Serine/threonine-protein kinase Nek7 [OS=Mus musculus]	16	3	3
Q9ES97-3	Isoform 3 of Reticulon-3 [OS=Mus musculus]	28	3	3
Q9ESD6-1	CKLF-like MARVEL transmembrane domain-containing protein 7 [OS=Mus musculus]	19	2	2
Q9ESJ0	Exportin-4 [OS=Mus musculus]	8	7	7
Q9ESL4-2	Isoform 2 of Mitogen-activated protein kinase kinase kinase 20 [OS=Mus musculus]	8	3	3
Q9ESP1	Stromal cell-derived factor 2-like protein 1 [OS=Mus musculus]	60	6	6
Q9EST4-1	Proteasome assembly chaperone 2 [OS=Mus musculus]	11	3	3
Q9EST5-2	Isoform 2 of Acidic leucine-rich nuclear phosphoprotein 32 family member B [OS=Mus musculus]	25	7	6
Q9EST8	NF-kappa-B inhibitor zeta [OS=Mus musculus]	5	2	2
Q9ESU6	Bromodomain-containing protein 4 [OS=Mus musculus]	6	6	5
Q9ESV0	ATP-dependent RNA helicase DDX24 [OS=Mus musculus]	9	4	4
Q9ESW4	Acylglycerol kinase, mitochondrial [OS=Mus musculus]	27	7	7
Q9ESY9	Gamma-interferon-inducible lysosomal thiol reductase [OS=Mus musculus]	29	7	7
Q9ESZ8-1	General transcription factor II-I [OS=Mus musculus]	34	21	21
Q9ET22	Dipeptidyl peptidase 2 [OS=Mus musculus]	13	5	5
Q9ET30	Transmembrane 9 superfamily member 3 [OS=Mus musculus]	8	3	3
Q9JHI5	Isovaleryl-CoA dehydrogenase, mitochondrial [OS=Mus musculus]	41	9	9
Q9JHI7	Exosome complex component RRP45 [OS=Mus musculus]	8	2	2
Q9JHJ0	tropomodulin-3 [OS=Mus musculus]	41	9	9
Q9JHJ3	Glycosylated lysosomal membrane protein [OS=Mus musculus]	10	2	2
Q9JHK5	pleckstrin [OS=Mus musculus]	53	14	14
Q9JHP7-1	KDEL motif-containing protein 1 [OS=Mus musculus]	17	6	6
Q9JHR7	insulin-degrading enzyme [OS=Mus musculus]	31	21	21
Q9JHS3	Ragulator complex protein LAMTOR2 [OS=Mus musculus]	74	5	5

Q9JHS4	ATP-dependent Clp protease ATP-binding subunit clpX-like, mitochondrial [OS=Mus musculus]	30	15	15
Q9JHU9	Inositol-3-phosphate synthase 1 [OS=Mus musculus]	34	11	11
Q9JHW2	Omega-amidase NIT2 [OS=Mus musculus]	70	14	14
Q9JI10	serine/threonine-protein kinase 3 [OS=Mus musculus]	16	6	3
Q9JI11	serine/threonine-protein kinase 4 [OS=Mus musculus]	21	7	4
Q9JI13-1	Something about silencing protein 10 [OS=Mus musculus]	16	4	4
Q9JI75	Ribosyldihydronicotinamide dehydrogenase [quinone] [OS=Mus musculus]	23	3	3
Q9JIA7	Sphingosine kinase 2 [OS=Mus musculus]	7	3	3
Q9JIF0-1	protein arginine N-methyltransferase 1 [OS=Mus musculus]	7	3	3
Q9JIF7	Coatomer subunit beta [OS=Mus musculus]	43	27	27
Q9JIG7	Coiled-coil domain-containing protein 22 [OS=Mus musculus]	41	17	17
Q9JIH2	Nuclear pore complex protein Nup50 [OS=Mus musculus]	42	11	11
Q9JII5	DAZ-associated protein 1 [OS=Mus musculus]	32	8	8
Q9JII6	alcohol dehydrogenase [NADP(+)] [OS=Mus musculus]	63	14	13
Q9JIK5	Nucleolar RNA helicase 2 [OS=Mus musculus]	43	24	24
Q9JIK9	28S ribosomal protein S34, mitochondrial [OS=Mus musculus]	56	10	10
Q9JIW9	Ras-related protein Ral-B [OS=Mus musculus]	51	10	4
Q9JIX8	Apoptotic chromatin condensation inducer in the nucleus [OS=Mus musculus]	3	2	2
Q9JIY5	Serine protease HTRA2, mitochondrial [OS=Mus musculus]	12	4	4
Q9JIZ9	Phospholipid scramblase 3 [OS=Mus musculus]	13	2	2
Q9JJ11	Transforming acidic coiled-coil-containing protein 3 [OS=Mus musculus]	5	2	2
Q9JJ28	protein flightless-1 homolog [OS=Mus musculus]	4	4	4
Q9JJ78	Lymphokine-activated killer T-cell-originated protein kinase [OS=Mus musculus]	8	2	2
Q9JJ80	Ribosome production factor 2 homolog [OS=Mus musculus]	9	2	2
Q9JJA4	ribosome biogenesis protein WDR12 [OS=Mus musculus]	10	2	2
Q9JJD0	THAP domain-containing protein 11 [OS=Mus musculus]	6	2	2
Q9JJF3	ribosomal oxygenase 1 [OS=Mus musculus]	20	6	6

Q9JJI8	60s ribosomal protein l38 [OS=Mus musculus]	57	7	7
Q9JJK2	lanC-like protein 2 [OS=Mus musculus]	12	3	3
Q9JJK5	homocysteine-responsive endoplasmic reticulum-resident ubiquitin-like domain member 1 protein [OS=Mus musculus]	10	2	2
Q9JJL8	Serine--tRNA ligase, mitochondrial [OS=Mus musculus]	24	7	7
Q9JJT0	RNA 3'-terminal phosphate cyclase-like protein [OS=Mus musculus]	10	3	3
Q9JJU8	SH3 domain-binding glutamic acid-rich-like protein [OS=Mus musculus]	84	8	8
Q9JJZ4	ubiquitin-conjugating enzyme e2 j1 [OS=Mus musculus]	29	5	5
Q9JK23	Proteasome assembly chaperone 1 [OS=Mus musculus]	34	6	6
Q9JK30-1	Origin recognition complex subunit 3 [OS=Mus musculus]	3	2	2
Q9JK38	glucosamine 6-phosphate N-acetyltransferase [OS=Mus musculus]	41	4	4
Q9JK48-2	Isoform 2 of Endophilin-B1 [OS=Mus musculus]	20	5	5
Q9JK81	UPF0160 protein MYG1, mitochondrial [OS=Mus musculus]	56	1<br>5	15
Q9JKB1	Ubiquitin carboxyl-terminal hydrolase isozyme L3 [OS=Mus musculus]	48	9	9
Q9JKB3-2	Isoform 2 of Y-box-binding protein 3 [OS=Mus musculus]	51	1<br>0	5
Q9JKC8	ap-3 complex subunit mu-1 [OS=Mus musculus]	8	2	2
Q9JKF1	Ras GTPase-activating-like protein IQGAP1 [OS=Mus musculus]	63	8<br>1	81
Q9JKF7	39S ribosomal protein L39, mitochondrial [OS=Mus musculus]	24	6	6
Q9JKK1-1	Syntaxin-6 [OS=Mus musculus]	22	4	4
Q9JKL4	NADH dehydrogenase [ubiquinone] 1 alpha subcomplex assembly factor 3 [OS=Mus musculus]	17	2	2
Q9JKP5	Muscleblind-like protein 1 [OS=Mus musculus]	25	5	5
Q9JKR6	Hypoxia up-regulated protein 1 [OS=Mus musculus]	49	4<br>4	44
Q9JKV1	Proteasomal ubiquitin receptor ADRM1 [OS=Mus musculus]	15	6	6
Q9JKW0	ADP-ribosylation factor-like protein 6-interacting protein 1 [OS=Mus musculus]	19	2	2
Q9JKX4-1	Protein AATF [OS=Mus musculus]	14	4	4
Q9JKX6	ADP-sugar pyrophosphatase [OS=Mus musculus]	48	7	7
Q9JKY0	CCR4-NOT transcription complex subunit 9 [OS=Mus musculus]	41	8	8
Q9JL16	Interferon-stimulated gene 20 kDa protein [OS=Mus musculus]	7	2	2
Q9JL26	formin-like protein 1 [OS=Mus musculus]	37	3<br>1	31
Q9JL35	High mobility group nucleosome-binding domain-containing protein 5 [OS=Mus musculus]	15	3	3

Q9JLB0-1	MAGUK p55 subfamily member 6 [OS=Mus musculus]	28	11	11
Q9JLI6-1	selenocysteine lyase [OS=Mus musculus]	11	3	3
Q9JLI8-1	Squamous cell carcinoma antigen recognized by T-cells 3 [OS=Mus musculus]	25	15	15
Q9JLJ2	4-trimethylaminobutyraldehyde dehydrogenase [OS=Mus musculus]	69	22	22
Q9JLQ0	CD2-associated protein [OS=Mus musculus]	21	8	8
Q9JLQ2	ARF GTPase-activating protein GIT2 [OS=Mus musculus]	22	8	7
Q9JLR9	HIG1 domain family member 1A, mitochondrial [OS=Mus musculus]	42	2	2
Q9JLT4	thioredoxin reductase 2, mitochondrial [OS=Mus musculus]	15	4	3
Q9JLV1	BAG family molecular chaperone regulator 3 [OS=Mus musculus]	5	2	2
Q9JLV5	Cullin-3 [OS=Mus musculus]	27	17	17
Q9JLV6-1	Bifunctional polynucleotide phosphatase/kinase [OS=Mus musculus]	46	13	13
Q9JM13	Rab5 GDP/GTP exchange factor [OS=Mus musculus]	21	8	8
Q9JM14	5'(3')-deoxyribonucleotidase, cytosolic type [OS=Mus musculus]	43	5	5
Q9JM76	Actin-related protein 2/3 complex subunit 3 [OS=Mus musculus]	33	6	6
Q9JM90-1	Signal-transducing adaptor protein 1 [OS=Mus musculus]	16	3	3
Q9JMA1	Ubiquitin carboxyl-terminal hydrolase 14 [OS=Mus musculus]	64	24	24
Q9JMA2	Queuine tRNA-ribosyltransferase catalytic subunit 1 [OS=Mus musculus]	20	5	5
Q9JMD0	BUB3-interacting and GLEBS motif-containing protein ZNF207 [OS=Mus musculus]	5	3	3
Q9JMG1	Endothelial differentiation-related factor 1 [OS=Mus musculus]	18	2	2
Q9JMH6-1	Thioredoxin reductase 1, cytoplasmic [OS=Mus musculus]	40	16	15
Q9JMH9-6	Isoform 6 of Unconventional myosin-XVIIIa [OS=Mus musculus]	4	5	5
Q9QUH0	Glutaredoxin-1 [OS=Mus musculus]	70	5	5
Q9QUI0	Transforming protein RhoA [OS=Mus musculus]	80	13	4
Q9QUJ7-1	Long-chain-fatty-acid--CoA ligase 4 [OS=Mus musculus]	38	16	15
Q9QUM9	Proteasome subunit alpha type-6 [OS=Mus musculus]	49	12	12
Q9QUN7	toll-like receptor 2 [OS=Mus musculus]	29	16	16
Q9QUR6	prolyl endopeptidase [OS=Mus musculus]	50	24	24

Q9QUR7	peptidyl-prolyl cis-trans isomerase NIMA-interacting 1 [OS=Mus musculus]	67	7	7
Q9QVP9	Protein-tyrosine kinase 2-beta [OS=Mus musculus]	3	2	2
Q9QWR8	alpha-N-acetylgalactosaminidase [OS=Mus musculus]	25	8	8
Q9QWT9-1	Kinesin-like protein KIFC1 [OS=Mus musculus]	9	5	5
Q9QXA5	U6 snRNA-associated Sm-like protein LSm4 [OS=Mus musculus]	11	2	2
Q9QXB9	Developmentally-regulated GTP-binding protein 2 [OS=Mus musculus]	35	9	9
Q9QXD8	LIM domain-containing protein 1 [OS=Mus musculus]	10	3	3
Q9QXE7	F-box-like/WD repeat-containing protein TBL1X [OS=Mus musculus]	23	6	3
Q9QXG4	Acetyl-coenzyme A synthetase, cytoplasmic [OS=Mus musculus]	4	2	2
Q9QXK2	E3 ubiquitin-protein ligase RAD18 [OS=Mus musculus]	9	3	3
Q9QXK3	Coatomer subunit gamma-2 [OS=Mus musculus]	34	17	16
Q9QXK7	Cleavage and polyadenylation specificity factor subunit 3 [OS=Mus musculus]	5	2	2
Q9QXS1-1	plectin [OS=Mus musculus]	2	6	6
Q9QXT0	Protein canopy homolog 2 [OS=Mus musculus]	55	8	8
Q9QXX4	Calcium-binding mitochondrial carrier protein Aralar2 [OS=Mus musculus]	58	24	20
Q9QXZ0	Microtubule-actin cross-linking factor 1 [OS=Mus musculus]	1	8	8
Q9QY24-1	Z-DNA-binding protein 1 [OS=Mus musculus]	30	5	5
Q9QY36	N-alpha-acetyltransferase 10 [OS=Mus musculus]	32	4	4
Q9QY76	vesicle-associated membrane protein-associated protein B [OS=Mus musculus]	32	6	5
Q9QYA2	Mitochondrial import receptor subunit TOM40 homolog [OS=Mus musculus]	68	13	13
Q9QYB1	Chloride intracellular channel protein 4 [OS=Mus musculus]	76	17	16
Q9QYC0-1	Alpha-adducin [OS=Mus musculus]	24	9	9
Q9QYE6	Golgin subfamily A member 5 [OS=Mus musculus]	22	10	10
Q9QYF1	Retinol dehydrogenase 11 [OS=Mus musculus]	31	8	8
Q9QYF9	protein NDRG3 [OS=Mus musculus]	17	4	4
Q9QYG0-2	Isoform 2 of Protein NDRG2 [OS=Mus musculus]	53	12	12
Q9QYI3	DnaJ homolog subfamily C member 7 [OS=Mus musculus]	42	16	16
Q9QYI4	DnaJ homolog subfamily B member 12 [OS=Mus musculus]	8	2	2
Q9QYJ0	DnaJ homolog subfamily A member 2 [OS=Mus musculus]	37	8	8
Q9QYJ3	dnaJ homolog subfamily B member 1 [OS=Mus musculus]	23	6	5

Q9QYR9	Acyl-coenzyme A thioesterase 2, mitochondrial [OS=Mus musculus]	27	8	8
Q9QYS9-7	Isoform 7 of Protein quaking [OS=Mus musculus]	25	6	6
Q9QYY8	spastin [OS=Mus musculus]	5	2	2
Q9QZ06	Toll-interacting protein [OS=Mus musculus]	15	3	3
Q9QZ08	N-acetyl-D-glucosamine kinase [OS=Mus musculus]	13	3	3
Q9QZ23	NFU1 iron-sulfur cluster scaffold homolog, mitochondrial [OS=Mus musculus]	17	3	3
Q9QZ73	DCN1-like protein 1 [OS=Mus musculus]	16	3	3
Q9QZ88	Vacuolar protein sorting-associated protein 29 [OS=Mus musculus]	42	5	5
Q9QZA0	Carbonic anhydrase 5B, mitochondrial [OS=Mus musculus]	9	2	2
Q9QZB7	Actin-related protein 10 [OS=Mus musculus]	15	4	4
Q9QZD8	Mitochondrial dicarboxylate carrier [OS=Mus musculus]	14	2	2
Q9QZD9	Eukaryotic translation initiation factor 3 subunit I [OS=Mus musculus]	55	10	10
Q9QZE5	Coatomer subunit gamma-1 [OS=Mus musculus]	47	24	23
Q9QZE7	Translin-associated protein X [OS=Mus musculus]	19	3	3
Q9QZH3	Peptidyl-prolyl cis-trans isomerase E [OS=Mus musculus]	14	2	2
Q9QZH6	Evolutionarily conserved signaling intermediate in Toll pathway, mitochondrial [OS=Mus musculus]	12	3	3
Q9QZK7-1	Docking protein 3 [OS=Mus musculus]	23	6	6
Q9QZL0	Receptor-interacting serine/threonine-protein kinase 3 [OS=Mus musculus]	17	5	5
Q9QZM0	Ubiquilin-2 [OS=Mus musculus]	17	8	6
Q9QZQ1	Afadin [OS=Mus musculus]	5	6	6
Q9QZQ8-1	Core histone macro-H2A.1 [OS=Mus musculus]	13	2	2
Q9QZS3-1	Protein numb homolog [OS=Mus musculus]	8	3	3
Q9R013	Cathepsin F [OS=Mus musculus]	7	2	2
Q9R059	four and a half LIM domains protein 3 [OS=Mus musculus]	19	4	4
Q9R060	Cytosolic Fe-S cluster assembly factor nubp1 [OS=Mus musculus]	18	4	4
Q9R061	Cytosolic Fe-S cluster assembly factor NUBP2 [OS=Mus musculus]	16	4	4
Q9R062	Glycogenin-1 [OS=Mus musculus]	9	2	2
Q9R099	Transducin beta-like protein 2 [OS=Mus musculus]	11	4	4
Q9R0A0	Peroxisomal membrane protein PEX14 [OS=Mus musculus]	30	8	8
Q9R0E1	Procollagen-lysine,2-oxoglutarate 5-dioxygenase 3 [OS=Mus musculus]	38	17	17
Q9R0E2	Procollagen-lysine,2-oxoglutarate 5-dioxygenase 1 [OS=Mus musculus]	32	15	15
Q9R0G7	zinc finger E-box-binding homeobox 2 [OS=Mus musculus]	4	3	3
Q9R0H0-1	Peroxisomal acyl-coenzyme A oxidase 1 [OS=Mus musculus]	10	3	3
Q9R0I7	YLP motif-containing protein 1 [OS=Mus musculus]	4	3	3

Q9R0M6	Ras-related protein Rab-9A [OS=Mus musculus]	37	5	5
Q9R0N0	galactokinase [OS=Mus musculus]	48	12	12
Q9R0P3	S-formylglutathione hydrolase [OS=Mus musculus]	88	20	20
Q9R0P5	Destrin [OS=Mus musculus]	48	6	6
Q9R0P6	Signal peptidase complex catalytic subunit SEC11A [OS=Mus musculus]	44	7	7
Q9R0Q3	Transmembrane emp24 domain-containing protein 2 [OS=Mus musculus]	26	3	3
Q9R0Q4	Mortality factor 4-like protein 2 [OS=Mus musculus]	35	7	5
Q9R0Q7	Prostaglandin E synthase 3 [OS=Mus musculus]	37	6	6
Q9R0U0-1	Serine/arginine-rich splicing factor 10 [OS=Mus musculus]	10	2	2
Q9R0X0	Mediator of RNA polymerase II transcription subunit 20 [OS=Mus musculus]	9	2	2
Q9R0X4	Acyl-coenzyme A thioesterase 9, mitochondrial [OS=Mus musculus]	35	13	13
Q9R112	Sulfide:quinone oxidoreductase, mitochondrial [OS=Mus musculus]	54	24	24
Q9R190	Metastasis-associated protein MTA2 [OS=Mus musculus]	31	17	15
Q9R1C7-1	pre-mRNA-processing factor 40 homolog A [OS=Mus musculus]	5	3	3
Q9R1J0	sterol-4-alpha-carboxylate 3-dehydrogenase, decarboxylating [OS=Mus musculus]	51	12	12
Q9R1P0	Proteasome subunit alpha type-4 [OS=Mus musculus]	57	9	9
Q9R1P1	proteasome subunit beta type-3 [OS=Mus musculus]	51	9	9
Q9R1P3	proteasome subunit beta type-2 [OS=Mus musculus]	78	12	12
Q9R1P4	Proteasome subunit alpha type-1 [OS=Mus musculus]	71	15	15
Q9R1Q7	proteolipid protein 2 [OS=Mus musculus]	35	3	3
Q9R1Q9	V-type proton ATPase subunit S1 [OS=Mus musculus]	27	10	10
Q9R1T2	SUMO-activating enzyme subunit 1 [OS=Mus musculus]	59	12	12
Q9R1T4	Septin-6 [OS=Mus musculus]	14	5	3
Q9R1X4	Protein timeless homolog [OS=Mus musculus]	2	2	2
Q9R207	Nibrin [OS=Mus musculus]	4	2	2
Q9R233	Tapasin [OS=Mus musculus]	32	9	9
Q9WTI7-1	Unconventional myosin-Ic [OS=Mus musculus]	34	24	23

Q9WTK5	Nuclear factor NF-kappa-B p100 subunit [OS=Mus musculus]	29	16	16
Q9WTM5	RuvB-like 2 [OS=Mus musculus]	64	22	22
Q9WTP6-1	Adenylate kinase 2, mitochondrial [OS=Mus musculus]	60	11	11
Q9WTP7	GTP:AMP phosphotransferase AK3, mitochondrial [OS=Mus musculus]	54	9	9
Q9WTQ8	Mitochondrial import inner membrane translocase subunit tim23 [OS=Mus musculus]	42	4	4
Q9WTX5	s-phase kinase-associated protein 1 [OS=Mus musculus]	63	6	6
Q9WTX6	Cullin-1 [OS=Mus musculus]	14	7	7
Q9WTX8-1	mitotic spindle assembly checkpoint protein MAD1 [OS=Mus musculus]	15	6	6
Q9WU28	prefoldin subunit 5 [OS=Mus musculus]	55	5	5
Q9WU40-2	Isoform 2 of Inner nuclear membrane protein Man1 [OS=Mus musculus]	8	4	4
Q9WU56	tRNA pseudouridine synthase A [OS=Mus musculus]	23	8	8
Q9WU78-3	Isoform 3 of Programmed cell death 6-interacting protein [OS=Mus musculus]	51	30	30
Q9WU81-2	Isoform 2 of Glucose-6-phosphate exchanger SLC37A2 [OS=Mus musculus]	7	2	2
Q9WU84	copper chaperone for superoxide dismutase [OS=Mus musculus]	14	2	2
Q9WUA2	Phenylalanine--tRNA ligase beta subunit [OS=Mus musculus]	38	16	16
Q9WUA3-1	ATP-dependent 6-phosphofructokinase, platelet type [OS=Mus musculus]	29	14	12
Q9WUD1	STIP1 homology and U box-containing protein 1 [OS=Mus musculus]	32	6	6
Q9WUK2-1	Eukaryotic translation initiation factor 4H [OS=Mus musculus]	43	8	8
Q9WUK4	Replication factor C subunit 2 [OS=Mus musculus]	40	9	9
Q9WUL7	ADP-ribosylation factor-like protein 3 [OS=Mus musculus]	18	2	2
Q9WUM3	Coronin-1B [OS=Mus musculus]	23	5	5
Q9WUM4	coronin-1C [OS=Mus musculus]	19	5	5
Q9WUM5	Succinate--CoA ligase [ADP/GDP-forming] subunit alpha, mitochondrial [OS=Mus musculus]	28	8	8
Q9WUP7-1	Ubiquitin carboxyl-terminal hydrolase isozyme L5 [OS=Mus musculus]	63	15	15
Q9WUQ2	Prolactin regulatory element-binding protein [OS=Mus musculus]	11	2	2
Q9WUR2	Enoyl-CoA delta isomerase 2, mitochondrial [OS=Mus musculus]	13	3	3
Q9WUR9	Adenylate kinase 4, mitochondrial [OS=Mus musculus]	48	7	7

Q9WUU7	Cathepsin Z [OS=Mus musculus]	53	11	11
Q9WUU8	TNFAIP3-interacting protein 1 [OS=Mus musculus]	4	2	2
Q9WV32	Actin-related protein 2/3 complex subunit 1B [OS=Mus musculus]	16	4	4
Q9WV54	Acid ceramidase [OS=Mus musculus]	39	12	12
Q9WV55	vesicle-associated membrane protein-associated protein A [OS=Mus musculus]	55	10	9
Q9WV60	Glycogen synthase kinase-3 beta [OS=Mus musculus]	8	2	2
Q9WV70	Nucleolar complex protein 2 homolog [OS=Mus musculus]	12	5	5
Q9WV80	Sorting nexin-1 [OS=Mus musculus]	31	13	11
Q9WV98	Mitochondrial import inner membrane translocase subunit Tim9 [OS=Mus musculus]	65	5	5
Q9WVA2	Mitochondrial import inner membrane translocase subunit Tim8 A [OS=Mus musculus]	23	2	2
Q9WVA3	Mitotic checkpoint protein BUB3 [OS=Mus musculus]	57	11	11
Q9WVA4	Transgelin-2 [OS=Mus musculus]	77	13	13
Q9WVE8	Protein kinase C and casein kinase substrate in neurons protein 2 [OS=Mus musculus]	21	7	7
Q9WVJ2	26S proteasome non-ATPase regulatory subunit 13 [OS=Mus musculus]	77	28	28
Q9WVK4	EH domain-containing protein 1 [OS=Mus musculus]	71	30	29
Q9WVL0	maleylacetoacetate isomerase [OS=Mus musculus]	18	3	3
Q9WVL2	signal transducer and activator of transcription 2 [OS=Mus musculus]	6	4	4
Q9WVM1	Rac GTPase-activating protein 1 [OS=Mus musculus]	9	4	4
Q9WVM3	Anaphase-promoting complex subunit 7 [OS=Mus musculus]	13	4	4
Q9WVQ5	Methylthioribulose-1-phosphate dehydratase [OS=Mus musculus]	16	3	3
Q9WVR4	Fragile X mental retardation syndrome-related protein 2 [OS=Mus musculus]	16	8	6
Q9Z0E0-1	Neurochondrin [OS=Mus musculus]	7	2	2
Q9Z0F8	Disintegrin and metalloproteinase domain-containing protein 17 [OS=Mus musculus]	25	13	13
Q9Z0G0	PDZ domain-containing protein GIPC1 [OS=Mus musculus]	26	6	6
Q9Z0H3-2	Isoform B of SWI/SNF-related matrix-associated actin-dependent regulator of chromatin subfamily B member 1 [OS=Mus musculus]	25	6	6

Q9Z0H4-4	Isoform 4 of CUGBP Elav-like family member 2 [OS=Mus musculus]	33	12	11
Q9Z0J0	Epididymal secretory protein E1 [OS=Mus musculus]	40	5	5
Q9Z0M5	lysosomal acid lipase/cholesteryl ester hydrolase [OS=Mus musculus]	39	11	11
Q9Z0N1	Eukaryotic translation initiation factor 2 subunit 3, X-linked [OS=Mus musculus]	48	15	4
Q9Z0N2	Eukaryotic translation initiation factor 2 subunit 3, Y-linked [OS=Mus musculus]	46	14	3
Q9Z0P5	Twinfilin-2 [OS=Mus musculus]	45	9	9
Q9Z0R6-1	Intersectin-2 [OS=Mus musculus]	5	5	5
Q9Z0R9	fatty acid desaturase 2 [OS=Mus musculus]	19	6	6
Q9Z0S1	3'(2'),5'-bisphosphate nucleotidase 1 [OS=Mus musculus]	30	5	5
Q9Z0U1	Tight junction protein ZO-2 [OS=Mus musculus]	2	2	2
Q9Z0V7	Mitochondrial import inner membrane translocase subunit Tim17-B [OS=Mus musculus]	30	3	3
Q9Z0V8	Mitochondrial import inner membrane translocase subunit Tim17-A [OS=Mus musculus]	32	2	2
Q9Z0X1	Apoptosis-inducing factor 1, mitochondrial [OS=Mus musculus]	44	20	20
Q9Z0Y1	Dynactin subunit 3 [OS=Mus musculus]	22	3	3
Q9Z103	Activity-dependent neuroprotector homeobox protein [OS=Mus musculus]	5	3	3
Q9Z110-1	delta-1-pyrroline-5-carboxylate synthase [OS=Mus musculus]	46	25	25
Q9Z120	tRNA (guanine-N(7)-)-methyltransferase [OS=Mus musculus]	18	3	3
Q9Z127	large neutral amino acids transporter small subunit 1 [OS=Mus musculus]	3	2	2
Q9Z130	Heterogeneous nuclear ribonucleoprotein D-like [OS=Mus musculus]	13	4	2
Q9Z148-1	Histone-lysine N-methyltransferase EHMT2 [OS=Mus musculus]	3	3	3
Q9Z1B5	Mitotic spindle assembly checkpoint protein MAD2A [OS=Mus musculus]	35	5	5
Q9Z1D1	Eukaryotic translation initiation factor 3 subunit G [OS=Mus musculus]	48	12	12
Q9Z1F9	SUMO-activating enzyme subunit 2 [OS=Mus musculus]	43	15	15
Q9Z1G3	V-type proton ATPase subunit C 1 [OS=Mus musculus]	52	14	14
Q9Z1J3-1	Cysteine desulfurase, mitochondrial [OS=Mus musculus]	20	6	6
Q9Z1K5	E3 ubiquitin-protein ligase ARIH1 [OS=Mus musculus]	16	6	6

Q9Z1K6	E3 ubiquitin-protein ligase ARIH2 [OS=Mus musculus]	6	2	2
Q9Z1M8	Protein Red [OS=Mus musculus]	14	6	6
Q9Z1N5	spliceosome RNA helicase DDX39B [OS=Mus musculus]	63	21	11
Q9Z1P6	NADH dehydrogenase [ubiquinone] 1 alpha subcomplex subunit 7 [OS=Mus musculus]	19	2	2
Q9Z1Q5	chloride intracellular channel protein 1 [OS=Mus musculus]	82	18	18
Q9Z1Q9	Valine--tRNA ligase [OS=Mus musculus]	50	39	39
Q9Z1R2	Large proline-rich protein BAG6 [OS=Mus musculus]	23	16	16
Q9Z1S0	Mitotic checkpoint serine/threonine-protein kinase BUB1 beta [OS=Mus musculus]	4	3	3
Q9Z1T1	AP-3 complex subunit beta-1 [OS=Mus musculus]	8	8	8
Q9Z1X4-3	Isoform 3 of Interleukin enhancer-binding factor 3 [OS=Mus musculus]	16	10	10
Q9Z1Z0-1	General vesicular transport factor p115 [OS=Mus musculus]	51	28	28
Q9Z1Z2	Serine-threonine kinase receptor-associated protein [OS=Mus musculus]	62	15	15
Q9Z204-2	Isoform C1 of Heterogeneous nuclear ribonucleoproteins C1/C2 [OS=Mus musculus]	26	6	6
Q9Z210	Peroxisomal membrane protein 11B [OS=Mus musculus]	19	4	4
Q9Z266-1	SNARE-associated protein Snapin [OS=Mus musculus]	43	3	3
Q9Z2D8-1	methyl-CpG-binding domain protein 3 [OS=Mus musculus]	39	6	6
Q9Z2G6-1	Protein sel-1 homolog 1 [OS=Mus musculus]	23	10	10
Q9Z2G9-1	Oxidoreductase htatip2 [OS=Mus musculus]	45	8	8
Q9Z2I0	mitochondrial proton/calcium exchanger protein [OS=Mus musculus]	40	18	18
Q9Z2I8	Succinate--CoA ligase [GDP-forming] subunit beta, mitochondrial [OS=Mus musculus]	54	16	16
Q9Z2I9	Succinate--CoA ligase [ADP-forming] subunit beta, mitochondrial [OS=Mus musculus]	38	12	12
Q9Z2K1	Keratin, type I cytoskeletal 16 [OS=Mus musculus]	13	6	2
Q9Z2L6	Multiple inositol polyphosphate phosphatase 1 [OS=Mus musculus]	11	3	3
Q9Z2L7-1	Cytokine receptor-like factor 3 [OS=Mus musculus]	34	6	6
Q9Z2M7	Phosphomannomutase 2 [OS=Mus musculus]	28	4	4
Q9Z2N8	Actin-like protein 6A [OS=Mus musculus]	36	10	10

Q9Z2Q5	39S ribosomal protein L40, mitochondrial [OS=Mus musculus]	25	3	3
Q9Z2U0	Proteasome subunit alpha type-7 [OS=Mus musculus]	65	14	14
Q9Z2U1	Proteasome subunit alpha type-5 [OS=Mus musculus]	48	9	9
Q9Z2W0	Aspartyl aminopeptidase [OS=Mus musculus]	47	13	13
Q9Z2X1-1	Heterogeneous nuclear ribonucleoprotein F [OS=Mus musculus]	64	15	13
Q9Z2X2	26S proteasome non-ATPase regulatory subunit 10 [OS=Mus musculus]	20	2	2
Q9Z2Y8	Pyridoxal phosphate homeostasis protein [OS=Mus musculus]	27	5	5
Q9Z2Z6	mitochondrial carnitine/acylcarnitine carrier protein [OS=Mus musculus]	18	4	4
Q9Z315	U4/U6.U5 tri-snRNP-associated protein 1 [OS=Mus musculus]	19	10	10

Supplementary Table S2. The p-values for the 50 most significant proteins identified and quantified in RAW 264.7 macrophages stimulated with phospholipid omega 3 or omega-6 alone and with LPS (n=36). (* if q < 0.05, ** if q < 0.01, *** if q < 0.001 and if **** q < 0.0001).

**Q07797**	Galectin-3-binding protein [OS=Mus musculus]	5,00237E-05	****
**P14901**	heme oxygenase 1 [OS=Mus musculus]	5,00237E-05	****
**Q64337**	sequestosome-1 [OS=Mus musculus]	5,00237E-05	****
**Q9R112**	Sulfide:quinone oxidoreductase, mitochondrial [OS=Mus musculus]	5,00237E-05	****
**P10852**	4F2 cell-surface antigen heavy chain [OS=Mus musculus]	5,00237E-05	****
**P37040**	NADPH--cytochrome P450 reductase [OS=Mus musculus]	5,00237E-05	****
**Q8R2Q8**	bone marrow stromal antigen 2 [OS=Mus musculus]	5,00237E-05	****
**P11928**	2'-5'-oligoadenylate synthase 1A [OS=Mus musculus]	5,00237E-05	****
**Q60766**	Immunity-related GTPase family M protein 1 [OS=Mus musculus]	5,00237E-05	****
**Q9JHK5**	pleckstrin [OS=Mus musculus]	5,00237E-05	****
**Q64516.3**	glycerol kinase [OS=Mus musculus]	5,00237E-05	****
**Q8VE37**	Regulator of chromosome condensation [OS=Mus musculus]	5,00237E-05	****
**P01900**	H-2 class I histocompatibility antigen, D-D alpha chain [OS=Mus musculus]	5,00237E-05	****
**O88569**	heterogeneous nuclear ribonucleoproteins A2/B1 [OS=Mus musculus]	5,00237E-05	****
**P61164**	Alpha-centractin [OS=Mus musculus]	5,00237E-05	****
**Q60737**	Casein kinase II subunit alpha [OS=Mus musculus]	5,00237E-05	****
**Q9CZ13**	Cytochrome b-c1 complex subunit 1, mitochondrial [OS=Mus musculus]	5,00237E-05	****

**P70404**	Isocitrate dehydrogenase [NAD] subunit gamma 1, mitochondrial [OS=Mus musculus]	5,00237E-05	****
**P97807.1**	fumarate hydratase, mitochondrial [OS=Mus musculus]	5,00237E-05	****
**P56480**	ATP synthase subunit beta, mitochondrial [OS=Mus musculus]	5,00237E-05	****
**P05202**	Aspartate aminotransferase, mitochondrial [OS=Mus musculus]	5,00237E-05	****
**Q9DB77**	Cytochrome b-c1 complex subunit 2, mitochondrial [OS=Mus musculus]	5,00237E-05	****
**Q8QZT1**	Acetyl-CoA acetyltransferase, mitochondrial [OS=Mus musculus]	5,00237E-05	****
**Q8BFR5**	elongation factor Tu, mitochondrial [OS=Mus musculus]	5,00237E-05	****
**Q9D6R2**	Isocitrate dehydrogenase [NAD] subunit alpha, mitochondrial [OS=Mus musculus]	5,00237E-05	****
**Q6ZQM8.1**	UDP-glucuronosyltransferase 1-7C [OS=Mus musculus]	5,00237E-05	****
**P24270**	catalase [OS=Mus musculus]	5,00237E-05	****
**O09172**	Glutamate--cysteine ligase regulatory subunit [OS=Mus musculus]	5,00237E-05	****
**Q923D2**	flavin reductase (NADPH) [OS=Mus musculus]	5,00237E-05	****
**Q9DC71**	28S ribosomal protein S15, mitochondrial [OS=Mus musculus]	5,00237E-05	****
**Q61881**	DNA replication licensing factor MCM7 [OS=Mus musculus]	5,00237E-05	****
**P97310**	DNA replication licensing factor mcm2 [OS=Mus musculus]	5,00237E-05	****
**Q99KC8**	von Willebrand factor A domain-containing protein 5A [OS=Mus musculus]	5,00237E-05	****
**Q61598**	Rab GDP dissociation inhibitor beta [OS=Mus musculus]	5,00237E-05	****
**P41241**	tyrosine-protein kinase CSK [OS=Mus musculus]	5,00237E-05	****
**P00493**	Hypoxanthine-guanine phosphoribosyltransferase [OS=Mus musculus]	5,00237E-05	****
**P00920**	Carbonic anhydrase 2 [OS=Mus musculus]	5,00237E-05	****
**Q921H8**	3-ketoacyl-CoA thiolase A, peroxisomal [OS=Mus musculus]	5,00237E-05	****

**Q8BWT1**	3-ketoacyl-CoA thiolase, mitochondrial [OS=Mus musculus]	5,00237E-05	****
**P00405**	Cytochrome c oxidase subunit 2 [OS=Mus musculus]	5,00237E-05	****
**O89017**	Legumain [OS=Mus musculus]	5,00237E-05	****
**Q9WV54**	Acid ceramidase [OS=Mus musculus]	5,00237E-05	****
**Q00612**	Glucose-6-phosphate 1-dehydrogenase X [OS=Mus musculus]	5,00237E-05	****
**P08905**	lysozyme c-2 [OS=Mus musculus]	5,00237E-05	****
**Q8BMS1**	Trifunctional enzyme subunit alpha, mitochondrial [OS=Mus musculus]	5,00237E-05	****
**Q9ESY9**	Gamma-interferon-inducible lysosomal thiol reductase [OS=Mus musculus]	5,00237E-05	****
**Q09014**	Neutrophil cytosol factor 1 [OS=Mus musculus]	5,00237E-05	****
**O88844**	Isocitrate dehydrogenase [NADP] cytoplasmic [OS=Mus musculus]	5,00237E-05	****
**Q9DCD0**	6-phosphogluconate dehydrogenase, decarboxylating [OS=Mus musculus]	5,00237E-05	****
**Q9JLJ2**	4-trimethylaminobutyraldehyde dehydrogenase [OS=Mus musculus]	5,00237E-05	****

Supplementary Table S3. Lipid species identified and quantified by HILIC-MS and MS/MS in RAW 264.7 macrophages stimulated with phospholipid omega-3 or omega-6 alone and with LPS (mass error < 5 ppm).

Label of the lipid species: AAA (xx:i) (AAA = lipid class abbreviation; xx = number of carbon atoms; i = number of double bonds). The 'O-' prefix is used for plasmanyl species to indicate the presence of an alkyl ether substituent, and the 'P-' prefix is used for plasmenyl species to indicate the alk-1-enyl ether substituent. Abbreviation of the lipid classes: acyl-carnitines (CAR), di-acylglycerols (DG), ceramides (Cer), hexosylceramides (HexCer), sphingomyelins (SM), phosphatidylcholines (PC), phosphatidylethanolamines (PE), phosphatidylinositols (PI), phosphatidylglycerols (PG), phosphatidylserine (PS), cardiolipins (CL), lysophosphatidylcholine (LPC), and lysophosphatidylethanolamines (LPE).

**Lipid species identified as [M-2H]2-**				
CL (54:0)	605,4006	605,4018	-1,9838	C63H122O17P2
CL (62:1)	660,4554	660,4545	1,3627	C71H134O17P2
CL (68:1)	702,5023	702,4996	3,8292	C77H148O17P2
CL (68:5)	698,4684	698,4713	-4,0490	C77H140O17P2
CL (70:3)	714,5043	714,5023	2,8131	C79H148O17P2
CL (70:4)	1427,9963	1427,9940	1,6106	C79H146O17P2
CL (70:6)	711,4788	711,4794	-0,8016	C79H142O17P2
CL (72:1)	730,5336	730,5333	0,4517	C81H154O17P2
CL (72:7)	724,4867	724,4857	1,3611	C81H144O17P2
CL (74:1)	744,5493	744,5513	-2,6996	C83H160O17P2
CL (74:3)	742,5336	742,5322	1,9258	C83H156O17P2
CL (74:9)	736,4867	736,4844	3,1365	C83H144O17P2
CL (76:1)	758,5649	758,5677	-3,7044	C85H164O17P2
CL (76:7)	752,5180	752,5173	0,9435	C85H152O17P2
CL (78:1)	772,5806	772,5843	-4,7491	C87H168O17P2
CL (78:13)	760,4867	760,4892	-3,3005	C87H144O17P2
CL (78:3)	770,5649	770,5663	-1,8168	C87H164O17P2
CL (78:7)	766,5336	766,5340	-0,4827	C87H156O17P2
CL (80:7)	780,5493	780,5519	-3,2797	C89H160O17P2
CL (82:3)	798,5962	798,5973	-1,3774	C91H172O17P2
CL (82:5)	796,5806	796,5815	-1,0671	C91H168O17P2
CL (84:11)	804,5493	804,5505	-1,4667	C93H160O17P2
**Lipid species identified as [M - H]-**				
CL (66:0)	1379,9963	1379,9908	3,9580	C75H145O17P2
CL (68:2)	1403,9963	1403,9904	4,2009	C77H145O17P2
CL (68:3)	1401,9800	1401,9801	-0,0635	C77H143O17P2
CL (68:4)	1399,9648	1399,9643	0,3793	C77H142O17P2
CL (70:5)	1425,9806	1425,9799	0,5007	C79H144O17P2
CL (70:7)	1421,9493	1421,9459	2,3742	C79H139O17P2

CL (72:4)	1456,0276	1456,0229	3,2273	C81H149O17P2
CL (72:5)	1454,0119	1454,0093	1,7572	C81H147O17P2
CL (72:6)	1451,9963	1451,9947	1,1191	C81H145O17P2
CL (74:7)	1478,0119	1478,0118	0,0670	C83H147O17P2
CL (74:8)	1475,9963	1475,9946	1,1186	C83H145O17P2
LPE (22:3)	530,3252	530,3256	-0,7128	C27H50NO7P
LPE (16:0)	452,2783	452,2788	-1,0171	C21H44NO7P
LPE (16:1)	450,2626	450,2626	0,0000	C21H42NO7P
LPE (18:0)	480,3096	480,3100	-0,8293	C23H48NO7P
LPE (18:1)	478,2939	478,2942	-0,5340	C23H46NO7P
LPE (18:2)	476,2783	476,2780	0,7139	C23H44NO7P
LPE (20:1)	506,3252	506,3262	-1,9158	C25H50NO7P
LPE (20:3)	502,2939	502,2948	-1,7910	C25H45NO7P
LPE (20:4)	500,2783	500,2790	-1,4592	C25H44NO7P
LPE (20:5)	498,2626	498,2636	-1,9668	C25H42NO7P
LPE (22:4)	528,3096	528,3101	-1,0019	C27H47NO7P
LPE (22:5)	526,2939	526,2942	-0,6227	C27H45NO7P
LPE (22:6)	524,2783	524,2781	0,3433	C27H44NO7P
LPE (O-16:0)	438,2990	438,2991	-0,1369	C21H46NO6P
LPE (O-16:1)	436,2833	436,2841	-1,9024	C21H44NO6P
LPE (O-18:0)	466,3303	466,3299	0,8363	C23H50NO6P
LPE (O-20:1)	492,3459	492,3446	2,6810	C25H52NO6P
LPE (P-18:0)	464,3147	464,3148	-0,3058	C23H47NO6P
LPE(O-18:1)	464,3146	464,3141	1,1199	C23H48NO6P
LPE(O-18:2)	462,2990	462,3000	-2,0549	C23H46NO6P
PE (30:0)	662,4766	662,4769	-0,4224	C35H69NO8P
PE(O-32:2)	672,4974	672,4965	1,2788	C37H72NO7P
PE(O-34:2)	700,5287	700,5294	-0,9564	C39H76NO7P
PE(O-34:3)	698,5130	698,5118	1,7466	C39H74NO7P
PE(O-36:1)	730,5756	730,5740	2,2174	C41H82NO7P
PE(O-36:2)	728,5600	728,5590	1,3451	C41H80NO7P
PE(O-36:3)	726,5443	726,5446	-0,4267	C41H78NO7P
PE(O-36:5)	722,5130	722,5128	0,2491	C41H74NO7P
PE(O-36:6)	720,4974	720,4957	2,2901	C41H72NO7P
PE(P-37:4)/PE(O-37:5)	736,5287	736,5285	0,2715	C42H76NO7P
PE(P-37:5)/PE(O-37:6)	734,5130	734,5131	-0,1361	C42H74NO7P
PE(P-37:6)/PE(O-37:7)	732,4974	732,4960	1,8430	C42H72NO7P
PE(O-38:2)	756,5913	756,5902	1,5332	C43H84NO7P
PE(O-38:5)	750,5443	750,5429	1,9453	C43H78NO7P
PE(O-38:6)	748,5287	748,5286	0,1737	C43H76NO7P
PE(O-38:7)	746,5130	746,5133	-0,4153	C43H74NO7P

PE(O-40:2)	784,6226	784,6216	1,2490	C45H88NO7P
PE(O-40:4)	780,5913	780,5892	2,7415	C45H84NO7P
PE(O-40:7)	774,5443	774,5428	1,9624	C45H78NO7P
PE(O-42:7)	802,5756	802,5721	4,4108	C47H82NO7P
PE (32:0)	690,5079	690,5045	4,9384	C37H74NO8P
PE (32:2)	686,4766	686,4755	1,6024	C37H70NO8P
PE (34:0)	718,5392	718,5373	2,6164	C39H78NO8P
PE (34:1)	716,5236	716,5228	1,1863	C39H76NO8P
PE (34:2)	714,5079	714,5085	-0,8537	C39H74NO8P
PE (34:3)	712,4923	712,4936	-1,7965	C39H72NO8P
PE (34:4)	710,4766	710,4778	-1,6327	C39H70NO8P
PE (36:1)	744,5549	744,5545	0,4969	C41H80NO8P
PE (36:2)	742,5392	742,5393	-0,1751	C41H78NO8P
PE (36:3)	740,5236	740,5249	-1,7285	C41H76NO8P
PE (36:4)	738,5079	738,5052	3,6289	C41H74NO8P
PE (36:5)	736,4923	736,4891	4,3992	C41H72NO8P
PE (38:2)	770,5705	770,5693	1,5705	C43H81NO8P
PE (38:4)	766,5392	766,5356	4,6964	C43H78NO8P
PE (38:5)	764,5236	764,5225	1,4388	C43H76NO8P
PE (38:6)	762,5079	762,5076	0,3148	C43H74NO8P
PE (38:7)	760,4923	760,4924	-0,1821	C43H71NO8P
PE (40:3)	796,5862	796,5855	0,8536	C45H84NO8P
PE (40:4)	794,5705	794,5692	1,6751	C45H81NO8P
PE (40:5)	792,5549	792,5551	-0,3028	C45H80NO8P
PE (40:6)	790,5392	790,5377	1,9227	C45H78NO8P
PE (40:7)	788,5236	788,5246	-1,2428	C45H76NO8P
PE (40:8)	786,5079	786,5100	-2,7209	C45H74NO8P
PE (42:7)	816,5549	816,5540	1,1144	C47H80NO8P
PG (32:0)	721,5025	721,5018	1,0118	C38H75O10P
PG (32:1)	719,4869	719,4841	3,8222	C38H73O10P
PG (34:1)	747,5182	747,5161	2,7825	C40H77O10P
PG (34:2)	745,5025	745,5039	-1,8109	C40H75O10P
PG (34:3)	743,4869	743,4857	1,5602	C40H73O10P
PG (36:1)	775,5495	775,5504	-1,0960	C42H81O10P
PG (36:2)	773,5338	773,5317	2,7536	C42H79O10P
PG (38:2)	801,5651	801,5657	-0,7610	C44H83O10P
PG (38:3)	799,5495	799,5470	3,1268	C44H81O10P
PG (38:4)	797,5338	797,5348	-1,2288	C44H79O10P
PG (38:5)	795,5182	795,5154	3,5323	C44H77O10P
PG (38:6)	793,5025	793,5015	1,2224	C44H75O10P
PG (38:7)	791,4869	791,4876	-0,8465	C44H73O10P

PG (40:7)	819,5182	819,5156	3,2092	C46H77O10P
PI (34:0)	837,5499	837,5469	3,5808	C43H82O13P
PI (36:0)	865,5812	865,5774	4,4478	C45H86O13P
PI (36:1)	863,5655	863,5646	0,9855	C45H84O13P
PI (36:3)	859,5342	859,5323	2,2541	C45H80O13P
PI (38:2)	889,5812	889,5783	3,3081	C47H86O13P
PI (38:3)	887,5655	887,5646	1,0009	C47H84O13P
PI (40:3)	915,5968	915,5953	1,6304	C49H88O13P
PI (40:4)	913,5812	913,5802	1,1018	C49H86O13P
PI (40:5)	911,5655	911,5654	0,1227	C49H84O13P
PI (O-36:1)	849,5862	849,5877	-1,7766	C45H87O12P
PI (O-36:2)	847,5706	847,5711	-0,5183	C45H85O12P
PI (O-38:5)	869,5549	869,5551	-0,2513	C47H83O12P
PI (O-39:5)	883,5706	883,5703	0,3622	C48H85O12P
PI (O-39:6)	881,5549	881,5568	-2,1439	C48H83O12P
PI (O-40:5)	897,5862	897,5859	0,3119	C49H87O12P
PI (O-40:6)	895,5706	895,5714	-0,8151	C49H85O12P
PI (32:1)	807,5029	807,5022	0,8700	C41H77O13P
PI (34:1)	835,5342	835,5333	1,0067	C43H81O13P
PI (34:2)	833,5186	833,5189	-0,3206	C43H79O13P
PI (36:2)	861,5499	861,5498	0,1307	C45H83O13P
PI (36:4)	857,5186	857,5169	1,9587	C45H79O13P
PI (38:1)	891,5968	891,5932	4,0131	C47H89O13P
PI (38:4)	885,5499	885,5501	-0,2328	C47H83O13P
PI (38:5)	883,5342	883,5344	-0,2558	C47H81O13P
PI (38:6)	881,5186	881,5191	-0,5963	C47H79O13P
PI (40:6)	909,5499	909,5502	-0,3050	C49H83O13P
PI (40:7)	907,5342	907,5349	-0,7383	C49H81O13P
PI (42:5)	939,5968	939,5969	-0,0639	C51H89O13P
PS (34:1)	760,5134	760,5138	-0,4734	C40H76NO10P
**Lipid species identified as [M+H]+**				
CAR (16:0)	400,3421	400,3408	3,1973	C23H45NO4
CAR (18:0)	428,3734	428,3723	2,5679	C25H49NO4
CAR (18:1)	426,3578	426,3568	2,4393	C25H47NO4
Cer (38:1);2O	594,5820	594,5813	1,1268	C38H75NO3
Cer (40:1);2O	622,6133	622,6126	1,1725	C40H79NO3
Cer (42:1);3O	648,6289	648,6295	-0,8479	C42H83NO4
Cer(34:1);2O	520,5088	520,5073	2,8242	C34H67NO3
Cer(42:1);2O	650,6426	650,6446	-2,9970	C42H83NO3
DG (24:1)	477,3550	477,3571	-4,4202	C27H50O5
DG (26:5)	497,3237	497,3241	-0,8043	C29H46O5

DG (28:2)	531,4020	531,4013	1,2796	C31H56O5
DG (28:3)	529,3863	529,3862	0,1700	C31H54O5
DG (32:8)	575,3707	575,3715	-1,3904	C35H52O5
DG (33:8)	589,3863	589,3875	-2,0360	C36H54O5
DG (34:7)	605,4176	605,4177	-0,1982	C37H58O5
DG (34:8)	603,4020	603,4026	-1,0275	C37H56O5
DG (36:7)	633,4489	633,4497	-1,2471	C39H62O5
DG (38:7)	661,4802	661,4835	-4,8981	C41H66O5
DG (38:9)	657,4489	657,4503	-2,1142	C41H62O5
DG (40:10)	683,4646	683,4653	-1,0242	C43H64O5
DG (42:10)	711,4959	711,4960	-0,1687	C45H68O5
DG (44:11)	737,5115	737,5098	2,2644	C47H70O5
DG (46:11)	765,5428	765,5433	-0,6662	C49H74O5
DG (46:12)	763,5272	763,5266	0,7989	C49H72O5
DG (48:11)	793,5741	793,5743	-0,2520	C51H78O5
DG (O-30:1)	542,5143	542,5151	-1,5852	C33H64O4
DG (O-32:1)	570,5456	570,5449	1,1743	C35H68O4
DG (O-34:1)	598,5769	598,5759	1,6205	C37H72O4
DG (30:3)	557,4176	557,4175	0,1794	C33H58O5
DG (44:10)	739,5272	739,5255	2,3123	C47H72O5
DG (44:9)	741,5428	741,5429	-0,1483	C47H74O5
Hex/Cer (34:1);2O	700,5722	700,5728	-0,8707	C40H77NO8
Hex/Cer (40:1);2O	784,6661	784,6646	1,8734	C46H89NO8
Hex/Cer (42:1);2O	812,6974	812,6974	0,0000	C48H93NO8
Hex/Cer (42:2);2O	810,6795	810,6817	-2,7138	C48H91NO8
LPC (14:0)	468,3085	468,3089	-0,9182	C22H47NO7P
LPC (20:0)	552,4024	552,4027	-0,4526	C28H59NO7P
LPC (20:1)	550,3867	550,3860	1,3263	C28H57NO7P
LPC (20:4)	544,3398	544,3383	2,6821	C28H51NO7P
LPC (22:0)	580,4337	580,4328	1,5850	C30H63NO7P
LPC (22:6)	568,3398	568,3406	-1,4076	C30H51NO7P
LPC (16:0)	496,3398	496,3396	0,4797	C24H51NO7P
LPC (16:1)	494,3241	494,3235	1,1228	C24H49NO7P
LPC (18:0)	524,3711	524,3712	-0,1430	C26H53NO7P
LPC (18:1)	522,3554	522,3553	0,3014	C26H53NO7P
LPC (18:2)	520,3398	520,3391	1,3945	C26H51NO7P
LPC(O-16:0)	482,3605	482,3599	1,1621	C24H53NO6P
LPC(O-18:0)	510,3918	510,3918	-0,0704	C26H57NO6P
LPC(O-18:1)	508,3761	508,3759	0,3272	C26H55NO6P
PC (38:0)	818,6633	818,6629	0,5252	C46H93NO8P
PC(O-32:2)	716,5589	716,5589	0,0000	C40H79NO7P

PC(O-32:3)	714,5432	714,5429	0,4338	C40H77NO7P
PC(O-38:1)	802,6684	802,6722	-4,7093	C46H93NO7P
PC(O-40:3)	826,6684	826,6674	1,2581	C48H93NO7P
PC(O-40:7)	818,6058	818,6060	-0,3054	C48H84NO7P
PC(O-41:7)	832,6215	832,6210	0,6245	C49H87NO7P
PC(O-42:5)	850,6684	850,6659	2,9506	C50H93NO7P
PC(P-32:1)	716,5589	716,5582	1,0448	C40H79NO7P
PC (30:0)	706,5381	706,5379	0,2205	C38H77NO8P
PC (30:1)	704,5225	704,5230	-0,6417	C38H75NO8P
PC (32:0)	734,5694	734,5691	0,4220	C40H81NO8P
PC (32:1)	732,5538	732,5524	1,8605	C40H79NO8P
PC (32:2)	730,5381	730,5374	0,9981	C40H77NO8P
PC (34:0)	762,6007	762,5978	3,8174	C42H85NO8P
PC (34:1)	760,5851	760,5835	2,0773	C42H83NO8P
PC (34:2)	758,5694	758,5693	0,1895	C42H81NO8P
PC (34:3)	756,5538	756,5518	2,6261	C42H79NO8P
PC (34:4)	754,5381	754,5355	3,4565	C42H77NO8P
PC (34:5)	752,5225	752,5195	3,9293	C42H75NO8P
PC (36:1)	788,6164	788,6153	1,3525	C44H87NO8P
PC (36:2)	786,6007	786,5997	1,3270	C44H85NO8
PC (36:3)	784,5851	784,5840	1,4479	C44H83NO8P
PC (36:4)	782,5694	782,5675	2,3772	C44H81NO8P
PC (36:5)	780,5538	780,5514	3,1311	C44H79NO8P
PC (36:6)	778,5381	778,5370	1,4367	C44H77NO8P
PC (38:1)	816,6477	816,6463	1,7474	C46H91NO8P
PC (38:2)	814,6320	814,6307	1,6353	C46H89NO8P
PC (38:3)	812,6164	812,6138	3,2537	C46H87NO8P
PC (38:4)	810,6007	810,5986	2,6077	C46H85NO8P
PC (38:5)	808,5851	808,5822	3,5499	C46H83NO8P
PC (38:6)	806,5694	806,5681	1,6244	C46H81NO8P
PC (38:7)	804,5538	804,5516	2,6849	C46H79NO8P
PC (38:8)	802,5381	802,5352	3,6661	C46H77NO8P
PC (40:2)	842,6633	842,6606	3,2352	C48H93NO8P
PC (40:3)	840,6477	840,6456	2,5039	C48H91NO8P
PC (40:4)	838,6320	838,6300	2,3922	C48H89NO8P
PC (40:5)	836,6164	836,6137	3,1877	C48H87NO8P
PC (40:6)	834,6007	834,5990	2,0365	C48H85NO8P
PC (40:7)	832,5851	832,5837	1,6831	C48H83NO8P
PC (40:8)	830,5694	830,5665	3,4466	C48H81NO8P
PC (40:9)	828,5538	828,5502	4,3388	C48H79NO8P
PC (42:1)	872,7103	872,7101	0,2445	C50H99NO8P

PC (42:10)	854,5694	854,5665	3,4108	C50H81NO8P
PC (42:2)	870,6946	870,6936	1,1306	C50H97NO8P
PC (42:5)	864,6477	864,6454	2,6383	C50H91NO8P
PC (42:6)	862,6320	862,6302	2,1415	C50H89NO8P
PC (42:7)	860,6164	860,6141	2,6802	C50H87NO8P
PC (44:4)	894,6946	894,6933	1,4078	C52H97NO8P
PC (44:7)	888,6477	888,6456	2,4123	C52H91NO8P
PC (44:9)	884,6164	884,6126	4,2600	C52H87NO8P
PC(O-28:0)	664,5276	664,5275	0,1122	C36H75NO7P
PC(O-30:0)	692,5589	692,5584	0,7240	C38H79NO7P
PC(O-30:1)	690,5432	690,5429	0,3970	C38H77NO7P
PC(O-32:0)	720,5902	720,5892	1,4492	C40H83NO7P
PC(O-32:1)	718,5745	718,5740	0,7932	C40H81NO7P
PC(O-34:0)	748,6215	748,6184	4,1549	C42H87NO7P
PC(O-34:1)	746,6058	746,6050	1,0745	C42H85NO7P
PC(O-34:2)	744,5902	744,5891	1,5370	C42H83NO7P
PC(O-34:3)	742,5745	742,5714	4,1557	C42H81NO7P
PC(O-34:4)	740,5589	740,5566	3,0419	C42H79NO7P
PC(O-36:1)	774,6371	774,6357	1,7989	C44H89NO7P
PC(O-36:2)	772,6215	772,6199	2,0724	C44H87NO7P
PC(O-36:3)	770,6058	770,6028	3,8154	C44H85NO7P
PC(O-36:4)	768,5902	768,5869	4,2497	C44H83NO7P
PC(O-36:5)	766,5745	766,5721	3,1605	C44H81NO7P
PC(O-38:2)	800,6528	800,6502	3,2172	C46H91NO7P
PC(O-38:3)	798,6371	798,6340	3,7989	C46H89NO7P
PC(O-38:4)	796,6215	796,6188	3,4682	C46H87NO7P
PC(O-38:5)	794,6058	794,6039	2,3190	C46H85NO7P
PC(O-38:6)	792,5902	792,5892	1,3155	C46H83NO7P
PC(O-40:4)	838,6320	838,6300	2,3922	C48H89NO8P
PC(O-40:5)	822,6371	822,6358	1,5560	C48H89NO7P
PC(O-40:6)	820,6215	820,6198	2,1569	C48H87NO7P
PC(O-42:6)	848,6528	848,6520	0,9104	C50H91NO7P
PC(P-32:1)	716,5589	716,5582	1,0448	C40H79NO7P
PC(P-40:6)	818,6058	818,6043	1,8247	C48H85NO7P
PC(P-42:6)	846,6371	846,6352	2,2283	C50H89NO7P
SM (32:1);2O	675,5436	675,5436	-0,0888	C37H75N2O6P
SM (32:7);2O	663,4497	663,4508	-1,6429	C37H63N2O6P
SM (34:7);3O	707,4759	707,4776	-2,4170	C39H67N2O7P
SM (36:0);2O	733,6218	733,6202	2,2491	C41H85N2O6P
SM (36:2);3O	745,5854	745,5853	0,1609	C41H81N2O7P
SM (38:0);2O	761,6531	761,6521	1,2867	C43H89N2O6P

SM (38:0);3O	777,6480	777,6481	-0,1157	C43H89N2O7P
SM (38:2);3O	773,6167	773,6185	-2,3267	C43H85N2O7P
SM (38:3);3O	771,6011	771,6042	-4,0565	C43H83N2O7P
SM (40:7);2O	775,5749	775,5734	1,9212	C45H79N2O6P
SM (42:1);3O	831,6950	831,6976	-3,0781	C47H95N2O7P
SM (42:2);3O	829,6793	829,6800	-0,8196	C47H93N2O7P
SM (42:3);3O	827,6637	827,6667	-3,6247	C47H91N2O7P
SM (42:4);2O	809,6531	809,6507	2,9395	C47H89N2O6P
SM (42:5);2O	807,6375	807,6389	-1,7458	C47H87N2O6P
SM (42:6);3O	821,6167	821,6142	3,0428	C47H85N2O7P
SM (42:7);3O	819,6011	819,6021	-1,2567	C47H83N2O7P
SM (44:1);3O	859,7263	859,7272	-1,0236	C49H99N2O7P
SM (52:2);3O	969,8358	969,8362	-0,4331	C57H113N2O7P
SM (52:3);3O	967,8202	967,8187	1,5085	C57H111N2O7P
SM (58:3);3O	1051,9141	1051,9148	-0,6940	C63H123N2O7P
SM (34:0);2O	705,5899	705,5889	1,4283	C39H81N2O6P
SM (34:1);2O	703,5738	703,5751	-1,8554	C39H79N2O6P
SM (36:1);2O	731,6060	731,6026	4,6511	C41H83N2O6P
SM (36:2);2O	729,5908	729,5902	0,8292	C41H81N2O6P
SM (36:3);2O	727,5724	727,5748	-3,2490	C41H80N2O6P
SM (38:1);2O	759,6374	759,6390	-2,0799	C43H88N2O6P
SM (40:1);2O	787,6679	787,6694	-1,8070	C45H91N2O6P
SM (40:2);2O	785,6525	785,6503	2,7293	C45H89N2O6P
SM (42:1);2O	815,6984	815,6985	-0,0492	C47H95N2O6P
SM (42:2);2O	813,6834	813,6825	1,1473	C47H93N2O6P
SM (42:3);2O	811,6675	811,6649	3,2953	C47H91N2O6P

Supplementary Table S4. The 273 lipid species showing significant variation between conditions, (* if q < 0.05, ** if q < 0.01, *** if q < 0.001 and if **** q < 0.0001). Label of the lipid species: AAA (xx:i) (AAA=lipid class abbreviation; xx=number of carbon atoms; i=number of double bonds). The 'O-' prefix is used for plasmanyl species to indicate the presence of an alkyl ether substituent, and the 'P-' prefix is used for plasmenyl species to indicate the alk-1-enyl ether substituent._

Lipid class abbreviations: acyl-carnitines (CAR), di-acylglycerols (DG), ceramides (Cer), hexosylceramides (HexCer), sphingomyelins (SM). phosphatidylcholines (PC), phosphatidylethanolamines (PE), phosphatidylinositols (PI), phosphatidylglycerols (PG), phosphatidylserine (PS), cardiolipins (CL), lysophosphatidylcholine (LPC), and lysophosphatidylethanolamines (LPE)

Lipid species	q-value	Statistical significance level
**CL(62:1)**	1,91E-04	***
**CL(66:0)**	9,00E-03	**
**CL(68:2)**	2,64E-02	*
**CL(68:3)**	4,89E-03	**
**CL(68:4)**	5,86E-04	***
**CL(70:4)**	2,56E-02	*
**CL(70:7)**	2,64E-04	***
**CL(72:1)**	2,66E-02	*
**CL(72:4)**	6,83E-04	***
**CL(72:5)**	3,87E-03	**
**CL(72:6)**	8,11E-03	**
**CL(72:7)**	8,21E-03	**
**CL(74:1)**	6,23E-03	**
**CL(74:3)**	5,35E-04	***
**CL(74:7)**	1,15E-03	**
**CL(74:8)**	2,00E-03	**
**CL(74:9)**	2,22E-02	*
**CL(76:1)**	1,39E-03	**
**CL(76:7)**	2,78E-03	**
**CL(78:1)**	1,20E-04	***
**CL(78:13)**	2,01E-02	*
**CL(78:3)**	5,09E-04	***
**CL(78:7)**	5,88E-05	****
**CL(80:7)**	1,56E-04	***
**CL(82:3)**	5,88E-05	****
**CL(82:5)**	5,88E-05	****
**CL(84:11)**	5,88E-05	****

**LPE(22:3)**	7,02E-05	****
**LPE(16:1)**	5,88E-05	****
**LPE(18:0)**	3,42E-04	***
**LPE(18:1)**	2,50E-03	**
**LPE(18:2)**	3,37E-03	**
**LPE(20:1)**	1,77E-04	***
**LPE(20:3)**	1,84E-04	***
**LPE(20:4)**	2,90E-04	***
**LPE(20:5)**	5,88E-05	****
**LPE(22:4)**	8,97E-05	****
**LPE(22:5)**	1,84E-04	***
**LPE(22:6)**	6,29E-05	****
**LPE(O-16:0)**	1,09E-04	***
**LPE(O-16:1)**	9,08E-05	****
**LPE(O-18:0)**	5,88E-05	****
**LPE(O-20:1)**	5,88E-05	****
**LPE(P-18:0)**	5,88E-05	****
**LPE(O-18:1)**	2,00E-03	**
**PE(30:1)**	1,65E-04	***
**PE(O-32:2)**	1,42E-04	***
**PE(O-34:2)**	1,10E-04	***
**PE(O-34:3)**	1,77E-04	***
**PE(O-36:1)**	5,88E-05	****
**PE(O-36:2)**	5,88E-05	****
**PE(O-36:3)**	1,49E-04	***
**PE(O-36:5)**	5,88E-05	****
**PE(O-36:6)**	5,14E-04	***
**PE(P-37:4)**	5,88E-05	****
**PE(P-37:5)**	9,22E-05	****
**PE(P-37:6)**	5,88E-05	****
**PE(O-38:2)**	5,88E-05	****
**PE(O-38:5)**	6,35E-05	****
**PE(O-38:6)**	1,65E-04	***
**PE(O-38:7)**	5,88E-05	****
**PE(O-40:2)**	5,89E-05	****
**PE(O-40:4)**	5,88E-05	****
**PE(O-40:7)**	1,84E-04	***
**PE(O-42:7)**	5,89E-05	****
**PE(32:0)**	3,23E-03	**

**PE(34:1)**	2,44E-03	**
**PE(34:3)**	4,52E-03	**
**PE(34:4)**	1,08E-04	***
**PE(36:1)**	7,97E-05	****
**PE(36:2)**	7,59E-05	****
**PE(36:4)**	5,49E-03	**
**PE(38:2)**	4,27E-03	**
**PE(38:4)**	6,07E-05	****
**PE(38:5)**	1,04E-02	*
**PE(38:6)**	3,26E-04	***
**PE(40:3)**	4,78E-04	***
**PE(40:4)**	5,88E-05	****
**PE(40:5)**	5,88E-05	****
**PE(40:6)**	5,89E-05	****
**PE(40:7)**	5,76E-03	**
**PE(42:7)**	2,10E-03	**
**PG(32:0)**	5,88E-05	****
**PG(32:1)**	5,88E-05	****
**PG(34:1)**	1,10E-04	***
**PG(34:2)**	3,47E-04	***
**PG(34:3)**	1,87E-04	***
**PG(36:1)**	8,75E-05	****
**PG(36:2)**	1,81E-02	*
**PG(38:2)**	5,88E-05	****
**PG(38:3)**	5,89E-05	****
**PG(38:4)**	5,63E-04	***
**PG(38:5)**	8,65E-03	**
**PG(38:7)**	1,01E-04	***
**PG(40:7)**	1,95E-02	*
**PI(34:0)**	5,88E-05	****
**PI(36:0)**	5,88E-05	****
**PI(36:1)**	5,88E-05	****
**PI(36:3)**	2,84E-04	***
**PI(38:2)**	3,77E-04	***
**PI(38:3)**	2,37E-04	***
**PI(40:3)**	5,88E-05	****
**PI(40:4)**	5,88E-05	****
**PI(40:5)**	5,88E-05	****
**PI(O-36:1)**	5,88E-05	****

**PI(O-36:2)**	6,86E-04	***
**PI(O-38:5)**	1,87E-04	***
**PI(O-39:5)**	1,32E-04	***
**PI(O-39:6)**	2,22E-02	*
**PI(O-40:5)**	5,88E-05	****
**PI(O-40:6)**	5,88E-05	****
**PI(32:1)**	1,99E-04	***
**PI(34:1)**	1,43E-04	***
**PI(36:2)**	1,07E-03	**
**PI(36:4)**	5,88E-05	****
**PI(38:1)**	5,88E-05	****
**PI(38:4)**	5,88E-05	****
**PI(38:5)**	5,89E-05	****
**PI(38:6)**	1,04E-04	***
**PI(40:6)**	5,88E-05	****
**PI(40:7)**	5,88E-05	****
**PI(42:5)**	1,66E-03	**
**CAR(16:0)**	5,88E-05	****
**CAR(18:0)**	9,19E-05	****
**CAR(18:1)**	5,88E-05	****
**Cer(38:1);2O**	2,57E-04	***
**Cer(40:1);2O**	5,88E-05	****
**Cer(42:1);3O**	5,88E-05	****
**Cer(34:1);2O**	5,88E-05	****
**Cer(42:1);2O**	5,88E-05	****
**DG(24:1)**	2,27E-02	*
**DG(28:2)**	7,60E-03	**
**DG(28:3)**	3,44E-03	**
**DG(32:8)**	1,37E-03	**
**DG(33:8)**	4,78E-04	***
**DG(34:7)**	2,93E-03	**
**DG(34:8)**	3,67E-02	*
**DG(36:7)**	1,52E-03	**
**DG(38:7)**	1,09E-03	**
**DG(40:10)**	3,90E-02	*
**DG(44:11)**	6,86E-04	***
**DG(46:12)**	5,70E-03	**
**DG(48:11)**	2,40E-04	***
**DG(O-30:1)**	7,16E-03	**

**DG(O-32:1)**	6,98E-03	**
**DG(O-34:1)**	1,13E-02	*
**DG(30:3)**	5,72E-04	***
**DG(44:10)**	6,52E-03	**
**DG(44:9)**	3,39E-04	***
**HexCer(34:1);2O**	1,03E-04	***
**HexCer(40:1);2O**	6,29E-05	****
**HexCer(42:1);2O**	5,88E-05	****
**HexCer(42:2);2O**	7,39E-05	****
**LPC(14.0)**	2,22E-04	***
**LPC(20.0)**	7,69E-05	****
**LPC(20:1)**	5,36E-04	***
**LPC(20:4)**	3,09E-03	**
**LPC(22:0)**	2,46E-04	***
**LPC(22:6)**	1,09E-04	***
**LPC(16:0)**	5,36E-04	***
**LPC(16:1)**	1,92E-02	*
**LPC(18:0)**	6,47E-05	****
**LPC(18:1)**	1,77E-02	*
**LPC(O-16:0)**	1,80E-04	***
**LPC(O-18:0)**	1,00E-04	***
**LPC(O-18:1)**	1,81E-03	**
**PC(38:0)**	5,88E-05	****
**PC(O-32:2)**	8,02E-04	***
**PC(O-32:3)**	5,88E-05	****
**PC(O-38:1)**	5,88E-05	****
**PC(O-40:3)**	3,14E-04	***
**PC(O-40:7)**	5,88E-05	****
**PC(O-41:7)**	3,73E-04	***
**PC(O-42:5)**	9,01E-04	***
**PC(P-32:1)**	8,02E-04	***
**PC(30:0)**	1,87E-04	***
**PC(30:1)**	3,25E-03	**
**PC(32:0)**	6,86E-03	**
**PC(32:1)**	2,68E-04	***
**PC(32:2)**	1,07E-04	***
**PC(34:0)**	3,66E-02	*
**PC(34:1)**	1,27E-02	*
**PC(34:2)**	2,77E-04	***

**PC(34:3)**	7,48E-03	**
**PC(34:4)**	5,89E-05	****
**PC(34:5)**	5,88E-05	****
**PC(36:1)**	5,88E-05	****
**PC(36:2)**	3,36E-03	**
**PC(36:3)**	2,75E-04	***
**PC(36:4)**	1,65E-04	***
**PC(36:5)**	5,88E-05	****
**PC(36:6)**	1,72E-02	*
**PC(38:1)**	5,88E-05	****
**PC(38:2)**	5,88E-05	****
**PC(38:3)**	5,88E-05	****
**PC(38:4)**	5,88E-05	****
**PC(38:5)**	6,04E-05	****
**PC(38:6)**	4,07E-04	***
**PC(38:7)**	1,61E-03	**
**PC(38:8)**	5,97E-04	***
**PC(40:2)**	5,88E-05	****
**PC(40:3)**	5,88E-05	****
**PC(40:4)**	5,88E-05	****
**PC(40:5)**	5,88E-05	****
**PC(40:6)**	5,88E-05	****
**PC(40:7)**	5,88E-05	****
**PC(40:8)**	3,96E-04	***
**PC(40:9)**	5,88E-05	****
**PC(42:1)**	1,64E-04	***
**PC(42:10)**	6,29E-05	****
**PC(42:2)**	7,13E-05	****
**PC(42:5)**	5,88E-05	****
**PC(42:6)**	5,88E-05	****
**PC(42:7)**	7,97E-05	****
**PC(44:4)**	6,29E-05	****
**PC(44:7)**	6,35E-05	****
**PC(44:9)**	5,88E-05	****
**PC(O-28:0)**	1,49E-04	***
**PC(O-30:0)**	1,34E-04	***
**PC(O-30:1)**	6,29E-05	****
**PC(O-32:0)**	5,72E-04	***
**PC(O-32:1)**	5,89E-05	****

**PC(O-34:0)**	7,69E-05	****
**PC(O-34:1)**	1,43E-04	***
**PC(O-34:2)**	8,75E-05	****
**PC(O-34:3)**	1,05E-02	*
**PC(O-34:4)**	9,96E-05	****
**PC(O-36:1)**	5,88E-05	****
**PC(O-36:2)**	2,00E-04	***
**PC(O-36:3)**	2,24E-04	***
**PC(O-36:4)**	3,50E-04	***
**PC(O-36:5)**	3,42E-04	***
**PC(O-38:2)**	5,89E-05	****
**PC(O-38:3)**	5,88E-05	****
**PC(O-38:4)**	5,88E-05	****
**PC(O-38:5)**	6,29E-05	****
**PC(O-38:6)**	5,88E-05	****
**PC(O-40:4)**	5,88E-05	****
**PC(O-40:5)**	5,89E-05	****
**PC(O-40:6)**	1,05E-04	***
**PC(O-42:6)**	1,82E-02	*
**PC(P-40:6)**	9,97E-05	****
**PC(P-42:6)**	7,69E-05	****
**SM(32:1);2O**	4,44E-03	**
**SM(36:0);2O**	1,04E-04	***
**SM(36:2(3O**	3,03E-04	***
**SM(38:0);2O**	1,27E-04	***
**SM(38:0);3O**	5,88E-05	****
**SM(38:2);3O**	5,88E-05	****
**SM(38:3);3O**	1,17E-04	***
**SM(40:7);2O**	1,10E-04	***
**SM(42:1);3O**	1,21E-03	**
**SM(42:2);3O**	2,71E-03	**
**SM(42:3);3O**	1,06E-02	*
**SM(42:4);2O**	2,40E-04	***
**SM(42:5);2O**	6,29E-05	****
**SM(42:6);3O**	6,29E-05	****
**SM(42:7);3O**	2,28E-03	**
**SM(44:1);3O**	3,04E-03	**
**SM(52:2);3O**	5,88E-05	****
**SM(52:3);3O**	5,89E-05	****

**SM(58:3);3O**	1,23E-04	***
**SM(32:1);2O**	4,25E-03	**
**SM(34:0);2O**	1,68E-04	***
**SM(36:1);2O**	9,51E-05	****
**SM(36:2);2O**	5,88E-05	****
**SM(36:3);2O**	1,04E-04	***
**SM(38:1);2O**	2,18E-04	***
**SM(40:1);2O**	1,04E-04	***
**SM(40:2);2O**	1,77E-04	***
**SM(42:1);2O**	1,04E-03	**
**SM(42:2);2O**	6,57E-03	**
**SM(42:3);2O**	7,22E-03	**

Supplementary Table S5. Metabolite species identified by LC-MS in RAW 264.7 macrophages supplemented with omega-3 and omega-6 phopholips alone and in the presence of LPS (mass error < 0.05 Da).

**Metabolite species identified as $[M+H]^+$**				
**1-Methyladenosine**	282,1197	282,1181	C11H16N5O4	0,0016
**1,2-Cyclohexanedione**	113,0597	113,0710	C6H8O2	-0,0113
**1,4-Cyclohexanedione**	113,0597	113,1073	C6H8O2	-0,0476
**2-Aminoacetophenone**	136,0757	136,0967	C8H9NO	-0,0210
**2-Methyl-3-hydroxybutyric acid**	119,0703	119,0853	C5H10O3	-0,0151
**2-Palmitoylglycerol**	331,2843	331,2829	C19H38O4	0,0013
**3-Hydroxyanthranilic acid**	154,0499	154,0578	C7H7NO3	-0,0079
**3-Methylhistidine**	170,0924	170,1169	C7H11N3O2	-0,0245
**4-Aminophenol**	110,0600	110,0601	C6H7NO	-0,0001
**4-Guanidinobutanoic acid**	146,0924	146,0923	C5H11N3O2	0,0001
**4-Hydroxyproline**	132,0655	132,0653	C5H9NO3	0,0002
**5-Hydroxyindoleacetic acid**	192,0655	192,0609	C10H9NO3	0,0046
**5'-Methylthioadenosine**	298,0968	298,0961	C11H15N5O3S	0,0007
**Adenosine monophosphate**	348,0704	348,0685	C10H14N5O7P	0,0019
**Agmatine**	131,1291	131,1177	C5H14N4	0,0114
**Benzamide**	122,0600	122,0812	C7H7NO	-0,0212
**beta-Alanine**	90,0550	90,0918	C3H7NO2	-0,0369
**Beta-Guanidinopropionic acid**	132,0768	132,0766	C4H9N3O2	0,0001
**Citrulline**	176,1030	176,1027	C6H13N3O3	0,0003
**Creatinine**	114,0662	114,0663	C4H7N3O	-0,0001
**Cyclohexylamine**	100,1121	100,1123	C6H13N	-0,0003
**Cytosine**	112,0505	112,0871	C4H5N3O	-0,0365
**D-Alanyl-D-alanine**	161,0921	161,0919	C6H12N2O3	0,0002

**Dihydrouracil**	115,0502	115,0866	C4H6N2O2	-0,0364
**Glutathione**	308,0911	308,0891	C10H17N3O6S	0,0020
**Glycine**	76,0393	76,0396	C2H5NO2	-0,0002
**Histamine**	112,0869	112,0870	C5H9N3	-0,0001
**Homo-L-arginine**	189,1346	189,1229	C7H16N4O2	0,0117
**Hypoxanthine**	137,0458	137,0456	C5H4N4O	0,0002
**L-Alanine**	90,0550	90,0554	C3H7NO2	-0,0004
**L-Arginine**	175,1190	175,1179	C6H14N4O2	0,0010
**L-Carnitine**	162,1125	162,1122	C7H15NO3	0,0002
**L-Histidine**	156,0768	156,0766	C6H9N3O2	0,0001
**L-Isoleucine**	132,1019	132,1017	C6H13NO2	0,0002
**homo-L-arginine**	147,1128	147,1125	C6H14N2O2	0,0003
**L-Proline**	116,0706	116,0707	C5H9NO2	-0,0001
**L-Threonine**	120,0655	120,0656	C4H9NO3	0,0000
**L-Valine**	118,0863	118,0863	C5H11NO2	-0,0001
**Methioninesulfoxide**	166,0532	166,0530	C5H11NO3S	0,0002
**N-Acetylhistidine**	198,0873	198,0870	C8H11N3O3	0,0003
**N-Cyclohexylformamide**	128,1070	128,1181	C7H13NO	-0,0112
**N-Methyl-L-glutamic acid**	162,0761	162,1124	C6H11NO4	-0,0363
**N-Methylalanine**	104,0706	104,0709	C4H9NO2	-0,0003
**N-Methylproline**	130,0863	130,0861	C6H11NO2	0,0001
**N-Methylthreonine**	134,0812	134,0631	C5H11NO3	0,0181
**N,N-Dimethylarginine**	203,1503	203,1135	C8H18N4O2	0,0368
**N8-Acetylspermidine**	188,1757	188,1747	C9H21N3O	0,0010
**Nicotinamide/ Niacinamide**	123,0553	123,0551	C6H6N2O	0,0002
**L-Ornithine**	133,0972	133,0969	C5H12N2O2	0,0002
**Phenylacetylglycine**	194,0812	194,1171	C10H11NO3	-0,0359
**Propionylcarnitine**	218,1387	218,1379	C10H19NO4	0,0008
**Pyridine**	80,0500	80,0500	C5H5N	0,0001
**S-Adenosyl-homocysteine**	385,1289	385,1273	C14H20N6O5S	0,0016
**S-Adenosyl-methionine**	399,1445	399,1432	C15H22N6O5S	0,0013
**Serine**	106,0499	106,0865	C3H7NO3	-0,0366
**Spermine**	203,2230	203,2227	C10H26N4	0,0003
**Taurine**	126,0219	126,0219	C2H7NO3S	0,0000
**Trimethylamine N-oxide**	76,0757	76,0762	C3H9NO	-0,0005
**Uridine 5'-monophosphate**	325,0432	325,0416	C9H13N2O9P	0,0016
**Urocanate**	139,0502	139,0500	C6H6N2O2	0,0002
**Metabolite species identified as [M - H]-**				
**2-Aminophenol**	108,0455	108,0808	C6H7NO	-0,0353
**2-Butyne-1,4-diol**	85,0295	85,0259	C4H6O2	0,0036
**2-Methylglutaric acid**	145,0506	145,0505	C6H10O4	0,0002

**3-Methyladipic acid**	159,0663	159,0662	C7H12O4	0,0001
**4-Hydroxybenzeneacetonitrile**	132,0455	132,0550	C8H7NO	-0,0095
**5-Methylcytidine**	256,0939	256,0587	C10H15N3O5	0,0352
**Acetylglycine**	116,0353	116,0446	C4H7NO3	-0,0093
**Adenine**	134,0472	134,0343	C5H5N5	0,0129
**Adipic acid**	145,0506	145,0502	C6H10O4	0,0004
**Allantoin**	157,0367	157,0510	C4H6N4O3	-0,0143
**cis-Aconitic acid**	173,0092	173,0079	C6H6O6	0,0013
**Citraconic acid**	129,0193	129,0176	C5H6O4	0,0017
**Citramalic acid**	147,0299	147,0281	C5H8O5	0,0018
**Citric acid**	191,0197	191,0175	C6H8O7	0,0023
**Cytidine**	242,0783	242,1034	C9H13N3O5	-0,0251
**Erythronolactone**	117,0193	117,0178	C4H6O4	0,0016
**Glutaric acid**	131,0349	131,0349	C5H8O4	0,0000
**Hydroxyphenyllactic acid**	181,0506	181,0510	C9H10O4	-0,0003
**Hydroxypropanoic acid**	89,0244	89,0229	C3H6O3	0,0015
**Indole-3-carboxaldehyde**	144,0454	144,0554	C9H7NO	-0,0100
**Inosine**	267,0735	267,0992	C10H12N4O5	-0,0258
**L-Aspartate**	132,0302	132,0279	C4H7NO4	0,0023
**L-Glutamate**	146,0459	146,0442	C5H9NO4	0,0017
**L-Glutamine**	145,0619	145,0602	C5H10N2O3	0,0017
**Maleic acid**	115,0037	115,0018	C4H4O4	0,0019
**Malic acid**	133,0143	133,0122	C4H6O5	0,0021
**Methylsuccinic acid**	131,0349	131,0441	C5H8O4	-0,0092
**N-Acetyl-L-alanine**	130,0510	130,0395	C5H9NO3	0,0114
**N-Acetylmannosamine**	220,0827	220,0997	C8H15NO6	-0,0170
**N-Acetylserine**	146,0459	146,0438	C5H9NO4	0,0021
**N6-Acetyl-L-lysine**	187,1088	187,0961	C8H16N2O3	0,0127
**Phosphoric acid**	96,9696	96,9585	H3O4P	0,0111
**Porphobilinogen**	225,0880	225,0889	C10H14N2O4	-0,0009
**Propionic acid**	73,0295	73,0277	C3H6O2	0,0018
**Pyroglutamic acid**	128,0353	128,0084	C5H7NO3	0,0269
**Pyrrole-2-carboxylic acid**	110,0248	110,0344	C5H5NO2	-0,0096
**Succinic acid**	117,0193	117,0176	C4H6O4	0,0018
**Threonic acid**	135,0299	135,0182	C4H8O5	0,0117
**Uracil**	111,0195	111,0067	C4H4N2O2	0,0127
**Uridine diphosphate glucose**	565,0477	565,0436	C15H24N2O17P2	0,0041
**Uridine diphosphate-N-acetylglucosamine**	606,0742	606,0719	C17H27N3O17P2	0,0023
**Metabolite species identified as $[M]^+$**				
**Choline**	104,1065	104,1072	C5H14NO	-0,0008

**L-Acetylcarnitine**	204,1225	204,1227	C9H18NO4	-0,0002

Supplementary Table S6 Significant pathways from the pathway analysis associated with ω3_LPS or ω6_LPS supplementation of the RAW 264.7 macrophage cell line.

Pathway	Total Cmpd	Hits	Raw p	-LOG10(p)	FDR	Impact
**Alanine, aspartate and glutamate metabolism**	28	7	0.000146	3.8363	0.003353	0.53446
**Butanoate metabolism**	15	2	0.000181	3.7418	0.003353	0
**D-Glutamine and D-glutamate metabolism**	6	2	0.00047	3.3281	0.004096	0.5
**Nitrogen metabolism**	6	2	0.00047	3.3281	0.004096	0
**Pyrimidine metabolism**	39	6	0.000554	3.2568	0.004096	0.19593
**Amino sugar and nucleotide sugar metabolism**	37	3	0.000914	3.0391	0.005636	0.13622
**Citrate cycle (TCA cycle)**	20	4	0.001336	2.8742	0.007061	0.21948
**Propanoate metabolism**	23	4	0.002729	2.564	0.012623	0
**Glyoxylate and dicarboxylate metabolism**	32	6	0.003176	2.4982	0.013055	0.16138
**Glycerophospholipid metabolism**	36	1	0.004078	2.3896	0.015087	0.02582
**Pentose and glucuronate interconversions**	18	1	0.006074	2.2165	0.016052	0.07812
**Starch and sucrose metabolism**	18	1	0.006074	2.2165	0.016052	0.00974
**Galactose metabolism**	27	1	0.006074	2.2165	0.016052	0.00228
**Ascorbate and aldarate metabolism**	8	1	0.006074	2.2165	0.016052	0
**Primary bile acid biosynthesis**	46	2	0.008062	2.0935	0.019887	0.01516
**Histidine metabolism**	16	6	0.009494	2.0225	0.021955	0.53278
**Aminoacyl-tRNA biosynthesis**	48	13	0.011898	1.9245	0.025896	0
**Glycine, serine and threonine metabolism**	33	6	0.013629	1.8656	0.028014	0.29611
**Selenocompound metabolism**	20	1	0.015696	1.8042	0.029051	0
**Taurine and hypotaurine metabolism**	8	1	0.015711	1.8038	0.029051	0.42857
**Glutathione metabolism**	28	6	0.016488	1.7828	0.029051	0.37144
**Porphyrin and chlorophyll metabolism**	30	4	0.024212	1.616	0.040719	0.08087
**Arginine and proline metabolism**	38	10	0.033884	1.47	0.05451	0.40735

Supplementary Table S7. Significant pathways from the pathway analysis associated with CTRL or CTRL_LPS supplementation of the macrophage RAW 264.7 cell line.

Pathway	Total Cmpd	Hits	Raw p	=-LOG10(p)	FDR	Impact
**Selenocompound metabolism**	20	1	2.77E-10	9.5569	1.03E-08	0
**Nicotinate and nicotinamide metabolism**	15	3	1.94E-09	8.7113	3.38E-08	0.33246
**Glycine, serine and threonine metabolism**	33	6	2.74E-09	8.5615	3.38E-08	0.29611
**Histidine metabolism**	16	6	1.27E-08	7.897	1.17E-07	0.53278
**Valine, leucine and isoleucine biosynthesis**	8	3	2.39E-08	7.6223	1.77E-07	0
**Arginine and proline metabolism**	38	10	3.50E-08	7.4563	2.16E-07	0.40735
**Aminoacyl-tRNA biosynthesis**	48	13	6.00E-08	7.2217	3.17E-07	0
**Porphyrin and chlorophyll metabolism**	30	4	9.34E-08	7.0294	4.32E-07	0.08087
**Cysteine and methionine metabolism**	33	6	3.65E-07	6.4382	1.50E-06	0.34283
**Purine metabolism**	65	5	5.70E-07	6.2439	2.11E-06	0.0883
**Alanine, aspartate and glutamate metabolism**	28	7	1.64E-06	5.7858	5.51E-06	0.53446
**Tryptophan metabolism**	41	2	1.92E-06	5.716	5.93E-06	0.06547
**Glutathione metabolism**	28	6	2.98E-06	5.5263	8.47E-06	0.37144
**beta-Alanine metabolism**	21	7	4.69E-06	5.329	1.16E-05	0.51119
**Pantothenate and CoA biosynthesis**	19	6	4.70E-06	5.3279	1.16E-05	0.05
**Glyoxylate and dicarboxylate metabolism**	32	6	9.06E-06	5.0429	2.10E-05	0.16138
**Pyrimidine metabolism**	39	6	2.39E-05	4.6217	5.20E-05	0.19593
**Arginine biosynthesis**	14	6	5.25E-05	4.2796	0.000108	0.48223
**D-Glutamine and D-glutamate metabolism**	6	2	7.46E-05	4.1275	0.000138	0.5
**Nitrogen metabolism**	6	2	7.46E-05	4.1275	0.000138	0
**Glycerophospholipid metabolism**	36	1	0.00011	3.9569	0.000195	0.02582
**Primary bile acid biosynthesis**	46	2	0.000137	3.8639	0.00023	0.01516
**Citrate cycle (TCA cycle)**	20	4	0.000432	3.3643	0.000695	0.21948
**Valine, leucine and isoleucine degradation**	40	2	0.001086	2.9643	0.001674	0

**Butanoate metabolism**	15	2	0.001239	2.9068	0.001834	0
**Taurine and hypotaurine metabolism**	8	1	0.00664	2.1778	0.009449	0.42857
**Amino sugar and nucleotide sugar metabolism**	37	3	0.008788	2.0561	0.012042	0.13622
**Lysine degradation**	25	2	0.01008	1.9966	0.012081	0.00469
**Pyruvate metabolism**	22	1	0.010122	1.9948	0.012081	0.20684
**Glycolysis / Gluconeogenesis**	26	1	0.010122	1.9948	0.012081	0.10044
**Tyrosine metabolism**	42	1	0.010122	1.9948	0.012081	0
**Propanoate metabolism**	23	4	0.012072	1.9182	0.013959	0
**Biotin metabolism**	10	1	0.015898	1.7987	0.017825	0
